# Associations between dimensions of the social environment and cardiometabolic health outcomes: a systematic review and meta-analysis

**DOI:** 10.1136/bmjopen-2023-079987

**Published:** 2024-08-28

**Authors:** Taymara C Abreu, Joline WJ Beulens, Fleur Heuvelman, Linda J Schoonmade, Joreintje D Mackenbach

**Affiliations:** 1 Department of Epidemiology & Data Science, Amsterdam UMC Location VUmc, Amsterdam, The Netherlands; 2 Amsterdam Public Health Research Institute, Amsterdam, The Netherlands; 3 Upstream Team, Amsterdam UMC, Amsterdam, Netherlands; 4 University Library, Vrije Universiteit Amsterdam, Amsterdam, The Netherlands

**Keywords:** hypertension, epidemiology, social support, cardiac epidemiology, diabetes & endocrinology, systematic review

## Abstract

**Abstract:**

**Objectives:**

The social environment (SE), that is, the social relationships and social context in which groups of people live and interact, is an understudied element of the broader living environment which impacts health. We aim to summarise the available evidence on the associations between SE and cardiometabolic disease (CMD) outcomes.

**Design:**

Systematic review and meta-analysis.

**Data sources:**

PubMed, Scopus and Web of Science Core Collection were searched from inception to 28 February 2024.

**Eligibility criteria:**

We included studies for which determinants were SE factors such as area-level deprivation and social network characteristics and outcomes were type 2 diabetes mellitus and cardiovascular diseases incidence and prevalence.

**Data extraction and synthesis:**

Titles and abstracts and full text were screened in duplicate. Data appraisal and extraction were based on the study protocol published in PROSPERO. Methodological quality was assessed with the Newcastle-Ottawa Scale. We synthesised the data through vote counting and meta-analyses.

**Results:**

From 10 143 records screened, 281 studies reporting 1108 relevant associations are included in this review. Of the 384 associations included in vote counting, 271 (71%) suggested that a worse SE is associated with a higher risk of CMD. 14 meta-analyses based on 180 associations indicated that worse SE was associated with increased odds of CMD outcomes, with 4 of them being statistically significant. For example, more economic and social disadvantage was associated with higher heart failure risk (OR 1.58, 95% CI 1.08 to 1.61; n=18; I^2^=95%). With the exception of two meta-analyses for men, meta-analysed sex-specific associations consistently showed results in the same direction as the overall meta-analyses.

**Conclusion:**

Worse SE seems to be associated with increased odds of CMD outcomes, although certain SE dimensions are underexplored in relation to CMD.

**PROSPERO registration number:**

CRD42021223035.

**STRENGTHS AND LIMITATIONS OF THIS STUDY**

- This systematic review employed a comprehensive scope along with a robust search strategy (eg, using extensive search terms, searching across multiple databases, conducting backward reference checks and applying a time-unrestricted search).
- The broad scope of the search strategy acknowledges the different dimensions of the social environment that cannot be (re)viewed in isolation.
- The decision not to include studies investigating mortality outcomes, while justifiable due to the varying roles of social environment on health and disease, may have potentially limited the depth of insights provided by this review.
- The quality assessment tools used in this review are susceptible to some degree of subjectivity, despite the authors’ efforts to mitigate its impact through piloting the application of the tools and adaptation of guidelines with specific decision rules.
- Due to the challenge of converting and harmonising continuous data into categorical data, measures that were not operationalised as dichotomous or categorical variables were not included in the meta-analysis. In contrast, the vote counting method included all eligible associations.

## Introduction

 Cardiometabolic diseases (CMDs), including type 2 diabetes mellitus and cardiovascular diseases (CVDs), are the leading cause of death worldwide.^1^ Despite a reduction in the burden of CMD in high-income countries over the last decades, the global burden has increased.^2 3^ In 2017, the global disability-adjusted life-years attributed to diabetes and CVD were 68 million^4^ and 366 million,^3^ respectively. Given the magnitude of CMD burden and the disproportionate burden in low-income and middle-income countries, it is of utmost importance to identify risk factors to prevent CMD. Proximal risk factors for CMD include unhealthy lifestyle behaviours, for instance, unhealthy dietary intake,^5 6^ sedentary behaviour,^7 8^ alcohol consumption,^9^ smoking^10 11^ and poor sleeping habits.^12^ They influence CMD outcomes through biological responses such as obesity, high low-density lipoprotein and high blood pressure levels.^13 14^ Unhealthy lifestyle behaviours are in turn influenced by more distal risk factors at the environmental level. These distal risk factors contribute to a substantial part of the disease burden, nevertheless, a number of them have not been identified yet.^15^ Among the environmental-level risk factors is the social environment, which is generally understood as the social relationships and social context in which groups of people live and interact. The different dimensions of the social environment operate in various spheres of influence, such as within a network of friends and in the neighbourhood context.^16^

**Figure 1:**
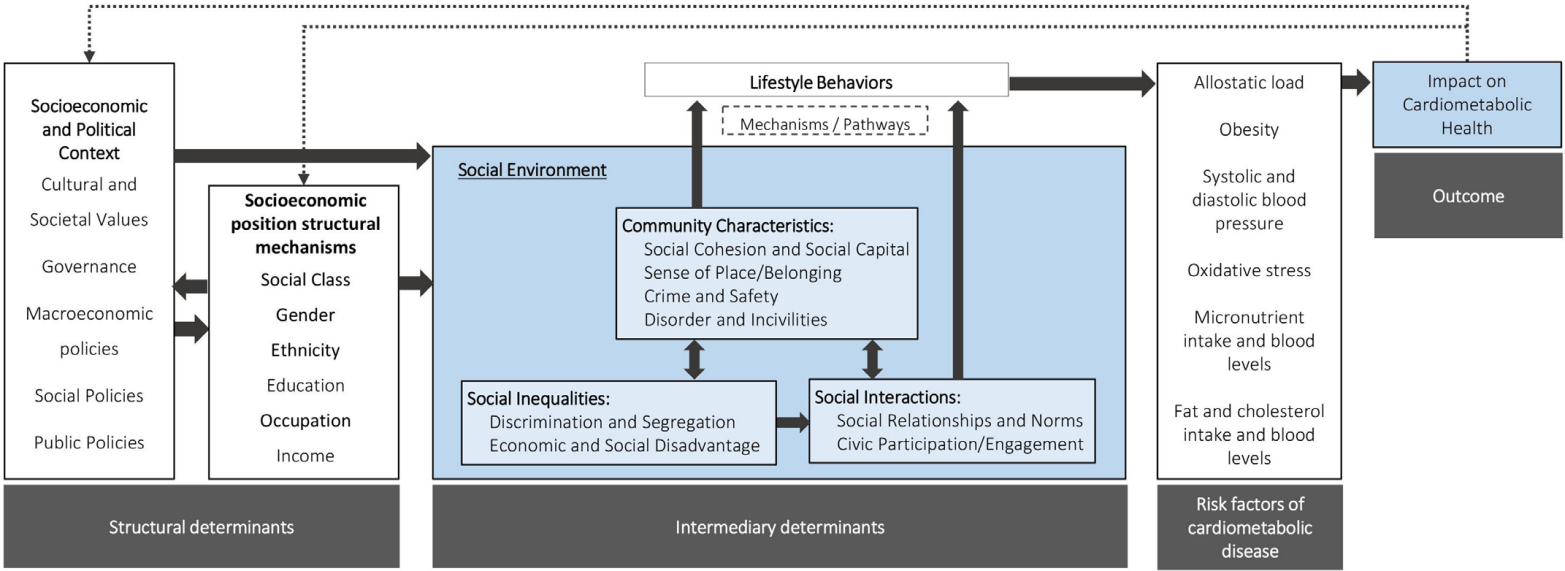
Conceptual framework on social environment and cardiometabolic health.

We use figure 1 to display the hypothesised pathways through which dimensions of the social environment affect CMD. This figure is adapted from the WHO framework on the social determinants of health^17^ and builds on the work of Kepper *et al*.^18^ The framework describes how structural determinants such as the socioeconomic and political context shape the social environment, and how social environmental factors enhance or constrain lifestyle behaviours, which may, in turn, generate biological responses that will increase or decrease CMD health. As is true for all frameworks, this is a simplified reflection of the undoubtedly complex reality. Previous work has highlighted the use of inconsistent terminology and conceptualisation of the dimensions of the social environment, with definitions and measurements unable to differentiate between various social phenomena or capture their magnitude.^1618^,^21^

There is ample evidence for an association of dimensions of the social environment with lifestyle factors. For example, a meta-analysis found that higher social support was associated with better sleep quality,^22^ individuals with stronger social networks had higher fruit and vegetable consumption,^23^ and stronger social ties buffered against stress.^24^ In addition, higher levels of neighbourhood crime have been linked to higher smoking rates,^25^ higher socioeconomic status to more physical activity^26 27^ and higher social capital to lower alcohol consumption.^28^ The literature base seems to suggest that these associations trickle through to CMD. Indeed, a number of studies have focused on social support,^29 30^ social cohesion,^31 32^ perception of safety, violent crime, and signs of incivility,^33^,^35^ and residential segregation^36 37^ as risk factors for CMD. Moreover, evidence on the relationship between social capital and CMD has grown in the last two decades^38^,^41^ and the evidence on the impact of area-level deprivation on CMD risk is substantive.^42^,^48^ However, no systematic overview exists of the extent to which dimensions of the social environment are associated with CMD risk. Therefore, we aimed to perform a systematic review and meta-analysis that summarises the knowledge on the link between dimensions of the social environment and CMD incidence or prevalence.

## Methods

### Data sources and searches

The systematic review followed the Preferred Reporting Items for Systematic Reviews and Meta-Analyses (PRISMA) statement (www.prisma-statement.org).^49 50^ The research protocol was prospectively registered with the International Prospective Register of Systematic Reviews (PROSPERO) database (ID: CRD42021223035). Post hoc amendments to the eligibility criteria were added to the protocol and registered with the PROSPERO database (ie, clarifications and a correction related to the exclusion of ecological studies) before data extraction began. We systematically searched the bibliographic databases PubMed, Scopus and the Web of Science Core Collection from inception to 29 February 2024 (TCA and LS). The search strategy—developed together with the library information specialist (LS)—comprised three blocks: (1) “cardiometabolic diseases” (eg, myocardial infarction, hyperlipidaemia, glycated haemoglobin (HbA1c)); (2) “dimensions of the social environment” (eg, social cohesion, area-level deprivation) and (3) “contextual level” of the social environment (eg, neighbourhood, network). We used free-text terms in all databases. For PubMed, the search terms also included indexed terms from MeSH. Duplicate articles were removed using EndNote. We manually searched the reference lists of the articles included for other relevant publications when they included at least one term from the contextual-level search block. The full search strategy for all databases can be found in online supplemental file 1. Due to the large number of papers included, we divided the results over two twin reviews according to whether the outcome under investigation was a cardiometabolic ‘hard outcome’ or ‘risk factor’ for CMD (eg, HbA1c, blood lipids and metabolic syndrome), the latter—based on a search run in February 2021—was published in Social Science & Medicine—Population Health.^51^

### Study selection

We reviewed original studies that examined associations between dimensions of the social environment and CMD incidence or prevalence in adults. The scope of this review was limited to exposures that assessed properties of the social context, that is, social factors assessed at the environmental level (eg, area-level income), but not those that assessed properties of individuals (eg, individual-reported pleasure derived from social network). We did, however, include social factors that were assessed at the individual level but reflect a property of the social context in which an individual is inserted (eg, one’s social network size). We defined CMD as type 2 diabetes mellitus, hypertensive diseases, stroke, ischaemic heart disease, heart failure, diseases of arteries, arterioles and capillaries, and unspecified CVD.

**Figure 2:**
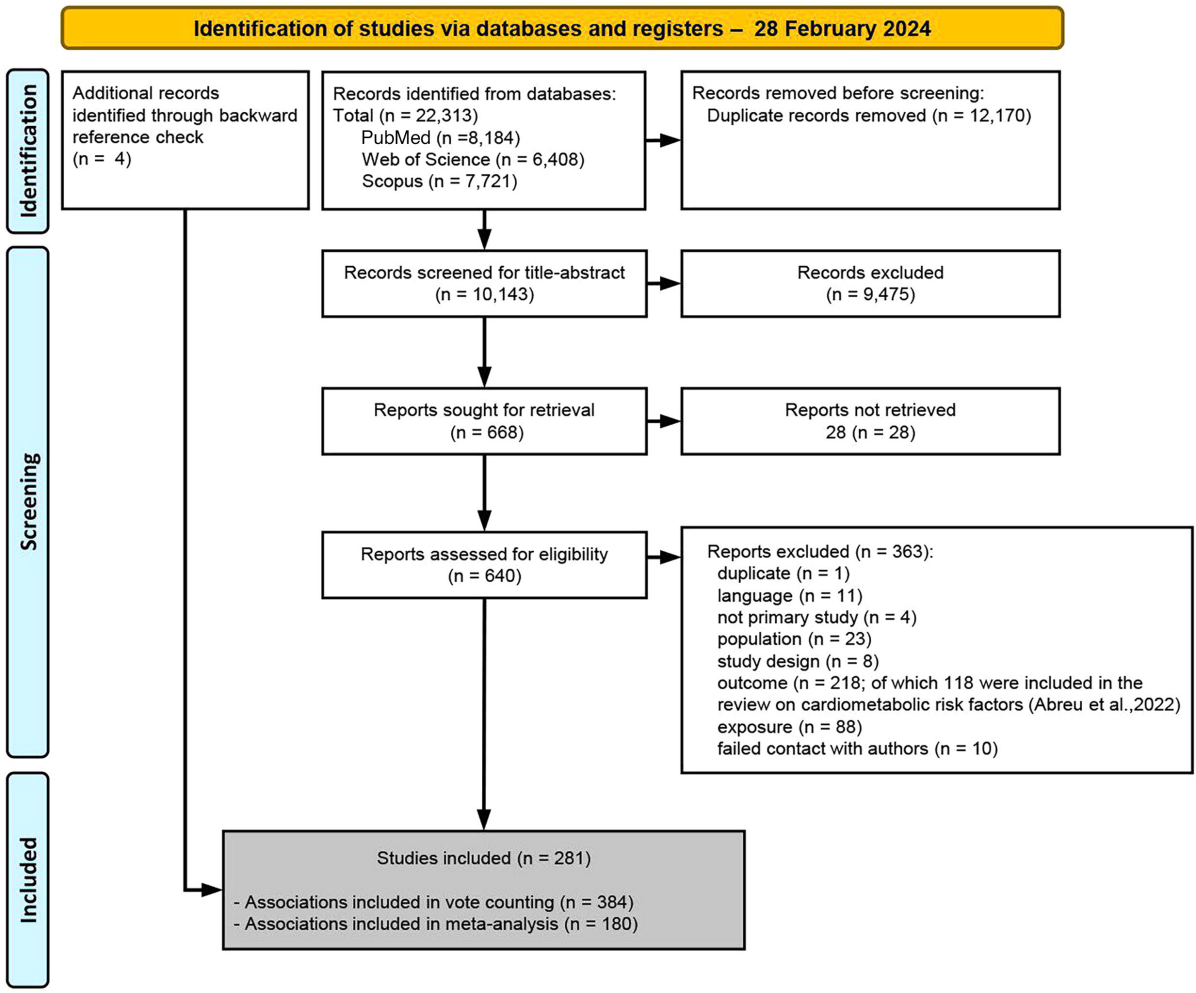
PRISMA flow diagram of study inclusion. PRISMA, Preferred Reporting Items for Systematic Reviews and Meta-Analyses.

All titles and abstracts of each study were screened independently by two authors (TCA and JDM or JB). We piloted the screening process in a random subsample of 100 studies. We used the mobile apps Covidence and Rayyan to manage and organise the screening process.^52^ Following title-abstract screening, full-text articles were assessed for eligibility by two authors independently (TCA and JDM or JB). Cohen’s kappa coefficients were computed for title and abstract and full-text screening to indicate the agreement between pairs of reviewers. A third author (JDM or JB) resolved any inconsistencies in study eligibility. See PRISMA flow diagram of study inclusion in figure 2.

We included studies if they (1) studied an adult population or followed children and adolescents beyond 18 years of age; (2) studied incidence or prevalence of CMD outcomes; (3) covered any measure of the social environment that potentially influences the risk of CMD; (4) were (quantitative or qualitative) observational or intervention studies and (5) were written in English. We excluded studies if they (1) were limited to children and adolescents; (2) studied obesity as an outcome—given the recent evidence available for this outcome^53^,^55^; (3) studied CMD with little or no influence of lifestyle behaviours (eg, congenital heart disease, rheumatic heart disease and type 1 diabetes) as outcomes; (4) focused on treatment, medication or management of disease outcomes; (5) were conducted in samples of patient populations or pregnant women; (6) were health economic evaluations, simulation studies or publications that did not report original scientific research or (7) studied mortality outcomes alone or did not differentiate between morbidity and mortality outcomes. Considering that the vast majority of diabetes cases in the adult general population are of type 2, we assumed that studies that did not explicitly specify the type of diabetes have included type 2 diabetes mellitus cases and were, therefore, considered eligible. However, if studies knowingly did not differentiate between type 1 and type 2 diabetes cases, they were excluded.

### Data extraction

Two authors (TCA or FH) performed data extraction from eligible studies, according to a standardised protocol and a predefined list of variables, which included study and sample characteristics as well as information on exposure, outcome and effect measures. A second author (JDM) qualitatively appraised the data extraction of a random subsample of 10% of the studies included; any inconsistencies were discussed and agreement was reached. We categorised social environment factors into one of the eight dimensions depicted in figure 1, that is, economic and social disadvantage includes, for instance, area-level poverty and deprivation, social relationships and norms include support from the social network, crime and safety include crimes per capita, social cohesion and social capital include neighbourhood collective efficacy and social trust, disorders and incivilities include neighbourhood disorder, civic participation and engagement include participation in social activities, discrimination and segregation include percentage of migrants in an area and sense of place/belonging includes place attachment and sense of community. Exposures were classified into one of the eight dimensions following the terminology employed by the included studies. When an exposure variable could not be clearly assigned to a category, we classified it based on the variable measurement and operationalisation. We categorised outcomes into one of seven categories namely type 2 diabetes mellitus, hypertensive diseases, stroke, ischaemic heart disease, heart failure, diseases of arteries, arterioles and capillaries, and unspecified CVD, following the International Classification of Diseases definitions.^56^ In case of missing data on effect measures, we contacted study investigators. There is clear evidence for sex differences in CMD risk,^57^ as well as in social relations.^58^ We, therefore, extracted data on sex-specific effect metrics when available. We extracted data from the model in which effect measures were adjusted to the highest degree (ie, fully adjusted models), except in cases where adjustments were made for potential confounders (eg, lifestyle behaviours).

### Quality assessment

Two authors (TCA or FH) assessed quality/risk of bias of all the studies included and a second author (JDM) qualitatively appraised the data extraction of a random subsample of 10% of the studies included; any inconsistencies were discussed and agreement was reached. The criteria and ratings for each domain varied depending on the study design. For observational studies, we used the Newcastle-Ottawa Scale (NOS) to assess cohort and case–control studies^59^ while to assess cross-sectional studies we used an adapted version of the NOS tool.^60^ We drew an overall quality score for each study based on seven or eight items divided into three domains namely selection, comparability and exposure (for case–control) or outcome (for cohorts and cross-sectional studies). For intervention studies, we used the Risk of Bias in Non-randomised Studies—of Interventions (ROBINS-I) tool and the Risk of Bias 2 (RoB 2) tool for randomised studies.^61 62^ ROBINS-I assesses the quality of the studies based on seven domains (namely bias due to confounding and selection of participants, bias in classification of interventions, bias due to deviations from intended interventions, missing data, measurement of outcomes and selection of the reported result) while RoB 2 is based on five domains (namely randomisation process, deviations from intended interventions, missing outcome data, measurement of the outcome and selection of the reported result).

Ratings reflect the risk of bias in the association between the relevant social environmental factor and CMD, even if this was not the primary research question of a study. Since no threshold for overall study quality is established for NOS, we rated a study as ‘low quality’ when it received less than 50% of all possible points and was, therefore, excluded from the vote count and meta-analyses. Prior to the quality assessment of included studies, we piloted the process in a random subsample of 10 studies. We generated a summary of quality assessment scores per tool per domain across all studies included.

### Data synthesis

We synthesised data extracted with two approaches namely vote counting for an overview of the direction of evidence,^63^ and meta-analysis for a statistical summary of the effect estimates. In both methods, we did not include associations from low-quality studies and associations from the dimension discrimination and segregation (eg, proportion of ethnic minority in a neighbourhood). The latter was excluded because its exposure measures were highly heterogeneous in operationalisation and poorly defined, and thus the associations were not comparable for vote counting and meta-analysis purposes. Moreover, we did not include effect measures from case–control (n=5) and intervention studies (n=6) given the low number of associations reported and limited comparability with observational studies. We classified exposure categories as indicating ‘better’ and ‘worse’ social environment. For instance, ‘worse’ social environment for the dimension economic and social disadvantage was the group experiencing the most deprivation and ‘better’ social environment for the dimension social relationships and norms was the category with the most social support.

#### Vote counting

In accordance with Cochrane’s handbook for systematic reviews of interventions,^63^ we synthesised the overall effect measures of included studies using a vote counting strategy whereby studies were classified according to the direction (but not statistical significance) of effect estimates. We did not include associations in vote counting when (1) they were sex-specific, rather than overall associations; (2) a direction of effect could not be established or (3) the effect estimate and reference category were not comparable (eg, reference category was the ‘middle’ category). We presented the results of vote counting in a direction-of-effect plot, for which we categorised effect estimates into one of two directions: (1) worse social environment is associated with an increased risk of CMD or (2) better social environment is associated with an increased risk of CMD. When the direction of effect was not directly reported by the study investigators, we defined it based on the data extracted.

#### Meta-analysis

We performed meta-analyses when three or more associations were available per combination of social environment dimension and CMD outcome. We did not include associations in meta-analysis when (1) there were less than three associations for a given exposure-and-outcome combination; (2) the data reported were not suitable for meta-analysis (eg, missing variance measure); (3) the effect estimates were not reported as ratios (eg, ORs, relative risk and HRs); (4) the exposure measures were not operationalised as dichotomous or categorical variables (eg, continuous exposure) and (5) reference category did not allow for comparison (eg, reference category was the ‘middle’ category). In the case of categorical exposure, we pooled the ratios comparing the two extreme categories of the exposure gradient (eg, highest quantile of area-level income vs lowest quantile of area-level income) in association with a CMD outcome. The exposures reference categories were harmonised and defined as the ‘best-off’ category. We performed random-effects meta-analysis which accounted for the multilevel structure of the data (ie, a single study can report more than one relevant association), based on a t-distribution. We performed subgroup analysis for sex-specific effect measures when data were available. We performed sensitivity analysis (1) according to the studies’ country income level,^64^ including only studies performed in LMIC, (2) including only longitudinal studies, (3) including studies of low quality only for the two most commonly examined combinations of exposure–outcome (ie, economic and social disadvantage and hypertensive diseases and economic and social disadvantage and type 2 diabetes mellitus) and (4) for the effect of meta-analysing only OR effect estimates instead of all ratios combined. We generated forest plots for each meta-analysis performed and assessed heterogeneity with I^2^ statistics accounting for the dependency among associations originating from the same study. Results are expressed as ORs and 95% CIs. Analyses were performed in R V.4.3.2,^65^ using the functions rma.mv, forest and funnel of the Metafor package.^66^

#### Funnel plots

Funnel plots were generated to qualitatively assess publication bias. We examined funnel plots for the exposure-and-outcome combinations of more than five associations and followed the same criteria applied to inclusion of associations in the meta-analysis.

### Patient and public involvement

None.

## Results

From 10 143 records screened for title and abstract, 640 full articles were screened (online supplemental file 2) and of these, 281 were included in this review. 59% (n=165) of the included studies applied a cross-sectional design, 37% (n=105) a longitudinal design, 2% (n=6) were (quasi-)experimental and 2% (n=5) were case–control studies. 73% (n=204) of the studies were published in the past 10 years, and all studies were conducted in high-income or middle-income countries (88%, n=246 and 12%, n=35, respectively) (online supplemental table 1). No qualitative studies were included. 23 studies were conducted exclusively in women (n=19) or men (n=4). The Cohen’s kappa value for title and abstract screening ranged between 0.52 and 0.58 (moderate agreement; depending on the combination of authors involved) and for full-text screening was 0.66 (substantial agreement).^67^ Among the studies included, 1108 relevant associations were reported, of which 66% (n=728) were overall associations and 34% (n=380) were sex-specific associations (online supplemental table 2). From the overall associations, 53% (n=384) were eligible for vote counting and 27% (n=196) for meta-analysis.

Of all social environment dimensions, the largest number of associations was related to the dimension economic and social disadvantage (61% of all associations), followed by social relationships and norms (19%) and discrimination and segregation (9%). Less evidence was found for the remaining dimensions (ie, social cohesion and social capital, crime and safety, civic participation and engagement and disorder and incivilities) in relation to the outcomes investigated and no evidence was found on the dimension sense of place/belonging (online supplemental table 2). Among the CMD outcomes considered, type 2 diabetes mellitus and hypertensive diseases were the only outcomes investigated in association with all dimensions of the social environment (34% and 25% of all associations included, respectively). Other commonly observed outcomes were ischaemic heart disease (17%) and stroke (11%), which were both investigated in association with all dimensions except disorder and incivilities. The remaining CMD outcomes (ie, unspecified CVD, heart failure and diseases of arteries, arterioles and capillaries) were less commonly investigated and explored in relation to fewer social environment dimensions (online supplemental table 2).

### Quality assessment

**Figure 3:**
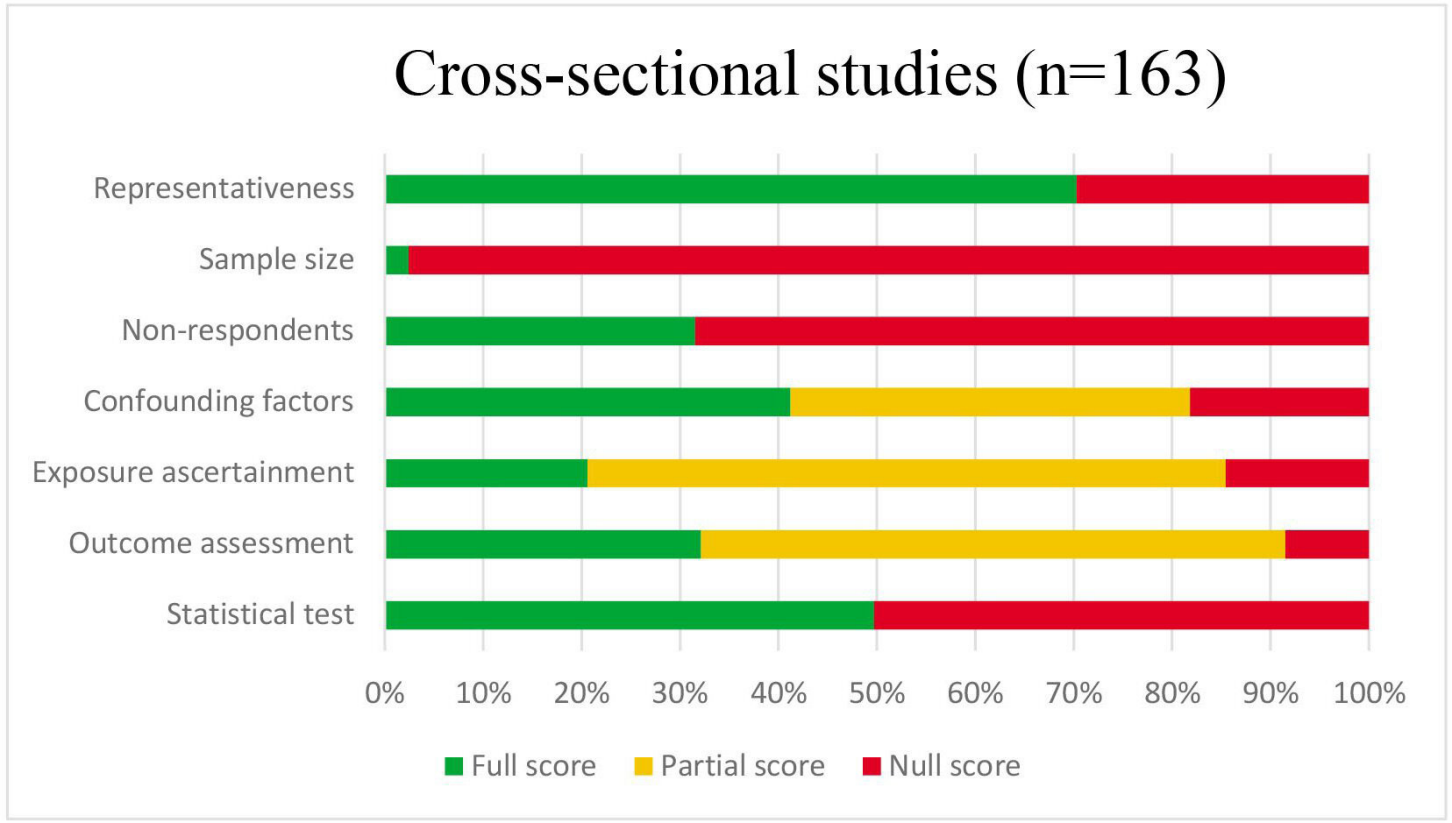
Summary of quality assessment across studies using New-Ottawa Castle tool Quality Assessment Scale adapted for cross-sectional studies

Of all included studies, 23% (n=66) were considered of low quality (onlinesupplemental tables 3A3E). Of these, 58 were cross-sectional studies and 5 were longitudinal studies. Overall, cohort studies (n=102; 79% of points scored) were of higher quality than cross-sectional studies (n=107; 60% of points scored) (figures3  4), which often did not report sample calculations and did not use validated tools to assess the social environmental factors and thus performed poorly on the ‘sample size’ and ‘exposure ascertainment’ items, respectively. Moreover, several cross-sectional studies insufficiently adjusted for confounders and the statistical analysis applied did not account for the multilevel structure of the data used. Of the four case–control studies, one was assessed as low quality. Randomised studies (n=2) were of low quality and quasi-randomised studies (n=4) were of low/moderate quality.

**Figure 4:**
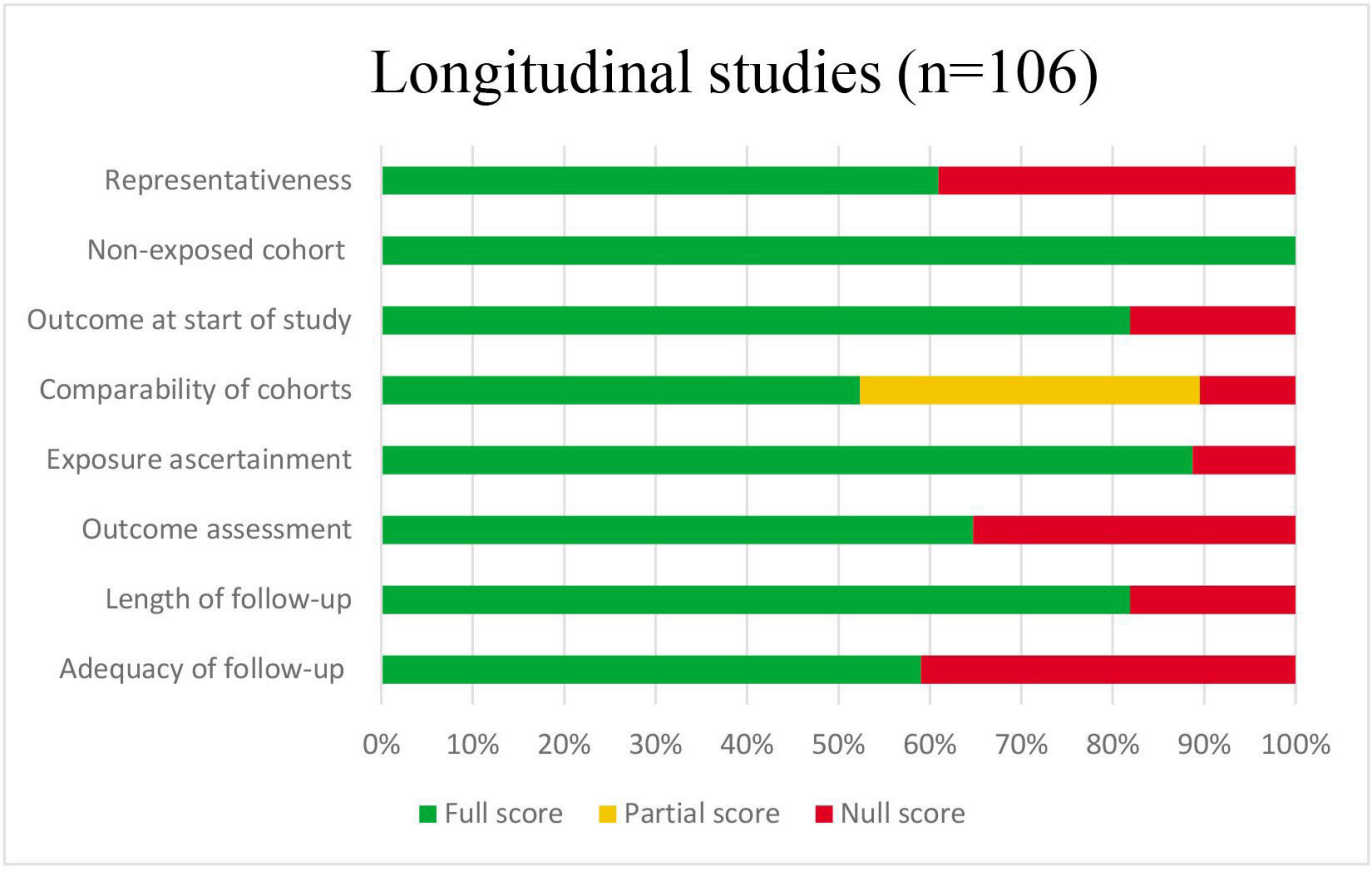
Summary of quality assessment across studies using New-Ottawa Castle tool Quality Assessment Scale for cohort studies

### Vote counting

**Figure 5:**
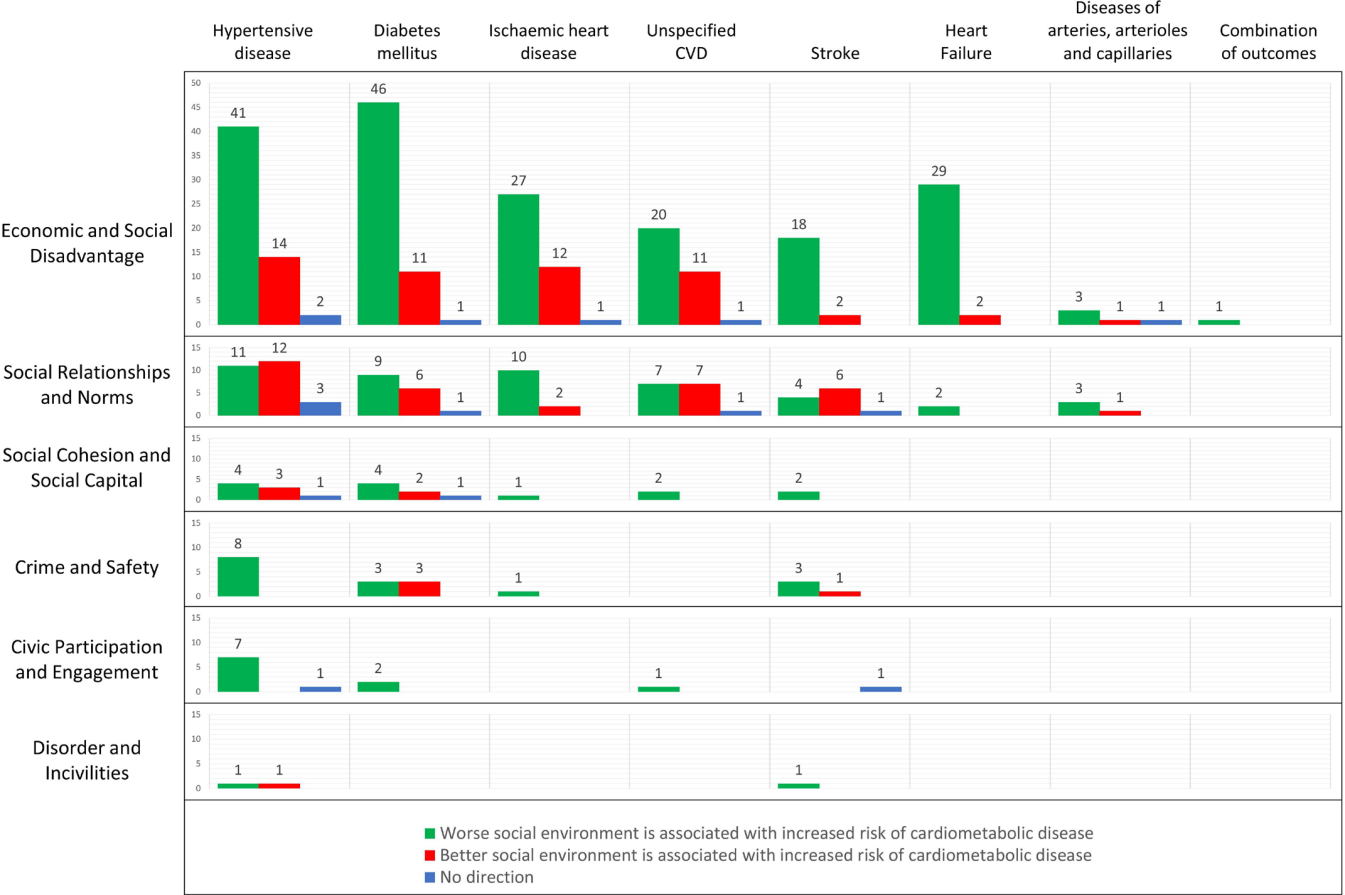
Vote count based on direction of effect for associations between social environment dimensions and cardiometabolic disease outcomes. CVD, cardiovascular disease.

Vote counting was possible for 30 exposure-and-outcome combinations (figure 5). All social environment dimensions were found in association with at least one outcome, except for the dimension sense of place/belonging and the dimension discrimination and segregation, which was not included in the vote counting. In 21 of the 30 combinations, the majority of the effect estimates indicated an increase in the risk of CMD with worse social environment. More specifically, this direction notably prevailed among the associations between economic and social disadvantage and all CMD outcomes. In contrast, the direction of effects was mostly inconsistent for the dimension social relationships and norms. In 11 of the 384 associations, the effect estimate was not indicating any direction (eg, OR=1.00).

### Meta-analysis

**Figure 6:**
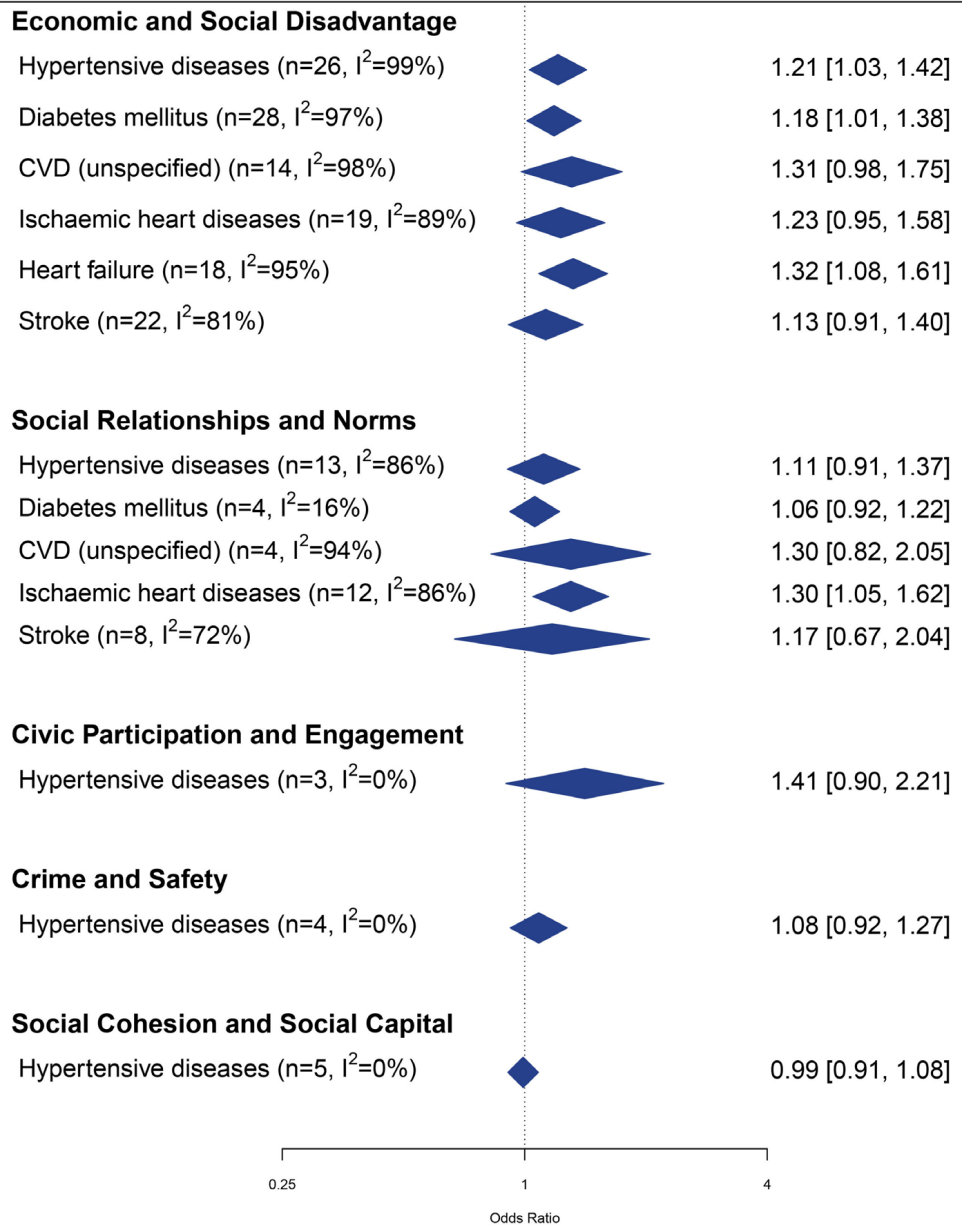
Summary of forest plots (random-effects model) for the meta-analyses of social environment dimensions and cardiometabolic health outcomes. CVD, cardiovascular disease.

Meta-analyses were possible for 14 exposure-and-outcome combinations (onlinesupplemental figure 1A1N; figure 6). All social environment dimensions were meta-analysed in relation to at least one outcome, except for the dimensions disorder and incivilities and sense of place/belonging, which did not have sufficient eligible associations, and the dimension discrimination and segregation, which was a priori excluded in the meta-analysis. The largest number of meta-analyses was possible for the dimension economic and social disadvantage (n=6). Regarding the outcomes, only diseases of arteries, arterioles and capillaries did not provide sufficient eligible data for meta-analysis.

We observed an overall trend suggesting that worse social environment was associated with an increased risk of CMD outcomes in all but one meta-analysis (ie, social cohesion and social capital and hypertensive diseases). Despite this tendency, only 4 out of 14 meta-analyses were statistically significant. Three of these four were related to economic and social disadvantage dimension: more economic and social disadvantage was associated with a higher risk/prevalence of hypertensive diseases (OR 1.21, 95% CI 1.03 to 1.42; n=26; I^2^=99%), type 2 diabetes mellitus (OR 1.18, 95% CI 1.01 to 1.38; n=28; I^2^=97%) and heart failure (OR 1.58, 95%CI 1.08 to 1.61; n=18; I^2^=95%). Also, worse social relationships and norms were associated with a higher risk/prevalence of ischaemic heart disease (OR 1.30, 95% CI 1.05 to 1.62; n=12; I^2^=86%). Overall, the magnitude of the pooled effects ranged from OR 0.99 (95% CI 0.91 to 1.08) for social cohesion and social capital and hypertensive diseases to OR 1.58 (95% CI 1.08 to 1.61) for economic and social disadvantage and heart failure. Generally, there was high heterogeneity in the meta-analyses, with 9 out of 14 meta-analyses having I^2^ values >75%. Insufficient evidence was available to conduct meta-analysis for the remaining exposure-and-outcome combinations.

It was possible to perform seven sex-specific meta-analyses for both men and women (table 1). 12 out of 14 sex-specific pooled effects showed the same direction of effect as the overall meta-analyses, that is, worse social environment was associated with increased risk/prevalence of CMD outcomes while 2 meta-analyses for men showed OR smaller than 1 (opposite direction as main meta-analysis). Estimates were more often statistically significant for men than for women, with two out of seven meta-analyses for men showing statistically significant results. Among men, more economic and social disadvantage was associated with a higher risk/prevalence of type 2 diabetes mellitus (OR 1.25, 95% CI 1.07 to 1.45; n=14; I^2^=94%) and ischaemic heart disease (OR 1.19, 95% CI 1.07 to 1.33; n=23; I^2^=80%) and worse social relationships and norms were associated with a higher risk/prevalence of type 2 diabetes mellitus (OR 1.32, 95% CI 1.08 to 1.60; n=6; I^2^=25%). The magnitude of the pooled effects was somewhat different for men and women, with ORs differing more than 10% between women and men in four out of six comparable meta-analyses. Heterogeneity seemed to be lower in meta-analysis performed among men, with three out of seven meta-analyses having I^2^ values >75% while among women six out of seven meta-analyses had I^2^ values >75%.

**Table 1:** Summary sex-specific pooled effects and between-study variance estimates and 95% CIs from meta-analysis models

Exposure	Outcome	n	OR	95% CI		Total I^2^
Women						
Economic and social disadvantage	Hypertensive diseases	11	1.03	0.86 to 1.23		91%
Economic and social disadvantage	Diabetes mellitus	16	1.31	0.94 to 1.82		97%
Economic and social disadvantage	Ischaemic heart diseases	23	1.24	0.91 to 1.69		95%
Social relationships and norms	Hypertensive diseases	5	1.17	0.71 to 1.93		81%
Social relationships and norms	Diabetes mellitus	9	1.14	0.94 to 1.38		96%
Social relationships and norms	Heart failure	3	1.26	0.53 to 2.99		85%
Crime and safety	Hypertensive diseases	3	1.12	0.63 to 1.98		6%
Men						
Economic and social disadvantage	Hypertensive diseases	3	1.18	0.71 to 1.95		66%
Economic and social disadvantage	Diabetes mellitus	14	1.25	1.07 to 1.45	**	94%
Economic and social disadvantage	Ischaemic heart diseases	23	1.19	1.07 to 1.33	**	80%
Social relationships and norms	Hypertensive diseases	5	1.38	0.61 to 3.10		94%
Social relationships and norms	Diabetes mellitus	6	1.32	1.08 to 1.60	*	25%
Social relationships and norms	Ischaemic heart diseases	3	0.96	0.56 to 1.63		1%
Crime and safety	Hypertensive diseases	3	0.98	0.38 to 2.50		69%

* p≤0.05; **p≤0.01.

For sensitivity analysis of studies performed in middle-income countries (online supplemental table 4), only three meta-analyses were possible, including at most seven associations each. The pooled effect for economic and social disadvantage and type 2 diabetes mellitus among middle-income countries (OR 0.77, 95% CI 0.53 to 1.13; n=4; I^2^=99.9%) was found to be in the opposite direction of that observed for all countries combined (OR 1.18, 95% CI 1.01 to 1.38; n=28; I^2^=97%). The other two meta-analyses among middle-income countries had similar magnitude compared with all countries combined; however, the 95% CIs were wider among middle-income countries.

For sensitivity analysis of longitudinal studies (n=11 meta-analyses; online supplemental table 5), no major differences in pooled effects in comparison with the meta-analysis including both longitudinal and observational studies were observed. Three out of four pooled effects that were statistically significant in the main meta-analysis remained statistically significant.

Sensitivity analysis, including studies of low quality compared with the main analysis, where these were excluded, was conducted for the two most commonly examined combinations of exposure–outcome. These analyses showed no major differences compared with the main meta-analysis, despite the increase in the number of associations included (ie, economic and social disadvantage and type 2 diabetes mellitus: OR 1.19, 95% CI 1.05 to 1.36; n=40; I^2^=95% and economic and social disadvantage and hypertensive diseases: OR 1.18, 95% CI 1.03 to 1.34; n=35; I^2^=99%).

Sensitivity analysis with OR effect estimates only (n=9 meta-analyses) revealed no major differences in pooled effects in comparison with the respective meta-analysis generated with all ratios combined (online supplemental table 6). Three out of nine meta-analyses performed differed more than 10% from the respective meta-analysis with all ratios combined but maintained the same direction of effect (eg, economic and social disadvantage and heart failure: OR (main analysis): 1.58, 95% CI 1.08 to 1.61 vs OR (only OR): 1.15, 95% CI 0.69 to 1.92). Generally, there was also high heterogeneity among meta-analyses performed with OR effect estimates only, with seven out of nine meta-analyses having I^2^ values >75%.

### Funnel plots

We found substantial asymmetry in the funnel plots (n=9), particularly for some exposure-and-outcome combinations, for instance, economic and social disadvantage and hypertensive diseases (onlinesupplemental figure 2A2I). This may suggest publication bias but may also reflect study heterogeneity.^68^

## Discussion

This systematic review and meta-analysis provides evidence that being exposed to a worse social environment is consistently associated with an increased risk of CMD, with worse economic and social disadvantage being significantly associated with a higher risk of hypertensive diseases, type 2 diabetes mellitus, and heart failure, and worse social relationships and norms with a higher risk of ischaemic heart disease. Both the meta-analyses and vote counting pointed in the same direction. Our findings also illustrate that even though effect estimates were approximately similar for men and women, they were less heterogeneous among men. We included 281 studies reporting on 1108 associations that explored the relationship between dimensions of the social environment and CMD risk in adults worldwide. To the best of our knowledge, this is the most comprehensive work to date on the aforementioned associations.

Most evidence was available for the social environment dimension economic and social disadvantage, followed by social relationships and norms, and the outcome type 2 diabetes mellitus, followed by hypertensive diseases and ischaemic heart disease. Other reviews also concluded that economic and social disadvantage was the most commonly studied social environmental dimension when investigated in relation to physical activity and obesity.^18^ Less evidence was available for some other dimensions such as disorder and incivilities and sense of place/belonging. This could be due to the fact that research on dimensions other than economic and social disadvantage in relation to CMD risk is only recently emerging, as confirmed by the majority of studies published in the past 10 years.

In relation to the magnitude of the effect estimates generated by the meta-analysis, the effect sizes ranged from OR=0.99 for social cohesion and social capital in relation to hypertensive diseases, to OR=1.58 for economic and social disadvantage and heart failure. The difference in effect sizes cannot be explained by the extent to which social environment dimensions could be considered as more or less distal. A consistent pattern for strong associations with a specific outcome was not visible as well. One could argue that living in a deprived neighbourhood or a lack of social relationships leads to stress, rising blood pressure and hypertensive diseases ‘directly’—resulting in larger effect sizes—whereas the level of civic participation may lead to stress via reduced social cohesion only—resulting in smaller effect sizes. However, we found no indication for this explanation.

There are few meta-analyses on social environmental factors and CMD risk in the literature. The few meta-analyses available are not entirely comparable to the methodology we adopted and used different operationalisation of social environment factors and outcomes.^69^,^71^ However, the magnitude of the pooled effects observed by them is similar to what we found. For instance, a meta-analysis focusing on area-level socioeconomic indexes and incident heart failure found associations to be significant and in line with our findings.^69^ Moreover, a meta-analysis of longitudinal studies found a statistically significant association between poor ‘social relationships’ and coronary heart disease and stroke.^70^ While observing the same direction of effect we found, a meta-analysis pooling results from studies performed in Australia and New Zealand found no statistically significant relationship for the exposure ‘social health’ (ie, social isolation, lack of social support and loneliness) in relation to coronary heart disease and stroke combined.^71^ These findings correspond to our observation that the economic and social disadvantage dimension showed on average stronger pooled effects than other dimensions.

Results were overall consistent across meta-analyses and vote counting but not for all dimensions of the social environment. Differences in consistency across the two synthesis methods may have arisen from the fact that the vote counting approach considered all eligible associations, including the ones with continuous exposures while the meta-analysis only considered extreme categories of exposure. The meta-analysis of continuous exposures was not performed due to the complexity of converting continuous data into categorical data and harmonising different effect estimates (eg, beta and OR), and thus only associations comparing CMD risk between extreme categories of exposure were included. The inconsistency between the two methods was most pronounced for the dimension social relationships and norms. It seems that the adverse effects of having no friends versus having at least one friend on CMD—as observed in the meta-analyses—reflect no linear, dose–response relation. Indeed, when measures from this dimension are treated in a continuous, linear fashion (eg, 1 unit increase in the number of friends), its relationship with CMD risk becomes inconsistent. This may suggest that some social environment factors could be associated with CMD risk in a curvilinear relationship or potentially have a threshold effect (eg, no additional benefit to CMD health after a certain exposure level), as opposed to a linear relationship (eg, increase on continuous exposure measure is associated with a graded linear change in CMD risk). Such non-linear relationships have been hypothesised, for instance, in the relationship between a number of social contacts and certain health outcomes^72 73^; however, this non-linearity has not been extensively explored in relation to CVD^74^ and type 2 diabetes mellitus. Therefore, further exploring the relationship between certain social environmental factors and CMD risk with adequate statistical approaches and exposure operationalisation is needed.

The majority of studies included were cross-sectional observational studies, which hamper the application of a ‘life course’^75^ or ‘exposome’^76^ perspective which assumes that the biological responses to adverse social environments would mainly arise when individuals are exposed to them for a longer duration of time. Indeed, included longitudinal studies were generally of better quality than the cross-sectional studies; however, for the exposure-and-outcome combinations where sensitivity analysis could be conducted exclusively using longitudinal studies, we did not observe marked differences. Considering this lack of evidence on ‘cumulative exposure’ to adverse social environments coupled with the fact that the studies included mostly focused on the impact of single social environment factors on the risk of single CMD outcomes, our results are likely a lower bound conservative estimate of the influence of the social environment on CMD risk. Given the existing evidence on links between the social environment and other health outcomes such as frailty,^77^ mental health^78^ and general health,^79^ the importance of the social environment goes beyond its effects on cardiometabolic health. There is thus a need for further research and design and implementation of effective policies and interventions in this arena.

Another weakness of the ascertained evidence is the heterogeneity in definitions and operationalisation of exposure measures, contributing to high levels of heterogeneity in some of our findings. Similarly, previous reviews observed the use of a multitude of measures to assess the social environment.^18 26^ For instance, a scoping review on measures of social environment for walkability mapped 20 different surveys, which employed 182 unique items.^80^ We dealt with the aforementioned heterogeneity by following the call for more consistency in the social environment literature,^18^ and thus combining all social environment factors that were categorised into the same dimension for the purposes of synthesis of findings. Such an approach hampers the interpretability of the pooled effect estimates but it allowed us to explore the combined effect of a given dimension on CMD risk. While a discussion regarding the definition and measurement of specific social environment constructs is beyond the scope of this review, others provide a comprehensive overview of the considerations therein.^81^

The issue with heterogeneity was particularly problematic for the discrimination and segregation dimension, where the variety of exposure measures adopted did not allow for the synthesis of the findings across different studies. For example, exposure ascertainment varied from simplistic approaches (eg, percentage of a minority group in an area) to more sophisticated methods (eg, spatial autocorrelation measures that take into account the racial/ethnic composition of surrounding areas). Moreover, studies often took into account two groups (eg, blacks vs whites) while geographical areas often have a more diverse composition. We also would argue that ‘discrimination and segregation’ measures are highly context-dependent,^82 83^ as a 1% increase in the population belonging to an ethnic minority group has a very different meaning for areas with a 2% ethnic minority population vs a 50% ethnic minority population.

Moreover, subgroup analyses by sex suggest that the heterogeneity found in the overall analyses might be partly due to sex differences and that the social environment may affect men and women differently, indicating that sex differences should be further investigated.

The main strength of this review is its large and comprehensive scope and broad systematic search of the literature on three bibliographic databases in addition to backward reference checks and searches without time restriction. This robust search strategy allowed us to capture as much relevant evidence as possible, and thus dilute the impact of the heterogeneity on social environmental factors in retrieval and synthesises of findings. Nevertheless, this review has some limitations. We excluded studies assessing mortality outcomes based on the fact that the social environment can play different roles across a disease course (eg, from prevention to mortality), and our scope was limited to CMD morbidity. However, this criterion also excluded studies combining morbidity and mortality outcomes. Also, the quality assessment tools used here have known limitations.^84^ We reduced the impact of these by piloting the application of the tools and by adapting specific guidelines and decision rules for each item.

### Implications

Future research should invest in standardising the definitions of social environment dimensions and harmonise the operationalisation of social environmental factors, using validated tools which allow for comparability of findings from different contexts and times. Besides a few of the included studies having adopted validated indexes (eg, the Australian Socio-Economic Indexes for Areas^85^ and the Berkman-Syme Social Network Index^86^), the use of validated tools to assess social environmental factors was rare. We highlight that the conceptualisation of such tools would ideally consider the fact that an individual’s perception of the social environment can have implicit or unconscious effects,^87^ and individuals may not be able to recall them in self-reported measures based on explicit perception. Second, the use of well-designed longitudinal studies, natural experiments and intervention studies, where possible, could help make causal inferences on the association between social environment and CMD risk. Such studies must consider, for instance, the various pathways and mechanisms that lead to CMD onset, with the potential for risk accumulation over the life course. Third, the role of the online social environment has not been extensively investigated in relation to CMD. In this regard, not only individual’s exposure to social environment factors such as social interactions in social networking platforms is of relevance, but also individuals’ exposure to paid marketing of unhealthy products while interacting with peers.^88^ Future research could, therefore, focus on the links between such social and commercial determinants of health, especially in view of the great increase in social media use observed during the COVID-19 pandemic.^89^ Fourth, the generalisability of the results to underexplored dimensions such as crime and safety and sense of place/belonging, to low-income and middle-income countries, to specific demographic subpopulations (eg, sex-stratified analysis), and potentially vulnerable groups based on race/ethnicity should be a topic of investigation.

Translating our findings to policy implications is notoriously challenging, especially given the largely observational nature of the evidence. Nevertheless, we demonstrated consistent evidence on the association between economic disadvantage and increased CMD risk, which reinforces the clear health potential of interventions and policies aimed at reducing poverty and deprivation. For instance, the Moving To Opportunity study successfully demonstrated that moving from high-poverty to low-poverty neighbourhoods reduced the risk of type 2 diabetes after 10–15 years.^47^ In addition, public policies and interventions should target social and norms, for instance, through early-life or marital social relationship interventions^90 91^ and through the screening of social circumstances (eg, ‘polysocial risk scores’^92^ or psychosocial screening protocols^93^) within the healthcare context. Yet, it may be difficult to put social relationships high on the list of public health priorities.^94^

In conclusion, findings from 14 meta-analyses across 180 associations suggest that worse social environment is associated with an increased risk of CMDs, particularly the association between economic and social disadvantage and hypertensive diseases, type 2 diabetes mellitus and heart failure, and between social relationships and norms and ischaemic heart disease. However, there was high heterogeneity within the meta-analyses and the existing literature varies notably in terminology and operationalisation of social environment measures. Moreover, certain social environmental dimensions such as social cohesion and social capital, crime and safety, civic participation and engagement, disorder and incivilities, and sense of place/belonging are underexplored in relation to CMD risk.

## supplementary material

10.1136/bmjopen-2023-079987 online supplemental file 1

10.1136/bmjopen-2023-079987 online supplemental file 2

10.1136/bmjopen-2023-079987 online supplemental file 3

10.1136/bmjopen-2023-079987 online supplemental file 4

10.1136/bmjopen-2023-079987 online supplemental file 5

10.1136/bmjopen-2023-079987 online supplemental file 6

10.1136/bmjopen-2023-079987 online supplemental file 7

10.1136/bmjopen-2023-079987 online supplemental file 8

10.1136/bmjopen-2023-079987 online supplemental file 9

## Data Availability

No data are available.
