# Supplementary material for: Associations between dimensions of the social environment and cardiometabolic health outcomes: a systematic review and meta-analysis
Source: BMJ Open. 2024 Aug 28;14(8):e079987. doi: 10.1136/bmjopen-2023-079987 (PMC11367359; doi:10.1136/bmjopen-2023-079987)
Supplement: online supplemental file 1 [file bmjopen-14-8-s001.docx]

**List of Supplemental Material**

**Supplementary Table 1.** Characteristics of included studies

**Supplementary Table 2.** Effect estimates of included studies

**Supplementary Table 3a-3e**. Quality assessment of included studies

**Supplementary Table 4.** Summary pooled effects and between-study variance estimates and 95% confidence intervals from meta-analysis models (middle-income countries only)

**Supplementary Table 5.** Summary pooled effects and between-study variance estimates and 95% confidence intervals from meta-analysis models (longitudinal studies only)

**Supplementary Table 6.** Summary pooled effects and between-study variance estimates and 95% confidence intervals from meta-analysis models (odds ratio only)

**Supplementary Figure 1A-1N** Overall Meta-Analysis Figures

**Supplementary Figure 2A-2I** Funnel Plots Figures

**Supplementary File 1** Full search strategies for all databases

**Supplementary File 2** List of studies excluded during full text screening, with reasons for exclusion
