# Supplementary material for: Associations between dimensions of the social environment and cardiometabolic health outcomes: a systematic review and meta-analysis
Source: BMJ Open. 2024 Aug 28;14(8):e079987. doi: 10.1136/bmjopen-2023-079987 (PMC11367359; doi:10.1136/bmjopen-2023-079987)
Supplement: online supplemental file 2 [file bmjopen-14-8-s002.pdf]

PubMed History and Search Details - 28 February 2024

Search	PubMed Query – 28 February 2024	Results
#5	#4 NOT ("Animals"[Mesh] NOT "Humans"[Mesh]) NOT (rats[ti] OR rat[ti] rodent*[ti] OR mice*[ti])	5,981
#4	#1 AND #2 AND #3	6,053
#3	"state-level"[tiab] OR "regional-level"[tiab] OR "region-level"[tiab] OR "county-level"[tiab] OR "district-level"[tiab] OR "city-level"[tiab] OR "municipality-level"[tiab] OR "town-level"[tiab] OR neighborhood*[tiab] OR neighbourhood*[tiab] OR "community-level"[tiab] OR "community-based"[tiab] OR "residential-level"[tiab] OR "residential-area"[tiab] OR "census block*"[tiab] OR "geographic region*"[tiab] OR "geographical region*"[tiab] OR "geographic-level"[tiab] OR "geographic-scale"[tiab] OR "spatial scale"[tiab] OR "area-level"[tiab] OR "area-based"[tiab] OR "local-level"[tiab] OR "local-area"[tiab] OR "group-level"[tiab] OR "social determinants"[tiab] OR ((social[tiab] OR psychosocial[tiab]) AND (environment[tiab] OR environmental[tiab])) OR "social group*"[tiab] OR "community group*"[tiab] OR "network member*"[tiab] OR "social relation*"[tiab] OR "social contact*"[tiab] OR "network index"[tiab] OR "social network*"[tiab] OR "family network*"[tiab] OR "family members"[tiab] OR "personal network*"[tiab] OR "friend network*"[tiab] OR "friends network*"[tiab] OR "health network*"[tiab] OR "peer group*"[tiab] OR "interpersonal relation*"[tiab] OR (social[tiab] AND ("network composition"[tiab] OR "network size*"[tiab] OR "network attribute*"[tiab] OR "network characteristic*"[tiab] OR "network structure*"[tiab] OR "network density"[tiab] OR "neighborhood network*"[tiab] OR "neighbourhood network*"[tiab]))	342,020
#2	"sense of belonging"[tiab] OR "sense of place"[tiab] OR "sense of community"[tiab] OR "feeling of belonging"[tiab] OR "feelings of belonging"[tiab] OR "social belonging"[tiab] OR "community belonging"[tiab] OR belongingness[tiab] OR "Place Attachment"[tiab] OR crime[tiab] OR crimes[tiab] OR violence[tiab] OR "sense of safety"[tiab] OR "social disorder*"[tiab] OR "public disorder*"[tiab] OR delinquen*[tiab] OR nuisance*[tiab] OR incivilit*[tiab] OR hostility[tiab] OR "litter"[tiab] OR "grafitti"[tiab] OR "empty house*"[tiab] OR "unoccupied hous*"[tiab] OR "abandoned hous*"[tiab] OR "area deprivation"[tiab] OR (("neighborhood"[tiab] OR "neighbourhood"[tiab]) AND (deprivation[tiab] OR deprived[tiab])) OR "Social Participation"[Mesh] OR "Social Isolation"[Mesh] OR vote[tiab] OR "collective action*"[tiab] OR "community empowerment"[tiab] OR "civic empowerment"[tiab] OR "social empowerment"[tiab] OR "community involvement*"[tiab] OR "civic involvement*"[tiab] OR "social involvement*"[tiab] OR "community	478,649

Search	PubMed Query – 28 February 2024	Results
	participation"[tiab] OR "civic participation"[tiab] OR "social participation"[tiab] OR "community engagement"[tiab] OR "civic engagement"[tiab] OR "social engagement"[tiab] OR "social isolation"[tiab] OR "Community Participation"[Mesh:NoExp] OR "community action*"[tiab] OR "public participation"[tiab] OR "public involvement"[tiab] OR "Social Desirability"[Mesh] OR "Social Norms"[Mesh] OR "Social Networking"[Mesh] OR "Social Conformity"[Mesh] OR "Social Dominance"[Mesh] OR "Social Identification"[Mesh] OR "social support*"[tiab] OR "social network*"[tiab] OR "social contact*"[tiab] OR "social tie*"[tiab] OR "social relation*"[tiab] OR "social interaction*"[tiab] OR "social norm*"[tiab] OR "cultural norm*"[tiab] OR "social desirabilit*"[tiab] OR "social adjustment"[tiab] OR "social conformit*"[tiab] OR "social dominanc*"[tiab] OR "social identification"[tiab] OR "Social Capital"[Mesh] OR "social capital"[tiab] OR "social control*"[tiab] OR "social cohesion"[tiab] OR "collective efficac*"[tiab] OR "collective effectiveness"[tiab] OR "group cohesion"[tiab] OR "social connected*"[tiab] OR (social[tiab] AND trust[tiab]) OR "Social Segregation"[Mesh] OR "Social Integration"[Mesh] OR "Social Discrimination"[Mesh] OR "social segregation*"[tiab] OR "social Integration"[tiab] OR "residential segregation*"[tiab] OR "ethnic composition*"[tiab] OR "ethnic densit*"[tiab] OR "ethnic concentration*"[tiab] OR "ethnic discriminati*"[tiab] OR "racial discriminati*"[tiab] OR "racial segregation*"[tiab] OR "racial densit*"[tiab] OR "racial composition*"[tiab] OR "racial concentration*"[tiab] OR "social discrimination"[tiab] OR "xenophobia"[tiab] OR "Poverty"[Mesh] OR "Social Class"[Mesh] OR "poverty"[tiab] OR "social class*"[tiab] OR "social gradient*"[tiab] OR "social patterning"[tiab] OR "social hierarch*"[tiab] OR "social depriv*"[tiab] OR "social inequalit*"[tiab] OR "social inequit*"[tiab] OR (("socioeconomic"[tiab] OR "socio-economic"[tiab]) AND ("factor*"[tiab] OR "difference*"[tiab] OR position*[tiab] OR status[tiab] OR disparit*[tiab] OR inequalit*[tiab] OR inequit*[tiab] OR adversit*[tiab] OR deprivation[tiab] OR deprived[tiab]))	
`#1	"Cardiovascular Diseases"[MeSH:NoExp] OR "Heart Diseases"[Mesh:NoExp] OR "Myocardial Ischemia"[Mesh] OR "Heart Failure"[Mesh] OR "Vascular Diseases"[Mesh:NoExp] OR "Cerebrovascular Disorders" [Mesh] OR "cardiovascular disease*" [tiab] OR "cardiometabolic*"[tiab] OR "cardiac disease*"[tiab] OR "cardiac disorder*"[tiab] OR "heart disease*"[tiab] OR "heart disorder*"[tiab] OR "vascular disease*"[tiab] OR "heart failure*" [tiab] OR "cardiac failure*" [tiab] OR "myocardial failure*"[tiab] OR "myocardial infarction*"[tiab] OR "heart infarction*"[tiab] OR "coronary disease*"[tiab] OR "artery disease*"[tiab] OR "aortic disease*" [tiab] OR "stroke*"[tiab] OR "myocardial ischaemia"[tiab] OR "myocardial ischemia"[tiab] OR "brain vascular accident*"[tiab] OR "cerebrovascular disease*"[tiab] OR	3,063,051

Search	PubMed Query – 28 February 2024	Results
	"cerebrovascular disorder*"[tiab] OR "atheroscleros*"[tiab] OR "cerebrovascular accident*"[tiab] OR CVD[tiab] OR CHD[tiab] OR CAD[tiab] OR IHD[tiab] OR "Blood pressure"[Mesh] OR "Hypertension"[Mesh] OR "Prehypertension"[Mesh] OR "blood pressure"[tiab] OR "diastolic pressure"[tiab] OR "systolic pressure"[tiab] OR "hypertensi*"[tiab] OR "prehypertensi*"[tiab] OR "Dyslipidemias"[Mesh] OR "Hypercholesterolemia"[Mesh] OR "Hyperlipidemias"[Mesh] OR "Triglycerides"[Mesh] OR "Cholesterol"[Mesh] OR "Lipoproteins, HDL"[Mesh] OR "Lipoproteins, LDL"[Mesh] OR "Lipoproteins, VLDL"[Mesh] OR "Lipoproteins, IDL"[Mesh] OR "dyslipem*"[tiab] OR "dyslipidem*"[tiab] OR "dyslipaem*"[tiab] OR "dyslipidaem*"[tiab] OR "hyperlipemi*"[tiab] OR "hyperlipid*"[tiab] OR "lipidemi*"[tiab] OR "lipidaemi*"[tiab] OR hypercholesterolemi*[tiab] OR "hypercholesteremi*"[tiab] OR "triglyceride*"[tiab] OR "hypertriglyceridemi*"[tiab] OR "cholesterol"[tiab] OR "hdl"[tiab] OR "ldl"[tiab] OR "vldl"[tiab] OR "idl"[tiab] OR "density lipoprotein*"[tiab] OR "hdl lipoprotein*"[tiab] OR "low density lipoprotein*"[tiab] OR "high density lipoprotein*"[tiab] OR "very low lipoprotein*"[tiab] OR "Diabetes Mellitus"[Mesh:NoExp] OR "Diabetes Mellitus, Type 2"[Mesh] OR "Prediabetic State"[Mesh] OR "Insulin Resistance"[Mesh] OR "diabet*"[tiab] OR "T2DM"[tiab] OR "type 2 dm"[tiab] OR "type II dm"[tiab] OR "dm type 2"[tiab] OR "dm type II"[tiab] OR "NIDDM"[tiab] OR "insulin resistan*"[tiab] OR "insulin sensitiv*"[tiab] OR "insulin insensitiv*"[tiab] OR "prediabet*"[tiab] OR "Glucose Intolerance"[Mesh] OR "Blood Glucose"[Mesh] OR "Hyperglycemia"[Mesh] OR "Glycated Hemoglobin A"[Mesh] OR "glucose intoleran*"[tiab] OR "glucose toleran*"[tiab] OR "glucose dysregulation"[tiab] OR "blood glucose"[tiab] OR "plasma glucose"[tiab] OR "glucose level*"[tiab] OR "glucose blood"[tiab] OR "fasting glucose"[tiab] OR "blood sugar"[tiab] OR "hyperglycem*"[tiab] OR "hyperglycaem*"[tiab] OR "Hb A1c"[tiab] OR "HbA1c"[tiab] OR "Hemoglobin A1c"[tiab] OR "igt"[tiab]	

Web of Science History and Search Details- 28 February 2024

Search	Web of Science Query – 28 February 2024	Results
#5	#4 NOT TI = (animal* OR rats OR rat rodent* OR mice*)	4,366
#4	#1 AND #2 AND #3	4,389
#3	TI = ("state-level" OR "regional-level" OR "region-level" OR "county-level" OR "district-level" OR "city-level" OR "municipality-level" OR "town-level" OR neighborhood* OR neighbourhood* OR "community-level" OR "community-based" OR "residential-level" OR "residential-area" OR "census block*" OR "geographic region*" OR "geographical region*" OR	554,653

Search	Web of Science Query – 28 February 2024	Results
	"geographic-level" OR "geographic-scale" OR "spatial scale" OR "area-level" OR "area-based" OR "local-level" OR "local-area" OR "group-level" OR "social determinants" OR ((social OR psychosocial) NEAR/3 (environment OR environmental)) OR "social group*" OR "community group*" OR "network member*" OR "social relation*" OR "social contact*" OR "network index" OR "social network*" OR "family network*" OR "family members" OR "personal network*" OR "friend network*" OR "friends network*" OR "health network*" OR "peer group*" OR "interpersonal relation*" OR (social AND ("network composition" OR "network size*" OR "network attribute*" OR "network characteristic*" OR "network structure*" OR "network density" OR "neighborhood network*" OR "neighbourhood network*"))) OR AB = ("state-level" OR "regional-level" OR "region-level" OR "county-level" OR "district-level" OR "city-level" OR "municipality-level" OR "town-level" OR neighborhood* OR neighbourhood* OR "community-level" OR "community-based" OR "residential-level" OR "residential-area" OR "census block*" OR "geographic region*" OR "geographical region*" OR "geographic-level" OR "geographic-scale" OR "spatial scale" OR "area-level" OR "area-based" OR "local-level" OR "local-area" OR "group-level" OR "social determinants" OR ((social OR psychosocial) NEAR/3 (environment OR environmental)) OR "social group*" OR "community group*" OR "network member*" OR "social relation*" OR "social contact*" OR "network index" OR "social network*" OR "family network*" OR "family members" OR "personal network*" OR "friend network*" OR "friends network*" OR "health network*" OR "peer group*" OR "interpersonal relation*" OR (social AND ("network composition" OR "network size*" OR "network attribute*" OR "network characteristic*" OR "network structure*" OR "network density" OR "neighborhood network*" OR "neighbourhood network*"))) OR AK = ("state-level" OR "regional-level" OR "region-level" OR "county-level" OR "district-level" OR "city-level" OR "municipality-level" OR "town-level" OR neighborhood* OR neighbourhood* OR "community-level" OR "community-based" OR "residential-level" OR "residential-area" OR "census block*" OR "geographic region*" OR "geographical region*" OR "geographic-level" OR "geographic-scale" OR "spatial scale" OR "area-level" OR "area-based" OR "local-level" OR "local-area" OR "group-level" OR "social determinants" OR ((social OR psychosocial) NEAR/3 (environment OR environmental)) OR "social group*" OR "community group*" OR "network member*" OR "social relation*" OR "social contact*" OR "network index" OR "social network*" OR "family network*" OR "family members" OR "personal network*" OR "friend network*" OR "friends network*" OR "health network*" OR "peer group*" OR "interpersonal relation*" OR (social AND ("network composition" OR "network size*" OR "network attribute*" OR "network characteristic*" OR "network structure*" OR "network density" OR "neighborhood network*" OR "neighbourhood network*")))	

Search	Web of Science Query – 28 February 2024	Results
#2	TI = ("sense of belonging" OR "sense of place" OR "sense of community" OR "feeling* of belonging" OR "social belonging" OR "community belonging" OR “belongingness” OR "place attachment" OR “crime” OR “crimes” OR “violence” OR "sense of safety" OR "social disorder*" OR "public disorder*" OR delinquen* OR nuisance* OR incivilit* OR “hostility” OR "litter" OR "grafitti" OR "empty house*" OR "unoccupied hous*" OR "abandoned hous*" OR "area deprivation" OR (("neighborhood" OR "neighbourhood") NEAR/3 (“deprivation” OR “deprived”)) OR “vote” OR "collective action*" OR "community empowerment" OR "civic empowerment" OR "social empowerment" OR "community involvement*" OR "civic involvement*" OR "social involvement*" OR "community participation" OR "civic participation" OR "social participation" OR "community engagement" OR "civic engagement" OR "social engagement" OR "social isolation" OR "community action*" OR "public participation" OR "public involvement” OR "social support*" OR "social network*" OR "social contact*" OR "social tie*" OR "social relation*" OR "social interaction*" OR "social norm*" OR "cultural norm*" OR "social desirabilit*" OR "social adjustment" OR "social conformit*" OR "social dominanc*" OR "social identification" OR "social capital" OR "social control*" OR "social cohesion" OR "collective efficac*" OR "collective effectiveness" OR "group cohesion" OR "social connected*" OR (“social” AND “trust”) OR "social segregation*" OR "social Integration" OR "residential segregation*" OR "ethnic composition*" OR "ethnic densit*" OR "ethnic concentration*" OR "ethnic discriminati*" OR "racial discriminati*" OR "racial segregation*" OR "racial densit*" OR "racial composition*" OR "racial concentration*" OR "social discrimination" OR "xenophobia" OR "poverty" OR "social class*" OR "social gradient*" OR "social patterning" OR "social hierarch*" OR "social depriv*" OR "social inequalit*" OR "social inequit*" OR (("socioeconomic" OR "socio-economic") NEAR/3 ("factor*" OR "difference*" OR position* OR status OR disparit* OR inequalit* OR inequit* OR adversit* OR deprivation OR deprived))) OR AB =("sense of belonging" OR "sense of place" OR "sense of community" OR "feeling* of belonging" OR "social belonging" OR "community belonging" OR “belongingness” OR "place attachment" OR “crime” OR “crimes” OR “violence” OR "sense of safety" OR "social disorder*" OR "public disorder*" OR delinquen* OR nuisance* OR incivilit* OR “hostility” OR "litter" OR "grafitti" OR "empty house*" OR "unoccupied hous*" OR "abandoned hous*" OR "area deprivation" OR (("neighborhood" OR "neighbourhood") NEAR/3 (“deprivation” OR “deprived”)) OR “vote” OR "collective action*" OR "community empowerment" OR "civic empowerment" OR "social empowerment" OR "community involvement*" OR "civic involvement*" OR "social involvement*" OR "community participation" OR "civic participation" OR "social participation" OR "community engagement" OR "civic engagement" OR "social engagement" OR "social isolation" OR "community action*" OR	751,924

Search	Web of Science Query – 28 February 2024	Results
	"public participation" OR "public involvement" OR "social support*" OR "social network*" OR "social contact*" OR "social tie*" OR "social relation*" OR "social interaction*" OR "social norm*" OR "cultural norm*" OR "social desirabilit*" OR "social adjustment" OR "social conformit*" OR "social dominanc*" OR "social identification" OR "social capital" OR "social control*" OR "social cohesion" OR "collective efficac*" OR "collective effectiveness" OR "group cohesion" OR "social connected*" OR ("social" AND "trust") OR "social segregation*" OR "social Integration" OR "residential segregation*" OR "ethnic composition*" OR "ethnic densit*" OR "ethnic concentration*" OR "ethnic discriminati*" OR "racial discriminati*" OR "racial segregation*" OR "racial densit*" OR "racial composition*" OR "racial concentration*" OR "social discrimination" OR "xenophobia" OR "poverty" OR "social class*" OR "social gradient*" OR "social patterning" OR "social hierarch*" OR "social depriv*" OR "social inequalit*" OR "social inequit*" OR (("socioeconomic" OR "socio-economic") NEAR/3 ("factor*" OR "difference*" OR position* OR status OR disparit* OR inequalit* OR inequit* OR adversit* OR deprivation OR deprived))) OR AK= ("sense of belonging" OR "sense of place" OR "sense of community" OR "feeling* of belonging" OR "social belonging" OR "community belonging" OR "belongingness" OR "place attachment" OR "crime" OR "crimes" OR "violence" OR "sense of safety" OR "social disorder*" OR "public disorder*" OR delinquen* OR nuisance* OR incivilit* OR "hostility" OR "litter" OR "grafitti" OR "empty house*" OR "unoccupied hous*" OR "abandoned hous*" OR "area deprivation" OR (("neighborhood" OR "neighbourhood") NEAR/3 ("deprivation" OR "deprived")) OR "vote" OR "collective action*" OR "community empowerment" OR "civic empowerment" OR "social empowerment" OR "community involvement*" OR "civic involvement*" OR "social involvement*" OR "community participation" OR "civic participation" OR "social participation" OR "community engagement" OR "civic engagement" OR "social engagement" OR "social isolation" OR "community action*" OR "public participation" OR "public involvement" OR "social support*" OR "social network*" OR "social contact*" OR "social tie*" OR "social relation*" OR "social interaction*" OR "social norm*" OR "cultural norm*" OR "social desirabilit*" OR "social adjustment" OR "social conformit*" OR "social dominanc*" OR "social identification" OR "social capital" OR "social control*" OR "social cohesion" OR "collective efficac*" OR "collective effectiveness" OR "group cohesion" OR "social connected*" OR ("social" AND "trust") OR "social segregation*" OR "social Integration" OR "residential segregation*" OR "ethnic composition*" OR "ethnic densit*" OR "ethnic concentration*" OR "ethnic discriminati*" OR "racial discriminati*" OR "racial segregation*" OR "racial densit*" OR "racial composition*" OR "racial concentration*" OR "social discrimination" OR "xenophobia" OR "poverty" OR "social class*" OR "social gradient*" OR "social patterning" OR "social hierarch*" OR "social depriv*" OR "social	

Search	Web of Science Query – 28 February 2024	Results
	inequalit*" OR "social inequit*" OR (("socioeconomic" OR "socio-economic") NEAR/3 ("factor*" OR "difference*" OR position* OR status OR disparit* OR inequalit* OR inequit* OR adversit* OR deprivation OR deprived)))	
#1	TI = ("cardiovascular disease*" OR "cardiometabolic*" OR "cardiac disease*" OR "cardiac disorder*" OR "heart disease*" OR "heart disorder*" OR "vascular disease*" OR "heart failure*" OR "cardiac failure*" OR "myocardial failure*" OR "myocardial infarction*" OR "heart infarction*" OR "coronary disease*" OR "artery disease*" OR "aortic disease*" OR "stroke*" OR "myocardial ischaemia" OR "myocardial ischemia" OR "brain vascular accident*" OR "cerebrovascular disease*" OR "cerebrovascular disorder*" OR "atheroscleros*" OR "cerebrovascular accident*" OR CVD OR CHD OR CAD OR IHD OR "blood pressure" OR "diastolic pressure" OR "systolic pressure" OR "hypertensi*" OR "prehypertensi*" OR "dyslipem*" OR "dyslipidem*" OR "dyslipaem*" OR "dyslipidaem*" OR "hyperlipemi*" OR "hyperlipid*" OR "lipidemi*" OR "lipidaemi*" OR hypercholesterolemi* OR "hypercholesteremi*" OR "triglyceride*" OR "hypertriglyceridemi*" OR "cholesterol" OR "hdl" OR "ldl" OR "vldl" OR "idl" OR "density lipoprotein*" OR "hdl lipoprotein*" OR "low density lipoprotein*" OR "high density lipoprotein*" OR "very low lipoprotein*" OR "diabet*" OR "T2DM" OR "type 2 dm" OR "type II dm" OR "dm type 2" OR "dm type II" OR "NIDDM" OR "insulin resistan*" OR "insulin sensitiv*" OR "insulin insensitiv*" OR "prediabet*" OR "glucose intoleran*" OR "glucose toleran*" OR "glucose dysregulation" OR "blood glucose" OR "plasma glucose" OR "glucose level*" OR "glucose blood" OR "fasting glucose" OR "blood sugar" OR "hyperglycem*" OR "hyperglycaem*" OR "Hb A1c" OR "HbA1c" OR "Hemoglobin A1c" OR "igt") OR AB = ("cardiovascular disease*" OR "cardiometabolic*" OR "cardiac disease*" OR "cardiac disorder*" OR "heart disease*" OR "heart disorder*" OR "vascular disease*" OR "heart failure*" OR "cardiac failure*" OR "myocardial failure*" OR "myocardial infarction*" OR "heart infarction*" OR "coronary disease*" OR "artery disease*" OR "aortic disease*" OR "stroke*" OR "myocardial ischaemia" OR "myocardial ischemia" OR "brain vascular accident*" OR "cerebrovascular disease*" OR "cerebrovascular disorder*" OR "atheroscleros*" OR "cerebrovascular accident*" OR CVD OR CHD OR CAD OR IHD OR "blood pressure" OR "diastolic pressure" OR "systolic pressure" OR "hypertensi*" OR "prehypertensi*" OR "dyslipem*" OR "dyslipidem*" OR "dyslipaem*" OR "dyslipidaem*" OR "hyperlipemi*" OR "hyperlipid*" OR "lipidemi*" OR "lipidaemi*" OR hypercholesterolemi* OR "hypercholesteremi*" OR "triglyceride*" OR "hypertriglyceridemi*" OR "cholesterol" OR "hdl" OR "ldl" OR "vldl" OR "idl" OR "density lipoprotein*" OR "hdl lipoprotein*" OR "low density lipoprotein*" OR "high density lipoprotein*" OR "very low lipoprotein*" OR "diabet*" OR "T2DM" OR "type 2 dm" OR "type II dm" OR "dm type	2,600,913

Search	Web of Science Query – 28 February 2024	Results
	2" OR "dm type II" OR "NIDDM" OR "insulin resistan*" OR "insulin sensitiv*" OR "insulin insensitiv*" OR "prediabet*" OR "glucose intoleran*" OR "glucose toleran*" OR "glucose dysregulation" OR "blood glucose" OR "plasma glucose" OR "glucose level*" OR "glucose blood" OR "fasting glucose" OR "blood sugar" OR "hyperglycem*" OR "hyperglycaem*" OR "Hb A1c" OR "HbA1c" OR "Hemoglobin A1c" OR "igt") OR AK = ("cardiovascular disease*" OR "cardiometabolic*" OR "cardiac disease*" OR "cardiac disorder*" OR "heart disease*" OR "heart disorder*" OR "vascular disease*" OR "heart failure*" OR "cardiac failure*" OR "myocardial failure*" OR "myocardial infarction*" OR "heart infarction*" OR "coronary disease*" OR "artery disease*" OR "aortic disease*" OR "stroke*" OR "myocardial ischaemia" OR "myocardial ischemia" OR "brain vascular accident*" OR "cerebrovascular disease*" OR "cerebrovascular disorder*" OR "atheroscleros*" OR "cerebrovascular accident*" OR CVD OR CHD OR CAD OR IHD OR "blood pressure" OR "diastolic pressure" OR "systolic pressure" OR "hypertensi*" OR "prehypertensi*" OR "dyslipem*" OR "dyslipidem*" OR "dyslipaem*" OR "dyslipidaem*" OR "hyperlipemi*" OR "hyperlipid*" OR "lipidemi*" OR "lipidaemi*" OR hypercholesterolemi* OR "hypercholesteremi*" OR "triglyceride*" OR "hypertriglyceridemi*" OR "cholesterol" OR "hdl" OR "ldl" OR "vldl" OR "idl" OR "density lipoprotein*" OR "hdl lipoprotein*" OR "low density lipoprotein*" OR "high density lipoprotein*" OR "very low lipoprotein*" OR "diabet*" OR "T2DM" OR "type 2 dm" OR "type II dm" OR "dm type 2" OR "dm type II" OR "NIDDM" OR "insulin resistan*" OR "insulin sensitiv*" OR "insulin insensitiv*" OR "prediabet*" OR "glucose intoleran*" OR "glucose toleran*" OR "glucose dysregulation" OR "blood glucose" OR "plasma glucose" OR "glucose level*" OR "glucose blood" OR "fasting glucose" OR "blood sugar" OR "hyperglycem*" OR "hyperglycaem*" OR "Hb A1c" OR "HbA1c" OR "Hemoglobin A1c" OR "igt")	

Scopus History and Search Details - 28 February 2024

Search	Scopus Query – 28 February 2024	Results
#5	#4 AND NOT TITLE (animal* OR rats OR rat OR rodent* OR mice*)	5,550
#4	#1 AND #2 AND #3	5,579
#3	TITLE-ABS ("state-level" OR "regional-level" OR "region-level" OR "county-level" OR "district-level" OR "city-level" OR "municipality-level" OR "town-level" OR neighborhood* OR neighbourhood* OR "community-level" OR "community-based" OR "residential-level" OR "residential-area" OR "census block*" OR "geographic region*" OR "geographical region*" OR "geographic-level" OR "geographic-scale" OR "spatial scale" OR "area-level" OR "area-based" OR "local-level" OR "local-area" OR "group-level"	963,262

Search	Scopus Query – 28 February 2024	Results
	OR "social determinants" OR ((social OR psychosocial) W/3 (environment OR environmental)) OR "social group*" OR "community group*" OR "network member*" OR "social relation*" OR "social contact*" OR "network index" OR "social network*" OR "family network*" OR "family members" OR "personal network*" OR "friend network*" OR "friends network*" OR "health network*" OR "peer group*" OR "interpersonal relation*" OR (social AND ("network composition" OR "network size*" OR "network attribute*" OR "network characteristic*" OR "network structure*" OR "network density" OR "neighborhood network*" OR "neighbourhood network*"))) OR AUTHKEY ("state-level" OR "regional-level" OR "region-level" OR "county-level" OR "district-level" OR "city-level" OR "municipality-level" OR "town-level" OR neighborhood* OR neighbourhood* OR "community-level" OR "community-based" OR "residential-level" OR "residential-area" OR "census block*" OR "geographic region*" OR "geographical region*" OR "geographic-level" OR "geographic-scale" OR "spatial scale" OR "area-level" OR "area-based" OR "local-level" OR "local-area" OR "group-level" OR "social determinants" OR ((social OR psychosocial) W/3 (environment OR environmental)) OR "social group*" OR "community group*" OR "network member*" OR "social relation*" OR "social contact*" OR "network index" OR "social network*" OR "family network*" OR "family members" OR "personal network*" OR "friend network*" OR "friends network*" OR "health network*" OR "peer group*" OR "interpersonal relation*" OR (social AND ("network composition" OR "network size*" OR "network attribute*" OR "network characteristic*" OR "network structure*" OR "network density" OR "neighborhood network*" OR "neighbourhood network*")))	
#2	TITLE-ABS ("sense of belonging" OR "sense of place" OR "sense of community" OR "feeling* of belonging" OR "social belonging" OR "community belonging" OR “belongingness” OR "place attachment" OR “crime” OR “crimes” OR “violence” OR "sense of safety" OR "social disorder*" OR "public disorder*" OR delinquen* OR nuisance* OR incivilit* OR “hostility” OR "litter" OR "grafitti" OR "empty house*" OR "unoccupied hous*" OR "abandoned hous*" OR "area deprivation" OR (("neighborhood" OR "neighbourhood") W/3 (“deprivation” OR “deprived”)) OR “vote” OR "collective action*" OR "community empowerment" OR "civic empowerment" OR "social empowerment" OR "community involvement*" OR "civic involvement*" OR "social involvement*" OR "community participation" OR "civic participation" OR "social participation" OR "community engagement" OR "civic engagement" OR "social engagement" OR "social isolation" OR "community action*" OR "public participation" OR "public involvement” OR "social support*" OR "social network*" OR "social contact*" OR "social tie*" OR "social relation*" OR "social interaction*" OR "social norm*" OR "cultural norm*" OR "social desirabilit*" OR "social adjustment" OR "social conformit*" OR	1,085,961

Search	Scopus Query – 28 February 2024	Results
	"social dominanc*" OR "social identification" OR "social capital" OR "social control*" OR "social cohesion" OR "collective efficac*" OR "collective effectiveness" OR "group cohesion" OR "social connected*" OR (“social” AND “trust”) OR "social segregation*" OR "social Integration" OR "residential segregation*" OR "ethnic composition*" OR "ethnic densit*" OR "ethnic concentration*" OR "ethnic discriminati*" OR "racial discriminati*" OR "racial segregation*" OR "racial densit*" OR "racial composition*" OR "racial concentration*" OR "social discrimination" OR "xenophobia" OR "poverty" OR "social class*" OR "social gradient*" OR "social patterning" OR "social hierarch*" OR "social depriv*" OR "social inequalit*" OR "social inequit*" OR (("socioeconomic" OR "socio-economic") W/3 ("factor*" OR "difference*" OR position* OR status OR disparit* OR inequalit* OR inequit* OR adversit* OR deprivation OR deprived))) OR AUTHKEY ("sense of belonging" OR "sense of place" OR "sense of community" OR "feeling* of belonging" OR "social belonging" OR "community belonging" OR “belongingness” OR "place attachment" OR “crime” OR “crimes” OR “violence” OR "sense of safety" OR "social disorder*" OR "public disorder*" OR delinquen* OR nuisance* OR incivilit* OR “hostility” OR "litter" OR "grafitti" OR "empty house*" OR "unoccupied hous*" OR "abandoned hous*" OR "area deprivation" OR (("neighborhood" OR "neighbourhood") W/3 (“deprivation” OR “deprived”)) OR “vote” OR "collective action*" OR "community empowerment" OR "civic empowerment" OR "social empowerment" OR "community involvement*" OR "civic involvement*" OR "social involvement*" OR "community participation" OR "civic participation" OR "social participation" OR "community engagement" OR "civic engagement" OR "social engagement" OR "social isolation" OR "community action*" OR "public participation" OR "public involvement” OR "social support*" OR "social network*" OR "social contact*" OR "social tie*" OR "social relation*" OR "social interaction*" OR "social norm*" OR "cultural norm*" OR "social desirabilit*" OR "social adjustment" OR "social conformit*" OR "social dominanc*" OR "social identification" OR "social capital" OR "social control*" OR "social cohesion" OR "collective efficac*" OR "collective effectiveness" OR "group cohesion" OR "social connected*" OR (“social” AND “trust”) OR "social segregation*" OR "social Integration" OR "residential segregation*" OR "ethnic composition*" OR "ethnic densit*" OR "ethnic concentration*" OR "ethnic discriminati*" OR "racial discriminati*" OR "racial segregation*" OR "racial densit*" OR "racial composition*" OR "racial concentration*" OR "social discrimination" OR "xenophobia" OR "poverty" OR "social class*" OR "social gradient*" OR "social patterning" OR "social hierarch*" OR "social depriv*" OR "social inequalit*" OR "social inequit*" OR (("socioeconomic" OR "socio-economic") W/3 ("factor*" OR "difference*" OR position* OR status OR disparit* OR inequalit* OR inequit* OR adversit* OR deprivation OR deprived)))	

Search	Scopus Query – 28 February 2024	Results
#1	TITLE-ABS ("cardiovascular disease*" OR "cardiometabolic*" OR "cardiac disease*" OR "cardiac disorder*" OR "heart disease*" OR "heart disorder*" OR "vascular disease*" OR "heart failure*" OR "cardiac failure*" OR "myocardial failure*" OR "myocardial infarction*" OR "heart infarction*" OR "coronary disease*" OR "artery disease*" OR "aortic disease*" OR "stroke*" OR "myocardial ischaemia" OR "myocardial ischemia" OR "brain vascular accident*" OR "cerebrovascular disease*" OR "cerebrovascular disorder*" OR "atheroscleros*" OR "cerebrovascular accident*" OR {CVD} OR {CHD} OR {CAD} OR {IHD} OR "blood pressure" OR "diastolic pressure" OR "systolic pressure" OR "hypertensi*" OR "prehypertensi*" OR "dyslipem*" OR "dyslipidem*" OR "dyslipaem*" OR "dyslipidaem*" OR "hyperlipemi*" OR "hyperlipid*" OR "lipidemi*" OR "lipidaemi*" OR hypercholesterolemi* OR "hypercholesteremi*" OR "triglyceride*" OR "hypertriglyceridemi*" OR "cholesterol" OR {hdl} OR {ldl} OR {vldl} OR {idl} OR "density lipoprotein*" OR "hdl lipoprotein*" OR "low density lipoprotein*" OR "high density lipoprotein*" OR "very low lipoprotein*" OR "diabet*" OR {T2DM} OR {type 2 dm} OR {type II dm} OR {dm type 2} OR {dm type II} OR {NIDDM} OR "insulin resistan*" OR "insulin sensitiv*" OR "insulin insensitiv*" OR "prediabet*" OR "glucose intoleran*" OR "glucose toleran*" OR "glucose dysregulation" OR "blood glucose" OR "plasma glucose" OR "glucose level*" OR "glucose blood" OR "fasting glucose" OR "blood sugar" OR "hyperglycem*" OR "hyperglycaem*" OR {Hb A1c} OR {HbA1c} OR "Hemoglobin A1c" OR {igt}) OR AUTHKEY ("cardiovascular disease*" OR "cardiometabolic*" OR "cardiac disease*" OR "cardiac disorder*" OR "heart disease*" OR "heart disorder*" OR "vascular disease*" OR "heart failure*" OR "cardiac failure*" OR "myocardial failure*" OR "myocardial infarction*" OR "heart infarction*" OR "coronary disease*" OR "artery disease*" OR "aortic disease*" OR "stroke*" OR "myocardial ischaemia" OR "myocardial ischemia" OR "brain vascular accident*" OR "cerebrovascular disease*" OR "cerebrovascular disorder*" OR "atheroscleros*" OR "cerebrovascular accident*" OR {CVD} OR {CHD} OR {CAD} OR {IHD} OR "blood pressure" OR "diastolic pressure" OR "systolic pressure" OR "hypertensi*" OR "prehypertensi*" OR "dyslipem*" OR "dyslipidem*" OR "dyslipaem*" OR "dyslipidaem*" OR "hyperlipemi*" OR "hyperlipid*" OR "lipidemi*" OR "lipidaemi*" OR hypercholesterolemi* OR "hypercholesteremi*" OR "triglyceride*" OR "hypertriglyceridemi*" OR "cholesterol" OR {hdl} OR {ldl} OR {vldl} OR {idl} OR "density lipoprotein*" OR "hdl lipoprotein*" OR "low density lipoprotein*" OR "high density lipoprotein*" OR "very low lipoprotein*" OR "diabet*" OR {T2DM} OR {type 2 dm} OR {type II dm} OR {dm type 2} OR {dm type II} OR {NIDDM} OR "insulin resistan*" OR "insulin sensitiv*" OR "insulin insensitiv*" OR "prediabet*" OR "glucose intoleran*" OR "glucose toleran*" OR "glucose dysregulation" OR "blood glucose" OR "plasma glucose" OR "glucose level*" OR "glucose blood" OR "fasting glucose" OR "blood sugar" OR "hyperglycem*" OR "hyperglycaem*" OR	3,068,792

Search	Scopus Query – 28 February 2024	Results
	{Hb A1c} OR {HbA1c} OR "Hemoglobin A1c" OR {igt})	
