# Supplementary material for: Associations between dimensions of the social environment and cardiometabolic health outcomes: a systematic review and meta-analysis
Source: BMJ Open. 2024 Aug 28;14(8):e079987. doi: 10.1136/bmjopen-2023-079987 (PMC11367359; doi:10.1136/bmjopen-2023-079987)
Supplement: online supplemental file 3 [file bmjopen-14-8-s003.pdf]

Supplementary File 2. List of studies excluded during full text screening, with reason for exclusion

First Author	Year	Title	Exclusion criteria	DOI
Adjaye-Gbewonyo	2018	Income inequality and cardiovascular disease risk factors in a highly unequal country: a fixed-effects analysis from South Africa	outcome (included in risk factors review)	10.1186/s12939-018-0741-0
Ala	2004	Evidence for affluence-related hypertension in urban Brazil	exposure	10.1038/sj.jhh.1001750
Al-Bayan	2016	Neighborhood perceptions and hypertension among low-income black women: a qualitative study	design	10.1186/s12889-016-3741-2
Aliarzadeh	2014	Association between socio-economic status and hemoglobin A1c levels in a Canadian primary care adult population without diabetes	outcome (included in risk factors review)	10.1186/1471-2296-15-7
Al-Kandari	2011	Relationship of strength of social support and frequency of social contact with hypertension and general health status among older adults in the mobile care unit in Kuwait	exposure	10.1007/s10823-011-9139-9
Alvarado	2023	Influence of neighborhood-level social determinants of health on a heart-healthy lifestyle among Black church members: A mixed-methods study	outcome	10.1016/j.ahjo.2023.100273
Andell	2020	Neighborhood socioeconomic status and aortic stenosis: A Swedish study based on nationwide registries and an echocardiographic screening cohort	outcome (included in risk factors review)	10.1016/j.ijcard.2020.06.034
Anjana	2017	Prevalence of diabetes and prediabetes in 15 states of India: results from the ICMR-INDIAB population-based cross-sectional study	outcome	10.1016/s2213-8587(17)30174-2
Ardern	2007	Geographic and demographic variation in the prevalence of the metabolic syndrome in Canada	exposure	10.1016/S1499-2671(07)11009-1
Aslanyan	2003	Effect of area-based deprivation on the severity, subtype, and outcome of ischemic stroke	patient population	10.1161/01.Str.0000097610.12803.D7

Asteazaran	2017	Health inequalities and the impact on the prevalence of cardiovascular risk factors and chronic complications in Argentina: a study on national risk factors surveys	language	10.5867/medwave.2017.09.7083
Auchincloss	2007	Association of insulin resistance with distance to wealthy areas: the multi-ethnic study of atherosclerosis	outcome (included in risk factors review)	10.1093/aje/kwk028
Bagheri	2014	Undiagnosed diabetes from cross-sectional GP practice data: an approach to identify communities with high likelihood of undiagnosed diabetes	design	10.1136/bmjopen-2014-005305
Bagheri	2015	Community cardiovascular disease risk from cross-sectional general practice clinical data: a spatial analysis	outcome (included in risk factors review)	10.5888/pcd12.140379
Bagheri	2019	Identifying hotspots of type 2 diabetes risk using general practice data and geospatial analysis: an approach to inform policy and practice	outcome (included in risk factors review)	10.1071/py19043
Bajaj	2016	Daily social interactions, close relationships, and systemic inflammation in two samples: Healthy middle-aged and older adults	outcome (included in risk factors review)	10.1016/j.bbi.2016.06.004
Baldock	2012	Associations between resident perceptions of the local residential environment and metabolic syndrome	outcome (included in risk factors review)	10.1155/2012/589409
Baldock	2018	Gender-specific associations between perceived and objective neighbourhood crime and metabolic syndrome	outcome (included in risk factors review)	10.1371/journal.pone.0201336

Balog	2019	Social relations in late adolescence and incident coronary heart disease: a 38-year follow-up of the Swedish 1969-1970 Conscription Cohort	exposure	10.1136/bmjopen-2019-030880
Barber	2016	Double-jeopardy: The joint impact of neighborhood disadvantage and low social cohesion on cumulative risk of disease among African American men and women in the Jackson Heart Study	outcome (included in risk factors review)	10.1016/j.socscimed.2016.02.001
Barrington	2019	Sex Differences in Life-Course Neighborhood Racial Composition and Adult Hypertension	full text not available	-
Barth	1998	Development of the cardiovascular morbidity and mortality in East Germany after the political change	language	10.1007/bf02956782
Bartig	2023	[Health of people with selected citizenships in Germany: prevalence of non-communicable diseases and associated social as well as migration-related factors]	language	10.1007/s00103-023-03767-4
Berkman	1982	Social network analysis and coronary heart disease	not a primary study	10.1159/000406195
Bey	2023	Biological Age Mediates the Effects of Perceived Neighborhood Problems on Heart Failure Risk Among Black Persons	outcome	10.1007/s40615-022-01476-3
Bey	2024	The Relationship of Neighborhood Disadvantage, Biological Aging, and Psychosocial Risk and Resilience Factors in Heart Failure Incidence Among Black Persons: A Moderated Mediation Analysis	outcome	10.1093/geronb/gbad121

Bey	2023	Distinct moderating pathways for psychosocial risk and resilience in the association of neighborhood disadvantage with incident heart failure among Black persons	outcome	10.1016/j.ssmph.2023.101475
BezerradeSouza	2021	Multimorbidity and its associated factors among adults aged 50 and over: A cross-sectional study in 17 European countries	outcome	10.1371/journal.pone.0246623
Bhavsar	2018	Value of Neighborhood Socioeconomic Status in Predicting Risk of Outcomes in Studies That Use Electronic Health Record Data	outcome	10.1001/jamanetworkopen.2018.2716
Bhise	2018	Prevalence and correlates of hypertension in Maharashtra, India: A multilevel analysis	exposure	10.1371/journal.pone.0191948
Birmingham	2009	Social ties and cardiovascular function: an examination of relationship positivity and negativity during stress	exposure	10.1016/j.ijpsycho.2009.08.002
Bjerregaard	2011	How well does social variation mirror secular change in prevalence of cardiovascular risk factors in a country in transition?	exposure	10.1002/ajhb.21209
Bland	2000	Long term relations between earthquake experiences and coronary heart disease risk factors	outcome (included in risk factors review)	10.1093/oxfordjournals.aje.a010152
Bland	1991	Social network and blood pressure: a population study	outcome (included in risk factors review)	10.1097/00006842-199111000-00002

Bocquier	2011	Prevalence of treated diabetes: Geographical variations at the small-area level and their association with area-level characteristics. A multilevel analysis in Southeastern France	outcome	10.1016/j.diabet.2010.07.004
Borissova	2016	Higher Prevalence of Diabetes Mellitus and Impaired Glucose Tolerance Among the Rural Population in Bulgaria	exposure	10.14740/jem334w
Bosma	1997	Low job control and risk of coronary heart disease in Whitehall II (prospective cohort) study	exposure	10.1136/bmj.314.7080.558
Boylan	2017	Neighborhood SES is particularly important to the cardiovascular health of low SES individuals	outcome (included in risk factors review)	10.1016/j.socscimed.2017.07.005
Brännström	1993	Changing social patterns of risk factors for cardiovascular disease in a Swedish community intervention programme	exposure	10.1093/ije/22.6.1026
Brawner	2022	A Convergent Mixed Methods Study of Cardiovascular Disease Risk Factors among Young Black Men in the United States	outcome	10.18865/ed.32.3.169
Breckenkamp	2007	Health inequalities in Germany: do regional-level variables explain differentials in cardiovascular risk?	outcome (included in risk factors review)	10.1186/1471-2458-7-132
Brown	2011	Neighborhood disadvantage and ischemic stroke: the Cardiovascular Health Study (CHS)	outcome	10.1016/j.socscimed.2005.02.003
Brown	2005	Individual socio-economic status, community socio-economic status and stroke in New Zealand: a case control study	outcome	10.1161/strokeaha.111.622134

Browning	2012	Neighborhood stressors and cardiovascular health: crime and C-reactive protein in Dallas, USA	outcome (included in risk factors review)	10.1016/j.socscimed.2012.03.027
Bu	2021	Relationship between loneliness, social isolation and modifiable risk factors for cardiovascular disease: a latent class analysis	outcome (included in risk factors review)	10.1136/jech-2020-215539
Burgard	2021	An elastic net penalized small area model combining unit- and area-level data for regional hypertension prevalence estimation	design	10.1080/02664763.2020.1765323
Bush	2022	The influence of neighbourhood-level socioeconomic deprivation on developing type 2 diabetes in older men: a longitudinal analysis of the British Regional Heart Study cohort data	full text not available	-
Carels	1998	Effect of satisfaction with social support on blood pressure in normotensive and borderline hypertensive men and women	outcome (included in risk factors review)	10.1207/s15327558ijbm0501_6
Carson	2007	Cumulative socioeconomic status across the life course and subclinical atherosclerosis	outcome (included in risk factors review)	10.1016/j.annepidem.2006.07.009
Carter-Edwards	2018	Designing Faith-Based Blood Pressure Interventions to Reach Young Black Men	exposure	10.1016/j.amepre.2018.05.009
Casey	2018	Measures of SES for Electronic Health Record-based Research	failed contact	10.1016/j.amepre.2017.10.004
Casper	1991	Variation in the magnitude of black-white differences in stroke mortality by community occupational structure	outcome	10.1136/jech.45.4.302
Caspi	2006	Socially isolated children 20 years later: risk of cardiovascular disease	outcome (included in risk factors review)	10.1001/archpedi.160.8.805

Cathorall	2015	Neighborhood Disadvantage and Variations in Blood Pressure	outcome (included in risk factors review)	10.1080/19325037.2015.1055018
Cené	2012	Social isolation, vital exhaustion, and incident heart failure: findings from the Atherosclerosis Risk in Communities Study	exposure	10.1093/eurjhf/hfs064
Chaix	2007	Income change at retirement, neighbourhood-based social support, and ischaemic heart disease: results from the prospective cohort study Men born in 1914""	exposure	10.1097/01.ede.0000249573.22856.9a
Chaix	2007	Neighborhood socioeconomic deprivation and residential instability: effects on incidence of ischemic heart disease and survival after myocardial infarction	outcome	10.1016/j.socscimed.2006.10.018
Chaix	2010	Individual/neighborhood social factors and blood pressure in the RECORD Cohort Study: which risk factors explain the associations?	outcome (included in risk factors review)	10.1161/hypertensionaha.109.143206
Chaix	2008	Residential environment and blood pressure in the PRIME Study: is the association mediated by body mass index and waist circumference?	outcome (included in risk factors review)	10.1097/HJH.0b013e3282fd991f
Chambergo-Michilot	2021	Socioeconomic determinants of hypertension and prehypertension in Peru: Evidence from the Peruvian Demographic and Health Survey	exposure	10.1371/journal.pone.0245730

Chamik	2018	Associations between psychological stress and smoking, drinking, obesity, and high blood pressure in an upper middle-income country in the African region	exposure	10.1002/smi.2766
Chaparro	2018	Neighborhood deprivation and biomarkers of health in Britain: the mediating role of the physical environment	outcome (included in risk factors review)	10.1186/s12889-018-5667-3
Chau	2011	Analysis of spatio-temporal variations in stroke incidence and case-fatality in Hong Kong	failed contact	10.4081/gh.2011.153
Chen	2021	The effect of racial discrimination on mental and physical health: A propensity score weighting approach	exposure	10.1016/j.socscimed.2021.114308
Chichlowska	2008	Individual and neighborhood socioeconomic status characteristics and prevalence of metabolic syndrome: the Atherosclerosis Risk in Communities (ARIC) Study	outcome (included in risk factors review)	10.1097/PSY.0b013e318183a491
Chowdhury	2021	Changes in prevalence and risk factors of hypertension among adults in Bangladesh: An analysis of two waves of nationally representative surveys	exposure	10.1371/journal.pone.0259507
Christine	2017	Exposure to Neighborhood Foreclosures and Changes in Cardiometabolic Health: Results From MESA	exposure	10.1093/aje/kww186
Clark	2012	Latent constructs in psychosocial factors associated with cardiovascular disease: an examination by race and sex	exposure	10.3389/fpsyt.2012.00005

Clark	2012	Cardiovascular inflammation in healthy women: multilevel associations with state-level prosperity, productivity and income inequality	outcome (included in risk factors review)	10.1186/1471-2458-12-211
Clark	2013	Neighborhood disadvantage, neighborhood safety and cardiometabolic risk factors in African Americans: biosocial associations in the Jackson Heart study	outcome (included in risk factors review)	10.1371/journal.pone.0063254
Climie	2019	Individual and Neighborhood Deprivation and Carotid Stiffness	outcome (included in risk factors review)	10.1161/hypertensionaha.118.12186
Cohen	2019	A Swiss paradox" in the United States? Level of spatial aggregation changes the association between income inequality and morbidity for older Americans"	outcome	10.1186/s12942-019-0192-x
Cohn	2017	Impact of Individual and Neighborhood Factors on Cardiovascular Risk in White Hispanic and Non-Hispanic Women and Men	outcome (included in risk factors review)	10.1002/nur.21778
Colantonio	2016	Performance of the Atherosclerotic Cardiovascular Disease Pooled Cohort Risk Equations by Socioeconomic Status. The REasons for Geographic And Racial Differences in Stroke (REGARDS) Study	outcome	10.1161/JAHA.117.005676
Congdon	2020	A diabetes risk index for small areas in England	outcome	10.1016/j.healthplace.2020.102340
Consolazio	2021	Individual and Territorial Socioeconomic Inequalities in Type 2 Diabetes Mellitus in the City of Milan: A Multilevel Study	full text not available	10.1424/102290

Coulon	2016	Multilevel Associations of Neighborhood Poverty, Crime, and Satisfaction With Blood Pressure in African-American Adults	outcome (included in risk factors review)	10.1093/ajh/hpv060
Coulon	2016	The Association of Neighborhood Gene-Environment Susceptibility with Cortisol and Blood Pressure in African-American Adults	outcome (included in risk factors review)	10.1007/s12160-015-9737-9
Coyte	2022	Social relationships and the risk of incident heart failure: results from a prospective population-based study of older men	exposure	10.1093/ehjopen/oeab045
Cozier	2016	Neighborhood Socioeconomic Status in Relation to Serum Biomarkers in the Black Women's Health Study	outcome (included in risk factors review)	10.1007/s11524-016-0034-0
Creaven	2013	Social support and trait personality are independently associated with resting cardiovascular function in women	outcome (included in risk factors review)	10.1111/bjhp.12001
Cross	2019	Cross-sectional study of area-level disadvantage and glycaemic-related risk in community health service users in the Southern.IML Research (SIMLR) cohort	outcome (included in risk factors review)	10.1071/ah16298
Cubbin	2001	Neighborhood context and cardiovascular disease risk factors: the contribution of material deprivation	full text not available	10.1080/14034940500327935
Cubbin	2005	Protective and harmful effects of neighborhood-level deprivation on individual-level health knowledge, behavior changes, and risk of coronary heart disease	outcome (included in risk factors review)	10.1093/aje/kwi250

Curtis	2013	Using GIS and secondary data to target diabetes-related public health efforts	exposure	10.1177/003335491312800311
DaCosta	2017	Relationship between organizational support at work and risk of non-communicable chronic diseases in a health service	language	10.5327/Z1679443520176046
Dalton	2017	Accuracy of Cardiovascular Risk Prediction Varies by Neighborhood Socioeconomic Position: A Retrospective Cohort Study	exposure	10.7326/m16-2543
Danelia	2005	Psychosocial work environment and coronary heart disease	full text not available	-
Davis	1999	Association of negative and positive social ties with fibrinogen levels in young women	exposure	10.1037//0278-6133.18.2.131
De Moraes	2019	Sex and ethnicity modify the associations between individual and contextual socioeconomic indicators and ideal cardiovascular health: MESA study	outcome (included in risk factors review)	10.1093/pubmed/fdy145
Deans	2009	Differences in atherosclerosis according to area level socioeconomic deprivation: cross sectional, population based study	outcome (included in risk factors review)	10.1136/bmj.b4170
DeBacquer	2005	Perceived job stress and incidence of coronary events: 3-year follow-up of the Belgian Job Stress Project cohort	outcome	10.1093/aje/kwi040
Deguen	2010	A small-area ecologic study of myocardial infarction, neighborhood deprivation, and sex: a Bayesian modeling approach	outcome	10.1097/EDE.0b013e3181e09925

DeSilva	2012	Social, cultural and economical determinants of diabetes mellitus in Kalutara district, Sri Lanka: a cross sectional descriptive study	exposure	10.1007/s11524-022-00632-8
Dhand	2018	Social Network Trajectories in Myocardial Infarction Versus Ischemic Stroke	exposure	10.1161/jaha.117.008029
Diez-Roux	2017	Neighborhood Environments and Coronary Heart Disease: A Multilevel Analysis	duplicate	10.1093/aje/kwx113
Diez-Roux	2001	Neighborhood of residence and incidence of coronary heart disease	outcome	10.1056/nejm200107123450205
Diez-Roux	2002	Neighborhood characteristics and components of the insulin resistance syndrome in young adults: the coronary artery risk development in young adults (CARDIA) study	outcome (included in risk factors review)	10.2337/diacare.25.11.1976
Do	2011	Circadian rhythm of cortisol and neighborhood characteristics in a population-based sample: the Multi-Ethnic Study of Atherosclerosis	outcome (included in risk factors review)	10.1016/j.healthplace.2010.12.019
dos Santos	1994	Risk factors of coronary artery disease and their relationships with dietetic and social variables	full text not available	-
Dragano	2009	Subclinical coronary atherosclerosis and neighbourhood deprivation in an urban region	outcome (included in risk factors review)	10.1007/s10654-008-9292-9
Dressler	1997	The cultural construction of social support in Brazil: associations with health outcomes	exposure	10.1111/maq.12213

Dressler	2016	Culture and the Immune System: Cultural Consonance in Social Support and C-reactive Protein in Urban Brazil	exposure	10.1023/a:1005394416255
Dressler	1986	Social support and arterial pressure in a central Mexican community	full text not available	10.1097/00006842-198605000-00004
Drewnowski	2014	The geography of diabetes by census tract in a large sample of insured adults in King County, Washington, 2005-2006	outcome	10.5888/pcd11.140135
Duncan	2016	Perceived spatial stigma, body mass index and blood pressure: a global positioning system study among low-income housing residents in New York City	outcome (included in risk factors review)	10.4081/gh.2016.399
Durfey	2019	Neighborhood disadvantage and chronic disease management	Patient population	10.1111/1475-6773.13092
Eichinger	2015	How are physical activity behaviors and cardiovascular risk factors associated with characteristics of the built and social residential environment?	outcome (included in risk factors review)	10.1371/journal.pone.0126010
Eleazu	2023	Associations of Cumulative Perceived Stress with Cardiovascular Risk Factors and Outcomes: Findings from The Dallas Heart Study	outcome	10.1101/2023.06.15.23291460
Ellaway	2007	Is social participation associated with cardiovascular disease risk factors?	outcome (included in risk factors review)	10.1016/j.socscimed.2006.11.022
Engström	2001	Geographic distribution of stroke incidence within an urban population: Relations to socioeconomic circumstances and prevalence of cardiovascular risk factors	outcome	10.1046/j.1365-2796.2000.00663.x

Espinosa de Los Monteros	2008	Individual and area-based indicators of acculturation and the metabolic syndrome among low-income Mexican American women living in a border region	exposure	10.2105/ajph.2008.141903
Faerstein	2014	Race and perceived racism, education, and hypertension among Brazilian civil servants: the Pró-Saúde Study	exposure	10.1590/1809-4503201400060007
Farid	2021	THE RELATIONSHIP BETWEEN WORKING BEHAVIOR AND THE INCIDENCE OF TYPE II DIABETES MELLITUS OF WORKERS WITH THE SNEHANDU THEORY	exposure	10.20473/ijph.v16i2.2021.208-218
Feldman	2021	Subgroup Variation and Neighborhood Social Gradients-an Analysis of Hypertension and Diabetes Among Asian Patients (New York City, 2014-2017)	failed contact	10.1007/s40615-020-00779-7
Finkelstein	2008	The Prevalence of Diabetes Among Overweight and Obese Individuals is Higher in Poorer than in Richer Neighbourhoods	outcome	10.1016/S1499-2671(08)23009-1
Fleetcroft	2017	Outcomes and inequalities in diabetes from 2004/2005 to 2011/2012: English longitudinal study	outcome	10.3399/bjgp16X688381
Foraker	2019	Distribution of Cardiovascular Health by Individual- and Neighborhood-Level Socioeconomic Status: Findings From the Jackson Heart Study	outcome (included in risk factors review)	10.1016/j.gheart.2019.04.007
Ford	2019	Social Integration and Quality of Social Relationships as Protective Factors for Inflammation in a Nationally Representative Sample of Black Women	outcome (included in risk factors review)	10.1007/s11524-018-00337-x

Forrester	2021	Accelerated aging: A marker for social factors resulting in cardiovascular events?	outcome	10.1016/j.ssmph.2021.100733
Franke	2010	Is job-related stress the link between cardiovascular disease and the law enforcement profession?	exposure	10.1097/JOM.0b013e3181dd086b
Freak-Poli	2021	Social isolation, social support and loneliness as predictors of cardiovascular disease incidence and mortality	outcome	10.1186/s12877-021-02602-2
Fuller	2019	Individual- and area-level socioeconomic inequalities in diabetes mellitus in Saskatchewan between 2007 and 2012: a cross-sectional analysis	outcome	10.9778/cmajo.20180042
Fuller	2018	ACE gene haplotypes and social networks: Using a biocultural framework to investigate blood pressure variation in African Americans	outcome (included in risk factors review)	10.1371/journal.pone.0204127
Gafarov	2005	[Risk of arterial hypertension and social support]	full text not available	10.3402/ijch.v72i0.21210
Gafarov	2005	[A study of the risk factors of stroke development in the framework of WHO program MONICA-psychosocial"]"	full text not available	-
Gafarov	2019	The risk of myocardial infarction, and social support among the population of 25-64 years in Russia/Siberia	language	10.15829/1560-4071-2019-6-34-41
Gafarov	2013	The influence of social support on risk of acute cardiovascular diseases in female population aged 25-64 in Russia	outcome	-

Gafarova	2019	Social support and stroke risk: An epidemiological study of a population aged 25–64 years in Russia/Siberia (the WHO MONICA-psychosocial program)	outcome	10.14412/2074-2711-2019-1-12-20
Gallagher	2014	Social identity influences stress appraisals and cardiovascular reactions to acute stress exposure	exposure	10.1111/bjhp.12056
Gallo	2012	Individual and neighborhood socioeconomic status and inflammation in Mexican American women: what is the role of obesity?	outcome (included in risk factors review)	10.1097/PSY.0b013e31824f5f6d
Galobardes	2003	Measuring the habitat as an indicator of socioeconomic position: methodology and its association with hypertension	exposure	10.1136/jech.57.4.248
Garg	2020	Associations of anger, vital exhaustion, anti-depressant use, and poor social ties with incident atrial fibrillation: The Atherosclerosis Risk in Communities Study	outcome	10.1177/2047487319897163
Gary-Webb	2020	Changes in perceptions of neighborhood environment and Cardiometabolic outcomes in two predominantly African American neighborhoods	outcome (included in risk factors review)	10.1186/s12889-019-8119-9
Gaston	2023	Racial and Ethnic Discrimination and Hypertension by Educational Attainment Among a Cohort of US Women	exposure	10.1001/jamanetworkopen.2023.44707
Gebreab	2015	Geographic variations in cardiovascular health in the United States: contributions of state- and individual-level factors	outcome (included in risk factors review)	10.1161/jaha.114.001673

Glover	2023	Social Networks and Cardiovascular Disease Events in the Jackson Heart Study	outcome	10.1161/jaha.123.030149
Goldman	2016	All in the family: The link between kin network bridging and cardiovascular risk among older adults	exposure	10.1016/j.socscimed.2016.07.035
González-Villoria	2018	Social vulnerability and its possible relation to the principal causes of morbidity and mortality in the Mexican state of Oaxaca	not a primary study	10.1186/s12939-018-0849-2
Grimaud	2011	Incidence of stroke and socioeconomic neighborhood characteristics: an ecological analysis of Dijon stroke registry	outcome	10.1161/strokeaha.110.596429
Grimaud	2013	Gender differences in the association between socioeconomic status and subclinical atherosclerosis	outcome (included in risk factors review)	10.1371/journal.pone.0080195
Gronewold	2020	Association of social relationships with incident cardiovascular events and all-cause mortality	outcome	10.1136/heartjnl-2019-316250
Gustafsson	2012	Do peer relations in adolescence influence health in adulthood? Peer problems in the school setting and the metabolic syndrome in middle-age	exposure	10.1371/journal.pone.0039385
Hall	2019	Work life, relationship, and policy determinants of health and well-being among Filipino domestic Workers in China: a qualitative study	exposure	10.1186/s12889-019-6552-4
Hammar	1998	Job strain, social support at work, and incidence of myocardial infarction	exposure	10.1136/oem.55.8.548

Han	2017	Social Activities, Incident Cardiovascular Disease, and Mortality	outcome	10.1177/0898264316635565
Hanigan	2017	Impact of scale of aggregation on associations of cardiovascular hospitalization and socio-economic disadvantage	failed contact	10.1371/journal.pone.0188161
Hanson	1988	Social anchorage and blood pressure in elderly men--a population study	outcome (included in risk factors review)	10.1097/00004872-198806000-00011
Harris	2008	Myocardial infarction and heart failure hospitalization rates in Maine, USA - variability along the urban-rural continuum	outcome	ISSN: 1445-6354
Hart	1997	People, places and coronary heart disease risk factors: a multilevel analysis of the Scottish Heart Health Study archive	exposure	10.1016/s0277-9536(96)00431-5
Hazlehurst	2018	Individual and Neighborhood Stressors, Air Pollution and Cardiovascular Disease	exposure	10.3390/ijerph15030472
Hedblad	1992	Influence of social support on cardiac event rate in men with ischaemic type ST segment depression during ambulatory 24-h long-term ECG recording. The prospective population study 'Men born in 1914', Malmö, Sweden	Patient population	10.1093/oxfordjournals.eurheartj.a060193
Heijmans	2017	Social network composition of vascular patients and its associations with health behavior and clinical risk factors	Patient population	10.1371/journal.pone.0185341
Helmert	1992	Social inequities in cardiovascular disease risk factors in East and West Germany	exposure	10.1016/0277-9536(92)90181-o

Helmert	1988	Social group and risk factors for coronary heart diseases: Results of a health survey in Western Germany	language	10.1007/BF02083579
Helminen	1995	Carotid atherosclerosis in middle-aged men. Relation to conjugal circumstances and social support	outcome (included in risk factors review)	10.1177/140349489502300306
Helminen	1997	Social network in relation to plasma fibrinogen	outcome (included in risk factors review)	10.1017/s0021932097001296
Helminen	1995	Validity assessment of a social support index	outcome (included in risk factors review)	10.1177/140349489502300112
Hempler	2016	Relationship between social network, social support and health behaviour in people with type 1 and type 2 diabetes: cross-sectional studies	Patient population	10.1186/s12889-016-2819-1
Henning	2014	Identification of direct and indirect social network effects in the pathophysiology of insulin resistance in obese human subjects	outcome (included in risk factors review)	10.1371/journal.pone.0093860
Hernandez	2004	Relationship between psychosocial aspects of work environment and cardiovascular heart risk in men	full text not available	10.1080/13557858.2017.1294660
Hernandez	2018	Structural social support and cardiovascular disease risk factors in Hispanic/Latino adults with diabetes: results from the Hispanic Community Health Study/Study of Latinos (HCHS/SOL)	Patient population	-
Heyden	2023	Neighborhood deprivation and household socioeconomic status as risk factors for diabetes in Brussels	full text not available	

Hickson	2011	Socioeconomic position is positively associated with blood pressure dipping among African-American adults: the Jackson Heart Study	outcome (included in risk factors review)	10.1038/ajh.2011.98
Hipp	2015	Spatial analysis and correlates of county-level diabetes prevalence, 2009-2010	outcome	10.5888/pcd12.140404
Holmes	2012	Neighborhoods and systemic inflammation: High CRP among legal and unauthorized Brazilian migrants	outcome (included in risk factors review)	10.1016/j.healthplace.2011.11.006
Holt-Lunstad	2003	Social relationships and ambulatory blood pressure: structural and qualitative predictors of cardiovascular function during everyday social interactions	exposure	10.1037/0278-6133.22.4.388
Hong	2020	Development and Validation of a County-Level Social Determinants of Health Risk Assessment Tool for Cardiovascular Disease	outcome	10.1370/afm.2534
Hughes	2000	Quality and quantity of social support as differential predictors of cardiovascular reactivity	exposure	10.1080/03033910.2000.10558237
Hughes	2007	Social support in ordinary life and laboratory measures of cardiovascular reactivity: gender differences in habituation-sensitization	exposure	10.1007/bf02872671
Hussain	2021	Psychosocial stressors predict lower cardiovascular disease risk among Mexican-American adults living in a high-risk community: Findings from the Texas City Stress and Health Study	outcome	10.1371/journal.pone.0257940

Hussein	2018	Unequal Exposure or Unequal Vulnerability? Contributions of Neighborhood Conditions and Cardiovascular Risk Factors to Socioeconomic Inequality in Incident Cardiovascular Disease in the Multi-Ethnic Study of Atherosclerosis	outcome	10.1093/aje/kwx363
Islam	2020	Neighborhood Characteristics and Ideal Cardiovascular Health Among Black Adults: Results From the Morehouse-Emory Cardiovascular (MECA) Center for Health Equity	outcome (included in risk factors review)	10.1016/j.annepidem.2020.11.009
Jackson	1999	Effects of race, sex, and socioeconomic status upon cardiovascular stress responsivity and recovery in youth	patient population	10.1016/j.socscimed.2008.09.041
Jacobson	2020	Residential mobility and chronic disease among World Trade Center Health Registry enrollees, 2004-2016	failed contact	10.1016/j.healthplace.2019.102270
James	1994	Social Identity Correlates of Minority Workers Health	exposure	10.2307/256834
Jiang	2018	Neighborhood characteristics and lifestyle intervention outcomes: Results from the Special Diabetes Program for Indians	outcome	10.1016/j.ypmed.2018.03.009
Jimenez	2019	Longitudinal associations of neighborhood socioeconomic status with cardiovascular risk factors: A 46-year follow-up study	outcome (included in risk factors review)	10.1016/j.socscimed.2019.112574

Johnson	1988	Job strain, work place social support, and cardiovascular disease: a cross-sectional study of a random sample of the Swedish working population	exposure	10.2105/ajph.78.10.1336
Joo	2018	The Association between Social Network Betweenness and Coronary Calcium: A Baseline Study of Patients with a High Risk of Cardiovascular Disease	patient population	10.5551/jat.40469
Judd	2019	The Role of Neighborhood Deprivation in Stroke Risk in Two Countries: France and the United States	full text not available	-
Juonala	2016	Childhood Psychosocial Factors and Coronary Artery Calcification in Adulthood: The Cardiovascular Risk in Young Finns Study	exposure	10.5694/mja2.50285
Juonala	2019	The Australian Aboriginal Birth Cohort study: socio-economic status at birth and cardiovascular risk factors to 25 years of age	failed contact	10.1001/jamapediatrics.2015.4121
Kapuku	2002	Relationships among socioeconomic status, stress induced changes in cortisol, and blood pressure in African American males	failed contact	10.1207/s15324796abm2404_08
Kaufman	1999	Blood pressure change in Africa: case study from Nigeria	exposure	-
Kent de Grey	2019	Enemies and friends in high-tech places: the development and validation of the Online Social Experiences Measure	outcome (included in risk factors review)	10.1177/2055207619878351

Kershaw	2015	Neighborhood-level racial/ethnic residential segregation and incident cardiovascular disease: the multi-ethnic study of atherosclerosis	outcome	10.1093/aje/kwr116
Kershaw	2017	Association of Changes in Neighborhood-Level Racial Residential Segregation With Changes in Blood Pressure Among Black Adults: The CARDIA Study	outcome (included in risk factors review)	10.1001/jamainternmed.2017.1226
Kihal-Talantikite	2017	Developing a data-driven spatial approach to assessment of neighbourhood influences on the spatial distribution of myocardial infarction	exposure	10.1186/s12942-017-0094-8
Kim	2014	Perceived neighbourhood social cohesion and myocardial infarction	outcome	10.1136/jech-2014-204009
Kim	2013	Perceived neighborhood social cohesion and stroke	outcome	10.5888/pcd16.180505
Kim	2019	Identification of Resilient and At-Risk Neighborhoods for Cardiovascular Disease Among Black Residents: the Morehouse-Emory Cardiovascular (MECA) Center for Health Equity Study	outcome	10.1136/bmjopen-2017-018793
Kim	2020	Associations between social network properties and metabolic syndrome and the mediating effect of physical activity: findings from the Cardiovascular and Metabolic Diseases Etiology Research Center (CMERC) Cohort	outcome (included in risk factors review)	10.1136/bmjdrc-2020-001272
Kim	2010	Do neighborhood socioeconomic deprivation and low social cohesion predict coronary calcification?: the CARDIA study	outcome (included in risk factors review)	10.1093/aje/kwq098

King	2011	Neighborhood context and social disparities in cumulative biological risk factors	outcome (included in risk factors review)	10.1097/PSY.0b013e318227b062
Kjærulff	2016	Geographical clustering of incident acute myocardial infarction in Denmark: A spatial analysis approach	outcome	10.1136/bmjopen-2018-024207
Kjærulff	2019	Geographical inequalities in acute myocardial infarction beyond neighbourhood-level and individual-level sociodemographic characteristics: a Danish 10-year nationwide population-based cohort study	outcome	10.1016/j.sste.2016.05.001
Koopman	2012	Neighbourhood socioeconomic inequalities in incidence of acute myocardial infarction: a cohort study quantifying age- and gender-specific differences in relative and absolute terms	exposure	10.1186/1471-2458-12-617
Kop	2005	Social network and coronary artery calcification in asymptomatic individuals	exposure	10.1097/01.psy.0000161201.45643.8d
Korda	2016	Socioeconomic variation in incidence of primary and secondary major cardiovascular disease events: an Australian population-based prospective cohort study	outcome	10.1186/s12939-016-0471-0
Kort	2017	Relationship between neighborhood socioeconomic status and venous thromboembolism: results from a population-based study	outcome	10.1111/jth.13868

Kowitt	2018	How is neighborhood social disorganization associated with diabetes outcomes? A multilevel investigation of glycemic control and self-reported use of acute or emergency health care services	patient population	10.1186/s40842-018-0069-0
Lee	2018	Prevalence of Non-valvular Atrial Fibrillation Based on Geographical Distribution and Socioeconomic Status in the Entire Korean Population	exposure	10.1210/js.2018-00001
Lee	2015	Social support and networks: cardiovascular responses following recall on immigration stress among Chinese Americans	exposure	10.1016/j.jstrokecerebrovasdis.2017.10.011
Lee	2017	The Association between Social Network Betweenness and Coronary Calcification: A Baseline Study on Patients with a High Risk of Cardiovascular Disease	full text not available	10.1007/s10903-013-9955-9
Lee	2018	Using Indirect Measures to Identify Geographic Hot Spots of Poor Glycemic Control: Cross-sectional Comparisons With an A1C Registry	outcome	10.2337/dc18-0181
Lee	2017	Threats to security and ischaemic heart disease deaths: the case of homicides in Mexico	outcome	10.1016/j.atherosclerosis.2017.06.297
Lee	2020	Complex Role of Touch in Social Relationships for Older Adults' Cardiovascular Disease Risk	outcome (included in risk factors review)	10.1177/0164027520915793

Lei	2018	Biological embedding of neighborhood disadvantage and collective efficacy: Influences on chronic illness via accelerated cardiometabolic age	outcome (included in risk factors review)	10.1017/s0954579418000937
Leitão	2012	[Prevalence and factors associated with metabolic syndrome in users of primary healthcare units in São Paulo--SP, Brazil]	language	-
Lemelin	2009	Life-course socioeconomic positions and subclinical atherosclerosis in the multi-ethnic study of atherosclerosis	outcome (included in risk factors review)	10.1016/j.socscimed.2008.10.038
Lewis	2010	Race, psychosocial factors, and aortic pulse wave velocity: the Health, Aging, and Body Composition Study	outcome (included in risk factors review)	10.1093/gerona/glq089
Li	2019	Neighborhood Racial Diversity and Metabolic Syndrome: 2003-2008 National Health and Nutrition Examination Survey	outcome (included in risk factors review)	10.1007/s10903-018-0728-3
Liaw	2018	Living in Cold Spot" Communities Is Associated with Poor Health and Health Quality"	outcome	10.3122/jabfm.2018.03.170421
Liese	2018	Neighborhood characteristics, food deserts, rurality, and type 2 diabetes in youth: Findings from a case-control study	patient population	10.1016/j.healthplace.2018.01.004
Linden	1993	Sex differences in social support, self-deception, hostility, and ambulatory cardiovascular activity	outcome (included in risk factors review)	10.1037//0278-6133.12.5.376
Linder	2018	A Population-Based Approach to Mapping Vulnerability to Diabetes	exposure	10.3390/ijerph15102167

Lisabeth	2007	Neighborhood environment and risk of ischemic stroke: the brain attack surveillance in Corpus Christi (BASIC) Project	outcome	10.1093/aje/kwk005
Livingood	2010	Using multiple sources of data to assess the prevalence of diabetes at the subcounty level, Duval County, Florida, 2007	exposure	-
Loose	2017	Blood pressure and psychological distress among North Africans in France: The role of perceived personal/group discrimination and gender	outcome (included in risk factors review)	10.1002/ajhb.23026
Lotfata	2023	Using geographical random forest models to explore spatial patterns in the neighborhood determinants of hypertension prevalence across chicago, illinois, USA	exposure	10.1177/23998083231153401
Loucks	2006	Relation of social integration to inflammatory marker concentrations in men and women 70 to 79 years	outcome (included in risk factors review)	10.1016/j.amjcard.2005.10.043
Loucks	2005	Social integration is associated with fibrinogen concentration in elderly men	outcome (included in risk factors review)	10.1097/01.psy.0000160482.89163.e8
Lovasi	2008	Evaluating options for measurement of neighborhood socioeconomic context: evidence from a myocardial infarction case-control study	failed contact	10.1016/j.healthplace.2007.09.004
Lund	2012	Do Demands and Worries from Close Social Relations Increase the Risk of Subsequent Incident Ihd Hospitalization? A 7 Year Longitudinal Study of Middle-Aged Danish Men and Women	full text not available	10.1136/jech.2009.106153

Ma	2016	Geographical and socioeconomic disparity analysis - An empirical study of hypertension and its comorbidities in China	full text not available	10.1109/HICSS.2016.414
Mackie	2011	Ischaemic manifestations in giant cell arteritis are associated with area level socio-economic deprivation, but not cardiovascular risk factors	patient population	10.1093/rheumatology/ker265
Maheswaran	2004	Socio-economic deprivation and excess winter mortality and emergency hospital admissions in the South Yorkshire Coalfields Health Action Zone, UK	outcome	10.1016/j.puhe.2003.09.004
Maki	2020	Social support, strain, and glycemic control: A path analysis	outcome (included in risk factors review)	10.1111/pere.12333
Martin	2019	Neighborhood disadvantage across the transition from adolescence to adulthood and risk of metabolic syndrome	outcome (included in risk factors review)	10.1016/j.healthplace.2019.03.002
Martins	2008	[Hypertension in impoverished social segments in the state of São Paulo]	language	10.1590/s1413-81232008000200023
Matricciani	2013	Investigating individual- and area-level socioeconomic gradients of pulse pressure among normotensive and hypertensive participants	outcome (included in risk factors review)	10.3390/ijerph10020571
Mayne	2018	Longitudinal Associations of Neighborhood Crime and Perceived Safety With Blood Pressure: The Multi-Ethnic Study of Atherosclerosis (MESA)	outcome (included in risk factors review)	10.1093/ajh/hpy066
Mayne	2019	Neighbourhood racial/ethnic residential segregation and cardiometabolic risk: the multiethnic study of atherosclerosis	outcome (included in risk factors review)	10.1136/jech-2018-211159

McCrory	2016	Social Disadvantage and Social Isolation Are Associated With a Higher Resting Heart Rate: Evidence From The Irish Longitudinal Study on Ageing	exposure	10.1093/geronb/gbu163
McCurley	2019	Association of Social Adversity with Comorbid Diabetes and Depression Symptoms in the Hispanic Community Health Study/Study of Latinos Sociocultural Ancillary Study: A Syndemic Framework	outcome	10.1093/abm/kaz009
McKenzie	2020	Ideal cardiovascular health in urban Jamaica: prevalence estimates and relationship to community property value, household assets and educational attainment: a cross-sectional study	outcome (included in risk factors review)	10.1136/bmjopen-2020-040664
McNamara	2017	The contribution of housing and neighbourhood conditions to educational inequalities in non-communicable diseases in Europe: findings from the European Social Survey (2014) special module on the social determinants of health	exposure	10.1093/eurpub/ckw224
Mellman	2015	Blood Pressure Dipping and Urban Stressors in Young Adult African Americans	outcome (included in risk factors review)	10.1007/s12160-014-9684-x
Mendez-Luck	2015	Community as a source of health in three racial/ethnic communities in Oregon: a qualitative study	outcome	10.1186/s12889-015-1462-6

Mensah	2022	Perceptions of community members on contextual factors driving cardiovascular disease behavioural risk in Ghana: a qualitative study	outcome	10.1186/s12889-022-13646-3
Merkin	2009	Neighborhoods and cumulative biological risk profiles by race/ethnicity in a national sample of U.S. adults: NHANES III	outcome (included in risk factors review)	10.1016/j.annepidem.2008.12.006
Merkin	2020	Race/ethnicity, neighborhood socioeconomic status and cardio-metabolic risk	outcome (included in risk factors review)	10.1016/j.ssmph.2020.100634
Merlo	2013	Revisiting causal neighborhood effects on individual ischemic heart disease risk: a quasi-experimental multilevel analysis among Swedish siblings	outcome	10.1016/j.socscimed.2012.08.034
Merlo	2001	Diastolic blood pressure and area of residence: multilevel versus ecological analysis of social inequity	outcome (included in risk factors review)	10.1136/jech.55.11.791
Meza	2020	Social network factors and cardiovascular health among baltimore public housing residents	outcome (included in risk factors review)	10.1016/j.pmedr.2020.101192
Mezuk	2013	Depression, neighborhood deprivation and risk of type 2 diabetes	full text not available	10.1016/j.healthplace.2013.05.004
Mitaishvili	2006	Personality type and coronary heart disease	full text not available	-
Mobley	2006	Environment, obesity, and cardiovascular disease risk in low-income women	outcome (included in risk factors review)	10.1016/j.amepre.2005.12.001
Moissl	2020	Area-based socioeconomic status and mortality: the Ludwigshafen Risk and Cardiovascular Health study	patient population	10.1007/s00392-019-01494-y

Møller	1991	Social class and cardiovascular risk factors in Danish men	exposure	10.1177/140349489101900207
Møller	1991	[Social status and cardiovascular risk factors in Danish males]	language	-
Moon	2023	Mediation of an association between neighborhood socioeconomic environment and type 2 diabetes through the leisure-time physical activity environment in an analysis of three independent samples	design	10.1136/bmjdrc-2022-003120
Moradi	2016	Is the status of diabetes socioeconomic inequality changing in Kurdistan province, west of Iran? A comparison of two surveys	exposure	PMCID: PMC4972065; PMID: 27493919
Morovatdar	2019	Socioeconomic Status and Long-Term Stroke Mortality, Recurrence and Disability in Iran: The Mashhad Stroke Incidence Study	outcome	10.1159/000494885
Morrissey	2016	A multinomial model for comorbidity in England of long-standing cardiovascular disease, diabetes and obesity	outcome	10.1111/hsc.12251
Morse	2015	Neighborhood-level racial/ethnic residential segregation and incident cardiovascular disease: The multi-ethnic study of atherosclerosis Kershaw KN, Osypuk TL, Do DP, et al. Circulation 2015;131:141-8	full text not available	10.1016/j.jemermed.2015.05.020

Mueller	2012	The influence of neighbourhood deprivation on the prevalence of diabetes in 25- to 74-year-old individuals: first results from the Dortmund Health Study	full text not available	10.1111/j.1464-5491.2011.03526.x
Müller	2013	[Neighbourhood deprivation and type 2 diabetes: results from the Dortmund Health Study (DHS)]	language	10.1055/s-0033-1333737
Murakami	2010	Neighborhood socioeconomic status in relation to dietary intake and insulin resistance syndrome in female Japanese dietetic students	outcome (included in risk factors review)	10.1016/j.nut.2009.08.025
Nagayoshi	2014	Social network, social support, and risk of incident stroke: Atherosclerosis Risk in Communities study	outcome	10.1161/strokeaha.114.005815
Naimi	2009	Associations between area-level unemployment, body mass index, and risk factors for cardiovascular disease in an urban area	outcome (included in risk factors review)	10.3390/ijerph6123082
Nazzal	2018	Educational Inequalities in Cardiovascular Risk Factor and Blood Pressure Control in the Elderly: Comparison of MESA Cohort and Chilean NHS Survey Outcome Measures	exposure	10.1016/j.gheart.2017.09.001
Ndejjo	2023	Drivers of cardiovascular disease risk factors in slums in Kampala, Uganda: a qualitative study	outcome	10.1080/16549716.2022.2159126
Neergheen	2019	Neighborhood social cohesion is associated with lower levels of interleukin-6 in African American women	outcome (included in risk factors review)	10.1016/j.bbi.2018.10.008

Neudorf	2015	An analytic approach for describing and prioritizing health inequalities at the local level in Canada: a descriptive study	failed contact	10.9778/cmajo.20150049
Ng	2012	Trends of blood pressure levels and management in Västerbotten County, Sweden, during 1990-2010	exposure	10.3402/gha.v5i0.18195
Ngo	2013	Area-level socioeconomic characteristics and incidence of metabolic syndrome: a prospective cohort study	outcome (included in risk factors review)	10.1186/1471-2458-13-681
Ngo	2014	Area-level socioeconomic characteristics, prevalence and trajectories of cardiometabolic risk	outcome (included in risk factors review)	10.3390/ijerph110100830
Nguyen	2021	Google Street View-Derived Neighborhood Characteristics in California Associated with Coronary Heart Disease, Hypertension, Diabetes	exposure	10.3390/ijerph181910428
Nichols	2018	Socioeconomic Disadvantage Is Associated with a Higher Incidence of Aneurysmal Subarachnoid Hemorrhage	outcome	10.1016/j.jstrokecerebrovasdis.2017.09.055
Niedhammer	1998	Psychosocial work environment and cardiovascular risk factors in an occupational cohort in France	exposure	10.1136/jech.52.2.93
Nishi	2018	Social Network Structure and Atherosclerotic Cardiovascular Disease	not a primary study	10.5551/jat.ED087
Non	2014	Childhood social disadvantage, cardiometabolic risk, and chronic disease in adulthood	exposure	10.1093/aje/kwu127

Nordstrom	2002	Neighborhood and individual socioeconomic status and early atherosclerosis: The Los Angeles atherosclerosis study	full text not available	-
Nordstrom	2004	The association of personal and neighborhood socioeconomic indicators with subclinical cardiovascular disease in an elderly cohort. The cardiovascular health study	outcome (included in risk factors review)	10.1016/j.socscimed.2004.03.017
O'Malley	2011	Longitudinal analysis of large social networks: estimating the effect of health traits on changes in friendship ties	outcome	10.1002/sim.4190
O'Neil	2020	Inequalities and Deteriorations in Cardiovascular Health in Premenopausal US Women, 1990-2016	not a primary study	10.2105/ajph.2020.305702
O'Reilly	1989	Role of support networks in maintenance of improved cardiovascular health status	outcome	10.1016/0277-9536(89)90268-2
Orth-Gomér	1998	Orth-Gomér	Patient population	10.1053/euhj.1998.1190
Palacio	2020	Social determinants of health score: does it help identify those at higher cardiovascular risk?	exposure	10.37765/ajmc.2020.88504
Park	2023	Examining the Relationship Between Multilevel Resilience Resources and Cardiovascular Disease Incidence, Overall and by Psychosocial Risks, Among Participants in the Jackson Heart Study, the Multi-Ethnic Study of Atherosclerosis, and the Mediators of Atherosclerosis in South Asians Living in America (MASALA) Study	outcome	10.1093/aje/kwad159

Pedersen	2016	Psychosocial risk factors for the metabolic syndrome: A prospective cohort study	outcome (included in risk factors review)	10.1016/j.ijcard.2016.04.076
Pengpid	2022	Prevalence and determinants of stroke among older adults in India: Results of a national community-dwelling survey in 2017-2018	full text not available	
Petrelli	2006	Socioeconomic inequalities in coronary heart disease in Italy: a multilevel population-based study	outcome	10.1016/j.socscimed.2006.01.018
Pham-Kanter	2009	Social comparisons and health: can having richer friends and neighbors make you sick?	exposure	10.1016/j.socscimed.2009.05.017
Picciotto	2006	Associations of area based deprivation status and individual educational attainment with incidence, treatment, and prognosis of first coronary event in Rome, Italy	outcome	10.1136/jech.2005.037846
Piferi	2006	Social support and ambulatory blood pressure: an examination of both receiving and giving	outcome (included in risk factors review)	10.1016/j.ijpsycho.2006.06.002
Pollack	2012	Neighborhood socioeconomic status and coronary heart disease risk prediction in a nationally representative sample	outcome (included in risk factors review)	10.1016/j.puhe.2012.05.028
Pollard	2003	Social networks and coronary heart disease risk factors in South Asians and Europeans in the UK	outcome (included in risk factors review)	10.1080/1355785032000136452
Pollitt	2008	Cumulative life course and adult socioeconomic status and markers of inflammation in adulthood	outcome (included in risk factors review)	10.1136/jech.2006.054106

Pollitt	2007	Early-life and adult socioeconomic status and inflammatory risk markers in adulthood	outcome (included in risk factors review)	10.1007/s10654-006-9082-1
Polyakova	2019	Local Area Variation in Morbidity Among Low-Income, Older Adults in the United States: A Cross-sectional Study	outcome	10.7326/m18-2800
Pujades-Rodriguez	2014	Socioeconomic deprivation and the incidence of 12 cardiovascular diseases in 1.9 million women and men: implications for risk prediction and prevention	outcome	10.1371/journal.pone.0104671
Pulkki-Råback	2017	Positive Psychosocial Factors in Childhood Predicting Lower Risk for Adult Type 2 Diabetes: The Cardiovascular Risk in Young Finns Study, 1980-2012	exposure	10.1016/j.amepre.2017.01.042
Pylypchuk	2018	Cardiovascular disease risk prediction equations in 400 000 primary care patients in New Zealand: a derivation and validation study	outcome	10.1016/s0140-6736(18)30664-0
Raghavan	2016	Incident Type 2 Diabetes Risk is Influenced by Obesity and Diabetes in Social Contacts: a Social Network Analysis	exposure	10.1007/s11606-016-3723-1
Ramkumar	2019	Association between socioeconomic status and incident atrial fibrillation	outcome	10.1111/imj.14214
Ramsay	2017	Individual and Neighbourhood-Level Socioeconomic Factors and Incidence of Type 2 Diabetes in Older Age: Results from a 14 Year Follow-up of a Cohort of Older British Men	full text not available	10.1136/jech-2017-SSMAbstracts.78

Reblin	2010	Provider and recipient factors that may moderate the effectiveness of received support: examining the effects of relationship quality and expectations for support on behavioral and cardiovascular reactions	exposure	10.1007/s10865-010-9270-z
Reddy	2022	Exposure to Neighborhood-Level Racial Residential Segregation in Young Adulthood to Midlife and Incident Subclinical Atherosclerosis in Black Adults: The Coronary Artery Risk Development in Young Adults Study	outcome	10.1161/circoutcomes.121.007986
Reed	1984	Psychosocial processes and general susceptibility to chronic disease	outcome	10.1093/oxfordjournals.aje.a113754
Reed	1983	Social networks and coronary heart disease among Japanese men in Hawaii	outcome	10.1093/oxfordjournals.aje.a113557
Reshetnyak	2020	Impact of Multiple Social Determinants of Health on Incident Stroke	outcome	10.1161/strokeaha.120.028530
Ribeiro	2019	Neighbourhood socioeconomic deprivation and allostatic load: a multi-cohort study	outcome (included in risk factors review)	10.1038/s41598-019-45432-4
Riva	2016	Association between individual-level and community-level socio-economic status and blood pressure among Inuit in Greenland	outcome (included in risk factors review)	10.3402/ijch.v75.32757
Rivera	2015	The influence of socioeconomic status on future risk for developing Type 2 diabetes in the Canadian population between 2011 and 2022: differential associations by sex	design	10.1186/s12939-015-0245-0

Robinette	2018	Perceived neighborhood social cohesion and cardiometabolic risk: a gene × environment study	duplicate	10.1080/19485565.2019.1579084
Robinette	2020	Perceived neighborhood social cohesion and cardiometabolic risk: a gene Ã— environment study	outcome (included in risk factors review)	10.1080/19485565.2019.1568672
Rodrigues	2019	Sociodemographic disparities in hypertension prevalence: Results from the first Portuguese National Health Examination Survey	exposure	10.1016/j.repc.2018.10.012
Rosenblatt	2021	Association between neighborhood social cohesion, awareness of chronic diseases, and participation in healthy behaviors in a community cohort	outcome	10.1186/s12889-021-11633-8
Rosengren	2004	Coronary disease in relation to social support and social class in Swedish men. A 15 year follow-up in the study of men born in 1933	outcome	10.1016/j.ehj.2003.10.005
Rosvall	2007	Area social characteristics and carotid atherosclerosis	outcome (included in risk factors review)	10.1093/eurpub/ckl239
Roth	2005	The community need index. A new tool pinpoints health care disparities in communities throughout the nation	full text not available	-
Rudolf	2021	Socioeconomic factors and the onset of peripheral artery disease in older adults	full text not available	10.1024/0301-1526/a000961
Rutledge	2008	Social networks and incident stroke among women with suspected myocardial ischemia	patient population	10.1097/PSY.0b013e3181656e09

Rutledge	2004	Social networks are associated with lower mortality rates among women with suspected coronary disease: the National Heart, Lung, and Blood Institute-Sponsored Women's Ischemia Syndrome Evaluation study	patient population	10.1097/01.psy.0000145819.94041.52
Sabzmakan	2014	Environmental determinants of cardiovascular diseases risk factors: a qualitative directed content analysis	outcome	10.5812/ircmj.11573
Saidi	2019	Explaining income-related inequalities in cardiovascular risk factors in Tunisian adults during the last decade: comparison of sensitivity analysis of logistic regression and Wagstaff decomposition analysis	exposure	10.1186/s12939-019-1047-6
Sajjad	2019	Association between area-level socioeconomic status, accessibility and diabetes-related hospitalisations: a cross-sectional analysis of data from Western Victoria, Australia	patient population	10.1136/bmjopen-2018-026880
Seeman	2014	Social relationships and their biological correlates: Coronary Artery Risk Development in Young Adults (CARDIA) study	outcome (included in risk factors review)	10.1016/j.psyneuen.2014.02.008
Shah	2021	Association of Social Network Characteristics With Cardiovascular Health and Coronary Artery Calcium in South Asian Adults in the United States: The MASALA Cohort Study	outcome	10.1161/jaha.120.019821

Shahu	2019	Disparities in Socioeconomic Context and Association With Blood Pressure Control and Cardiovascular Outcomes in ALLHAT	patient population	10.1161/jaha.119.012277
Shakoor-Abdullah	1997	Incorporating socio-economic and risk factor diversity into the development of an African-American community blood pressure control program	full text not available	-
Sheehy	2023	Perceived Interpersonal Racism and Incident Stroke Among US Black Women	outcome	10.1001/jamanetworkopen.2023.43203
Shirey	2021	Relation of Neighborhood Disadvantage to Heart Failure Symptoms and Hospitalizations	patient population	10.1016/j.amjcard.2020.10.057
Shishehbor	2006	Association of socioeconomic status with functional capacity, heart rate recovery, and all-cause mortality	patient population	10.1001/jama.295.7.784
Siegel	2023	The relationship between state-level structural racism and disparities between the non-hispanic black and non-hispanic white populations in multiple health outcomes	outcome	10.1016/j.jnma.2023.01.010
Silventoinen	2022	Joint associations of depression, genetic susceptibility and the area of residence for coronary heart disease incidence	exposure	10.1136/jech-2021-216451
Singh	2017	Social Determinants of Health in the United States: Addressing Major Health Inequality Trends for the Nation, 1935-2016	exposure	10.21106/ijma.236
Slopen	2014	Childhood adversity, adult neighborhood context, and cumulative biological risk for chronic diseases in adulthood	exposure	10.1097/psy.0000000000000081

Slopen	2015	Early origins of inflammation: An examination of prenatal and childhood social adversity in a prospective cohort study	exposure	10.1016/j.psyneuen.2014.10.016
Smith	2012	The psychosocial work environment and incident diabetes in Ontario, Canada	outcome	10.1093/occmed/kqs128
Sorman	2016	Blood pressure levels and longitudinal changes in relation to social network factors	outcome (included in risk factors review)	URL: https://hrcak.srce.hr/156333
Sprung	2019	Neighborhood crime is differentially associated with cardiovascular risk factors as a function of race and sex	outcome (included in risk factors review)	10.4081/jphr.2019.1643
Starr	2000	Seven-year follow-up of blood pressure in the Healthy Old People in Edinburgh (HOPE) cohort	exposure	10.1038/sj.jhh.1001039
Steppuhn	2019	Individual and area-level determinants associated with C-reactive protein as a marker of cardiometabolic risk among adults: Results from the German National Health Interview and Examination Survey 2008-2011	outcome (included in risk factors review)	10.1371/journal.pone.0211774
Stewart	2011	Diabetes and the socioeconomic and built environment: geovisualization of disease prevalence and potential contextual associations using ring maps	outcome	10.1186/1476-072x-10-18
Stjärne	2006	Neighborhood socioeconomic context, individual income and myocardial infarction	outcome	10.1097/01.ede.0000187178.51024.a7
Strogatz	1997	Social support, stress, and blood pressure in black adults	outcome (included in risk factors review)	10.1097/00001648-199709000-00002

Sun	2023	Social Determinants, Cardiovascular Disease, and Health Care Cost: A Nationwide Study in the United States Using Machine Learning	design	10.1161/jaha.122.027919
Sundquist	2006	Low linking social capital as a predictor of coronary heart disease in Sweden: a cohort study of 2.8 million people	outcome	10.1016/j.socscimed.2005.06.049
Sundquist	2006	Neighborhood violent crime and unemployment increase the risk of coronary heart disease: a multilevel study in an urban setting	outcome	10.1093/aje/kwh096
Sundquist	2004	Neighbourhood deprivation and incidence of coronary heart disease: a multilevel study of 2.6 million women and men in Sweden	outcome	10.1016/j.socscimed.2005.08.051
Szőcs	2019	Socioeconomic gap between neighborhoods of Budapest: Striking impact on stroke and possible explanations	exposure	10.1371/journal.pone.0212519
Tabb	2022	Spatially varying racial inequities in cardiovascular health and the contribution of individual- and neighborhood-level characteristics across the United States: The REasons for geographic and racial differences in stroke (REGARDS) study	outcome	10.1016/j.sste.2021.100473
Tawakol	2019	Stress-Associated Neurobiological Pathway Linking Socioeconomic Disparities to Cardiovascular Disease	outcome	10.1016/j.jacc.2019.04.042

Theorell	1982	Blood pressure variations across areas in the greater Stockholm region: analysis of 74,000 18-year-old men	outcome (included in risk factors review)	10.1016/0277-9536(82)90055-7
Thompson	2008	Reducing diabetes risk in American Indian women	exposure	10.1016/j.amepre.2007.11.014
Thorpe	2022	Neighborhood Socioeconomic Environment and Risk of Type 2 Diabetes: Associations and Mediation Through Food Environment Pathways in Three Independent Study Samples	design	10.2337/dc21-1693
Tideman	2013	A comparison of Australian rural and metropolitan cardiovascular risk and mortality: the Greater Green Triangle and North West Adelaide population surveys	outcome	10.1136/bmjopen-2013-003203
Toms	2020	Geographic variation in cardiometabolic risk factor prevalence explained by area-level disadvantage in the Illawarra-Shoalhaven region of the NSW, Australia	outcome (included in risk factors review)	10.1038/s41598-020-69552-4
Troxel	2010	Social integration, social contacts, and blood pressure dipping in African-Americans and whites	outcome (included in risk factors review)	10.1097/HJH.0b013e328333ab01
Tsiampalis	2023	The Spatial Variability of the Sex and Age- Specific 10-Year Incidence of Cardiovascular Diseases and its Clinical Risk Factors in Athens Metropolitan Area: a Local Statistical Modelling Approach in the Context of the ATTICA Epidemiological Study	outcome	10.1007/s12061-023-09508-z

Tydén	2001	Geographical pattern of female deaths from myocardial infarction in an urban population: Fatal outcome out-of-hospital related to socio-economic deprivation	outcome	10.1046/j.1365-2796.2001.00877.x
Uchino	2001	Heterogeneity in the social networks of young and older adults: prediction of mental health and cardiovascular reactivity during acute stress	exposure	10.1037/pag0000092
Uchino	2016	The quality of social networks predicts age-related changes in cardiovascular reactivity to stress	exposure	10.1023/a:1010634902498
Uchino	2013	The quality of spouses' social networks contributes to each other's cardiovascular risk	outcome (included in risk factors review)	10.1371/journal.pone.0071881
Undén	1991	Cardiovascular effects of social support in the work place: twenty-four-hour ECG monitoring of men and women	exposure	10.1097/00006842-199101000-00005
Unger	2014	Association of neighborhood characteristics with cardiovascular health in the multi-ethnic study of atherosclerosis	outcome (included in risk factors review)	10.1161/circoutcomes.113.000698
Valtorta	2018	Loneliness, social isolation and risk of cardiovascular disease in the English Longitudinal Study of Ageing	outcome	10.1177/2047487318792696
VanCauwenberg	2019	Population density is beneficially associated with 12-year diabetes risk marker change among residents of lower socio-economic neighborhoods	exposure	10.1016/j.healthplace.2019.02.006

Vastagh	2020	[The connection between the socioeconomic status and stroke in Budapest]	language	10.18071/isz.73.0389
Virtanen	2018	Unfavorable and favorable changes in modifiable risk factors and incidence of coronary heart disease: The Whitehall II cohort study	outcome	10.1016/j.ijcard.2018.07.005
Walker	2015	Classifying high-prevalence neighborhoods for cardiovascular disease in Texas	exposure	10.1016/j.apgeog.2014.11.011
Wang	2022	DNA Methylation Mediates the Association Between Individual and Neighborhood Social Disadvantage and Cardiovascular Risk Factors	outcome	10.3389/fcvm.2022.848768
Wang	2020	Association of the Robert Wood Johnson Foundations' social determinants of health and Medicare hospitalisations for ischaemic strokes: a cross-sectional data analysis	patient population	-
Wee	2011	The effect of neighborhood, socioeconomic status and a community-based program on multi-disease health screening in an Asian population: a controlled intervention study	outcome	10.1016/j.ypmed.2011.05.005
Weinehall	2001	Can a sustainable community intervention reduce the health gap?--10-year evaluation of a Swedish community intervention program for the prevention of cardiovascular disease	exposure	-

Wennerholm	2011	Cardiovascular disease occurrence in two close but different social environments	exposure	10.1186/1476-072X-10-5
Whittaker	2012	Combining psychosocial data to improve prediction of cardiovascular disease risk factors and events: The National Heart, Lung, and Blood Institute--sponsored Women's Ischemia Syndrome Evaluation study	outcome (included in risk factors review)	10.1097/PSY.0b013e31824a58ff
Willets	2019	Association Between Perceived Neighborhood Characteristics and Carotid Artery Intima-Media Thickness: Cross-Sectional Results From the ELSA-Brasil Study	outcome (included in risk factors review)	10.1016/j.gheart.2019.09.002
Williams	2012	Area-level socioeconomic status and incidence of abnormal glucose metabolism: the Australian Diabetes, Obesity and Lifestyle (AusDiab) study	outcome (included in risk factors review)	10.2337/dc11-1410
Wing	2016	Change in Neighborhood Characteristics and Change in Coronary Artery Calcium: A Longitudinal Investigation in the MESA (Multi-Ethnic Study of Atherosclerosis) Cohort	outcome (included in risk factors review)	10.1161/circulationaha.115.020534
Winkleby	2007	Inequities in CHD incidence and case fatality by neighborhood deprivation	outcome	10.1016/j.amepre.2006.10.002
Wood	2021	Should Strengthening Bonds Be a Public Health Priority? A Population-Based Analysis	full text not available	10.1027/2512-8442/a000074
Xie	2010	[Study on health-related behaviors among males in rural and urban residents from Guangdong province]	full text not available	-

Yan	2017	Cardiovascular Diseases and Risk-Factor Burden in Urban and Rural Communities in High-, Middle-, and Low-Income Regions of China: A Large Community-Based Epidemiological Study	outcome	10.1161/jaha.116.004445
Yang	2023	Association of Neighborhood Racial and Ethnic Composition and Historical Redlining With Built Environment Indicators Derived From Street View Images in the US	exposure	10.1001/jamanetworkopen.2022.51201
Yao	2019	Income distribution and health: can polarization explain health outcomes better than inequality?	outcome (included in risk factors review)	10.1007/s10198-018-1016-9
Yeager	2006	Religious involvement and health outcomes among older persons in Taiwan	exposure	10.1016/j.socscimed.2006.05.007
Yokobayashi	2017	Association between Social Relationship and Glycemic Control among Older Japanese: JAGES Cross-Sectional Study	patient population	10.1371/journal.pone.0169904
Zanelatto	2019	Perception of neighborhood disorder and blood pressure in adults: a multilevel population-based study	outcome (included in risk factors review)	10.1590/0102-311x00016418
Zghebi	2017	Examining trends in type 2 diabetes incidence, prevalence and mortality in the UK between 2004 and 2014	failed contact	10.1111/dom.12964
Zhang	2019	Type 2 diabetes mellitus and neighborhood deprivation index: A spatial analysis in Zhejiang, China	design	10.1111/jdi.12899

Zhao	2015	Using integrated visualization techniques to investigate associations between cardiovascular health outcomes and residential migration in Auckland, New Zealand	patient population	10.1080/15230406.2015.1013567
Zhou	2015	Geographical variation in diabetes prevalence and detection in china: multilevel spatial analysis of 98,058 adults	exposure	10.2337/dc14-1100
Zoller	2013	Neighbourhood deprivation and hospitalization for atrial fibrillation in Sweden	outcome (included in risk factors review)	10.1093/europace/eut019
Zöller	2012	Neighborhood deprivation and hospitalization for venous thromboembolism in Sweden	outcome	10.1007/s11239-012-0728-4
Zoughbie	2019	A social-network behavioral health program on sustained long-term body weight and glycemic outcomes: 2-year follow-up of a 4-month Microclinic Health Program in Jordan	patient population	10.1016/j.pmedr.2018.12.002
Zghebi	2017	Examining trends in type 2 diabetes incidence, prevalence and mortality in the UK between 2004 and 2014	failed contact	10.1111/dom.12964
Zhang	2019	Type 2 diabetes mellitus and neighborhood deprivation index: A spatial analysis in Zhejiang, China	design	10.1111/jdi.12899
Zhao	2015	Using integrated visualization techniques to investigate associations between cardiovascular health outcomes and residential migration in Auckland, New Zealand	patient population	10.1080/15230406.2015.1013567

Zhou	2015	Geographical variation in diabetes prevalence and detection in china: multilevel spatial analysis of 98,058 adults	exposure	10.2337/dc14-1100
Zoller	2013	Neighbourhood deprivation and hospitalization for atrial fibrillation in Sweden	outcome (included in risk factors review)	10.1093/europace/eut019
Zöller	2012	Neighborhood deprivation and hospitalization for venous thromboembolism in Sweden	outcome	10.1007/s11239-012-0728-4
Zoughbie	2019	A social-network behavioral health program on sustained long-term body weight and glycemic outcomes: 2-year follow-up of a 4-month Microclinic Health Program in Jordan	patient population	10.1016/j.pmedr.2018.12.002
