# Supplementary material for: Associations between dimensions of the social environment and cardiometabolic health outcomes: a systematic review and meta-analysis
Source: BMJ Open. 2024 Aug 28;14(8):e079987. doi: 10.1136/bmjopen-2023-079987 (PMC11367359; doi:10.1136/bmjopen-2023-079987)
Supplement: online supplemental file 4 [file bmjopen-14-8-s004.pdf]

Supplementary Figures 1A-1N Overall Meta-Analysis Figures

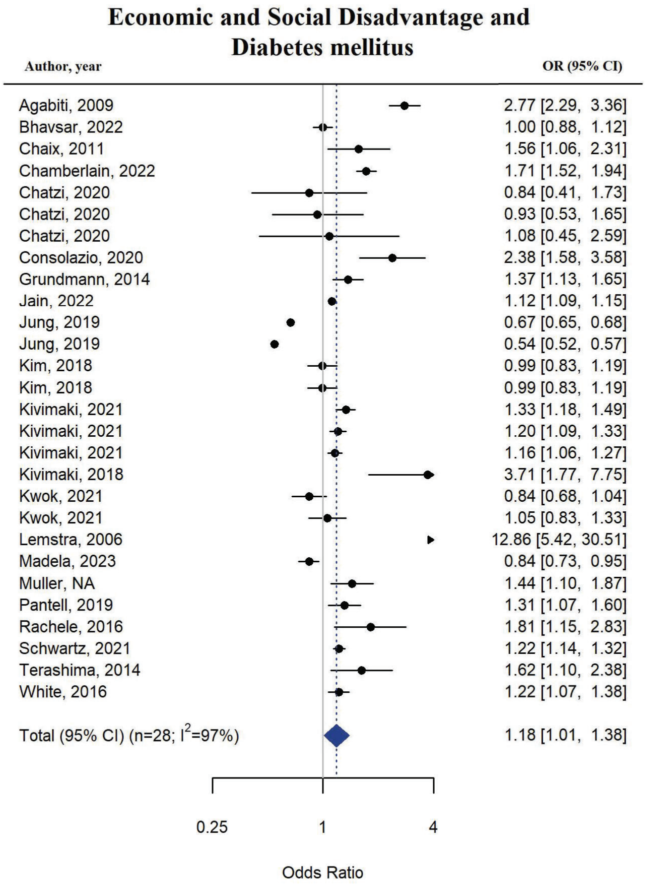

Supplementary Figure 1a

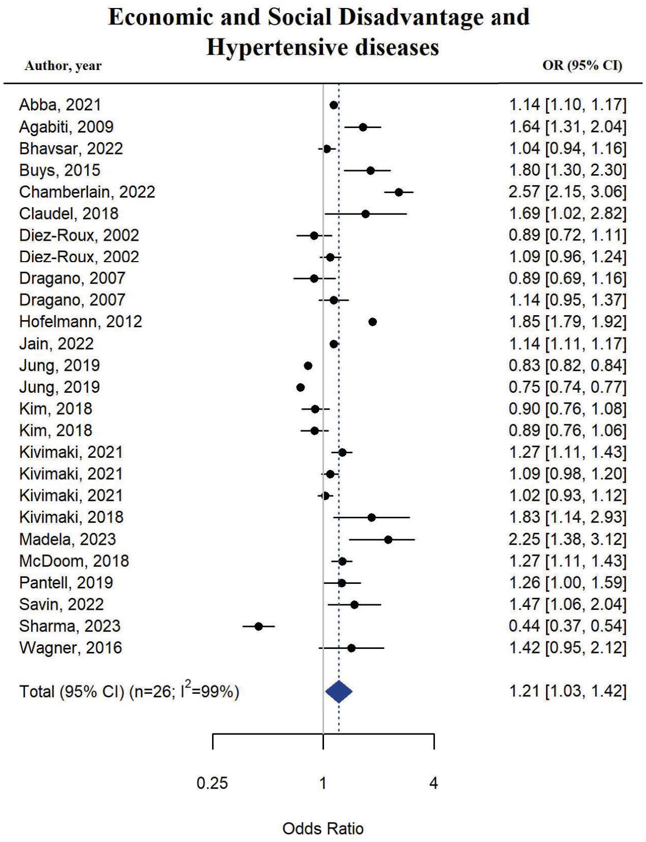

Supplementary Figure 1b

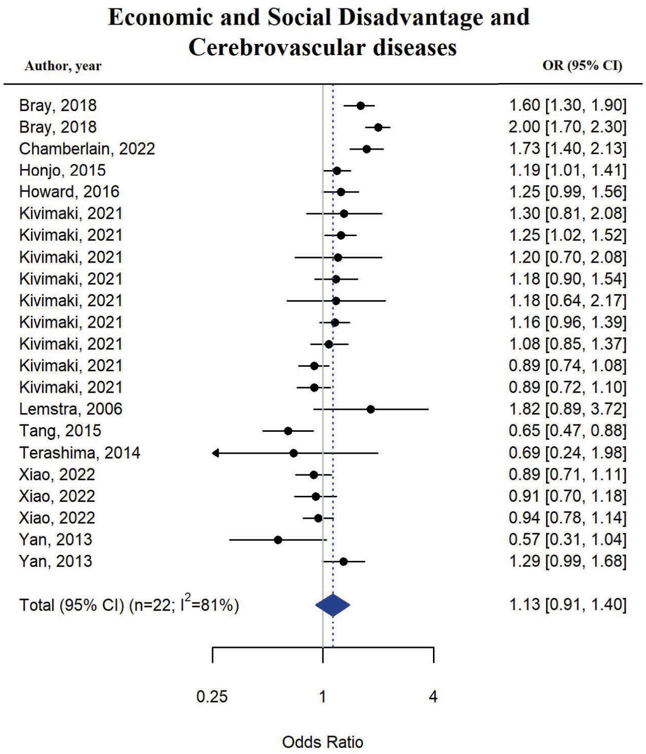

Supplementary Figure 1c

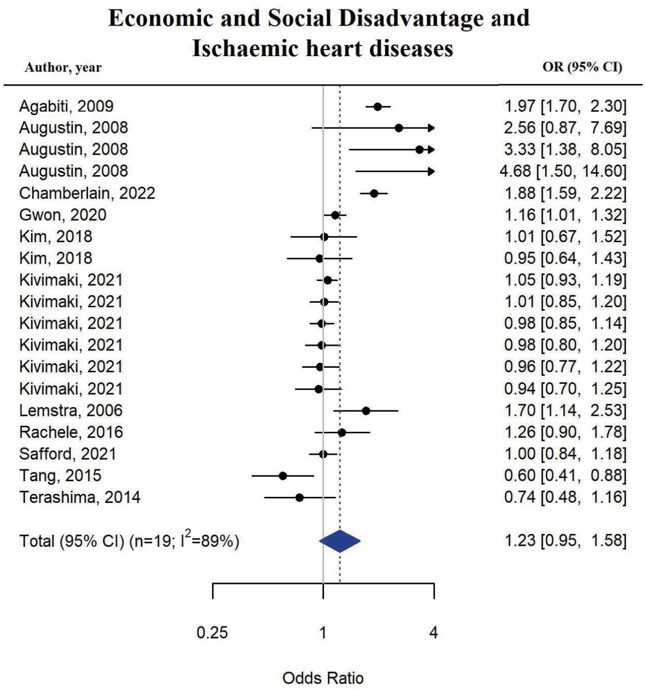

Supplementary Figure 1d

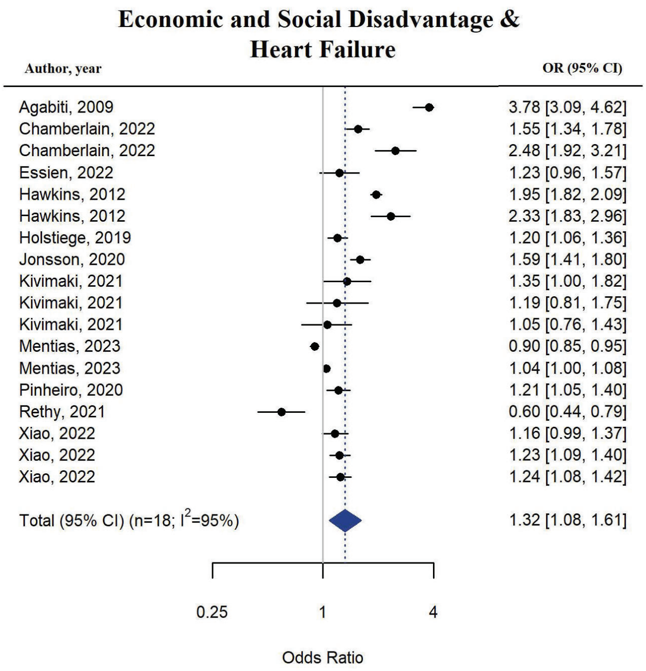

Supplementary Figure 1e

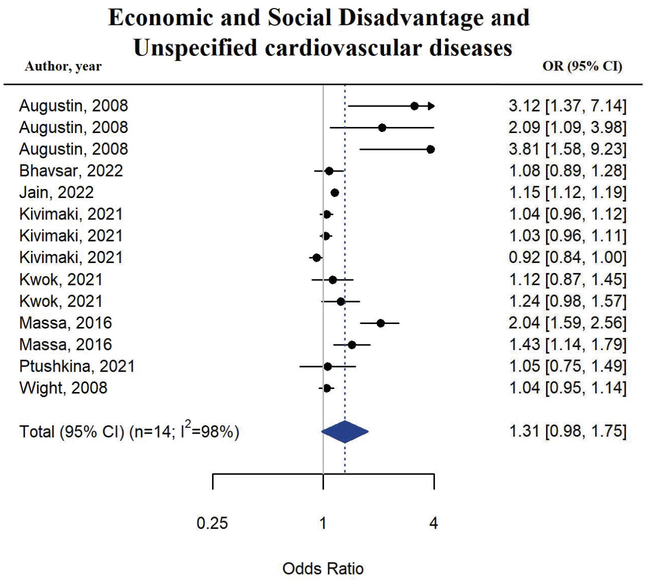

Supplementary Figure 1f

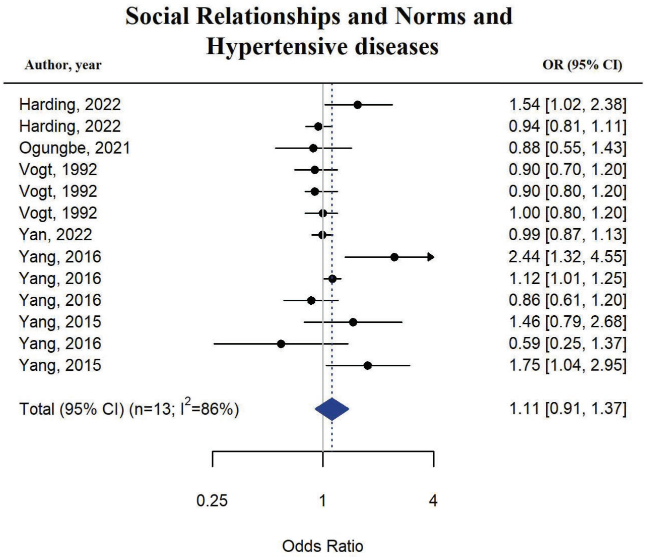

Supplementary Figure 1g

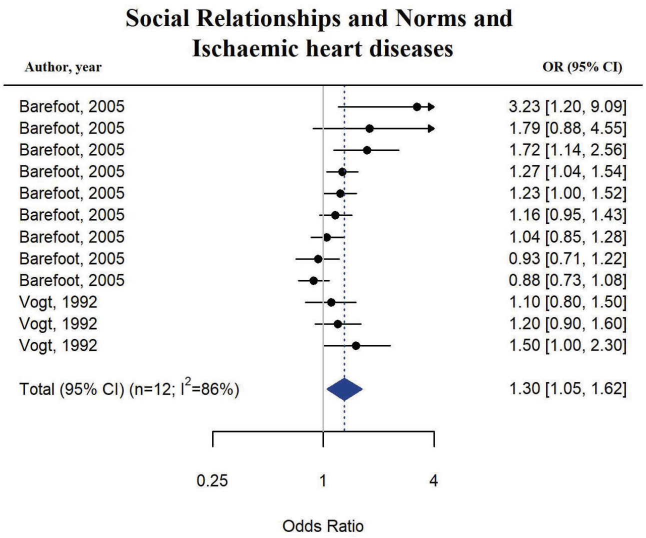

Supplementary Figure 1h

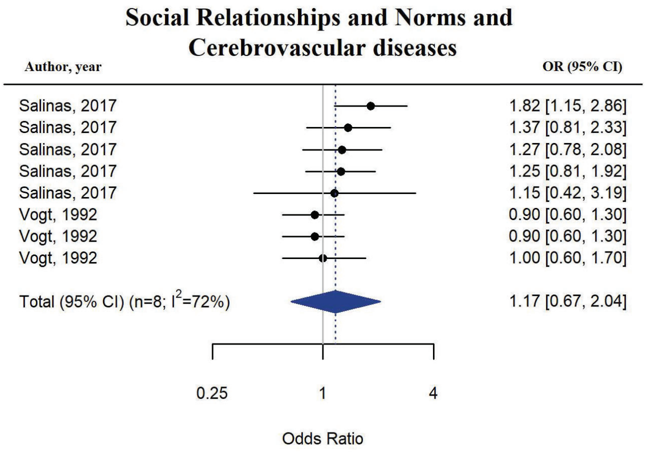

Supplementary Figure 1i

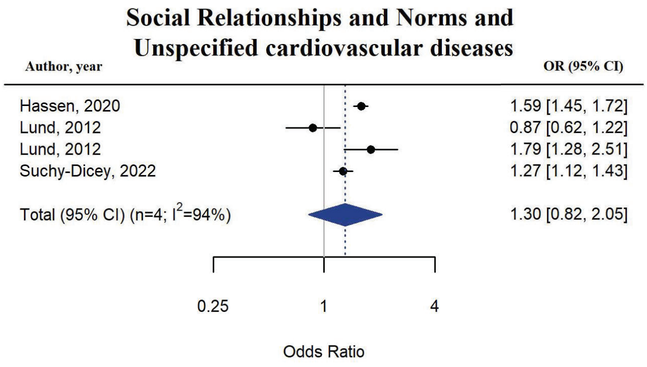

Supplementary Figure 1j

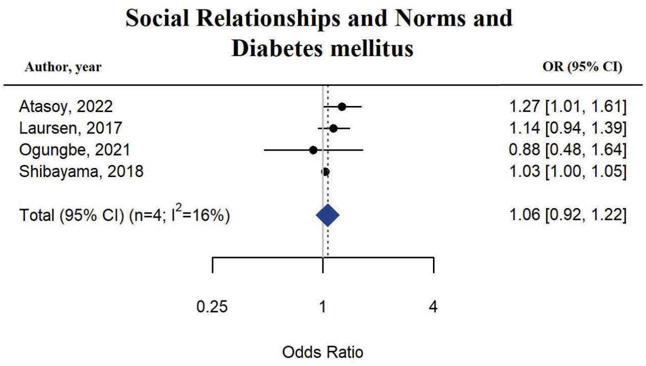

Supplementary Figure 1k

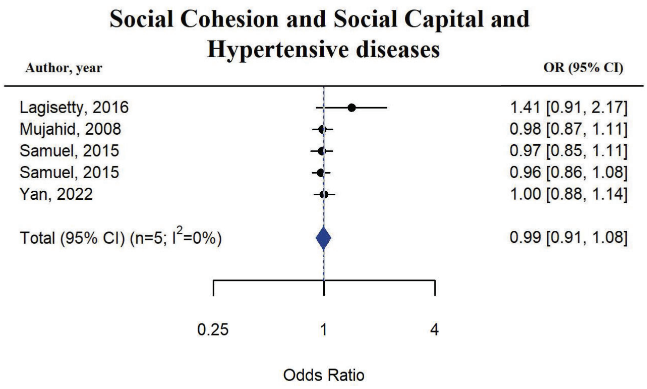

Supplementary Figure 1l

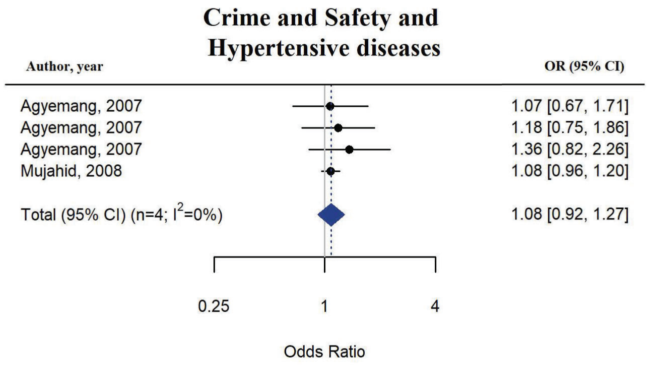

Supplementary Figure 1m

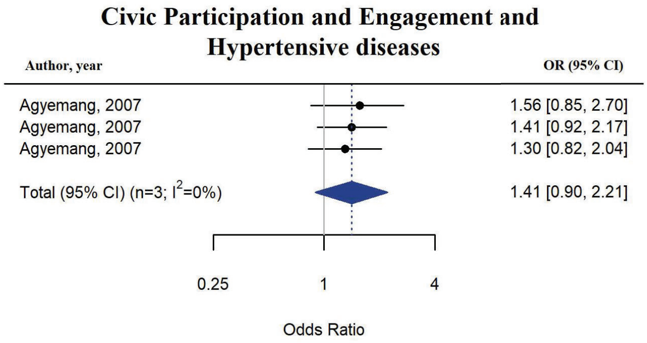

Supplementary Figure 1n
