# Supplementary material for: Associations between dimensions of the social environment and cardiometabolic health outcomes: a systematic review and meta-analysis
Source: BMJ Open. 2024 Aug 28;14(8):e079987. doi: 10.1136/bmjopen-2023-079987 (PMC11367359; doi:10.1136/bmjopen-2023-079987)
Supplement: online supplemental file 5 [file bmjopen-14-8-s005.pdf]

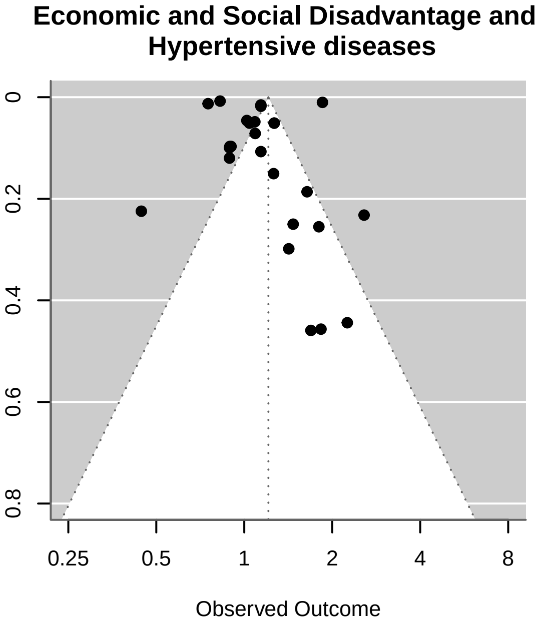

Supplementary Figure 2a

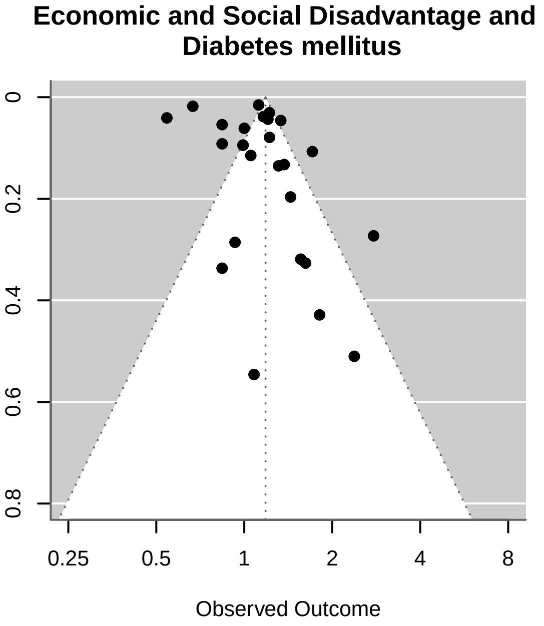

Supplementary Figure 2b

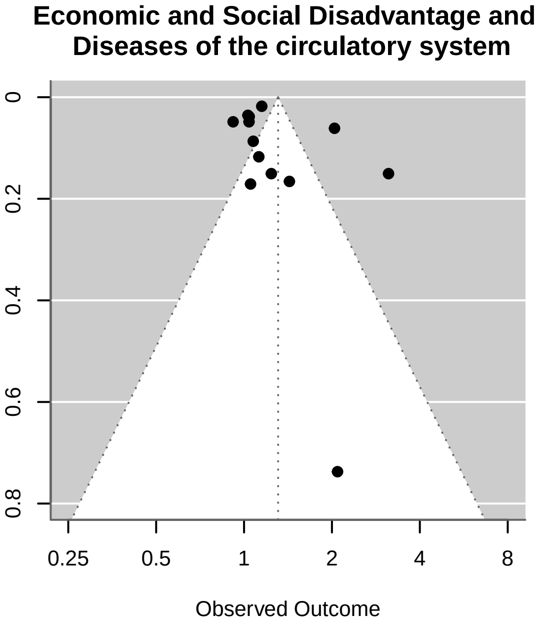

Supplementary Figure 2c

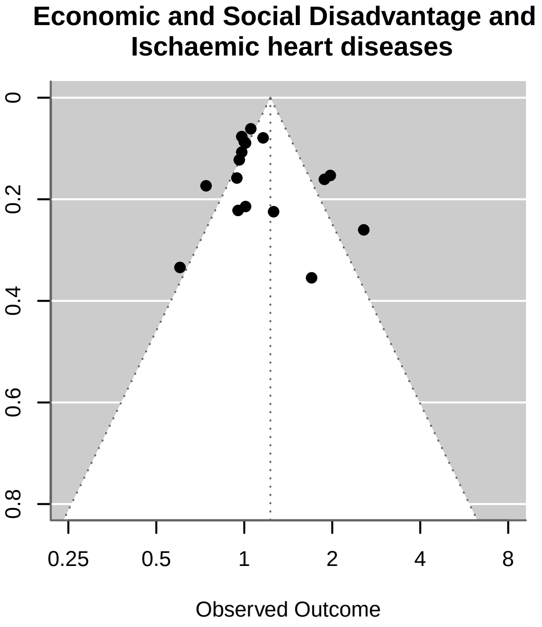

Supplementary Figure 2d

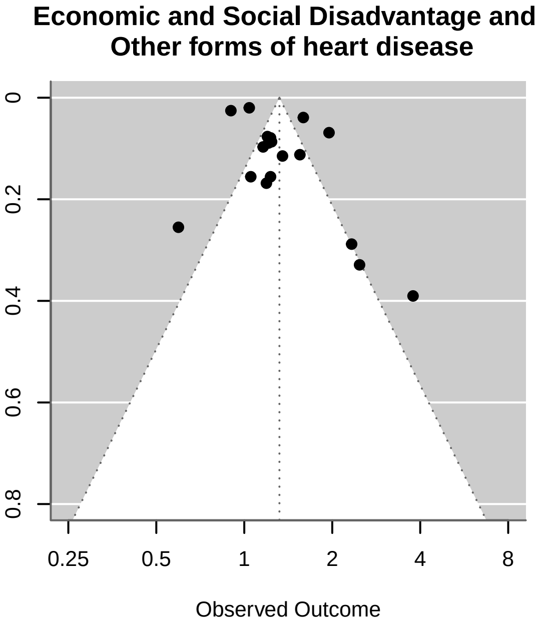

Supplementary Figure 2e

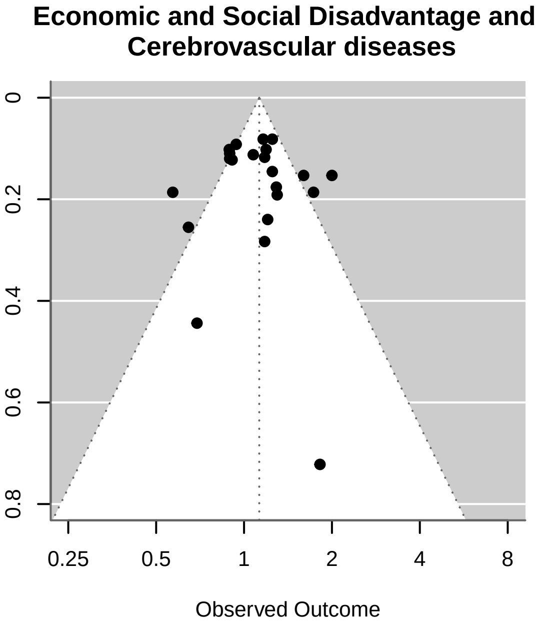

Supplementary Figure 2f

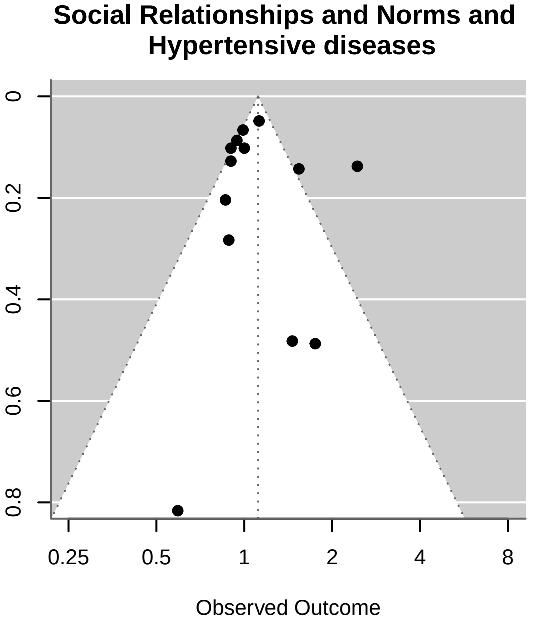

Supplementary Figure 2g

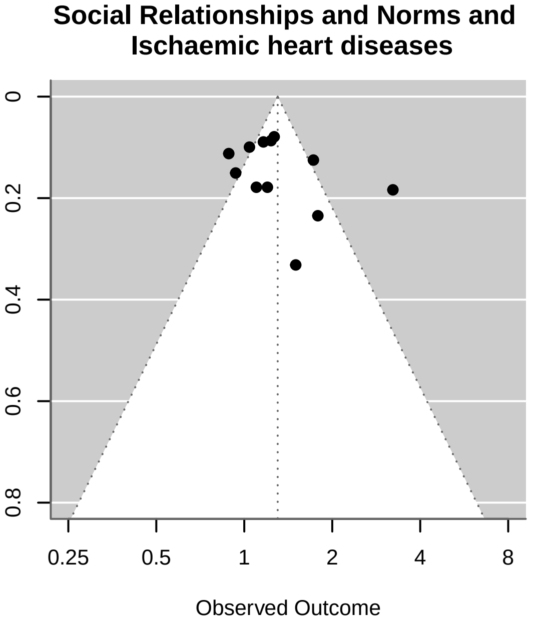

Supplementary Figure 2h

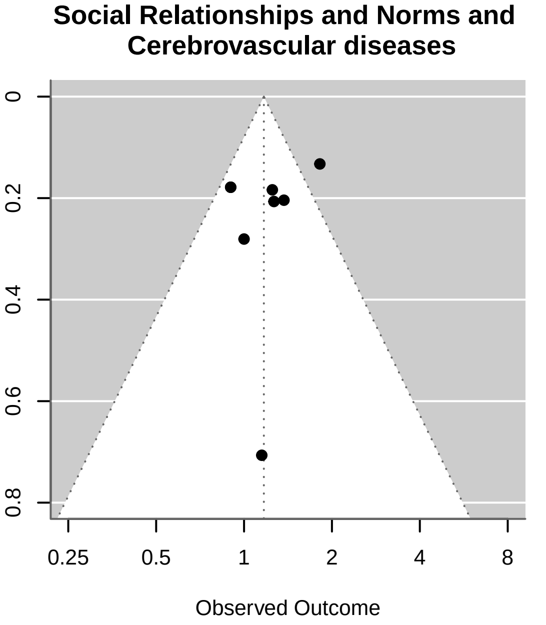

Supplementary Figure 2i

Supplementary Figure 2A-2I Funnel Plots

Note: Figures do not display standard errors larger than 0.8 (i.e., one in figure 2b, one in figure 2c,and two in figure 2d)
