# Supplementary material for: Associations between dimensions of the social environment and cardiometabolic health outcomes: a systematic review and meta-analysis
Source: BMJ Open. 2024 Aug 28;14(8):e079987. doi: 10.1136/bmjopen-2023-079987 (PMC11367359; doi:10.1136/bmjopen-2023-079987)
Supplement: online supplemental file 6 [file bmjopen-14-8-s006.pdf]

Supplementary Table 1. Characteristics of included studies

Reference	First author, year	Study design	Population description	Sample Size	Average age (years)	Women (% of sample)	Country	Income
(1)	Abba, 2021	cross-sectional	general adult population	888,925	not reported	84%	Albania, Bangladesh, Benin, Ghana, Haiti, India, the Kyrgyz Republic, Lesotho, Namibia, Nepal, South Africa and Tajikistan	UPPER-MIDDLE and MIDDLE
(2)	Adams, 2009	cross-sectional	general adult population	4,060	not reported	not reported	Australia	HIGH
(3)	Agabiti , 2009	cross-sectional	general adult population	3,391,127	54.0 (SD 9.4)	39%	Italy	HIGH
(4)	Agyemang, 2007	cross-sectional	general adult population	1,322	not reported	not reported	Netherlands	HIGH
(5)	Ahern, 2005	cross-sectional	general adult population + oversample of Black and over 60y people	769	62.7 (SD 14.4)	64%	USA	HIGH
(6)	Akwo , 2018	longitudinal	resource-limited underinsured population + general population	27,078	55.5 (SD 10.4)	63%	USA	HIGH
(7)	Alemi, 2023	longitudinal	general population	not reported	not reported	not reported	USA	HIGH
(8)	Allan, 2020	cross-sectional	adult population treated by the emergency department for out-of-hospital cardiac arrest (OHCA)	3,746	38.8 (SD 3.6)	52%	Canada	HIGH
(9)	Altevers, 2016	longitudinal	general adult population	8,952	not reported	48%	Germany	HIGH
(10)	Andersen, 2008	cross-sectional	adult female population	4,286	not reported	100%	UK	HIGH
(11)	Anderson, 2019	longitudinal	general adult population	379,673	2005: 51.7 (SD 17.1); 2007: 54.1 (SD 16.8)	62%	USA	HIGH
(12)	Atasoy, 2022	longitudinal	general adult population	9,448	49.2 (SD 11.7)	49%	Germany	HIGH
(13)	Augustin, 2008	cross-sectional	general adult population	1,140	59.0 (no SD)	66%	USA	HIGH

(14)	Avogo, 2023	cross-sectional	general female adult population	9,396	not reported	100%	Ghana	LOWER-MIDDLE
(15)	Baek, 2016	cross-sectional	general adult population	595	Men 71.9 (SD 6.5); Women 70.6 (SD 7.8)	58%	South Korea	HIGH
(16)	Banchani, 2020	cross-sectional	adult female population	2,504	not reported	100%	Ghana	LOWER-MIDDLE
(17)	Bancks, 2017	longitudinal	general adult population	4,251	At baseline: 25 (SD 3.6)	54%	USA	HIGH
(18)	Barber, 2016	cross-sectional	African American adult population	4,096	not reported	not reported	USA	HIGH
(19)	Barber, 2018	cross-sectional	civil servants	10,617	52.5 (SD 9.2)	56%	Brazil	UPPER-MIDDLE
(20)	Barefoot, 2005	longitudinal	general adult population	9,573	57.5 (no SD)	56%	Denmark	HIGH
(21)	Basile Ibrahim, 2005	cross-sectional	African American adult women with a child	250	31.3 (no SD)	100%	USA	HIGH
(22)	Bevan, 2023	cross-sectional	general adult population	308,243,060	SVI Q1: 42 (SD 7.6), SVI Q2: 40.4 (SD 7.7), SVI Q3: 38.5 (SD 7.1), SVI Q4: 34.4 (SD 6.3)	not reported	USA	HIGH
(23)	Bhavsar, 2022	longitudinal	general adult population	10,807	41.7 (IQR 27.8–53.9)	55%	USA	HIGH
(24)	Bhopal, 2002	cross-sectional	adult population from different ethnic groups	2,193	not reported	51%	UK	HIGH
(25)	Bilal, 2018	longitudinal	general adult population	269,942	56.5 (IQR 47.4-69.8)	55%	Spain	HIGH
(26)	Bocour, 2016	longitudinal	general adult population	not reported	not reported	not reported	USA	HIGH
(27)	Borges, 2021	cross-sectional	not reported	not reported	not reported	not reported	USA	HIGH
(28)	Boruzs, 2018	cross-sectional	general adult population	not reported	not reported	not reported	Hungary	HIGH
(29)	Bravo, 2018	cross-sectional	general adult population	147,351	not reported	58%	USA	HIGH
(30)	Bravo, 2019	cross-sectional	general adult population	147,351	not reported	58%	USA	HIGH
(31)	Bray, 2018	longitudinal	whole British population + adults hospitalised for first-ever acute ischemic stroke or primary intracerebral haemorrhage	43,749,578	not reported	50%	UK	HIGH

(32)	Brinkhues, 2017	cross-sectional	general adult population + oversample of individuals with T2DM	2,861	60.0 (SD 8.2)	49%	Netherlands	HIGH
(33)	Bush, 2023	longitudinal	general male adult population	3,706	Q1: 68.48 (SD 5.47); Q2: 68.26 (SD 5.53); Q3: 68.80 (SD 5.68); Q4 68.79 (SD 5.69); Q5: 69.09 (SD 5.30)	0%	UK	HIGH
(34)	Butler, 2010	cross-sectional	general population	19,567,428	not reported	not reported	Australia	HIGH
(35)	Buys, 2015	cross-sectional	general adult population	1,000	74.3 (SD 6.7)	49%	USA	HIGH
(36)	Carlsson, 2016	longitudinal	general adult population	1,151,652	At baseline: 41 (no SD)	49%	Sweden	HIGH
(37)	Carlsson, 2017	longitudinal	general adult population	1,153,451	At baseline: 40 (no SD)	49%	Sweden	HIGH
(38)	Cebrecos, 2018	cross-sectional	general population	1,446,994	not reported	not reported	Spain	HIGH
(39)	Cené, 2022	longitudinal	general female adult population	36,457	62.6 (SD 7.2)	100%	USA	HIGH
(40)	Chaix, 2011	cross-sectional	adult population participating in a voluntary free medical examination	6,876	not reported	not reported	France	HIGH
(41)	Chamberlain, 2022	cross-sectional	general adult population	197,578	not reported	54%	USA	HIGH
(42)	Chan, 2022	cross-sectional	general adult population	94,114	46.2 (no SD)	52%	Brazil	UPPER-MIDDLE
(43)	Chang, 2017	longitudinal	female nurses	76,362	57.9 (SD 7.1)	100%	USA	HIGH
(44)	Chatzi, 2020	longitudinal	general adult population	5,179	not reported	56%	UK	HIGH
(45)	Cheruvalath, 2022	case-control	Cases: patients with stroke; Controls: patients with non-stroke diagnosis	Cases: 1,174; Controls: 4,696	70 (no SD)	53%	USA	HIGH
(46)	Child , 2022	longitudinal	not reported	1,159	not reported	21-30 y: 51%; 50-70 y: 54%	USA	HIGH
(47)	Christine, 2015	longitudinal	general adult population	5,124	60.7 (SD 9.9)	54%	USA	HIGH
(48)	Chum, 2015	cross-sectional	general adult population	2,411	44.0 (no SD)	54%	Canada	HIGH

(49)	Clark, 2011	longitudinal	Blacks and non-Hispanic whites adult population	3,816	74.7 (SD 7.0)	60%	USA	HIGH
(50)	Claudel, 2018	longitudinal	general adult population	1,174	40.1 (SD 9.6)	58%	USA	HIGH
(51)	Coelho, 2023	cross-sectional	adult latin American population	109,184	42.7 (SD 16.4)	58%	Latin America (Argentina, Brazil, Chile, Colombia, Mexico, Peru)	UPPER-MIDDLE and HIGH
(52)	Cofie, 2021	cross-sectional	general adult population	1,153	Distribution: 21-30: 40.5%; 50-70: 59.4%	53%	USA	HIGH
(53)	Connolly, 2000	cross-sectional	general population	285,157	not reported	not reported	UK	HIGH
(54)	Consolazio, 2020	cross-sectional	general adult population	2,056	not reported	47%	Netherlands	HIGH
(55)	Cookson, 2012	cross-sectional	general adult population	not reported	not reported	not reported	UK	HIGH
(56)	Cox, 2007	cross-sectional	general adult population	33,820	not reported	not reported	Scotland	HIGH
(57)	Cozier, 2007	longitudinal	adult Black female population	36,099	36 (no IQR)	100%	USA	HIGH
(58)	Cromer, 2023	cross-sectional	general population	MGBB: European ancestry: 27,164; UKB: European ancestry: 216,384	MGBB: European ancestry: 61.4 (SD 16.6); UKB: European ancestry: 56.5 (SD 8.1)	MGBB: European ancestry: 53%; UKB: European ancestry: 53%	USA/UK (replication analysis)	HIGH
(59)	Cubbin, 2006	cross-sectional	general adult population	18,081	not reported	51%	Sweden	HIGH
(60)	Cunningham, 2018	cross-sectional	general population	not reported	not reported	not reported	USA	HIGH
(61)	Cuthbertson, 2018	longitudinal	adult population users of healthcare provider	109,756	At baseline: 72.0 (SD 7.4)	60%	USA	HIGH
(62)	de Oliveira , 2023	cross-sectional	civil servants	Belo Horizonte: 2,486; Salvador: 1,849	not reported	Belo Horizonte: 54%; Salvador 59%	Brazil	UPPER-MIDDLE
(63)	de Silva, 2022	cross-sectional	general population	not reported	not reported	not reported	USA	HIGH
(64)	Desmond, 2015	cross-sectional	leaseholders of rental houses	1,048	38.8 (SD 14.7)	62%	USA	HIGH

(65)	Diez-Roux, 2002	longitudinal	general adult population	8,187	not reported	55%	USA	HIGH
(66)	Diez-Roux, 1997	cross-sectional	general adult population	12,601	not reported	55%	USA	HIGH
(67)	Diez-Roux, 2000	cross-sectional	general adult population	70,534	not reported	57%	USA	HIGH
(68)	Djekic, 2018	cross-sectional	general adult population	1,067	57.7 (SD 4.4)	51%	Sweden	HIGH
(69)	Dragano, 2007	cross-sectional	general adult population	11,554	Czech Republic 57.8 (SD 6.9); Germany 57.7 (SD 6.6)	Czech Republic 55%; Germany 51%	Czech Republic and Germany	HIGH
(70)	Dubowitz, 2012	cross-sectional	general female adult population	60,775	68.2 (SD 7.3)	100%	USA	HIGH
(71)	Dwane, 2020	cross-sectional	general population	25,532	not reported	58%	South Africa	UPPER-MIDDLE
(72)	Dyck, 2021	cross-sectional	Healthcare service beneficiares (at some point)	not reported	not reported	not reported	USA	HIGH
(73)	Ekholuenetale, 2020	cross-sectional	general female adult population	7,712	not reported	100%	Benin	LOWER-MIDDLE
(74)	Eng, 2002	longitudinal	male adult population (health professionals)	28,369	not reported	0%	USA	HIGH
(75)	Engstrom, 2001	cross-sectional	general adult population	28,466	not reported	60%	Sweden	HIGH
(76)	Eschbach, 2004	cross-sectional	general adult population	3,050	not reported	not reported	USA	HIGH
(77)	Essien, 2022	longitudinal	general adult population	28,858	51.4 (SD 11.3)	57%	USA	HIGH
(78)	Exeter, 2015	longitudinal	general adult population	641,532	not reported	52%	New Zealand	HIGH
(79)	Faka, 2018	cross-sectional	general adult population	2,445	not reported	not reported	Greece	HIGH
(80)	Feero, 1995	cross-sectional	general population	≈ 500,000	not reported	not reported	USA	HIGH
(81)	Ferguson, 2020	cross-sectional	general young adult population	2,556	17.9 (SD 2.0)	57%	Jamaica	UPPER-MIDDLE
(82)	Fitzpatrick, 2020	cross-sectional	general adult population	103,020,808	not reported	not reported	USA	HIGH
(83)	Fleischer, 2008	cross-sectional	general adult population	1,510	Men: 45.5 (SD 18.9); Women 49.6 (SD 20.0)	59%	Argentina	UPPER-MIDDLE

(84)	Ford, 2006	cross-sectional	general adult population	14,818	Men: 44.0 (SE 0.4); Women: 45.7 (SE 0.5)	53%	UK	HIGH
(85)	Forsberg, 2018	longitudinal	general population	4,721,081	not reported	not reported	Sweden	HIGH
(86)	Forsberg , 2023	longitudinal	general adult population	892,321	not reported	52%	Sweden	HIGH
(87)	Freedman, 2011	longitudinal	general adult population	not reported	not reported	57%	USA	HIGH
(88)	Gao, 2022	longitudinal	general adult population	1,937	Non-Hispanic Black: 58.4 (SD 9.8); Hispanic: 58.0 (SD 9.7); Chinese: 59.4 (SD 9.8)	Non-Hispanic Black: 52%; Hispanic: 49%; Chinese: 50%	USA	HIGH
(89)	Garcia, 2015	longitudinal	Latino adult population	1,777	70.7 (SD 7.1)	59%	USA	HIGH
(90)	Garcia, 2016	longitudinal	Latino adult population	1,777	not reported	not reported	USA	HIGH
(91)	Gary-Webb, 2023	cross-sectional	general population (urban residents)	849	48 (SD 18.5)	67%	Jamaica	UPPER-MIDDLE
(92)	Gaskin, 2014	cross-sectional	Black and non-White adult population	4,403	not reported	47%	USA	HIGH
(93)	Gebreab, 2017	longitudinal	African American adult population	3,670	not reported	63%	USA	HIGH
(94)	Gero, 2022	longitudinal	general adult population	5,351	46.6 (SD 2.2)	53%	USA	HIGH
(95)	Glover, 2019	cross-sectional	African American adult population	5,104	55.3 (no SD)	64%	USA	HIGH
(96)	Grundmann, 2014	cross-sectional	general adult population	39,908	not reported	54%	Germany	HIGH
(97)	Guion, 2024	longitudinal	general adult population	24,228,526	not reported	not reported	France	HIGH
(98)	Gwon, 2020	longitudinal	general adult population	356,126	not reported	49%	South Korea	HIGH
(99)	Halonen, 2015	longitudinal	public sector workers	37,699	49.9 (SD 10.4)	79%	Finland	HIGH
(100)	Hamad, 2020	longitudinal	adult immigrants	49,305	30.5 (IQR 24.9-39.8)	43%	Denmark	HIGH
(101)	Hanefeld, 2018	longitudinal	general population	365,406	not reported	not reported	Germany	HIGH
(102)	Hanigan, 2020	cross-sectional	general adult population	not reported	not reported	not reported	USA	HIGH
(103)	Harding , 2022	longitudinal	African American adult population	1,516	50.0 (SD 12.0)	36%	USA	HIGH

(104)	Hashemi, 2023	cross-sectional	general adult population	10,009	not reported	60%	Iran	LOWER-MIDDLE
(105)	Hassen, 2020	longitudinal	general adult population	14,322	64.9 (SD 10.9)	56%	Belgium and France	HIGH
(106)	Hawkins, 2012	cross-sectional	general adult population	not reported	not reported	not reported	UK	HIGH
(107)	Heeley, 2011	longitudinal	general population	not reported	not reported	not reported	Australia and New Zealand	HIGH
(108)	Hendryx, 2020	longitudinal	adult female population	139,924	not reported	100%	USA	HIGH
(109)	Henriksson, 2010	longitudinal	general adult population	1,284,955	not reported	50%	Sweden	HIGH
(110)	Heredia, 2022	cross-sectional	Latino population from poor communities	500	not reported	70%	USA/Mexico border	HIGH
(111)	Herrera-Anazco, 2019	longitudinal	patients of any healthcare interventions in health establishments of the Peruvian Ministry of Health	not reported	not reported	not reported	Peru	UPPER-MIDDLE
(112)	Herrick, 2016	cross-sectional	adults employees	15,522	41.0 (SD 12.6)	79%	USA	HIGH
(113)	Hilding, 2015	longitudinal	adult population, enriched with participants with family history for diabetes	4,963	not reported	59%	Sweden	HIGH
(114)	Hill, 2014	longitudinal	general adult population	6,642	68.0 (SD 10.7)	59%	USA	HIGH
(115)	Hofelmann, 2012	cross-sectional	general adult population	1,720	38.1 (95% CI 37.5-38.6)	56%	Brazil	UPPER-MIDDLE
(116)	Holstiege, 2019	cross-sectional	general adult population	40,481,732	not reported	not reported	Germany	HIGH
(117)	Holtgrave, 2006	cross-sectional	not reported	not reported	not reported	not reported	USA	HIGH
(118)	Honda, 2021	longitudinal	Adult population (45-64 years)	11,104	56.7 (SD 5.7)	56%	USA	HIGH
(119)	Honjo, 2015	longitudinal	general adult population	90,843	50.3 (SD 7.6)	52%	Japan	HIGH
(120)	Horsten, 1999	cross-sectional	general female adult population	300	56.0 (SD 7.0)	100%	Sweden	HIGH

(121)	Hosseini, 2020	cross-sectional	general adult population	28,238	62.8 (SD 10.2)	51%	Canada	HIGH
(122)	Howard, 2016	longitudinal	general adult population + oversample of black participants and residents of the "stroke belt"	24,875	not reported	49%	USA	HIGH
(123)	Hu, 2020	cross-sectional	general adult population	not reported	not reported	not reported	USA	HIGH
(124)	Hu, 2021	cross-sectional	adults who trained for and/or participated in oil spill response and clean-up	9,626	not reported	not reported	USA	HIGH
(125)	Huang, 2022	cross-sectional	Adult population (≥ 18 years)	not reported	not reported	not reported	USA	HIGH
(126)	Hwang, 2020	longitudinal	general adult population	2,156,829	not reported	not reported	South Korea	HIGH
(127)	Jack, 2019	longitudinal	general population	not reported	not reported	not reported	UK	HIGH
(128)	Jackson, 2008	cross-sectional	general adult population	5,226	not reported	56%	UK	HIGH
(129)	Jadow, 2023	cross-sectional	Adult population (≥ 18 years)	not reported	not reported	not reported	USA	HIGH
(130)	Jain, 2022	cross-sectional	Adult population (≥ 18 years)	1,745,999	not reported	SVI tertile 1: 51%; SVI tertile 2: 51%; SVI tertile 3: 51%	USA	HIGH
(131)	Jensen, 2023	quasi-randomised	refugees	37,281	Distribution: 18-24: 23.6%; 25-29: 21.4%; 30-34: 16.7%; 35-39: 11.9%; 40-44: 7.5%; 45-49: 4.7%; >= 50: 14.3%	45%	Denmark	HIGH
(132)	Jonsson, 2020	cross-sectional	general population	2,308,143	not reported	not reported	Sweden	HIGH
(133)	Jung, 2019	cross-sectional	general adult population	Sample 1 (NFHS-4): 757655; Sample 2 (DLHS-4/AHS): 1,618,282	Sample 1: Female: 30.2 (SD 9.9); Male: 31.8 (SD 11.1); Sample 2 (DLHS-4/AHS): Female: 40.7 (SD 15.6) 40.80 (SD 16.2)	Sample 1: 85%; Sample 2: 48%	India	LOWER-MIDDLE
(134)	Kaiser, 2016	longitudinal	general adult population	3,382	59.1 (no SD)	51%	USA	HIGH
(135)	Kakinami, 2017	longitudinal	population of children followed into adulthood	3,820	At baseline: boys 9.6 (SD 2.6); girls 9.4 (SD 2.6)	50%	Canada	HIGH

(136)	Kauhl, 2018	cross-sectional	adult population insurants of heathcare insurer	≈1.8 million	not reported	not reported	Germany	HIGH
(137)	Kawachi, 1996	longitudinal	male adult population (health professionals)	32,624	not reported	0%	USA	HIGH
(138)	Keita, 2014	cross-sectional	general adult population + oversampled of African Americans	19,079	not reported	not reported	USA	HIGH
(139)	Kelli, 2017	cross-sectional	university employees	1,421	49.4 (SD 10.2)	62%	USA	HIGH
(140)	Kershaw, 2011	cross-sectional	general adult population	8,071	Black: 45.7 (SD 0.3); White: 49.1 (SD 0.3)	Black: 55%; White: 51%	USA	HIGH
(141)	Kim, 2018	quasi-randomised	general adult population (residents of public housing projects)	28,858	not reported	not reported	Canada	HIGH
(142)	Kim, 2022	quasi-randomised	Black adult population	1,053	28 (range 18-44)	60%	USA	HIGH
(143)	Kim, 2022	longitudinal	general adult population	8,826	63.9 (SE 0.16)	58%	USA	HIGH
(144)	Kivimaki, 2018	longitudinal	children followed up into adulthood	3,002	At baseline: 10.9 (SD 4.4)	52%	Finland	HIGH
(145)	Kivimaki, 2021	longitudinal	general adult population and public sector personnel	114,786	44.4 (SD 11.1)	76%	Finland	HIGH
(146)	Kling, 2007	randomised	general adult population	2,533 (Control group: 1,080; Tradicional voucher: 993; Experimental: 1,453)	At baseline: Control: 39.6 years (no SD); Tradicional voucher: 40.1 years (no SD); Experimental: 39.7 years (no SD)	Control: 98%; Tradicional voucher: 98%; Experimental: 99%	USA	HIGH
(147)	Kolpak, 2017	cross-sectional	general population	Toronto: 2,503,280; Chicago: 2,695,598	not reported	not reported	USA and Canada	HIGH
(148)	Krieger, 1992	cross-sectional	Healthcare service beneficiares	14,420	not reported	White: 54%; Black: 60%	USA	HIGH

(149)	Krishnan, 2010	longitudinal	African American female adult population	46,382	not reported	100%	USA	HIGH
(150)	Kwok, 2021	cross-sectional	general population	17,607	44.0 (SD 20.3)	53%	China	UPPER-MIDDLE
(151)	Lachkhem, 2018	longitudinal	general population	not reported	not reported	not reported	France	HIGH
(152)	Lagisetty, 2016	cross-sectional	adult South Asians ancestry population living in USA	906	55.0 (SD 9.4)	46%	USA	HIGH
(153)	Larranaga, 2005	cross-sectional	general adult population	64,526	not reported	52%	Spain	HIGH
(154)	Laursen, 2017	longitudinal	general adult population	7,662	63.0 (SD 56.7)	56%	UK	HIGH
(155)	Lawlor, 2005	cross-sectional	general female adult population	4,286	68.9 (no SD)	100%	UK	HIGH
(156)	Lee, 2018	cross-sectional	adult population users of emergency care	≈5 million	not reported	not reported	USA	HIGH
(157)	Lee, 2018	cross-sectional	general adult population	33,216,621	not reported	51%	South Korea	HIGH
(158)	Lemstra, 2006	cross-sectional	general population	184,284	not reported	not reported	Canada	HIGH
(159)	Leyland, 2005	cross-sectional	general adult population	8,804	not reported	55% among those with the condition and 57% among those without condition	UK	HIGH
(160)	Li, 2017	cross-sectional	adult Latino population	1,563	41.8 (SD 15.0)	69%	USA	HIGH
(161)	Linde, 2023	cross-sectional	not reported	not reported	not reported	not reported	USA	HIGH
(162)	Ling, 2009	longitudinal	general adult population	16,000	63.9 (SD 10.3)	59%	China	UPPER-MIDDLE
(163)	Lippert, 2017	longitudinal	general adult population (adolescents followed into adulthood)	11,767	15.4 (SD 0.03)	51%	USA	HIGH
(164)	Lonn, 2019	longitudinal	general adult population	3,140,657	not reported	48%	Sweden	HIGH

(165)	Loucks, 2006	cross-sectional	general adult population	3,231	not reported	54%	USA	HIGH
(166)	Lu, 2019	cross-sectional	Asian American adult population	530	not reported	58%	USA	HIGH
(167)	Ludwig, 2011	randomised	adult female population from high poverty areas	4,498	not reported	100%	USA	HIGH
(168)	Lukachko, 2014	cross-sectional	general adult population	32,752	not reported	not reported	USA	HIGH
(169)	Lukaschek, 2017	longitudinal	general adult population	6,839	not reported	48%	Germany	HIGH
(170)	Lund, 2012	longitudinal	general adult population	4,573	not reported	50%	Denmark	HIGH
(171)	Lund, 2014	longitudinal	general adult population	8,550	not reported	55%	Denmark	HIGH
(172)	Ma, 2021	cross-sectional	general adult population	14,585	72.6 (SD 11.5)	55%	China, Ghana, India, Mexico, Russia, and South Africa	UPPER-MIDDLE and LOWER-MIDDLE
(173)	Madela, 2023	cross-sectional	Black South Africans population	7,303	not reported	72%	South Africa	UPPER-MIDDLE
(174)	Madela, 2023	cross-sectional	Black South Africans population	3,685	60.5 (SD 11.2)	75%	South Africa	UPPER-MIDDLE
(175)	Maheswaran, 2018	cross-sectional	general adult population	10,185	not reported	not reported	UK	HIGH
(176)	Maier, 2014	cross-sectional	general adult population	33,690	not reported	57%	Germany	HIGH
(177)	Malino, 2014	cross-sectional	female adult population visiting hospital's dispensaries	306	not reported	100%	Haiti	LOWER-MIDDLE
(178)	Marley, 2015	longitudinal	adolescents followed into adulthood	11,110	not reported	not reported	USA	HIGH
(179)	Massa, 2016	cross-sectional	general adult population	1,333	not reported	60%	Brazil	UPPER-MIDDLE
(180)	Matheson, 2010	cross-sectional	general adult population	103,419	not reported	51%	Canada	HIGH
(181)	Matthew, 2018	longitudinal	general adult population	2,604,341	54.5 (SD 16.3)	59%	USA	HIGH

(182)	Mayne, 2020	longitudinal	Black adult population	2,175	not reported	not reported	USA	HIGH
(183)	McDoom, 2018	longitudinal	general adult population	3,372	61.0 (SD 5.5)	55%	USA	HIGH
(184)	Menec, 2010	cross-sectional	general adult population	77,930	not reported	59%	USA	HIGH
(185)	Mentias, 2023	longitudinal	White and Black healthcare provider beneficiaries	2,388,955	71.2 (SD 11.6)	53%	USA	HIGH
(186)	Metcalf, 2008	cross-sectional	general adult population	4,020	not reported	52%	New Zealand	HIGH
(187)	Mezuk, 2014	cross-sectional	general adult population	887,603	not reported	not reported	Sweeden	HIGH
(188)	Mohottige, 2023	cross-sectional	general population	not reported	35.6 (IQR 31.2-42.9)	not reported	USA	HIGH
(189)	Moore, 2014	cross-sectional	general adult population	2,616	not reported	65%	Canada	HIGH
(190)	Morenoff, 2007	cross-sectional	general adult population	2,933	not reported	not reported	USA	HIGH
(191)	Morris, 2008	longitudinal	general male adult population	5,049	not reported	0%	UK	HIGH
(192)	Mujahid, 2008	cross-sectional	general adult population	2,612	62.0 (SD 10.0)	54%	USA	HIGH
(193)	Müller, 2013	cross-sectional	general adult population	8,871	not reported	49%	Germany	HIGH
(194)	Müller, 2013	cross-sectional	general adult population	8,879	not reported	49%	Germany	HIGH
(195)	Murray, 2010	longitudinal	general female adult population	2,592	not reported	100%	USA	HIGH
(196)	Nakagomi, 2019	cross-sectional	general adult population	116,013	73.5 (SD 6.1)	50%	Japan	HIGH
(197)	Nazmi, 2010	cross-sectional	general adult population	5,370	62.2 (SD 10.2)	not reported	USA	HIGH
(198)	Neufcourt, 2019	cross-sectional	general adult population	62,247	47.9 (SD 13.4)	53%	France	HIGH
(199)	Nikulina, 2014	longitudinal	neglected and non-neglected children followed up into adulthood	539	41.0 (no SD)	51%	USA	HIGH
(200)	Odoi, 2020	longitudinal	general population	not reported	not reported	not reported	USA	HIGH

(201)	Ogungbe, 2021	cross-sectional	African American adult population	465	46.8 (SD 11.5)	60%	USA	HIGH
(202)	Ohanyan, 2022	cross-sectional	general adult population	14,829	50.7 (SD 9.4)	56%	Netherlands	HIGH
(203)	Oktamianti, 2022	cross-sectional	general adult population	624,563	not reported	not reported	Indonesia	UPPER-MIDDLE
(204)	Oladele, 2020	cross-sectional	general adult population	1,989	57.1 (SD 10.4)	68%	USA Virgin Islands, Puerto Rico, Trinidad and Tobago, and Barbados	HIGH
(205)	Omariba, 2014	longitudinal	general adult population	1,459,953	not reported	52%	Canada	HIGH
(206)	Osborn, 2023	cross-sectional	Latino adults	23,478	43.0 (no SD)	54%	USA	HIGH
(207)	Pantell, 2019	longitudinal	adult population user of healthcare provider	18,133	56.2 (SD 16.9)	56%	USA	HIGH
(208)	Penninx, 1999	cross-sectional	general adult population	2,788	not reported	51%	Netherlands	HIGH
(209)	Pichora, 2018	cross-sectional	general adult population	24,070,000	not reported	not reported	Canada	HIGH
(210)	Pinheiro, 2020	longitudinal	general adult population + oversample of participants from regions with high stroke mortality	25,790	not reported	not reported	USA	HIGH
(211)	Piwońska, 2023	cross-sectional	general adult population	6,043	Women: 51 (IQR 37-62); Men: 49 (IQR 35-61)	55%	Poland	HIGH
(212)	Ptushkina, 2021	cross-sectional	general adult population	6,601	SHIP-TREND: 53.9 (SD 13.3); KORA-F4: 55.7 (SD 12.9)	SHIP-TREND: 52%; KORA-F4: 51%	Germany	HIGH
(213)	Quashie, 2023	longitudinal	general adult population	2,769	Distribution: 60–64: 31.3%; 65–69: 25.6%; 70–74: 19.5%; 75–79: 12.9%; 80–84: 6.5%; 85+: 4.4%	57%	Porto Rico	HIGH
(214)	Quiñones, 2021	cross-sectional	general adult population	not reported	not reported	not reported	USA	HIGH

(215)	Rachele, 2016	cross-sectional	general adult population	10,620	not reported	55%	Australia	HIGH
(216)	Redondo-Sendino, 2005	cross-sectional	general adult population	2,367	Women: 72.4 (SD 7.7); Men: 71.0 (SD 8.0)	57%	Spain	HIGH
(217)	Rethy, 2021	longitudinal	patients of healthcare provider	28,858	51.4 (SD 11.3)	57%	USA	HIGH
(218)	Riddell, 2004	cross-sectional	general adult population	not reported	not reported	not reported	New Zealand	HIGH
(219)	Rod, 2011	longitudinal	general adult population	8,670	57.0 (15.0)	57%	Denmark	HIGH
(220)	Rose, 2009	longitudinal	general adult population	359,866	not reported	not reported	USA	HIGH
(221)	Safford, 2021	longitudinal	general adult population + oversample of participants from regions with high stroke mortality	22,152	not reported	59%	USA	HIGH
(222)	Salinas, 2017	longitudinal	general adult population	3,210	65.0 (SD 11.0)	56%	USA	HIGH
(223)	Samuel, 2015	cross-sectional	general adult population (racially integrated & low income)	1,326	White: 43.8 (16.1); African American: 38.3 (13.2)	White: 57%; African American: 53%	USA	HIGH
(224)	Savin, 2022	longitudinal	Hispanic/Latinos adults	2,090	39.4 (SE 0.5)	53%	USA	HIGH
(225)	Schieb, 2013	longitudinal	adult population user of healthcare provider	≈56 million	not reported	not reported	USA	HIGH
(226)	Schootman, 2007	longitudinal	adult African American population	644	56.2 (SD 4.3)	56%	USA	HIGH
(227)	Schwartz, 2021	longitudinal	general adult population	≈1.6 million	not reported	not reported	USA	HIGH
(228)	Schwartz, 2022	case-control	Healthcare service beneficiares	Cases: 15,888; Controls: 79,435	55 (no SD)	not reported	USA	HIGH
(229)	Sharma, 2023	cross-sectional	general adult population	14,652	38.6 (SD 17.6)	57%	Nepal	LOWER-MIDDLE

(230)	Sharp, 2023	longitudinal	general adult population from poverty strata	1,046	44.0 (SD 15.5)	48%	USA	HIGH
(231)	Sheets, 2017	cross-sectional	general adult population (primary care patients)	4,770	not reported	not reported	USA	HIGH
(232)	Shibayama, 2018	longitudinal	general adult population	27,079	54.6 (SD 2.7)	53%	Japan	HIGH
(233)	Siegel, 2015	cross-sectional	general adult population	79,610	not reported	52%	Germany	HIGH
(234)	Singh, 2016	cross-sectional	adult female population	124,385	not reported	100%	India	LOWER-MIDDLE
(235)	Smith, 1998	cross-sectional	general adult population	14,952	not reported	53%	UK	HIGH
(236)	Smurthwaite, 2017	cross-sectional	general adult population (primary care patients)	20,594	not reported	55%	Australia	HIGH
(237)	Spicer, 1993	case-control	Cases: survivors of myocardial infarction; Controls: general adult population	Cases: 273; Controls: 770	Cases: Male 52.7 (SD 7.3), Female 56.5 (SD 7.2); Controls: Male 52.7 (SD 6.9), Female 55.2 (SD 6.4)	Cases: 24%; Controls: 41%	New Zealand	HIGH
(238)	Splan, 2021	cross-sectional	adult patients of healthcare provider	not reported	not reported	not reported	USA	HIGH
(239)	Steckel, 2013	longitudinal	general population	not reported	not reported	not reported	USA	HIGH
(240)	Suchy-Dicey, 2022	longitudinal	adult American Indians population	2,786	40.8 (SD 17.3)	60%	USA	HIGH
(241)	Sun, 2020	cross-sectional	general adult population	252,650	56.0 (16.8)	51%	USA	HIGH
(242)	Sundquist, 2004	longitudinal	general adult population	637,628	not reported	49%	Sweden	HIGH
(243)	Swain, 2019	cross-sectional	general adult population	not reported	not reported	not reported	India	LOWER-MIDDLE
(244)	Tang, 2015	cross-sectional	general adult population (rural population)	14,424	not reported	66%	China	UPPER-MIDDLE
(245)	Tapager, 2023	cross-sectional	general population	not reported	not reported	not reported	Denmark	HIGH

(246)	Terashima, 2014	cross-sectional	general young population	11,233	not reported	not reported	Canada	HIGH
(247)	Thrift, 2006	longitudinal	general adult population	306,631	not reported	not reported	Australia	HIGH
(248)	Tompkins, 2010	cross-sectional	general adult population	352,395	not reported	52%	Canada	HIGH
(249)	Trifan, 2023	cross-sectional	adult American Latin population	11,623	Cases: 58.9 (56.4-61.5); Non-cases: 46.8 (46.3-47.4)	Cases: 51%; Non-cases: 52%	USA	HIGH
(250)	Tung, 2018	cross-sectional	adult patients of primary care center	14,331	56 (IQR 38–72)	77%	USA	HIGH
(251)	Tung, 2019	longitudinal	adult patients of academic medical center	17,783	58.0 (IQR 41–71)	68%	USA	HIGH
(252)	Uddin, 2022	cross-sectional	general adult population + oversampled Black individuals and individuals residing in southern states	27,159	Higher Density Urban: 65.2 (SD 9.6); Lower Density Urban: 65.2 (SD 9.5); Suburban/Small Town: 64.5 (SD 9.2); Rural: 63.9 (SD 9.1)	Higher Density Urban: 60%; Lower Density Urban: 54%; Suburban/Small Town: 53%; Rural: 54%	USA	HIGH
(253)	Uddin, 2023	longitudinal	REGARDS: general adult population; G/JHU: general adult population (case-control); VADR: veterans	REGARDS: 11,208; G/JHU: 95,323; VADR: 4,100,650	REGARDS: 63.0 (8.5); G/JHU: 54.9 (15.2); VADR: 59.4 (17.2)	REGARDS: 56%; G/JHU: 49.1%; VADR: 8%	USA	HIGH
(254)	Usher, 2018	cross-sectional	general adult population (academic medical patients)	2,045	not reported	not reported	USA	HIGH
(255)	Vart, 2017	longitudinal	general adult population	12,517	not reported	not reported	USA	HIGH
(256)	Villani, 2018	longitudinal	general adult population	5,996 million	not reported	not reported	Australia	HIGH
(257)	Vintimilla, 2023	cross-sectional	Mexican American and Non-hispanic White American population	1,867	65.9 (SD 12.5)	61%	USA	HIGH

(258)	Vogt, 1992	longitudinal	adult population users of a healthcare provider	2,396	not reported	54%	USA	HIGH
(259)	Wagner, 2016	cross-sectional	general adult population	1,705	70.7 (SD 8.0)	64%	Brazil	UPPER-MIDDLE
(260)	Walter, 2019	cross-sectional	adult Latina female population	364	35.1 (9.5)	100%	USA	HIGH
(261)	Wang, 2019	cross-sectional	general population	≈10 million	not reported	not reported	China	UPPER-MIDDLE
(262)	Wang, 2021	cross-sectional	general adult population	179,059	Distribution: 18-29: 8.7% ; 30-39: 12.0%; 40-49: 22.1%; 50-59: 24.5%; 60-69: 22.2%; >70: 10.5%	53%	China	UPPER-MIDDLE
(263)	Welin, 1996	case-control	Cases: patients admitted to the hospital; Controls: general adult population	Cases: 343; Controls: 412	Cases: Male 55.6 (SD 7.4), Female 56.5 (SD 5.0); Controls: Male 55.4 (SD 5.6), Female 55.1 (SD 5.9)	Cases: 16%; Controls: 31%	Sweden	HIGH
(264)	White, 2011	cross-sectional	Black adult population	4,499	US born: 43.3 (17.5); Foreign born: 42.6 (14.8)	US born: 59%; Foreign born: 54%	USA	HIGH
(265)	White, 2016	quasi-randomised	refugees	61,386	not reported	53%	Sweden	HIGH
(266)	Wight, 2008	cross-sectional	general adult population	3,442	77.1 (SD 5.7)	62%	USA	HIGH
(267)	Williams, 2023	cross-sectional	general adult population	170,432	47.4 (SD 0.10)	52%	USA	HIGH
(268)	Xiao, 2022	longitudinal	general adult population	9,692	Stratified by race/ethnicity and quartile of neighborhood SES. Ranged from 59 (IQR 56-63) to 63 (IQR 58-68)	Stratified by race/ethnicity and quartile of neighborhood SES. Ranged from 55% to 70%	USA	HIGH
(269)	Xie, 2021	cross-sectional	general adult population	not reported	not reported	not reported	China	UPPER-MIDDLE

(270)	Xu, 2022	cross-sectional	adult female population	47,329	Distribution: <=45: 16%; 46-50: 17%; 51-55: 21%; 56-60: 20%; 61-65: 15%; >65: 14%	100%	USA	HIGH
(271)	Xu, 2023	cross-sectional	general adult population	not reported	not reported	not reported	China	UPPER-MIDDLE
(272)	Yadav, 2021	case-control	general adult population (rural population)	Cases: 90; Controls: 90	Cases: 49.5 (SD 11.9); Controls: 49.5 years (SD 11.8)	Cases: 69%; Controls: 69%	India	LOWER-MIDDLE
(273)	Yadav, 2022	cross-sectional	Healthcare service beneficiares	not reported	not reported	not reported	USA	HIGH
(274)	Yan, 2013	longitudinal	general adult population	4,619	not reported	61%	USA	HIGH
(275)	Yan, 2022	cross-sectional	general adult population	2,961	40.0 (IQR 28-55)	58%	Haiti	LOWER-MIDDLE
(276)	Yang, 2013	cross-sectional	general adult population	4,323	53.7 (SD 10.0)	55%	USA	HIGH
(277)	Yang, 2015	longitudinal	general adult population	1,264	67.3 (SD 7.8)	53%	USA	HIGH
(278)	Yang, 2016	longitudinal	general adult populations (4 lifecourse stages)	Adolescence and Young Adulthood n= 7,889; Midadulthood n = 863; Late Adulthood n =1571 and n = 4,323	Adolescence and Young Adulthood = 28.2 (SD 1.9); Midadulthood = 44.0 (SD 9.9); Late Adulthood = 67.3 (SD 10.5) and 67.3 (SD 7.1)	Adolescence and Young Adulthood = 48%; Midadulthood = 55%; Late Adulthood = 55% and 53%	USA	HIGH
(279)	Yazawa, 2016	cross-sectional	general adult population	4,582	71.7 (SD 5.2)	50%	Japan	HIGH
(280)	Young, 2018	cross-sectional	adult population with overweight or obesity and beneficiares of healthcare provider	4,060,585	not reported	not reported	USA	HIGH
(281)	Yu, 2024	cross-sectional	general adult population	10,419	66.1 (0.11)	56%	USA	HIGH

References of included studies

1. Abba MS, Nduka CU, Anjorin S, Mohamed SF, Agogo E, Uthman OA. Influence of contextual socioeconomic position on hypertension risk in low- and middle-income countries: disentangling context from composition. BMC Public Health. 2021;21(1):2218.
2. Adams RJ, Howard N, Tucker G, Appleton S, Taylor AW, Chittleborough C, et al. Effects of area deprivation on health risks and outcomes: a multilevel, cross-sectional, Australian population study. Int J Public Health. 2009;54(3):183-92.
3. Agabiti N, Pirani M, Schifano P, Cesaroni G, Davoli M, Bisanti L, et al. Income level and chronic ambulatory care sensitive conditions in adults: a multicity population-based study in Italy. BMC Public Health. 2009;9:457.
4. Agyemang C, van Hooijdonk C, Wendel-Vos W, Ujcic-Voortman JK, Lindeman E, Stronks K, Droomers M. Ethnic differences in the effect of environmental stressors on blood pressure and hypertension in the Netherlands. BMC Public Health. 2007;7:118.
5. Ahern MM, Hendryx MS. Social capital and risk for chronic illnesses. Chronic Illn. 2005;1(3):183-90.
6. Akwo EA, Kabagambe EK, Harrell FE, Jr., Blot WJ, Bachmann JM, Wang TJ, et al. Neighborhood Deprivation Predicts Heart Failure Risk in a Low-Income Population of Blacks and Whites in the Southeastern United States. Circ Cardiovasc Qual Outcomes. 2018;11(1):e004052.
7. Alemi F, Lee KH, Vang J, Lee D, Schwartz M. Social and Medical Determinants of Diabetes: A Time-Constrained Multiple Mediator Analysis. Cureus. 2023;15(9):e46227.
8. Allan KS, Ray JG, Gozdyra P, Morrison LJ, Kiss A, Buick JE, et al. High risk neighbourhoods: The effect of neighbourhood level factors on cardiac arrest incidence. Resuscitation. 2020;149:100-8.
9. Altevers J, Lukaschek K, Baumert J, Kruse J, Meisinger C, Emeny RT, Ladwig KH. Poor structural social support is associated with an increased risk of Type 2 diabetes mellitus: findings from the MONICA/KORA Augsburg cohort study. Diabet Med. 2016;33(1):47-54.
10. Andersen AF, Carson C, Watt HC, Lawlor DA, Avlund K, Ebrahim S. Life-course socio-economic position, area deprivation and Type 2 diabetes: findings from the British Women's Heart and Health Study. Diabet Med. 2008;25(12):1462-8.
11. Anderson KF, Bjorklund E, Rambotti S. Income Inequality and Chronic Health Conditions: A Multilevel Analysis of the U.S. States. Sociol Focus. 2019;52(1):65-85.
12. Atasoy S, Johar H, Kruse J, Lukaschek K, Peters A, Ladwig KH. The Association of Social Connectivity and Body Weight With the Onset of Type 2 Diabetes: Findings From the Population-Based Prospective MONICA/KORA Cohort. Psychosom Med. 2022;84(9):1050-5.
13. Augustin T, Glass TA, James BD, Schwartz BS. Neighborhood psychosocial hazards and cardiovascular disease: the Baltimore Memory Study. Am J Public Health. 2008;98(9):1664-70.
14. Avogo WA. Community characteristics and the risk of non-communicable diseases in Ghana. PLOS Glob Public Health. 2023;3(1):e0000692.
15. Baek J, Hur NW, Kim HC, Youm Y. Sex-specific effects of social networks on the prevalence, awareness, and control of hypertension among older Korean adults. J Geriatr Cardiol. 2016;13(7):580-6.
16. Banchani E, Tenkorang EY, Midodzi W. Examining the effects of individual and neighbourhood socioeconomic status/wealth on hypertension among women in the Greater Accra Region of Ghana. Health Soc Care Community. 2020.
17. Bancks MP, Kershaw K, Carson AP, Gordon-Larsen P, Schreiner PJ, Carnethon MR. Association of Modifiable Risk Factors in Young Adulthood With Racial Disparity in Incident Type 2 Diabetes During Middle Adulthood. Jama. 2017;318(24):2457-65.
18. Barber S, Hickson DA, Wang X, Sims M, Nelson C, Diez-Roux AV. Neighborhood Disadvantage, Poor Social Conditions, and Cardiovascular Disease Incidence Among African American Adults in the Jackson Heart Study. Am J Public Health. 2016;106(12):2219-26.
19. Barber S, Diez Roux AV, Cardoso L, Santos S, Toste V, James S, et al. At the intersection of place, race, and health in Brazil: Residential segregation and cardio-metabolic risk factors in the Brazilian Longitudinal Study of Adult Health (ELSA-Brasil). Soc Sci Med. 2018;199:67-76.
20. Barefoot JC, Grønbaek M, Jensen G, Schnohr P, Prescott E. Social network diversity and risks of ischemic heart disease and total mortality: findings from the Copenhagen City Heart Study. Am J Epidemiol. 2005;161(10):960-7.

21. Basile Ibrahim B, Barcelona V, Condon EM, Crusto CA, Taylor JY. The Association Between Neighborhood Social Vulnerability and Cardiovascular Health Risk Among Black/African American Women in the InterGEN Study. Nurs Res. 2021;70(5):S3-s12.
22. Bevan G, Pandey A, Griggs S, Dalton JE, Zidar D, Patel S, et al. Neighborhood-level Social Vulnerability and Prevalence of Cardiovascular Risk Factors and Coronary Heart Disease. Curr Probl Cardiol. 2023;48(8):101182.
23. Bhavsar NA, Yang LZ, Phelan M, Shepherd-Banigan M, Goldstein BA, Peskoe S, et al. Association between Gentrification and Health and Healthcare Utilization. J Urban Health. 2022;99(6):984-97.
24. Bhopal R, Hayes L, White M, Unwin N, Harland J, Ayis S, Alberti G. Ethnic and socio-economic inequalities in coronary heart disease, diabetes and risk factors in Europeans and South Asians. J Public Health Med. 2002;24(2):95-105.
25. Bilal U, Hill-Briggs F, Sánchez-Perruca L, Del Cura-González I, Franco M. Association of neighbourhood socioeconomic status and diabetes burden using electronic health records in Madrid (Spain): the HeartHealthyHoods study. BMJ Open. 2018;8(9):e021143.
26. Bocour A, Tria M. Preventable Hospitalization Rates and Neighborhood Poverty among New York City Residents, 2008-2013. J Urban Health. 2016;93(6):974-83.
27. Borges CM, Pollock JC, Crowley M, Purandare R, Sparano J, Spike K. Social capital or vulnerability: Which has the stronger connection with selected U.S. health outcomes? SSM Popul Health. 2021;15:100812.
28. Boruzs K, Juhász A, Nagy C, Szabó Z, Jakovljevic M, Bíró K, Ádány R. High Inequalities Associated With Socioeconomic Deprivation in Cardiovascular Disease Burden and Antihypertensive Medication in Hungary. Front Pharmacol. 2018;9:839.
29. Bravo MA, Anthopolos R, Kimbro RT, Miranda ML. Residential Racial Isolation and Spatial Patterning of Type 2 Diabetes Mellitus in Durham, North Carolina. Am J Epidemiol. 2018;187(7):1467-76.
30. Bravo MA, Batch BC, Miranda ML. Residential Racial Isolation and Spatial Patterning of Hypertension in Durham, North Carolina. Prev Chronic Dis. 2019;16:E36.
31. Bray BD, Paley L, Hoffman A, James M, Gompertz P, Wolfe CDA, et al. Socioeconomic disparities in first stroke incidence, quality of care, and survival: a nationwide registry-based cohort study of 44 million adults in England. Lancet Public Health. 2018;3(4):e185-e93.
32. Brinkhues S, Dukers-Muijrers N, Hoebe C, van der Kallen CJH, Dagnelie PC, Koster A, et al. Socially isolated individuals are more prone to have newly diagnosed and prevalent type 2 diabetes mellitus - the Maastricht study. BMC Public Health. 2017;17(1):955.
33. Bush KJ, Papacosta AO, Lennon LT, Rankin J, Whincup PH, Wannamethee SG, Ramsay SE. Influence of neighborhood-level socioeconomic deprivation and individual socioeconomic position on risk of developing type 2 diabetes in older men: a longitudinal analysis in the British Regional Heart Study cohort. BMJ Open Diabetes Res Care. 2023;11(5).
34. Butler DC, Petterson S, Bazemore A, Douglas KA. Use of measures of socioeconomic deprivation in planning primary health care workforce and defining health care need in Australia. Aust J Rural Health. 2010;18(5):199-204.
35. Buys DR, Howard VJ, McClure LA, Buys KC, Sawyer P, Allman RM, Levitan EB. Association between neighborhood disadvantage and hypertension prevalence, awareness, treatment, and control in older adults: results from the University of Alabama at Birmingham Study of Aging. Am J Public Health. 2015;105(6):1181-8.
36. Carlsson AC, Li X, Holzmann MJ, Wändell P, Gasevic D, Sundquist J, Sundquist K. Neighbourhood socioeconomic status and coronary heart disease in individuals between 40 and 50 years. Heart. 2016;102(10):775-82.
37. Carlsson AC, Li X, Holzmann MJ, Ärnlöv J, Wändell P, Gasevic D, et al. Neighborhood socioeconomic status at the age of 40 years and ischemic stroke before the age of 50 years: A nationwide cohort study from Sweden. Int J Stroke. 2017;12(8):815-26.
38. Cebrecos A, Dominguez-Berjon MF, Duque I, Franco M, Escobar F. Geographic and statistic stability of deprivation aggregated measures at different spatial units in health research. Appl Geogr. 2018;95:9-18.
39. Cené CW, Leng XI, Faraz K, Allison M, Breathett K, Bird C, et al. Social Isolation and Incident Heart Failure Hospitalization in Older Women: Women's Health Initiative Study Findings. J Am Heart Assoc. 2022;11(5):e022907.

40. Chaix B, Billaudeau N, Thomas F, Havard S, Evans D, Kestens Y, Bean K. Neighborhood effects on health: correcting bias from neighborhood effects on participation. Epidemiology. 2011;22(1):18-26.
41. Chamberlain AM, St Sauver JL, Finney Rutten LJ, Fan C, Jacobson DJ, Wilson PM, et al. Associations of Neighborhood Socioeconomic Disadvantage With Chronic Conditions by Age, Sex, Race, and Ethnicity in a Population-Based Cohort. Mayo Clin Proc. 2022;97(1):57-67.
42. Chan JJL, Tran-Nhu L, Pitcairn CFM, Laverty AA, Mrejen M, Pescarini JM, Hone TV. Inequalities in the prevalence of cardiovascular disease risk factors in Brazilian slum populations: A cross-sectional study. PLOS Glob Public Health. 2022;2(9):e0000990.
43. Chang SC, Glymour M, Cornelis M, Walter S, Rimm EB, Tchetgen Tchetgen E, et al. Social Integration and Reduced Risk of Coronary Heart Disease in Women: The Role of Lifestyle Behaviors. Circ Res. 2017;120(12):1927-37.
44. Chatzi G, Mason T, Chandola T, Whittaker W, Howarth E, Cotterill S, et al. Sociodemographic disparities in non-diabetic hyperglycaemia and the transition to type 2 diabetes: evidence from the English Longitudinal Study of Ageing. Diabet Med. 2020;37(9):1536-44.
45. Cheruvalath H, Homa J, Singh M, Vilar P, Kassam A, Rovin RA. Associations Between Residential Greenspace, Socioeconomic Status, and Stroke: A Matched Case-Control Study. J Patient Cent Res Rev. 2022;9(2):89-97.
46. Child ST, Ruppel EH, Albert MA, Lawton L. Network Support and Negative Life Events Associated With Chronic Cardiometabolic Disease Outcomes. Am J Prev Med. 2022;62(1):e21-e8.
47. Christine PJ, Auchincloss AH, Bertoni AG, Carnethon MR, Sánchez BN, Moore K, et al. Longitudinal Associations Between Neighborhood Physical and Social Environments and Incident Type 2 Diabetes Mellitus: The Multi-Ethnic Study of Atherosclerosis (MESA). JAMA Intern Med. 2015;175(8):1311-20.
48. Chum A, O'Campo P. Cross-sectional associations between residential environmental exposures and cardiovascular diseases. BMC Public Health. 2015;15:438.
49. Clark CJ, Guo H, Lunos S, Aggarwal NT, Beck T, Evans DA, et al. Neighborhood cohesion is associated with reduced risk of stroke mortality. Stroke. 2011;42(5):1212-7.
50. Claudel SE, Adu-Brimpong J, Banks A, Ayers C, Albert MA, Das SR, et al. Association between neighborhood-level socioeconomic deprivation and incident hypertension: A longitudinal analysis of data from the Dallas heart study. Am Heart J. 2018;204:109-18.
51. Coelho DM, de Souza Andrade AC, Silva UM, Lazo M, Slesinski SC, Quistberg A, et al. Gender differences in the association of individual and contextual socioeconomic status with hypertension in 230 Latin American cities from the SALURBAL study: a multilevel analysis. BMC Public Health. 2023;23(1):1532.
52. Cofie LE, Hirth JM, Lee JGL. Social Support Networks and Foreign-Birth Status Associated With Obesity, Hypertension and Diabetes Prevalence Among 21-30 and 50-70 Year Old Adults Living in the San Francisco Bay Area. Am J Health Promot. 2021;35(8):1105-13.
53. Connolly V, Unwin N, Sherriff P, Bilous R, Kelly W. Diabetes prevalence and socioeconomic status: a population based study showing increased prevalence of type 2 diabetes mellitus in deprived areas. J Epidemiol Commun H. 2000;54(3):173-7.
54. Consolazio D, Koster A, Sarti S, Schram MT, Stehouwer CDA, Timmermans EJ, et al. Neighbourhood property value and type 2 diabetes mellitus in the Maastricht study: A multilevel study. PLoS One. 2020;15(6):e0234324.
55. Cookson R, Laudicella M, Donni PL. Measuring change in health care equity using small-area administrative data - evidence from the English NHS 2001-2008. Soc Sci Med. 2012;75(8):1514-22.
56. Cox M, Boyle PJ, Davey PG, Feng Z, Morris AD. Locality deprivation and Type 2 diabetes incidence: a local test of relative inequalities. Soc Sci Med. 2007;65(9):1953-64.
57. Cozier YC, Palmer JR, Horton NJ, Fredman L, Wise LA, Rosenberg L. Relation between neighborhood median housing value and hypertension risk among black women in the United States. Am J Public Health. 2007;97(4):718-24.
58. Cromer SJ, Lakhani CM, Mercader JM, Majarian TD, Schroeder P, Cole JB, et al. Association and Interaction of Genetics and Area-Level Socioeconomic Factors on the Prevalence of Type 2 Diabetes and Obesity. Diabetes Care. 2023;46(5):944-52.

59. Cubbin C, Sundquist K, Ahlén H, Johansson SE, Winkleby MA, Sundquist J. Neighborhood deprivation and cardiovascular disease risk factors: protective and harmful effects. Scand J Public Health. 2006;34(3):228-37.
60. Cunningham SA, Patel SA, Beckles GL, Geiss LS, Mehta N, Xie H, Imperatore G. County-level contextual factors associated with diabetes incidence in the United States. Ann Epidemiol. 2018;28(1):20-5.e2.
61. Cuthbertson CC, Heiss G, Wright JD, Camplain R, Patel MD, Foraker RE, et al. Socioeconomic status and access to care and the incidence of a heart failure diagnosis in the inpatient and outpatient settings. Ann Epidemiol. 2018;28(6):350-5.
62. de Oliveira FLP, Pimenta AM, Duncan BB, Griep RH, de Souza G, Barreto SM, Giatti L. Spatial clusters of diabetes: individual and neighborhood characteristics in the ELSA-Brasil cohort study. CADERNOS DE SAUDE PUBLICA. 2023;39(5).
63. De Silva SSA, Meeker MA, Ryan V, Algur Y, Long DL, Carson AP, et al. Comparing Global and Spatial Composite Measures of Neighborhood Socioeconomic Status Across US Counties. J Urban Health. 2022;99(3):457-68.
64. Desmond M, An WH. Neighborhood and Network Disadvantage among Urban Renters. Sociol Sci. 2015;2:329-49.
65. Diez Roux AV, Chambless L, Merkin SS, Arnett D, Eigenbrodt M, Nieto FJ, et al. Socioeconomic disadvantage and change in blood pressure associated with aging. Circulation. 2002;106(6):703-10.
66. Diez-Roux AV, Nieto FJ, Muntaner C, Tyroler HA, Comstock GW, Shahar E, et al. Neighborhood environments and coronary heart disease: a multilevel analysis. Am J Epidemiol. 1997;146(1):48-63.
67. Diez-Roux AV, Link BG, Northridge ME. A multilevel analysis of income inequality and cardiovascular disease risk factors. Soc Sci Med. 2000;50(5):673-87.
68. Djekic D, Angerås O, Lappas G, Fagman E, Fagerberg B, Bergström G, Rosengren A. Impact of socioeconomic status on coronary artery calcification. Eur J Prev Cardiol. 2018;25(16):1756-64.
69. Dragano N, Bobak M, Wege N, Peasey A, Verde PE, Kubinova R, et al. Neighbourhood socioeconomic status and cardiovascular risk factors: a multilevel analysis of nine cities in the Czech Republic and Germany. BMC Public Health. 2007;7:255.
70. Dubowitz T, Ghosh-Dastidar M, Eibner C, Slaughter ME, Fernandes M, Whitsel EA, et al. The Women's Health Initiative: The food environment, neighborhood socioeconomic status, BMI, and blood pressure. Obesity (Silver Spring). 2012;20(4):862-71.
71. Dwane N, Wabiri N, Manda S. Small-area variation of cardiovascular diseases and select risk factors and their association to household and area poverty in South Africa: Capturing emerging trends in South Africa to better target local level interventions. PLoS One. 2020;15(4):e0230564.
72. Dyck J, Tate R, Uhanova J, Torabi M. Social determinants and spatio-temporal variation of Ischemic Heart Disease in Manitoba. BMC Public Health. 2021;21(1):2325.
73. Ekholuenetale M, Barrow A. Prevalence and determinants of self-reported high blood pressure among women of reproductive age in Benin: a population-based study. Clin Hypertens. 2020;26:12.
74. Eng PM, Rimm EB, Fitzmaurice G, Kawachi I. Social ties and change in social ties in relation to subsequent total and cause-specific mortality and coronary heart disease incidence in men. Am J Epidemiol. 2002;155(8):700-9.
75. Engström G, Jerntorp I, Pessah-Rasmussen H, Hedblad B, Berglund G, Janzon L. Geographic distribution of stroke incidence within an urban population: Relations to socioeconomic circumstances and prevalence of cardiovascular risk factors. Stroke. 2001;32(5):1098-103.
76. Eschbach K, Ostir GV, Patel KV, Markides KS, Goodwin JS. Neighborhood context and mortality among older Mexican Americans: is there a barrio advantage? Am J Public Health. 2004;94(10):1807-12.
77. Essien UR, McCabe ME, Kershaw KN, Youmans QR, Fine MJ, Yancy CW, Khan SS. Association Between Neighborhood-Level Poverty and Incident Atrial Fibrillation: a Retrospective Cohort Study. J Gen Intern Med. 2022;37(6):1436-43.
78. Exeter DJ, Sabel CE, Hanham G, Lee AC, Wells S. Movers and stayers: The geography of residential mobility and CVD hospitalisations in Auckland, New Zealand. Soc Sci Med. 2015;133:331-9.

79. Faka A, Chalkias C, Montano D, Georgousopoulou EN, Tripitsidis A, Koloverou E, et al. Association of Socio-Environmental Determinants with Diabetes Prevalence in the Athens Metropolitan Area, Greece: A Spatial Analysis. Rev Diabet Stud. 2018;14(4):381-9.
80. Feero S, Hedges JR, Stevens P. Demographics of cardiac arrest: association with residence in a low-income area. Acad Emerg Med. 1995;2(1):11-6.
81. Ferguson TS, Younger-Coleman NOM, Mullings J, Francis D, Greene LG, Lyew-Ayee P, Wilks R. Neighbourhood socioeconomic characteristics and blood pressure among Jamaican youth: a pooled analysis of data from observational studies. PeerJ. 2020;8:e10058.
82. Fitzpatrick KM, Willis D. Chronic Disease, the Built Environment, and Unequal Health Risks in the 500 Largest U.S. Cities. Int J Environ Res Public Health. 2020;17(8).
83. Fleischer NL, Diez Roux AV, Alazraqui M, Spinelli H. Social patterning of chronic disease risk factors in a Latin American city. J Urban Health. 2008;85(6):923-37.
84. Ford ES, Loucks EB, Berkman LF. Social integration and concentrations of C-reactive protein among US adults. Ann Epidemiol. 2006;16(2):78-84.
85. Forsberg PO, Ohlsson H, Sundquist K. Causal nature of neighborhood deprivation on individual risk of coronary heart disease or ischemic stroke: A prospective national Swedish co-relative control study in men and women. Health Place. 2018;50:1-5.
86. Forsberg PO, Ohlsson H, Sundquist K. Workplace socioeconomic characteristics and coronary heart disease: a nationwide follow-up study. BMJ Open. 2023;13(7):e065285.
87. Freedman VA, Grafova IB, Rogowski J. Neighborhoods and chronic disease onset in later life. Am J Public Health. 2011;101(1):79-86.
88. Gao X, Kershaw KN, Barber S, Schreiner PJ, Do DP, Diez Roux AV, Mujahid MS. Associations Between Residential Segregation and Incident Hypertension: The Multi-Ethnic Study of Atherosclerosis. J Am Heart Assoc. 2022;11(3):e023084.
89. Garcia L, Lee A, Zeki Al Hazzouri A, Neuhaus J, Epstein M, Haan M. The Impact of Neighborhood Socioeconomic Position on Prevalence of Diabetes and Prediabetes in Older Latinos: The Sacramento Area Latino Study on Aging. Hisp Health Care Int. 2015;13(2):77-85.
90. Garcia L, Lee A, Zeki Al Hazzouri A, Neuhaus JM, Moyce S, Aiello A, et al. Influence of neighbourhood socioeconomic position on the transition to type II diabetes in older Mexican Americans: the Sacramento Area Longitudinal Study on Aging. BMJ Open. 2016;6(8):e010905.
91. Gary-Webb TL, Dyer H, McKenzie J, Younger-Coleman N, Tulloch-Reid M, Blake A, et al. Community stressors (violence, victimization, and neighborhood disorder) with cardiometabolic outcomes in urban Jamaica. Front Public Health. 2023;11:1130830.
92. Gaskin DJ, Thorpe RJ, Jr., McGinty EE, Bower K, Rohde C, Young JH, et al. Disparities in diabetes: the nexus of race, poverty, and place. Am J Public Health. 2014;104(11):2147-55.
93. Gebreab SY, Hickson DA, Sims M, Wyatt SB, Davis SK, Correa A, Diez-Roux AV. Neighborhood social and physical environments and type 2 diabetes mellitus in African Americans: The Jackson Heart Study. Health Place. 2017;43:128-37.
94. Gero K, Noubary F, Kawachi I, Baum CF, Wallace RB, Briesacher BA, Kim D. Associations of state-level and county-level hate crimes with individual-level cardiovascular risk factors in a prospective cohort study of middle-aged Americans: the National Longitudinal Survey of Youths 1979. BMJ Open. 2022;12(1):e054360.
95. Glover LM, Bertoni AG, Golden SH, Baltrus P, Min YI, Carnethon MR, et al. Sex differences in the association of psychosocial resources with prevalent type 2 diabetes among African Americans: The Jackson Heart Study. J Diabetes Complications. 2019;33(2):113-7.
96. Grundmann N, Mielck A, Siegel M, Maier W. Area deprivation and the prevalence of type 2 diabetes and obesity: analysis at the municipality level in Germany. BMC Public Health. 2014;14:1264.
97. Guion M, Mandereau-Bruno L, Goria S, Cosson E, Fosse-Edorh S. Eleven-year trends in socioeconomic inequalities in the prevalence and incidence of pharmacologically treated type 2 diabetes in France, 2010-2020. Diabetes Metab. 2024;50(2):101509.
98. Gwon JG, Choi J, Han YJ. Community-level socioeconomic inequality in the incidence of ischemic heart disease: a nationwide cohort study. BMC Cardiovasc Disord. 2020;20(1):87.
99. Halonen JI, Stenholm S, Pentti J, Kawachi I, Subramanian SV, Kivimäki M, Vahtera J. Childhood Psychosocial Adversity and Adult Neighborhood Disadvantage as Predictors of Cardiovascular Disease: A Cohort Study. Circulation. 2015;132(5):371-9.

100. Hamad R, Öztürk B, Foverskov E, Pedersen L, Sørensen HT, Bøtker HE, White JS. Association of Neighborhood Disadvantage With Cardiovascular Risk Factors and Events Among Refugees in Denmark. JAMA Netw Open. 2020;3(8):e2014196.
101. Hanefeld C, Haschemi A, Lampert T, Trampisch HJ, Mügge A, Miebach J, et al. Social Gradients in Myocardial Infarction and Stroke Diagnoses in Emergency Medicine. Dtsch Arztebl Int. 2018;115(4):41-8.
102. Hanigan IC, Cochrane T, Davey R. Impact of scale of aggregation on associations of cardiovascular hospitalization and socio-economic disadvantage. PLoS One. 2017;12(11):e0188161.
103. Harding BN, Hawley CN, Kalinowski J, Sims M, Muntner P, Young Mielcarek BA, et al. Relationship between social support and incident hypertension in the Jackson Heart Study: a cohort study. BMJ Open. 2022;12(3):e054812.
104. Hashemi SJ, Jasemzadeh M, Saki N, Cheraghian B, Sarvandian S, Montazeri A, et al. Social determinants of health and diabetes: Results from a cohort study in Iran. ASIAN JOURNAL OF SOCIAL HEALTH AND BEHAVIOR. 2023;6(2):86-91.
105. Hassen HY, Bastiaens H, Van Royen K, Abrams S. Socioeconomic and behavioral determinants of cardiovascular diseases among older adults in Belgium and France: A longitudinal analysis from the SHARE study. PLoS One. 2020;15(12):e0243422.
106. Hawkins NM, Scholes S, Bajekal M, Love H, O'Flaherty M, Raine R, Capewell S. Community care in England: reducing socioeconomic inequalities in heart failure. Circulation. 2012;126(9):1050-7.
107. Heeley EL, Wei JW, Carter K, Islam MS, Thrift AG, Hankey GJ, et al. Socioeconomic disparities in stroke rates and outcome: pooled analysis of stroke incidence studies in Australia and New Zealand. Med J Aust. 2011;195(1):10-4.
108. Hendryx M, Nicholson W, Manson JE, Kroenke CH, Lee J, Weitlauf JC, et al. Social Relationships and Risk of Type 2 Diabetes Among Postmenopausal Women. J Gerontol B Psychol Sci Soc Sci. 2020;75(7):1597-608.
109. Henriksson G, Weitoft GR, Allebeck P. Associations between income inequality at municipality level and health depend on context - a multilevel analysis on myocardial infarction in Sweden. Soc Sci Med. 2010;71(6):1141-9.
110. Heredia NI, Xu T, Lee M, McNeill LH, Reininger BM. The Neighborhood Environment and Hispanic/Latino Health. Am J Health Promot. 2022;36(1):38-45.
111. Herrera-Añazco P, Amaya E, Atamari-Anahui N, Ccorahua-Rios M, Hernandez AV. Association between social determinants of health and trends in prevalence of hypertension in patients of the Peruvian Ministry of Health. Trop Med Int Health. 2019;24(12):1434-41.
112. Herrick CJ, Yount BW, Eyler AA. Implications of supermarket access, neighbourhood walkability and poverty rates for diabetes risk in an employee population. Public Health Nutr. 2016;19(11):2040-8.
113. Hilding A, Shen C, Östenson CG. Social network and development of prediabetes and type 2 diabetes in middle-aged Swedish women and men. Diabetes Res Clin Pract. 2015;107(1):166-77.
114. Hill PL, Weston SJ, Jackson JJ. Connecting social environment variables to the onset of major specific health outcomes. Psychol Health. 2014;29(7):753-67.
115. Höfelmann DA, Antunes JL, Santos Silva DA, Peres MA. Is income area level associated with blood pressure in adults regardless of individual-level characteristics? A multilevel approach. Health Place. 2012;18(5):971-7.
116. Holstiege J, Akmatov MK, Störk S, Steffen A, Bätzing J. Higher prevalence of heart failure in rural regions: a population-based study covering 87% of German inhabitants. Clin Res Cardiol. 2019;108(10):1102-6.
117. Holtgrave DR, Crosby R. Is social capital a protective factor against obesity and diabetes? Findings from an exploratory study. Ann Epidemiol. 2006;16(5):406-8.
118. Honda Y, Mok Y, Mathews L, Hof JRV, Daumit G, Kucharska-Newton A, et al. Psychosocial factors and subsequent risk of hospitalizations with peripheral artery disease: The Atherosclerosis Risk in Communities (ARIC) Study. Atherosclerosis. 2021;329:36-43.
119. Honjo K, Iso H, Nakaya T, Hanibuchi T, Ikeda A, Inoue M, et al. Impact of neighborhood socioeconomic conditions on the risk of stroke in Japan. J Epidemiol. 2015;25(3):254-60.

120. Horsten M, Mittleman MA, Wamala SP, Schenck-Gustafsson K, Orth-Gomér K. Social relations and the metabolic syndrome in middle-aged Swedish women. J Cardiovasc Risk. 1999;6(6):391-7.
121. Hosseini Z, Veenstra G, Khan NA, Conklin AI. Social connections and hypertension in women and men: a population-based cross-sectional study of the Canadian Longitudinal Study on Aging. J Hypertens. 2020.
122. Howard VJ, McClure LA, Kleindorfer DO, Cunningham SA, Thrift AG, Diez Roux AV, Howard G. Neighborhood socioeconomic index and stroke incidence in a national cohort of blacks and whites. Neurology. 2016;87(22):2340-7.
123. Hu L, Ji J, Li Y, Liu B, Zhang Y. Quantile Regression Forests to Identify Determinants of Neighborhood Stroke Prevalence in 500 Cities in the USA: Implications for Neighborhoods with High Prevalence. J Urban Health. 2020.
124. Hu MD, Lawrence KG, Bodkin MR, Kwok RK, Engel LS, Sandler DP. Neighborhood Deprivation, Obesity, and Diabetes in Residents of the US Gulf Coast. Am J Epidemiol. 2021;190(2):295-304.
125. Huang H. Moderating Effects of Racial Segregation on the Associations of Cardiovascular Outcomes with Walkability in Chicago Metropolitan Area. Int J Environ Res Public Health. 2022;19(21).
126. Hwang SE, Choi S, Kim K, Lee JK, Oh J, Park SM. Association between social trust and the risk of cardiovascular disease in older adults in Korea: a nationwide retrospective cohort study. BMC Public Health. 2020;20(1):1844.
127. Jack E, Lee D, Dean N. Estimating the changing nature of Scotland's health inequalities by using a multivariate spatiotemporal model. J R Stat Soc Ser A Stat Soc. 2019;182(3):1061-80.
128. Jackson CH, Richardson S, Best NG. Studying place effects on health by synthesising individual and area-level outcomes. Soc Sci Med. 2008;67(12):1995-2006.
129. Jadow BM, Hu L, Zou J, Labovitz D, Ibeh C, Ovbiagele B, Esenwa C. Historical Redlining, Social Determinants of Health, and Stroke Prevalence in Communities in New York City. JAMA Netw Open. 2023;6(4):e235875.
130. Jain V, Al Rifai M, Khan SU, Kalra A, Rodriguez F, Samad Z, et al. Association Between Social Vulnerability Index and Cardiovascular Disease: A Behavioral Risk Factor Surveillance System Study. J Am Heart Assoc. 2022;11(15):e024414.
131. Jensen NK, Frøslev T, Foverskov E, Glymour M, Sørensen HT, Hamad R. The association of neighborhood socioeconomic characteristics with cardiovascular health: A quasi-experimental study of refugees to Denmark. Health Place. 2023;84:103128.
132. Jonsson M, Ljungman P, Härkönen J, Van Nieuwenhuizen B, Møller S, Ringh M, Nordberg P. Relationship between socioeconomic status and incidence of out-of-hospital cardiac arrest is dependent on age. J Epidemiol Community Health. 2020;74(9):726-31.
133. Jung L, De Neve JW, Chen S, Manne-Goehler J, Jaacks LM, Corsi DJ, et al. Nationally representative household survey data for studying the interaction between district-level development and individual-level socioeconomic gradients of cardiovascular disease risk factors in India. Data Brief. 2019;27:104486.
134. Kaiser P, Diez Roux AV, Mujahid M, Carnethon M, Bertoni A, Adar SD, et al. Neighborhood Environments and Incident Hypertension in the Multi-Ethnic Study of Atherosclerosis. Am J Epidemiol. 2016;183(11):988-97.
135. Kakinami L, Serbin LA, Stack DM, Karmaker SC, Ledingham JE, Schwartzman AE. Neighbourhood disadvantage and behavioural problems during childhood and the risk of cardiovascular disease risk factors and events from a prospective cohort. Prev Med Rep. 2017;8:294-300.
136. Kauhl B, Maier W, Schweikart J, Keste A, Moskwyn M. Exploring the small-scale spatial distribution of hypertension and its association to area deprivation based on health insurance claims in Northeastern Germany. BMC Public Health. 2018;18(1).
137. Kawachi I, Colditz GA, Ascherio A, Rimm EB, Giovannucci E, Stampfer MJ, Willett WC. A prospective study of social networks in relation to total mortality and cardiovascular disease in men in the USA. J Epidemiol Community Health. 1996;50(3):245-51.
138. Keita AD, Judd SE, Howard VJ, Carson AP, Ard JD, Fernandez JR. Associations of neighborhood area level deprivation with the metabolic syndrome and inflammation among middle- and older- age adults. BMC Public Health. 2014;14:1319.

139. Kelli HM, Hammadah M, Ahmed H, Ko YA, Topel M, Samman-Tahhan A, et al. Association Between Living in Food Deserts and Cardiovascular Risk. Circ Cardiovasc Qual Outcomes. 2017;10(9).
140. Kershaw KN, Diez Roux AV, Burgard SA, Lisabeth LD, Mujahid MS, Schulz AJ. Metropolitan-level racial residential segregation and black-white disparities in hypertension. Am J Epidemiol. 2011;174(5):537-45.
141. Kim D, Glazier RH, Zagorski B, Kawachi I, Oreopoulos P. Neighbourhood socioeconomic position and risks of major chronic diseases and all-cause mortality: a quasi-experimental study. BMJ Open. 2018;8(5):e018793.
142. Kim MH, Schwartz GL, White JS, Glymour MM, Reardon SF, Kershaw KN, et al. School racial segregation and long-term cardiovascular health among Black adults in the US: A quasi-experimental study. PLoS Med. 2022;19(6):e1004031.
143. Kim Y, Lee A, Cubbin C. Effect of Social Environments on Cardiovascular Disease in the United States. J Am Heart Assoc. 2022;11(20):e025923.
144. Kivimäki M, Vahtera J, Tabák AG, Halonen JI, Vineis P, Pentti J, et al. Neighbourhood socioeconomic disadvantage, risk factors, and diabetes from childhood to middle age in the Young Finns Study: a cohort study. Lancet Public Health. 2018;3(8):e365-e73.
145. Kivimäki M, Batty GD, Pentti J, Nyberg ST, Lindbohm JV, Ervasti J, et al. Modifications to residential neighbourhood characteristics and risk of 79 common health conditions: a prospective cohort study. Lancet Public Health. 2021;6(6):e396-e407.
146. Kling JR, Liebman JB, Katz LF. Experimental analysis of neighborhood effects. Econometrica. 2007;75(1):83-119.
147. Kolpak P, Wang L. Exploring the social and neighbourhood predictors of diabetes: a comparison between Toronto and Chicago. Prim Health Care Res Dev. 2017;18(3):291-9.
148. Krieger N. Overcoming the absence of socioeconomic data in medical records: validation and application of a census-based methodology. Am J Public Health. 1992;82(5):703-10.
149. Krishnan S, Cozier YC, Rosenberg L, Palmer JR. Socioeconomic status and incidence of type 2 diabetes: results from the Black Women's Health Study. Am J Epidemiol. 2010;171(5):564-70.
150. Kwok MK, Kawachi I, Rehkopf D, Ni MY, Leung GM, Schooling CM. Relative Deprivation, Income Inequality, and Cardiovascular Health: Observational and Mendelian Randomization Studies in Hong Kong Chinese. Front Public Health. 2021;9:726617.
151. Lachkhem Y, Minvielle É, Rican S. Geographic Variations of Stroke Hospitalization across France: A Diachronic Cluster Analysis. Stroke Res Treat. 2018;2018:1897569.
152. Lagisetty PA, Wen M, Choi H, Heisler M, Kanaya AM, Kandula NR. Neighborhood Social Cohesion and Prevalence of Hypertension and Diabetes in a South Asian Population. J Immigr Minor Health. 2016;18(6):1309-16.
153. Larrañaga I, Arteagoitia JM, Rodriguez JL, Gonzalez F, Esnaola S, Piniés JA. Socio-economic inequalities in the prevalence of Type 2 diabetes, cardiovascular risk factors and chronic diabetic complications in the Basque Country, Spain. Diabet Med. 2005;22(8):1047-53.
154. Laursen KR, Hulman A, Witte DR, Terkildsen Maindal H. Social relations, depressive symptoms, and incident type 2 diabetes mellitus: The English Longitudinal Study of Ageing. Diabetes Res Clin Pract. 2017;126:86-94.
155. Lawlor DA, Davey Smith G, Patel R, Ebrahim S. Life-course socioeconomic position, area deprivation, and coronary heart disease: findings from the British Women's Heart and Health Study. Am J Public Health. 2005;95(1):91-7.
156. Lee DC, Gallagher MP, Gopalan A, Osorio M, Vinson AJ, Wall SP, et al. Identifying Geographic Disparities in Diabetes Prevalence Among Adults and Children Using Emergency Claims Data. J Endocr Soc. 2018;2(5):460-70.
157. Lee J, Bahk J, Kim I, Kim YY, Yun SC, Kang HY, et al. Geographic Variation in Morbidity and Mortality of Cerebrovascular Diseases in Korea during 2011-2015. J Stroke Cerebrovasc Dis. 2018;27(3):747-57.
158. Lemstra M, Neudorf C, Opondo J. Health disparity by neighbourhood income. Can J Public Health. 2006;97(6):435-9.
159. Leyland AH. Socioeconomic gradients in the prevalence of cardiovascular disease in Scotland: the roles of composition and context. J Epidemiol Community Health. 2005;59(9):799-803.

160. Li K, Wen M, Henry KA. Ethnic density, immigrant enclaves, and Latino health risks: A propensity score matching approach. Soc Sci Med. 2017;189:44-52.
161. Linde S, Egede LE. Community Social Capital and Population Health Outcomes. JAMA Netw Open. 2023;6(8):e2331087.
162. Ling DC. Do the Chinese "Keep up with the Jones"?: Implications of peer effects, growing economic disparities and relative deprivation on health outcomes among older adults in China. China Econ Rev. 2009;20(1):65-81.
163. Lippert AM, Evans CR, Razak F, Subramanian SV. Associations of Continuity and Change in Early Neighborhood Poverty With Adult Cardiometabolic Biomarkers in the United States: Results From the National Longitudinal Study of Adolescent to Adult Health, 1995-2008. Am J Epidemiol. 2017;185(9):765-76.
164. Lönn SL, Melander O, Crump C, Sundquist K. Accumulated neighbourhood deprivation and coronary heart disease: a nationwide cohort study from Sweden. BMJ Open. 2019;9(9):e029248.
165. Loucks EB, Sullivan LM, D'Agostino RB, Sr., Larson MG, Berkman LF, Benjamin EJ. Social networks and inflammatory markers in the Framingham Heart Study. J Biosoc Sci. 2006;38(6):835-42.
166. Lu X, Juon HS, He X, Dallal CM, Wang MQ, Lee S. The Association Between Perceived Stress and Hypertension Among Asian Americans: Does Social Support and Social Network Make a Difference? J Community Health. 2019;44(3):451-62.
167. Ludwig J, Sanbonmatsu L, Gennetian L, Adam E, Duncan GJ, Katz LF, et al. Neighborhoods, obesity, and diabetes--a randomized social experiment. N Engl J Med. 2011;365(16):1509-19.
168. Lukachko A, Hatzenbuehler ML, Keyes KM. Structural racism and myocardial infarction in the United States. Soc Sci Med. 2014;103:42-50.
169. Lukaschek K, Baumert J, Kruse J, Meisinger C, Ladwig KH. Sex differences in the association of social network satisfaction and the risk for type 2 diabetes. BMC Public Health. 2017;17(1):379.
170. Lund R, Rod NH, Christensen U. Are negative aspects of social relations predictive of angina pectoris? A 6-year follow-up study of middle-aged Danish women and men. J Epidemiol Community Health. 2012;66(4):359-65.
171. Lund R, Rod NH, Thielen K, Nilsson CJ, Christensen U. Negative aspects of close social relations and 10-year incident ischaemic heart disease hospitalization among middle-aged Danes. Eur J Prev Cardiol. 2014;21(10):1249-56.
172. Ma R, Romano E, Vancampfort D, Firth J, Stubbs B, Koyanagi A. Physical Multimorbidity and Social Participation in Adult Aged 65 Years and Older From Six Low- and Middle-Income Countries. J Gerontol B Psychol Sci Soc Sci. 2021;76(7):1452-62.
173. Madela S, Harriman NW, Sewpaul R, Mbewu AD, Williams DR, Sifunda S, et al. Individual and area-level socioeconomic correlates of hypertension prevalence, awareness, treatment, and control in uMgungundlovu, KwaZulu-Natal, South Africa. BMC Public Health. 2023;23(1):417.
174. Madela SLM, Harriman NW, Sewpaul R, Mbewu AD, Williams DR, Sifunda S, et al. Area-level deprivation and individual-level socioeconomic correlates of the diabetes care cascade among black south africans in uMgungundlovu, KwaZulu-Natal, South Africa. PLoS One. 2023;18(12):e0293250.
175. Maheswaran R, Chan D, Fryers PT, McManus C, McCabe H. Socio-economic deprivation and excess winter mortality and emergency hospital admissions in the South Yorkshire Coalfields Health Action Zone, UK. Public Health. 2004;118(3):167-76.
176. Maier W, Scheidt-Nave C, Holle R, Kroll LE, Lampert T, Du Y, et al. Area Level Deprivation Is an Independent Determinant of Prevalent Type 2 Diabetes and Obesity at the National Level in Germany. Results from the National Telephone Health Interview Surveys 'German Health Update' GEDA 2009 and 2010. Plos One. 2014;9(2).
177. Malino C, Kershaw T, Angley M, Frederic R, Small M. Social capital and hypertension in rural Haitian women. Matern Child Health J. 2014;18(10):2253-60.
178. Marley TL, Metzger MW. A longitudinal study of structural risk factors for obesity and diabetes among American Indian young adults, 1994-2008. Prev Chronic Dis. 2015;12:E69.
179. Massa KH, Pabayo R, Lebrão ML, Chiavegatto Filho AD. Environmental factors and cardiovascular diseases: the association of income inequality and green spaces in elderly residents of São Paulo, Brazil. BMJ Open. 2016;6(9):e011850.
180. Matheson FI, White HL, Moineddin R, Dunn JR, Glazier RH. Neighbourhood chronic stress and gender inequalities in hypertension among Canadian adults: a multilevel analysis. J Epidemiol Community Health. 2010;64(8):705-13.

181. Matthew P, Brodersen DM. Income inequality and health outcomes in the United States: An empirical analysis. Soc Sci J. 2018;55(4):432-42.
182. Mayne SL, Loizzo L, Bancks MP, Carnethon MR, Barber S, Gordon-Larsen P, et al. Racial residential segregation, racial discrimination, and diabetes: The Coronary Artery Risk Development in Young Adults study. Health Place. 2020;62:102286.
183. McDoom MM, Palta P, Vart P, Juraschek SP, Kucharska-Newton A, Diez Roux AV, Coresh J. Late life socioeconomic status and hypertension in an aging cohort: the Atherosclerosis Risk in Communities Study. J Hypertens. 2018;36(6):1382-90.
184. Menec VH, Shooshtari S, Nowicki S, Fournier S. Does the relationship between neighborhood socioeconomic status and health outcomes persist into very old age? A population-based study. J Aging Health. 2010;22(1):27-47.
185. Mentias A, Mujahid MS, Sumarsono A, Nelson RK, Madron JM, Powell-Wiley TM, et al. Historical Redlining, Socioeconomic Distress, and Risk of Heart Failure Among Medicare Beneficiaries. Circulation. 2023;148(3):210-9.
186. Metcalf PA, Scragg RR, Schaaf D, Dyall L, Black PN, Jackson RT. Comparison of different markers of socioeconomic status with cardiovascular disease and diabetes risk factors in the Diabetes, Heart and Health Survey. N Z Med J. 2008;121(1269):45-56.
187. Mezuk B, Cederin K, Li X, Rice K, Kendler KS, Sundquist J, Sundquist K. Immigrant enclaves and risk of diabetes: a prospective study. BMC Public Health. 2014;14:1093.
188. Mohottige D, Davenport CA, Bhavsar N, Schappe T, Lyn MJ, Maxson P, et al. Residential Structural Racism and Prevalence of Chronic Health Conditions. JAMA Netw Open. 2023;6(12):e2348914.
189. Moore S, Stewart S, Teixeira A. Decomposing social capital inequalities in health. J Epidemiol Community Health. 2014;68(3):233-8.
190. Morenoff JD, House JS, Hansen BB, Williams DR, Kaplan GA, Hunte HE. Understanding social disparities in hypertension prevalence, awareness, treatment, and control: the role of neighborhood context. Soc Sci Med. 2007;65(9):1853-66.
191. Morris RW, Wannamethee G, Lennon LT, Thomas MC, Whincup PH. Do socioeconomic characteristics of neighbourhood of residence independently influence incidence of coronary heart disease and all-cause mortality in older British men? Eur J Cardiovasc Prev Rehabil. 2008;15(1):19-25.
192. Mujahid MS, Diez Roux AV, Morenoff JD, Raghunathan TE, Cooper RS, Ni H, Shea S. Neighborhood characteristics and hypertension. Epidemiology. 2008;19(4):590-8.
193. Müller G, Hartwig S, Greiser KH, Moebus S, Pundt N, Schipf S, et al. Gender differences in the association of individual social class and neighbourhood unemployment rate with prevalent type 2 diabetes mellitus: a cross-sectional study from the DIAB-CORE consortium. BMJ Open. 2013;3(6).
194. Müller G, Kluttig A, Greiser KH, Moebus S, Slomiany U, Schipf S, et al. Regional and neighborhood disparities in the odds of type 2 diabetes: results from 5 population-based studies in Germany (DIAB-CORE consortium). Am J Epidemiol. 2013;178(2):221-30.
195. Murray ET, Diez Roux AV, Carnethon M, Lutsey PL, Ni H, O'Meara ES. Trajectories of neighborhood poverty and associations with subclinical atherosclerosis and associated risk factors: the multi-ethnic study of atherosclerosis. Am J Epidemiol. 2010;171(10):1099-108.
196. Nakagomi A, Tsuji T, Hanazato M, Kobayashi Y, Kondo K. Association Between Community-Level Social Participation and Self-reported Hypertension in Older Japanese: A JAGES Multilevel Cross-sectional Study. Am J Hypertens. 2019;32(5):503-14.
197. Nazmi A, Diez Roux A, Ranjit N, Seeman TE, Jenny NS. Cross-sectional and longitudinal associations of neighborhood characteristics with inflammatory markers: findings from the multi-ethnic study of atherosclerosis. Health Place. 2010;16(6):1104-12.
198. Neufcourt L, Deguen S, Bayat S, Paillard F, Zins M, Grimaud O. Geographical variations in the prevalence of hypertension in France: Cross-sectional analysis of the CONSTANCES cohort. Eur J Prev Cardiol. 2019;26(12):1242-51.
199. Nikulina V, Widom CS. Do race, neglect, and childhood poverty predict physical health in adulthood? A multilevel prospective analysis. Child Abuse Negl. 2014;38(3):414-24.
200. Odoi EW, Nagle N, Zaretzki R, Jordan M, DuClos C, Kintziger KW. Sociodemographic Determinants of Acute Myocardial Infarction Hospitalization Risks in Florida. J Am Heart Assoc. 2020;9(11):e012712.

201. Ogungbe O, Turkson-Ocran RA, Nkimbeng M, Cudjoe J, Miller HN, Baptiste D, et al. Social determinants of hypertension and diabetes among African immigrants: the African immigrants health study. Ethn Health. 2021:1-13.
202. Ohanyan H, Portengen L, Kaplani O, Huss A, Hoek G, Beulens JWJ, et al. Associations between the urban exposome and type 2 diabetes: Results from penalised regression by least absolute shrinkage and selection operator and random forest models. Environ Int. 2022;170:107592.
203. Oktamianti P, Kusuma D, Amir V, Tjandrarini DH, Paramita A. District-Level Inequalities in Hypertension among Adults in Indonesia: A Cross-Sectional Analysis by Sex and Age Group. Int J Environ Res Public Health. 2022;19(20).
204. Oladele CR, Thompson TA, Wang K, Galusha D, Tran E, Martinez-Brockman JL, et al. Egocentric Health Networks and Cardiovascular Risk Factors in the ECHORN Cohort Study. J Gen Intern Med. 2020;35(3):784-91.
205. Omariba DW, Ross NA, Sanmartin C, Tu JV. Neighbourhood immigrant concentration and hospitalization: a multilevel analysis of cardiovascular-related admissions in Ontario using linked data. Can J Public Health. 2014;105(6):e404-11.
206. Osborn B, Morey BN, Billimek J, Ro A. Food Insecurity and Type 2 Diabetes Among Latinos: Examining Neighborhood Cohesion as a Protective Factor. J Racial Ethn Health Disparities. 2023;10(4):2061-70.
207. Pantell MS, Prather AA, Downing JM, Gordon NP, Adler NE. Association of Social and Behavioral Risk Factors With Earlier Onset of Adult Hypertension and Diabetes. JAMA Netw Open. 2019;2(5):e193933.
208. Penninx BW, van Tilburg T, Kriegsman DM, Boeke AJ, Deeg DJ, van Eijk JT. Social network, social support, and loneliness in older persons with different chronic diseases. J Aging Health. 1999;11(2):151-68.
209. Pichora E, Polsky JY, Catley C, Perumal N, Jin J, Allin S. Comparing individual and area-based income measures: impact on analysis of inequality in smoking, obesity, and diabetes rates in Canadians 2003-2013. Can J Public Health-Rev Can Sante Publ. 2018;109(3):410-8.
210. Pinheiro LC, Reshetnyak E, Sterling MR, Levitan EB, Safford MM, Goyal P. Multiple Vulnerabilities to Health Disparities and Incident Heart Failure Hospitalization in the REGARDS Study. Circ Cardiovasc Qual Outcomes. 2020;13(8):e006438.
211. Piwońska AM, Piwoński J, Cicha-Mikołajczyk A, Kozela M, Pająk A, Śmigielski W, et al. Identifying associations between the social network index, its components, and the prevalence of cardiovascular diseases in Polish adults. Results of the cross-sectional WOBASZ II study. Kardiol Pol. 2023;81(12):1237-46.
212. Ptushkina V, Seidel-Jacobs E, Maier W, Schipf S, Völzke H, Markus MRP, et al. Educational Level, but Not Income or Area Deprivation, is Related to Macrovascular Disease: Results From Two Population-Based Cohorts in Germany. Int J Public Health. 2021;66:633909.
213. Quashie NT, García C, Meltzer G, Andrade FCD, Matos-Moreno A. Neighborhood socioeconomic position, living arrangements, and cardiometabolic disease among older Puerto Ricans: An examination using PREHCO 2002-2007. PLoS One. 2023;18(8):e0289170.
214. Quiñones S, Goyal A, Ahmed ZU. Geographically weighted machine learning model for untangling spatial heterogeneity of type 2 diabetes mellitus (T2D) prevalence in the USA. Sci Rep. 2021;11(1):6955.
215. Rachele JN, Giles-Corti B, Turrell G. Neighbourhood disadvantage and self-reported type 2 diabetes, heart disease and comorbidity: a cross-sectional multilevel study. Ann Epidemiol. 2016;26(2):146-50.
216. Redondo-Sendino A, Guallar-Castillón P, Banegas JR, Rodríguez-Artalejo F. [Relationship between social network and hypertension in older people in Spain]. Rev Esp Cardiol. 2005;58(11):1294-301.
217. Rethy LB, McCabe ME, Kershaw KN, Ahmad FS, Lagu T, Pool LR, Khan SS. Neighborhood Poverty and Incident Heart Failure: an Analysis of Electronic Health Records from 2005 to 2018. J Gen Intern Med. 2021;36(12):3719-27.
218. Riddell T. Heart failure hospitalisations and deaths in New Zealand: patterns by deprivation and ethnicity. N Z Med J. 2004;118(1208):U1254.
219. Rod NH, Andersen I, Prescott E. Psychosocial risk factors and heart failure hospitalization: a prospective cohort study. Am J Epidemiol. 2011;174(6):672-80.
220. Rose KM, Suchindran CM, Foraker RE, Whitsel EA, Rosamond WD, Heiss G, Wood JL. Neighborhood disparities in incident hospitalized myocardial infarction in four U.S. communities: the ARIC surveillance study. Ann Epidemiol. 2009;19(12):867-74.

221. Safford MM, Reshetnyak E, Sterling MR, Richman JS, Muntner PM, Durant RW, et al. Number of Social Determinants of Health and Fatal and Nonfatal Incident Coronary Heart Disease in the REGARDS Study. Circulation. 2021;143(3):244-53.
222. Salinas J, Beiser A, Himali JJ, Satizabal CL, Aparicio HJ, Weinstein G, et al. Associations between social relationship measures, serum brain-derived neurotrophic factor, and risk of stroke and dementia. Alzheimers Dement (N Y). 2017;3(2):229-37.
223. Samuel LJ, Thorpe RJ, Jr., Bower KM, LaVeist TA. Community Characteristics are Associated with Blood Pressure Levels in a Racially Integrated Community. J Urban Health. 2015;92(3):403-14.
224. Savin KL, Roesch SC, Oren E, Carlson JA, Allison MA, Sotres-Alvarez D, et al. Social and built neighborhood environments and blood pressure 6 years later: Results from the Hispanic Community Health Study/Study of Latinos and the SOL CASAS ancillary study. Soc Sci Med. 2022;292:114496.
225. Schieb LJ, Mobley LR, George M, Casper M. Tracking stroke hospitalization clusters over time and associations with county-level socioeconomic and healthcare characteristics. Stroke. 2013;44(1):146-52.
226. Schootman M, Andresen EM, Wolinsky FD, Malmstrom TK, Miller JP, Yan Y, Miller DK. The effect of adverse housing and neighborhood conditions on the development of diabetes mellitus among middle-aged African Americans. American Journal of Epidemiology. 2007;166(4):379-87.
227. Schwartz BS, Pollak J, Poulsen MN, Bandeen-Roche K, Moon K, DeWalle J, et al. Association of community types and features in a case-control analysis of new onset type 2 diabetes across a diverse geography in Pennsylvania. BMJ Open. 2021;11(1):e043528.
228. Schwartz BS, Kolak M, Pollak JS, Poulsen MN, Bandeen-Roche K, Moon KA, et al. Associations of four indexes of social determinants of health and two community typologies with new onset type 2 diabetes across a diverse geography in Pennsylvania. PLoS One. 2022;17(9):e0274758.
229. Sharma I, Karen Campbell M, Choi YH, Luginaah I, Were JM, Vargas-Gonzalea JC, Stranges S. Does the place of residence influence your risk of being hypertensive? A study-based on Nepal Demographic and Health Survey. Hypertens Res. 2023;46(6):1363-74.
230. Sharp G, Carpiano RM. Neighborhood social organization exposures and racial/ethnic disparities in hypertension risk in Los Angeles. PLoS One. 2023;18(3):e0282648.
231. Sheets L, Petroski GF, Jaddoo J, Barnett Y, Barnett C, Kelley LEH, et al. The Effect of Neighborhood Disadvantage on Diabetes Prevalence. AMIA Annu Symp Proc. 2017;2017:1547-53.
232. Shibayama T, Noguchi H, Takahashi H, Tamiya N. Relationship between social engagement and diabetes incidence in a middle-aged population: Results from a longitudinal nationwide survey in Japan. J Diabetes Investig. 2018;9(5):1060-6.
233. Siegel M, Mielck A, Maier W. Individual Income, Area Deprivation, and Health: Do Income-Related Health Inequalities Vary by Small Area Deprivation? Health Econ. 2015;24(11):1523-30.
234. Singh S, Zhou R, Li X, Tong LP. The complex relationship with health: Rural and urban "poor' women. Int Soc Work. 2016;59(1):32-46.
235. Smith GD, Hart C, Watt G, Hole D, Hawthorne V. Individual social class, area-based deprivation, cardiovascular disease risk factors, and mortality: the Renfrew and Paisley Study. J Epidemiol Community Health. 1998;52(6):399-405.
236. Smurthwaite K, Bagheri N. Using Geographical Convergence of Obesity, Cardiovascular Disease, and Type 2 Diabetes at the Neighborhood Level to Inform Policy and Practice. Prev Chronic Dis. 2017;14:E91.
237. Spicer J, Jackson R, Scragg R. The Effects of Anger Management and Social Contact on Risk of Myocardial-Infarction in Type-as and Type-Bs. Psychol Health. 1993;8(4):243-55.
238. Splan ED, Magerman AB, Forbes CE. Associations of regional racial attitudes with chronic illness in the United States. Soc Sci Med. 2021;281:114077.
239. Steckel RH. The hidden cost of moving up: type 2 diabetes and the escape from persistent poverty in the American South. Am J Hum Biol. 2013;25(4):508-15.
240. Suchy-Dicey A, Eyituoyo H, O'Leary M, Cole SA, Traore A, Verney S, et al. Psychological and social support associations with mortality and cardiovascular disease in middle-aged American Indians: the Strong Heart Study. Soc Psychiatry Psychiatr Epidemiol. 2022;57(7):1421-33.

241. Sun WJ, Gong F, Xu J. Individual and contextual correlates of cardiovascular diseases among adults in the United States: a geospatial and multilevel analysis. GeoJournal. 2020;85(6):1685-700.
242. Sundquist K, Malmström M, Johansson SE. Neighbourhood deprivation and incidence of coronary heart disease: a multilevel study of 2.6 million women and men in Sweden. J Epidemiol Community Health. 2004;58(1):71-7.
243. Swain PK, Behera B, Das D. Association between Area-Level Socio-Economic Status and Hypertension in Eag States of India : An Insight from Nfhs-Iv 2015-16. Int J Agric Stat Sci. 2019;15(1):39-52.
244. Tang X, Laskowitz DT, He L, Østbye T, Bettger JP, Cao Y, et al. Neighborhood socioeconomic status and the prevalence of stroke and coronary heart disease in rural China: a population-based study. Int J Stroke. 2015;10(3):388-95.
245. Tapager I, Bender AM, Andersen I. A decade of socioeconomic inequality in type 2 diabetes area-level prevalence: an unshakeable status quo? Scand J Public Health. 2023;51(2):268-74.
246. Terashima M, Rainham DG, Levy AR. A small-area analysis of inequalities in chronic disease prevalence across urban and non-urban communities in the Province of Nova Scotia, Canada, 2007-2011. BMJ Open. 2014;4(5):e004459.
247. Thrift AG, Dewey HM, Sturm JW, Paul SL, Gilligan AK, Srikanth VK, et al. Greater incidence of both fatal and nonfatal strokes in disadvantaged areas: the Northeast Melbourne Stroke Incidence Study. Stroke. 2006;37(3):877-82.
248. Tompkins JW, Luginaah IN, Booth GL, Harris SB. The geography of diabetes in London, Canada: the need for local level policy for prevention and management. Int J Environ Res Public Health. 2010;7(5):2407-22.
249. Trifan G, Gallo LC, Lamar M, Garcia-Bedoya O, Perreira KM, Pirzada A, et al. Association of Unfavorable Social Determinants of Health With Stroke/Transient Ischemic Attack and Vascular Risk Factors in Hispanic/Latino Adults: Results From Hispanic Community Health Study/Study of Latinos. J Stroke. 2023;25(3):361-70.
250. Tung EL, Wroblewski KE, Boyd K, Makelarski JA, Peek ME, Lindau ST. Police-Recorded Crime and Disparities in Obesity and Blood Pressure Status in Chicago. J Am Heart Assoc. 2018;7(7).
251. Tung EL, Chua RFM, Besser SA, Lindau ST, Kolak M, Anyanwu EC, et al. Association of Rising Violent Crime With Blood Pressure and Cardiovascular Risk: Longitudinal Evidence From Chicago, 2014-2016. Am J Hypertens. 2019;32(12):1192-8.
252. Uddin J, Malla G, Long DL, Zhu S, Black N, Cherrington A, et al. The association between neighborhood social and economic environment and prevalent diabetes in urban and rural communities: The Reasons for Geographic and Racial Differences in Stroke (REGARDS) study. SSM Popul Health. 2022;17:101050.
253. Uddin J, Zhu S, Adhikari S, Nordberg CM, Howell CR, Malla G, et al. Age and sex differences in the association between neighborhood socioeconomic environment and incident diabetes: Results from the diabetes location, environmental attributes and disparities (LEAD) network. SSM Popul Health. 2023;24:101541.
254. Usher T, Gaskin DJ, Bower K, Rohde C, Thorpe RJ, Jr. Residential Segregation and Hypertension Prevalence in Black and White Older Adults. J Appl Gerontol. 2018;37(2):177-202.
255. Vart P, Coresh J, Kwak L, Ballew SH, Heiss G, Matsushita K. Socioeconomic Status and Incidence of Hospitalization With Lower-Extremity Peripheral Artery Disease: Atherosclerosis Risk in Communities Study. J Am Heart Assoc. 2017;6(8).
256. Villani M, Earnest A, Smith K, de Courten B, Zoungas S. Geographical variation of diabetic emergencies attended by prehospital Emergency Medical Services is associated with measures of ethnicity and socioeconomic status. Sci Rep. 2018;8(1):5122.
257. Vintimilla R, Seyedahmadi A, Hall J, Johnson L, O'Bryant S. Association of Area Deprivation Index and hypertension, diabetes, dyslipidemia, and Obesity: A Cross-Sectional Study of the HABS-HD Cohort. Gerontol Geriatr Med. 2023;9:23337214231182240.
258. Vogt TM, Mullooly JP, Ernst D, Pope CR, Hollis JF. Social networks as predictors of ischemic heart disease, cancer, stroke and hypertension: incidence, survival and mortality. J Clin Epidemiol. 1992;45(6):659-66.
259. Wagner KJ, Boing AF, Subramanian SV, Höfelmann DA, D'Orsi E. Effects of neighborhood socioeconomic status on blood pressure in older adults. Rev Saude Publica. 2016;50:78.

260. Walter N, Robbins C, Murphy ST, Ball-Rokeach SJ. The weight of networks: the role of social ties and ethnic media in mitigating obesity and hypertension among Latinas. Ethn Health. 2019;24(7):790-803.
261. Wang Q, Lan ZL. Park green green spaces, public health and social inequalities: Understanding the interrelationships for policy implications. Land Use Pol. 2019;83:66-74.
262. Wang W, Zhang M, Xu CD, Ye PP, Liu YN, Huang ZJ, et al. Hypertension Prevalence, Awareness, Treatment, and Control and Their Associated Socioeconomic Factors in China: A Spatial Analysis of A National Representative Survey. Biomed Environ Sci. 2021;34(12):937-51.
263. Welin CL, Rosengren A, Wilhelmsen LW. Social relationships and myocardial infarction: a case-control study. J Cardiovasc Risk. 1996;3(2):183-90.
264. White K, Borrell LN, Wong DW, Galea S, Ogedegbe G, Glymour MM. Racial/ethnic residential segregation and self-reported hypertension among US- and foreign-born blacks in New York City. Am J Hypertens. 2011;24(8):904-10.
265. White JS, Hamad R, Li X, Basu S, Ohlsson H, Sundquist J, Sundquist K. Long-term effects of neighbourhood deprivation on diabetes risk: quasi-experimental evidence from a refugee dispersal policy in Sweden. Lancet Diabetes Endocrinol. 2016;4(6):517-24.
266. Wight RG, Cummings JR, Miller-Martinez D, Karlamangla AS, Seeman TE, Aneshensel CS. A multilevel analysis of urban neighborhood socioeconomic disadvantage and health in late life. Soc Sci Med. 2008;66(4):862-72.
267. Williams PC, Alhasan DM, Gaston SA, Henderson KL, Braxton Jackson W, 2nd, Jackson CL. Perceived neighborhood social cohesion and type 2 diabetes mellitus by age, sex/gender, and race/ethnicity in the United States. Prev Med. 2023;170:107477.
268. Xiao Q, Heiss G, Kucharska-Newton A, Bey G, Love SM, Whitsel EA. Life-Course Neighborhood Socioeconomic Status and Cardiovascular Events in Black and White Adults in the Atherosclerosis Risk in Communities Study. Am J Epidemiol. 2022;191(8):1470-84.
269. Xie HJ, Wang QK, Zhou XL, Yang YP, Mao YW, Zhang X. Built Environment Factors Influencing Prevalence of Hypertension at Community Level in China: The Case of Wuhan. SUSTAINABILITY. 2021;13(10).
270. Xu J, Lawrence KG, O'Brien KM, Jackson CL, Sandler DP. Association between neighbourhood deprivation and hypertension in a US-wide Cohort. J Epidemiol Community Health. 2022;76(3):268-73.
271. Xu J, Jing Y, Xu X, Zhang X, Liu Y, He H, et al. Spatial scale analysis for the relationships between the built environment and cardiovascular disease based on multi-source data. Health Place. 2023;83:103048.
272. Yadav S, Garg S, Raut AV. Evaluation of association of psychosocial stress and hypertension in adults >30 years of age: A community-based case-control study from Rural Central India. INTERNATIONAL JOURNAL OF NONCOMMUNICABLE DISEASES. 2021;6(3):142-8.
273. Yadav RS, Chaudhary D, Avula V, Shahjouei S, Azarpazhooh MR, Abedi V, et al. Social Determinants of Stroke Hospitalization and Mortality in United States' Counties. J Clin Med. 2022;11(14).
274. Yan T, Escarce JJ, Liang LJ, Longstreth WT, Jr., Merkin SS, Ovbiagele B, et al. Exploring psychosocial pathways between neighbourhood characteristics and stroke in older adults: the cardiovascular health study. Age Ageing. 2013;42(3):391-7.
275. Yan LD, McNairy ML, Dévieux JG, Pierre JL, Dade E, Sufra R, et al. Neighborhood cohesion and violence in Port-au-Prince, Haiti, and their relationship to stress, depression, and hypertension: Findings from the Haiti cardiovascular disease cohort study. PLOS Glob Public Health. 2022;2(7).
276. Yang YC, Li T, Ji Y. Impact of social integration on metabolic functions: evidence from a nationally representative longitudinal study of US older adults. BMC Public Health. 2013;13:1210.
277. Yang YC, Boen C, Mullan Harris K. Social relationships and hypertension in late life: evidence from a nationally representative longitudinal study of older adults. J Aging Health. 2015;27(3):403-31.
278. Yang YC, Boen C, Gerken K, Li T, Schorpp K, Harris KM. Social relationships and physiological determinants of longevity across the human life span. Proc Natl Acad Sci U S A. 2016;113(3):578-83.

279. Yazawa A, Inoue Y, Fujiwara T, Stickley A, Shirai K, Amemiya A, et al. Association between social participation and hypertension among older people in Japan: the JAGES Study. Hypertens Res. 2016;39(11):818-24.
280. Young DR, Fischer H, Arterburn D, Bessesen D, Cromwell L, Daley MF, et al. Associations of overweight/obesity and socioeconomic status with hypertension prevalence across racial and ethnic groups. J Clin Hypertens (Greenwich). 2018;20(3):532-40.
281. Yu MY, Velasquez AJ, Campos B, Robinette JW. Perceived neighborhood disorder and type 2 diabetes disparities in Hispanic, Black, and White Americans. Front Public Health. 2024;12:1258348.
