# Supplementary material for: Associations between dimensions of the social environment and cardiometabolic health outcomes: a systematic review and meta-analysis
Source: BMJ Open. 2024 Aug 28;14(8):e079987. doi: 10.1136/bmjopen-2023-079987 (PMC11367359; doi:10.1136/bmjopen-2023-079987)
Supplement: online supplemental file 7 [file bmjopen-14-8-s007.pdf]

Supplementary Table 1. Effect estimates of included studies

Reference	First author, year	Social environment dimension	Outcome category	Sex-specific	Effect metric	Association n	Association level	Exposure level	Effect estimate	Lower bound 95%CI	Upper bound 95%CI	SE, if available
[1]	Abba, 2021	Economic and Social Disadvantage	Hypertensive diseases	overall	OR (95%CI)	1	1	Least deprived (reference)	1.00	-	-	
						1	2	Moderate deprived	1.10	1.06	1.14	
						1	3	Most deprived	1.14	1.10	1.17	
[2]	Adams, 2009	Economic and Social Disadvantage	Diabetes mellitus	overall	OR (95%CI)	1	1	I (most disadvantage, reference)	1.00	-	-	
						1	2	II	0.80	not reported	not reported	
						1	3	III	0.70	not reported	not reported	
						1	4	IV	0.80	not reported	not reported	
						1	5	V	0.70	not reported	not reported	
[3]	Agabiti, 2009	Economic and Social Disadvantage	Diabetes mellitus	overall	RR (95%CI)	1	1	I (reference)	1.00	-	-	
						1	2	II	1.08	0.87	1.34	
						1	3	III	1.46	1.19	1.80	
						1	4	IV	1.68	1.37	2.07	
						1	5	V (low income)	2.77	2.29	3.36	
		Economic and Social Disadvantage	Hypertensive diseases	overall	RR (95%CI)	2	1	I (reference)	1.00	-	-	
						2	2	II	1.00	0.80	1.26	
						2	3	III	1.19	0.95	1.48	
						2	4	IV	1.33	1.06	1.68	
						2	5	V (low income)	1.64	1.31	2.04	
		Economic and Social Disadvantage	Other forms of heart disease	overall	RR (95%CI)	3	1	I (reference)	1.00	-	-	
						3	2	II	1.80	1.44	2.24	
						3	3	III	2.18	1.76	2.71	
						3	4	IV	2.97	2.41	3.65	
						3	5	V (low income)	3.78	3.09	4.62	
		Economic and Social Disadvantage	Ischaemic heart diseases	overall	RR (95%CI)	4	1	I (reference)	1.00	-	-	
						4	2	II	1.33	1.13	1.57	
						4	3	III	1.43	1.21	1.68	
						4	4	IV	1.53	1.31	1.79	
						4	5	V (low income)	1.97	1.70	2.30	

[4]	Agyemang, 2007	Crime and Safety	Hypertensive diseases	overall	OR (95%CI)	1	1	Dutch	1.07	0.67	1.71	
		Crime and Safety	Hypertensive diseases	overall	OR (95%CI)	2	1	Turk	1.18	0.75	1.86	
		Crime and Safety	Hypertensive diseases	overall	OR (95%CI)	3	1	Moroc.	1.36	0.82	2.26	
		Civic Participation and Engagement	Hypertensive diseases	overall	OR (95%CI)	4	1	Dutch	0.71	0.46	1.09	
		Civic Participation and Engagement	Hypertensive diseases	overall	OR (95%CI)	5	1	Turk	0.77	0.49	1.22	
		Civic Participation and Engagement	Hypertensive diseases	overall	OR (95%CI)	6	1	Moroccan	0.64	0.37	1.18	
[5]	Ahern, 2005	Social Cohesion and Social Capital	Hypertensive diseases	overall	OR (95%CI)	1	1	Collective generalized reciprocity	2.58	1.28	5.21	
		Social Cohesion and Social Capital	Diabetes mellitus	overall	OR (95%CI)	2	1	Personal quantitative social support (increased value = better social support)	1.38	1.10	1.72	
		Social Cohesion and Social Capital	Diabetes mellitus	overall	OR (95%CI)	3	1	Personal neighbourhood trust (increased value = better neighborhood trust)	0.68	0.50	0.92	
		Social Cohesion and Social Capital	Diabetes mellitus	overall	OR (95%CI)	4	1	Collective generalized reciprocity (increased value = better reciprocity)	4.01	1.49	10.79	
[6]	Akwo, 2018	Economic and Social Disadvantage	Other forms of heart disease	overall	HR (95%CI)	1	1		1.12	1.07	1.18	
[7]	Alemi, 2023	Discrimination and Segregation	Diabetes mellitus	overall	LASSO regression	1	1		0.27	-	-	0.45
		Discrimination and Segregation	Diabetes mellitus	overall	LASSO regression	2	1		0.25	-	-	0.44
		Economic and Social Disadvantage	Diabetes mellitus	overall	LASSO regression	3	1		0.43	-	-	0.49
[8]	Allan, 2020	Economic and Social Disadvantage	Other forms of heart disease	overall	Incidence rate per 100,000 persons (95%CI)	1	1	I (low deprivation, reference)	63.60	61.50	65.70	
						1	2	II	68.50	67.00	70.00	
						1	3	III	77.40	76.10	78.80	
						1	4	IV	72.40	71.00	73.90	
						1	5	V	77.10	75.40	78.90	
		Economic and Social Disadvantage	Other forms of heart disease	overall	Incidence rate per 100,000 persons (95%CI)	2	1	I (low income, reference)	74.10	72.50	75.80	
						2	2	II	70.00	68.60	71.40	
						2	3	III	74.50	73.10	75.90	
						2	4	IV	73.40	71.90	74.90	
						2	5	V	68.20	66.30	70.10	

[9]	Altevers, 2016	Social Relationships and Norms	Diabetes mellitus	male	HR (95%CI)	1	1		1.31	1.11	1.55
		Social Relationships and Norms	Diabetes mellitus	female	HR (95%CI)	2	1		1.10	0.88	1.37
		Social Relationships and Norms	Hypertensive diseases	male	%	3	1	poor structural social support	0.46	not reported	not reported
						3	2	good structural social support	0.45	not reported	not reported
		Social Relationships and Norms	Hypertensive diseases	female	%	4	1	poor structural social support	0.34	not reported	not reported
						4	2	good structural social support	0.27	not reported	not reported
[10]	Andersen, 2008	Economic and Social Disadvantage	Diabetes mellitus	female	OR (95%CI)	1	1		1.30	1.12	1.51
[11]	Anderson, 2019	Economic and Social Disadvantage	Diabetes mellitus	overall	OR (95%CI)	1	1		1.07	not reported	not reported
		Economic and Social Disadvantage	Diabetes mellitus	overall	OR (95%CI)	2	1		1.13	not reported	not reported
		Economic and Social Disadvantage	Diabetes mellitus	overall	OR (95%CI)	3	1		0.91	not reported	not reported
		Economic and Social Disadvantage	Diabetes mellitus	overall	OR (95%CI)	4	1		0.99	not reported	not reported
		Economic and Social Disadvantage	Hypertensive diseases	overall	OR (95%CI)	5	1		1.06	not reported	not reported
		Economic and Social Disadvantage	Hypertensive diseases	overall	OR (95%CI)	6	1		1.10	not reported	not reported
		Economic and Social Disadvantage	Hypertensive diseases	overall	OR (95%CI)	7	1		0.93	not reported	not reported
		Economic and Social Disadvantage	Hypertensive diseases	overall	OR (95%CI)	8	1		1.00	not reported	not reported
		Economic and Social Disadvantage	Ischaemic heart diseases	overall	OR (95%CI)	9	1		1.04	not reported	not reported
		Economic and Social Disadvantage	Ischaemic heart diseases	overall	OR (95%CI)	10	1		1.08	not reported	not reported
		Economic and Social Disadvantage	Ischaemic heart diseases	overall	OR (95%CI)	11	1		0.92	not reported	not reported
		Economic and Social Disadvantage	Ischaemic heart diseases	overall	OR (95%CI)	12	1		0.97	not reported	not reported

[12]	Atasoy, 2022	Social Relationships and Norms	Diabetes mellitus	overall	HR (95%CI)	1	1	least socially connected	1.27	1.01	1.61
						1	2		na	-	-
						1	3		na	-	-
						1	4	strongly socially connected (reference)	1.00	-	-
[13]	Augustin, 2008	Economic and Social Disadvantage	Ischaemic heart diseases	overall	OR (95%CI)	1	1	Q1 (lowest index, reference)	1.00	-	-
						1	2	Q2	1.89	0.65	5.46
						1	3	Q3	2.15	0.69	6.64
						1	4	Q4 (highest scale/index)	4.68	1.50	14.60
		Economic and Social Disadvantage	Ischaemic heart diseases	overall	OR (95%CI)	2	1	Q1 (lowest index, reference)	1.00	-	-
						2	2	Q2	1.85	0.72	4.78
						2	3	Q3	1.92	0.84	4.38
						2	4	Q4 (highest scale/index)	3.33	1.38	8.05
		Economic and Social Disadvantage	Ischaemic heart diseases	overall	OR (95%CI)	3	1	Q1 (lowest index, reference)	1.00	-	-
						3	2	Q2	1.10	0.52	2.34
						3	3	Q3	0.63	0.29	1.39
						3	4	Q4 (highest scale/index)	0.39	0.13	1.15
		Economic and Social Disadvantage	Diseases of the circulatory system	overall	OR (95%CI)	4	1	Q1 (lowest index, reference)	1.00	-	-
						4	2	Q2	2.46	1.09	5.54
						4	3	Q3	3.37	1.46	7.81
						4	4	Q4 (highest scale/index)	3.81	1.58	9.23
		Economic and Social Disadvantage	Diseases of the circulatory system	overall	OR (95%CI)	5	1	Q1 (lowest index, reference)	1.00	-	-
						5	2	Q2	1.90	1.00	3.63
						5	3	Q3	2.14	1.23	3.74
						5	4	Q4 (highest scale/index)	2.09	1.09	3.98
		Economic and Social Disadvantage	Diseases of the circulatory system	overall	OR (95%CI)	6	1	Q1 (lowest index, reference)	1.00	-	-
						6	2	Q2	1.02	0.59	1.75
						6	3	Q3	0.69	0.40	1.18
						6	4	Q4 (highest scale/index)	0.32	0.14	0.73
[14]	Avogo, 2023	Economic and Social Disadvantage	Hypertensive diseases	female	OR (95%CI)	1	1	low percent of women educated (reference)	1.00	-	-
						1	2	Medium and High	1.03	0.86	1.25

		Economic and Social Disadvantage	Hypertensive diseases	female	OR (95%CI)	2	1	low percent of poor women (reference)	1.00	-	-	
						2	2	Medium and High	0.78	0.66	0.92	
		Economic and Social Disadvantage	Hypertensive diseases	female	OR (95%CI)	3	1	low percent of working women(reference)	1.00	-	-	
						3	2	Medium and High	1.12	1.01	1.24	
[15]	Baek, 2016	Social Relationships and Norms	Hypertensive diseases	male	OR (95%CI)	1	1		0.75	0.58	0.96	
		Social Relationships and Norms	Hypertensive diseases	female	OR (95%CI)	2	1		1.00	0.78	1.28	
		Social Relationships and Norms	Hypertensive diseases	male	OR (95%CI)	3	1		0.90	0.50	1.64	
		Social Relationships and Norms	Hypertensive diseases	female	OR (95%CI)	4	1		0.84	0.49	1.46	
[16]	Banchani, 2020	Economic and Social Disadvantage	Hypertensive diseases	female	OR (95%CI)	1	1		1.08	not reported	not reported	0.15
[17]	Bancks, 2017	Discrimination and Segregation	Diabetes mellitus	female	HR (95%CI)	1	1		1.01	0.81	1.26	
		Discrimination and Segregation	Diabetes mellitus	male	HR (95%CI)	2	1		1.21	0.96	1.54	
		Economic and Social Disadvantage	Diabetes mellitus	female	HR (95%CI)	3	1		0.99	0.83	1.17	
		Economic and Social Disadvantage	Diabetes mellitus	male	HR (95%CI)	4	1		0.88	0.72	1.08	
[18]	Barber, 2016	Economic and Social Disadvantage	Diabetes mellitus	overall	%	1	1	high disadvantage	22.80	not reported	not reported	
					%	1	2	medium	15.50	not reported	not reported	
						1	3	low	12.50	not reported	not reported	
		Economic and Social Disadvantage	Hypertensive diseases	overall	%	2	1	high disadvantage	63.60	not reported	not reported	
						2	2	medium	55.60	not reported	not reported	
						2	3	low	47.70	not reported	not reported	
[19]	Barber, 2018	Economic and Social Disadvantage	Hypertensive diseases	overall	OR (95%CI)	1	1		1.05	1.03	1.08	
		Economic and Social Disadvantage	Diabetes mellitus	overall	OR (95%CI)	2	1		1.07	1.04	1.11	

[20]	Barefoot, 2005	Social Relationships and Norms	Ischaemic heart diseases	overall	HR (95%CI)	1	1	Parents	0.58	0.39	0.88	
		Social Relationships and Norms	Ischaemic heart diseases	overall	HR (95%CI)	2	1	Children	0.81	0.66	1.00	
		Social Relationships and Norms	Ischaemic heart diseases	overall	HR (95%CI)	3	1	Family	0.79	0.65	0.96	
		Social Relationships and Norms	Ischaemic heart diseases	overall	HR (95%CI)	4	1	Colleagues	1.07	0.82	1.41	
		Social Relationships and Norms	Ischaemic heart diseases	overall	HR (95%CI)	5	1	Neighbors	1.13	0.93	1.37	
		Social Relationships and Norms	Ischaemic heart diseases	overall	HR (95%CI)	6	1	Friends	0.86	0.70	1.05	
		Social Relationships and Norms	Ischaemic heart diseases	overall	HR (95%CI)	7	1	Spouse/partner	0.96	0.78	1.17	
		Social Relationships and Norms	Ischaemic heart diseases	overall	HR (95%CI)	3	1	6 to 7 sources	0.56	0.22	1.14	
						3	2	5	0.87	0.59	1.29	
						3	3	4	0.67	0.47	0.94	
						3	4	3	0.83	0.60	1.13	
						3	5	2	0.83	0.60	1.15	
						3	6	0-1 (reference)	1.00	-	-	
		Social Relationships and Norms	Ischaemic heart diseases	overall	HR (95%CI)	4	1	6 to 7 sources	0.31	0.11	0.83	
						4	2	5	0.62	0.37	1.03	
						4	3	4	0.64	0.40	1.05	
						4	4	3	0.79	0.49	1.28	
						4	5	2	0.85	0.51	1.42	
						4	6	0-1 (reference)	1.00	-	-	
[21]	Basile Ibrahim, 2021	Economic and Social Disadvantage	Hypertensive diseases	female	OR (95%CI)	1	1	less vulnerable neigborhoods (SVI index) (reference)	1.00	-	-	
						1	2	most vulnerable neigborhoods (SVI index)	3.29	1.30	8.32	
[22]	Bevan, 2023	Economic and Social Disadvantage	Ischaemic heart diseases	overall	Beta	1	1		0.05			0.00
[23]	Bhavsar, 2022	Economic and Social Disadvantage	Diabetes mellitus	overall	OR (95%CI)	1	1	gentrified neighborhood	1.00	0.89	1.13	
						1	2	not gentrified neighborhood (reference)	1.00	-	-	

		Economic and Social Disadvantage	Hypertensive diseases	overall	OR (95%CI)	2	1	gentrified neighborhood	0.96	0.86	1.06
						2	2	not gentrified neighborhood (reference)	1.00	-	-
		Economic and Social Disadvantage	Diseases of the circulatory system	overall	OR (95%CI)	3	1	gentrified neighborhood	0.93	0.78	1.12
						3	2	not gentrified neighborhood (reference)	1.00	-	-
[24]	Bhopal, 2002	Economic and Social Disadvantage	Ischaemic heart diseases	male	Percentage excess of OR	1	1		27.00	not reported	not reported
		Economic and Social Disadvantage	Ischaemic heart diseases	male	Percentage excess of OR	2	1		-10.00	not reported	not reported
		Economic and Social Disadvantage	Ischaemic heart diseases	male	Percentage excess of OR	3	1		40.00	not reported	not reported
		Economic and Social Disadvantage	Ischaemic heart diseases	male	Percentage excess of OR	4	1		-43.00	not reported	not reported
		Economic and Social Disadvantage	Ischaemic heart diseases	male	Percentage excess of OR	5	1		12.00	not reported	not reported
		Economic and Social Disadvantage	Ischaemic heart diseases	female	Percentage excess of OR	6	1		55.00	not reported	not reported
		Economic and Social Disadvantage	Ischaemic heart diseases	female	Percentage excess of OR	7	1		-8.00	not reported	not reported
		Economic and Social Disadvantage	Ischaemic heart diseases	female	Percentage excess of OR	8	1		-24.00	not reported	not reported
		Economic and Social Disadvantage	Ischaemic heart diseases	female	Percentage excess of OR	9	1		63.00	not reported	not reported
		Economic and Social Disadvantage	Ischaemic heart diseases	female	Percentage excess of OR	10	1		-45.00	not reported	not reported
[25]	Bilal, 2018	Economic and Social Disadvantage	Diabetes mellitus	male	PR (95%CI)	1	1	T1 (low neighborhood socioeconomic status, reference)	1.00	-	-
						1	2	T2 (middle neighborhood socioeconomic status)	0.92	0.89	0.96
						1	3	T3 (high neighborhood socioeconomic status)	0.76	0.74	0.80
		Economic and Social Disadvantage	Diabetes mellitus	male	PR (95%CI)	2	1		0.86	0.84	0.87
		Economic and Social Disadvantage	Diabetes mellitus	male	PR (95%CI)	3	1	T1 (low neighborhood socioeconomic status, reference)	1.00	-	-
						3	2	T2 (middle neighborhood socioeconomic status)	0.76	0.73	0.79

						3	3	T3 (high neighborhood socioeconomic status)	0.54	0.52	0.57
		Economic and Social Disadvantage	Diabetes mellitus	male	PR (95%CI)	4	1		0.74	0.72	0.75
		Economic and Social Disadvantage	Diabetes mellitus	male	HR (95%CI)	5	1	T1 (low neighborhood socioeconomic status, reference)	1.00	-	-
						5	2	T2 (middle neighborhood socioeconomic status)	0.87	0.77	0.99
						5	3	T3 (high neighborhood socioeconomic status)	0.80	0.71	0.91
		Economic and Social Disadvantage	Diabetes mellitus	male	HR (95%CI)	6	1		0.90	0.85	0.94
		Economic and Social Disadvantage	Diabetes mellitus	male	HR (95%CI)	7	1	T1 (low neighborhood socioeconomic status, reference)	1.00	-	-
						7	2	T2 (middle neighborhood socioeconomic status)	0.83	0.71	0.97
						7	3	T3 (high neighborhood socioeconomic status)	0.69	0.59	0.80
		Economic and Social Disadvantage	Diabetes mellitus	male	HR (95%CI)	8	1		0.82	0.77	0.87
[26]	Bocour, 2016	Economic and Social Disadvantage	Hypertensive diseases	overall	comparison of rate ratios	1	1	comparison of rate ratios	3.77 to 3.03, APC = -7.10% (APC = annual percent change)	not reported	not reported
		Economic and Social Disadvantage	Other forms of heart disease	overall	comparison of rate ratios	2	1	comparison of rate ratios	2.75 to 2.61, APC = -1.64% (APC = annual percent change)	not reported	not reported
		Economic and Social Disadvantage	Ischaemic heart diseases	overall	comparison of rate ratios	3	1	comparison of rate ratios	3.90 to 2.89, APC = -9.73% (APC = annual percent change)	not reported	not reported
[27]	Borges, 2021	Social Cohesion and Social Capital	Diabetes mellitus	overall	Pearson Correlations	1	1		0.34	-	-
		Social Cohesion and Social Capital	Diabetes mellitus		Pearson Correlations	2	1		0.11	-	-

		Social Cohesion and Social Capital	Diabetes mellitus		Pearson Correlations	3	1		0.03	-	-
		Social Cohesion and Social Capital	Diabetes mellitus		Pearson Correlations	4	1		-0.19	-	-
		Social Cohesion and Social Capital	Diabetes mellitus		Pearson Correlations	5	1		0.34	-	-
		Social Cohesion and Social Capital	Diabetes mellitus		Pearson Correlations	6	1		0.23	-	-
[28]	Boruzs, 2018	Economic and Social Disadvantage	Ischaemic heart diseases	overall	RR (95%CI)	1	1		0.74	0.73	0.74
						1	2		1.07	1.07	1.08
						1	3		1.31	1.30	1.31
		Economic and Social Disadvantage	Cerebrovascular diseases	overall	RR (95%CI)	2	1		0.80	0.80	0.81
						2	2		1.08	1.07	1.08
						2	3		1.20	1.19	1.21
		Economic and Social Disadvantage	Hypertensive diseases	overall	RR (95%CI)	3	1		0.94	0.94	0.94
						3	2		1.03	1.03	1.04
						3	3		1.05	1.04	1.05
[29]	Bravo, 2018	Discrimination and Segregation	Diabetes mellitus	overall	RR (95%CI)	1	1		1.07	1.05	1.10
		Discrimination and Segregation	Diabetes mellitus	overall	RR (95%CI)	2	1		1.24	1.17	1.31
[30]	Bravo, 2019	Discrimination and Segregation	Hypertensive diseases	overall	OR (95%CI)	1	1		1.06	1.03	1.10
		Discrimination and Segregation	Hypertensive diseases	overall	OR (95%CI)	2	1		1.11	1.07	1.16
[31]	Bray, 2018	Economic and Social Disadvantage	Cerebrovascular diseases	overall	IRR (95%CI)	1	1	1- most deprived area	2.00	1.70	2.30
						1	2	2	1.80	1.50	2.00
						1	3	3	1.60	1.40	1.80
						1	4	4	1.50	1.30	1.70
						1	5	5	1.40	1.20	1.60
						1	6	6	1.30	1.10	1.50
						1	7	7	1.20	1.00	1.40
						1	8	8	1.20	1.00	1.40
						1	9	9	1.10	1.00	1.30
						1	10	10 - least deprived areas (reference)	1.00	-	-

		Economic and Social Disadvantage	Cerebrovascular diseases	overall	IRR (95%CI)	2	1	1- most deprived area	1.60	1.30	1.90
						2	2	2	1.50	1.20	1.70
						2	3	3	1.30	1.10	1.60
						2	4	4	1.20	1.00	1.40
						2	5	5	1.20	1.00	1.40
						2	6	6	1.10	0.90	1.30
						2	7	7	1.10	0.90	1.20
						2	8	8	1.00	0.90	1.20
						2	9	9	1.00	0.90	1.20
						2	10	10 - least deprived areas (reference)	1.00	-	-
[32]	Brinkhues, 2017	Social Relationships and Norms	Diabetes mellitus	female	OR (95%CI)	1	1		1.08	1.04	1.13
		Social Relationships and Norms	Diabetes mellitus	female	OR (95%CI)	2	1		0.98	0.97	1.00
		Social Relationships and Norms	Diabetes mellitus	female	OR (95%CI)	3	1		1.07	0.99	1.15
		Social Relationships and Norms	Diabetes mellitus	female	OR (95%CI)	4	1		1.09	1.01	1.19
		Social Relationships and Norms	Diabetes mellitus	female	OR (95%CI)	5	1		1.14	1.04	1.26
		Social Relationships and Norms	Diabetes mellitus	female	OR (95%CI)	6	1		2.12	1.44	3.13
		Social Relationships and Norms	Diabetes mellitus	female	OR (95%CI)	7	1		1.09	0.97	1.23
		Social Relationships and Norms	Diabetes mellitus	female	OR (95%CI)	8	1		1.12	0.99	1.27
		Social Relationships and Norms	Diabetes mellitus	female	OR (95%CI)	9	1		1.11	0.98	1.26
		Social Relationships and Norms	Diabetes mellitus	female	OR (95%CI)	10	1		1.16	1.02	1.32
		Social Relationships and Norms	Diabetes mellitus	female	OR (95%CI)	11	1		1.21	1.05	1.41
		Social Relationships and Norms	Diabetes mellitus	male	OR (95%CI)	12	1		1.05	1.02	1.09
		Social Relationships and Norms	Diabetes mellitus	male	OR (95%CI)	13	1		0.99	0.98	1.02

		Social Relationships and Norms	Diabetes mellitus	male	OR (95%CI)	14	1		1.04	0.98	1.09
		Social Relationships and Norms	Diabetes mellitus	male	OR (95%CI)	15	1		1.05	0.99	1.12
		Social Relationships and Norms	Diabetes mellitus	male	OR (95%CI)	16	1		1.04	0.98	1.11
		Social Relationships and Norms	Diabetes mellitus	male	OR (95%CI)	17	1		1.94	1.29	2.93
		Social Relationships and Norms	Diabetes mellitus	male	OR (95%CI)	18	1		1.02	0.93	1.10
		Social Relationships and Norms	Diabetes mellitus	male	OR (95%CI)	19	1		1.06	0.96	1.16
		Social Relationships and Norms	Diabetes mellitus	male	OR (95%CI)	20	1		1.11	1.01	1.22
		Social Relationships and Norms	Diabetes mellitus	male	OR (95%CI)	21	1		1.04	0.95	1.14
		Social Relationships and Norms	Diabetes mellitus	male	OR (95%CI)	22	1		1.13	1.02	1.25
[33]	Bush, 2023	Economic and Social Disadvantage	Diabetes mellitus	male	HR (95%CI)	1	1	Q1 least socioeconomically deprived (reference)	1.00	-	-
						1	2		0.95	0.69	1.32
						1	3		1.13	0.81	1.59
						1	4		1.42	1.01	1.99
						1	5	Q5 most socioeconomically deprived	1.16	0.81	1.67
[34]	Butler, 2010	Economic and Social Disadvantage	Diabetes mellitus	overall	Correlation	1	1	Pairwise correlation of disadvantage score (IRSD) and diabetes rate per 1000	0.53	not reported	not reported
[35]	Buys, 2015	Economic and Social Disadvantage	Hypertensive diseases	overall	OR (95%CI)	1	1	low neighborhood disadvantage	1.00	-	-
						1	2	mid neighborhood disadvantage	1.60	1.20	2.10
						1	3	high neighborhood disadvantage	1.80	1.30	2.30
		Social Relationships and Norms	Hypertensive diseases	overall	OR (95%CI)	2	1		1.00	1.00	1.10
		Disorder and Incivilities	Hypertensive diseases	overall	OR (95%CI)	3	1	yes (disorder) vs. no (disorder)	0.70	0.50	1.10
[36]	Carlsson, 2016	Economic and Social Disadvantage	Ischaemic heart diseases	male	HR (95%CI)	1	1	high neighborhood socioeconomic status	0.75	0.66	0.84

						1	2	middle neighborhood socioeconomic status (reference)	1.00	-	-
						1	3	low neighborhood socioeconomic status	1.24	1.13	1.36
		Economic and Social Disadvantage	Ischaemic heart diseases	female	HR (95%CI)	2	1	high neighborhood socioeconomic status	0.67	0.55	0.83
						2	2	middle neighborhood socioeconomic status (reference)	1.00	-	-
						2	3	low neighborhood socioeconomic status	1.17	1.00	1.38
		Economic and Social Disadvantage	Ischaemic heart diseases	male	HR (95%CI)	3	1	high neighborhood socioeconomic status	0.73	0.66	0.80
						3	2	middle neighborhood socioeconomic status (reference)	1.00	-	-
						3	3	low neighborhood socioeconomic status	1.23	1.14	1.32
		Economic and Social Disadvantage	Ischaemic heart diseases	female	HR (95%CI)	4	1	high neighborhood socioeconomic status	0.74	0.64	0.85
						4	2	middle neighborhood socioeconomic status (reference)	1.00	-	-
						4	3	low neighborhood socioeconomic status	1.24	1.10	1.40
[37]	Carlsson, 2017	Economic and Social Disadvantage	Cerebrovascular diseases	male	HR (95%CI)	1	1	high neighborhood socioeconomic status	0.92	0.80	1.06
						1	2	middle neighborhood socioeconomic status (reference)	1.00	-	-
						1	3	low neighborhood socioeconomic status	1.04	0.92	1.18
		Economic and Social Disadvantage	Cerebrovascular diseases	female	HR (95%CI)	2	1	high neighborhood socioeconomic status	0.91	0.78	1.06
						2	2	middle neighborhood socioeconomic status (reference)	1.00	-	-
						2	3	low neighborhood socioeconomic status	1.21	1.05	1.39
		Economic and Social Disadvantage	Cerebrovascular diseases	overall	HR (95%CI)	3	1	high neighborhood socioeconomic status	0.91	0.83	1.01
						3	2	middle neighborhood socioeconomic status (reference)	1.00	-	-
						3	3	low neighborhood socioeconomic status	1.12	1.02	1.22
[38]	Cebrecos, 2018	Economic and Social Disadvantage	Diseases of the circulatory system	overall	Correlation	1	1		0.33	not reported	not reported

		Economic and Social Disadvantage	Diseases of the circulatory system	overall	Correlation	2	1		0.64	not reported	not reported
		Economic and Social Disadvantage	Diseases of the circulatory system	overall	Correlation	3	1		0.74	not reported	not reported
[39]	Cené, 2022	Social Relationships and Norms	Other forms of heart disease	female	HR (95%CI)	1	1	Social isolation yes	1.56	1.37	1.78
						1	2	Social isolation no (reference)	1.00	-	-
[40]	Chaix, 2011	Economic and Social Disadvantage	Diabetes mellitus	overall	OR (95%CI)	1	1	high	1.00	-	-
						1	2	mid-high	1.05	0.70	1.56
						1	3	mid-low	1.19	0.80	1.75
						1	4	low	1.56	1.06	2.31
[41]	Chamberlain, 2022	Economic and Social Disadvantage	Hypertensive diseases	overall	OR (95%CI)	1	1	lowest depreviation quantile (reference)	1.00	-	-
						1	2		1.35	1.15	1.58
						1	3		1.66	1.42	1.94
						1	4		1.89	1.62	2.21
						1	5	highest depreviation quantile	2.57	2.15	3.06
		Economic and Social Disadvantage	Diabetes mellitus	overall	OR (95%CI)	2	1	lowest depreviation quantile (reference)	1.00	-	-
						2	2		1.12	1.00	1.25
						2	3		1.24	1.11	1.38
						2	4		1.37	1.23	1.53
						2	5	highest depreviation quantile	1.71	1.52	1.94
		Economic and Social Disadvantage	Other forms of heart disease	overall	OR (95%CI)	3	1	lowest depreviation quantile (reference)	1.00	-	-
						3	2		1.05	0.93	1.19
						3	3		1.14	1.01	1.28
						3	4		1.30	1.15	1.47
						3	5	highest depreviation quantile	1.55	1.34	1.78
		Economic and Social Disadvantage	Ischaemic heart diseases	overall	OR (95%CI)	4	1	lowest depreviation quantile (reference)	1.00	-	-
						4	2		1.13	0.98	1.31
						4	3		1.33	1.15	1.53
						4	4		1.51	1.30	1.75
						4	5	highest depreviation quantile	1.88	1.59	2.22

		Economic and Social Disadvantage	Other forms of heart disease	overall	OR (95%CI)	5	1	lowest depreviation quantile (reference)	1.00	-	-
						5	2		1.36	1.08	1.72
						5	3		1.56	1.24	1.96
						5	4		1.94	1.54	2.45
						5	5	highest depreviation quantile	2.48	1.92	3.21
		Economic and Social Disadvantage	Cerebrovascular diseases	overall	OR (95%CI)	6	1	lowest depreviation quantile (reference)	1.00	-	-
						6	2		1.17	0.97	1.40
						6	3		1.28	1.07	1.53
						6	4		1.47	1.23	1.77
						6	5	highest depreviation quantile	1.73	1.40	2.13
[42]	Chan, 2022	Economic and Social Disadvantage	Hypertensive diseases	overall	PR (95%CI)	1	1	non-slum urban (reference)	1.00	-	-
						1	2	Slum	0.99	0.94	1.03
						1	3	rural	0.91	0.87	0.95
		Economic and Social Disadvantage	Diabetes mellitus	overall	PR (95%CI)	2	1	non-slum urban (reference)	1.00	-	-
						2	2	Slum	1.03	0.94	1.13
						2	3	rural	0.73	0.67	0.80
		Economic and Social Disadvantage	Diseases of the circulatory system	overall	PR (95%CI)	3	1	non-slum urban (reference)	1.00	-	-
						3	2	Slum	1.01	0.89	1.14
						3	3	rural	0.79	0.71	0.88
[43]	Chang, 2017	Civic Participation and Engagement	Ischaemic heart diseases	female	HR (95%CI)	1	1	I (lowest social integration, reference)	1.00	-	-
						1	2	II	0.92	0.87	1.17
						1	3	III	0.90	0.80	1.01
						1	4	IV	0.84	0.74	0.96
[44]	Chatzi, 2020	Economic and Social Disadvantage	Diabetes mellitus	overall	OR (95%CI)	1	1	V (least deprived, reference)	1.00	-	-
						1	2	IV	0.73	0.45	1.19
						1	3	III	0.64	0.38	1.08
						1	4	II	1.21	0.72	2.01
						1	5	I (most deprived)	0.93	0.53	1.65
		Economic and Social Disadvantage	Diabetes mellitus	overall	OR (95%CI)	2	1	V (least deprived, reference)	1.00	-	-

						2	2	IV	1.65	0.32	1.16
						2	3	III	0.56	0.28	1.12
						2	4	II	1.20	0.64	2.25
						2	5	I (most deprived)	0.84	0.41	1.73
		Economic and Social Disadvantage	Diabetes mellitus	overall	OR (95%CI)	3	1	V (least deprived, reference)	1.00	-	-
						3	2	IV	0.90	0.44	1.86
						3	3	III	0.76	0.35	1.65
						3	4	II	1.21	0.54	2.69
						3	5	I (most deprived)	1.08	0.45	2.59
[45]	Cheruvalath, 2022	Economic and Social Disadvantage	Cerebrovascular diseases	overall	OR (95%CI)	1	1	Quartile 1 (reference, lowest deprivation)	1.00	-	-
						1	2	Quartile 2	1.18	0.98	1.41
						1	3	Quartile 3	1.05	0.88	1.26
						1	4	Quartile 4 (highest deprivation)	1.28	1.02	1.60
		Economic and Social Disadvantage	Cerebrovascular diseases	overall	OR (95%CI)	2	1	per SD increase	1.01	0.99	1.04
[46]	Child, 2022	Social Relationships and Norms	combined outcome	overall	Average Marginal Effects	1	1		0.02	0.01	0.04
		Social Relationships and Norms	combined outcome	overall	Average Marginal Effects	2	1		−0,03	−0,05	−0,01
		Social Relationships and Norms	combined outcome	overall	Average Marginal Effects	3	1		0.00	−0,02	0.02
		Social Relationships and Norms	combined outcome	overall	Average Marginal Effects	4	1		−0,02	−0,04	−0,01
		Social Relationships and Norms	combined outcome	overall	Average Marginal Effects	5	1		−0,02	−0,03	0.00
		Social Relationships and Norms	combined outcome	overall	Average Marginal Effects	6	1		−0,01	−0,02	0.01
		Social Relationships and Norms	combined outcome	overall	Average Marginal Effects	7	1		−0,00	−0,01	0.02
		Social Relationships and Norms	combined outcome	overall	Average Marginal Effects	8	1		0.00	−0,01	0.02
		Social Relationships and Norms	combined outcome	overall	Average Marginal Effects	9	1		−0,01	−0,02	0.00
		Social Relationships and Norms	combined outcome	overall	Average Marginal Effects	10	1		0.01	−0,01	0.02

[47]	Christine, 2015	Social Cohesion and Social Capital	Diabetes mellitus	overall	HR (95%CI)	1	1		1.00	0.89	1.11
		Crime and Safety	Diabetes mellitus	overall	HR (95%CI)	2	1		0.96	0.82	1.11
		Social Cohesion and Social Capital	Diabetes mellitus	overall	HR (95%CI)	3	1		0.98	0.88	1.10
		Crime and Safety	Diabetes mellitus	overall	HR (95%CI)	4	1		0.98	0.88	1.10
[48]	Chum, 2015	Crime and Safety	Ischaemic heart diseases	overall	OR (95%CI)	1	1	Q1 (lowest number of violent crimes)	0.87	0.76	0.98
						1	2	Q2	0.93	0.85	1.01
						1	3	Q3	0.98	0.87	1.09
						1	4	Q4 (high, reference)	1.00	-	-
		Crime and Safety	Diseases of the circulatory system	overall	OR (95%CI)	2	1	Q1 (lowest number of violent crimes)	0.80	0.71	0.89
						2	2	Q2	0.88	0.79	0.97
						2	3	Q3	0.95	0.72	1.18
						2	4	Q4 (high, reference)	1.00	-	-
[49]	Clark, 2011	Social Cohesion and Social Capital	Cerebrovascular diseases	overall	HR (95%CI)	1	1	1-point increase in cohesion	0.47	0.24	0.90
[50]	Claudel, 2018	Economic and Social Disadvantage	Hypertensive diseases	overall	OR (95%CI)	1	1	Low deprivation (NDI) (reference)	1.00	-	-
						1	2	medium	1.42	0.94	2.14
						1	3	High deprivation	1.69	1.02	2.82
[51]	Coelho, 2023	Economic and Social Disadvantage	Hypertensive diseases	female	OR (95%CI)	1	1	The OR is estimated for a 1 SD (0.30847) difference in sub-city educational attainment scores	1.07	1.02	1.12
		Economic and Social Disadvantage	Hypertensive diseases	male	OR (95%CI)	2	1	The OR is estimated for a 1 SD (0.30847) difference in sub-city educational attainment scores	1.11	1.05	1.18
[52]	Cofie, 2021	Social Relationships and Norms	Hypertensive diseases	overall	OR (95%CI)	1	1	network structure (% composition)	3.54	1.09	11.48
		Social Relationships and Norms	Hypertensive diseases	overall	OR (95%CI)	2	1	network structure (% composition)	1.39	0.57	3.37
		Social Relationships and Norms	Hypertensive diseases	overall	OR (95%CI)	3	1	network structure (% composition)	2.82	0.34	23.52
		Social Relationships and Norms	Hypertensive diseases	overall	OR (95%CI)	4	1	network structure (% composition)	1.27	0.38	4.23
		Social Relationships and Norms	Hypertensive diseases	overall	OR (95%CI)	5	1	network support (counts)	1.07	0.98	1.18
		Social Relationships and Norms	Hypertensive diseases	overall	OR (95%CI)	6	1	network support (counts)	1.07	0.90	1.28

		Social Relationships and Norms	Hypertensive diseases	overall	OR (95%CI)	7	1	network support (counts)	0.86	0.73	1.02
		Social Relationships and Norms	Hypertensive diseases	overall	OR (95%CI)	8	1	network support (counts)	0.97	0.83	1.13
		Social Relationships and Norms	Diabetes mellitus	overall	OR (95%CI)	9	1	network structure (% composition)	2.04	0.46	9.11
		Social Relationships and Norms	Diabetes mellitus	overall	OR (95%CI)	10	1	network structure (% composition)	1.55	0.48	4.97
		Social Relationships and Norms	Diabetes mellitus	overall	OR (95%CI)	11	1	network structure (% composition)	1.50	0.11	19.99
		Social Relationships and Norms	Diabetes mellitus	overall	OR (95%CI)	12	1	network structure (% composition)	0.36	0.06	2.11
		Social Relationships and Norms	Diabetes mellitus	overall	OR (95%CI)	13	1	network support (counts)	1.00	0.88	1.13
		Social Relationships and Norms	Diabetes mellitus	overall	OR (95%CI)	14	1	network support (counts)	0.91	0.75	1.11
		Social Relationships and Norms	Diabetes mellitus	overall	OR (95%CI)	15	1	network support (counts)	1.07	0.87	1.31
		Social Relationships and Norms	Diabetes mellitus	overall	OR (95%CI)	16	1	network support (counts)	0.92	0.75	1.14
[53]	Connolly, 2000	Economic and Social Disadvantage	Diabetes mellitus	male	Prevalence per 1,000 population	1	1	Q1, least deprived	13.40	11.50	15.40
						1	2	Q2	10.70	9.60	12.00
						1	3	Q3	16.00	14.80	17.30
						1	4	Q4	17.60	16.00	19.20
						1	5	Q5, most deprived	17.20	15.50	18.90
		Economic and Social Disadvantage	Diabetes mellitus	female	Prevalence per 1,000 population	2	1	Q1, least deprived	10.80	9.00	12.70
						2	2	Q2	11.10	9.80	12.30
						2	3	Q3	12.90	11.70	14.00
						2	4	Q4	13.50	12.10	14.80
						2	5	Q5, most deprived	15.50	13.80	17.10
[54]	Consolazio, 2020	Economic and Social Disadvantage	Diabetes mellitus	overall	OR (95%CI)	1	1	Extremely high value (reference)	1.00	-	-
						1	2	Moderately high	1.14	0.76	1.71
						1	3	Moderately low	1.27	0.85	1.91
						1	4	Extremely low	2.38	1.58	3.58

[55]	Cookson, 2012	Economic and Social Disadvantage	Other forms of heart disease	overall	PR (95%CI)	1	1		1.00	1.00	1.00	
		Economic and Social Disadvantage	Diabetes mellitus	overall	PR (95%CI)	2	1		1.01	1.01	1.01	
		Economic and Social Disadvantage	Other forms of heart disease	overall	PR (95%CI)	3	1		1.01	1.01	1.01	
		Economic and Social Disadvantage	Hypertensive diseases	overall	PR (95%CI)	4	1		1.00	1.00	1.00	
		Economic and Social Disadvantage	Cerebrovascular diseases	overall	PR (95%CI)	5	1		1.00	1.00	1.00	
		Economic and Social Disadvantage	Diseases of the circulatory system	overall	PR (95%CI)	6	1		1.00	1.00	1.00	
[56]	Cox, 2007	Economic and Social Disadvantage	Diabetes mellitus	overall	Negative binomial regression (SE)	1	1		0.09	not reported	not reported	0.01
		Economic and Social Disadvantage	Diabetes mellitus	overall	Negative binomial regression (SE)	2	1		-0.04	not reported	not reported	0.01
[57]	Cozier, 2007	Economic and Social Disadvantage	Hypertensive diseases	female	IRR (95%CI)	1	1	1 (lowest)	1.29	1.14	1.45	
						1	2	2	1.29	1.15	1.45	
						1	3	3	1.23	1.09	1.38	
						1	4	4	0.99	0.87	1.12	
						1	5	5 (highest median value, reference)	1.00	-	-	
[58]	Cromer, 2023	Economic and Social Disadvantage	Diabetes mellitus	overall	OR (95%CI)	1	1	per SD change in socioeconomic risk measures	1.29	1.23	1.35	
		Economic and Social Disadvantage	Diabetes mellitus	overall	OR (95%CI)	2	1	per SD change in socioeconomic risk measures	1.39	1.37	1.42	
[59]	Cubbin, 2006	Economic and Social Disadvantage	Hypertensive diseases	overall	OR (95%CI)	1	1	< 1 SD below mean (low deprivation)	0.68	not reported	not reported	
						1	2	Within 1 SD of mean (reference)	1.00	-	-	
						1	3	>1 SD above mean (high deprivation)	0.91	not reported	not reported	
		Economic and Social Disadvantage	Diabetes mellitus	overall	OR (95%CI)	2	1	< 1 SD below mean (low deprivation)	0.84	not reported	not reported	
						2	2	Within 1 SD of mean (reference)	1.00	-	-	
						2	3	>1 SD above mean (high deprivation)	1.04	not reported	not reported	
[60]	Cunningham, 2018	Economic and Social Disadvantage	Diabetes mellitus	overall	Incidence rate per 100,000 persons (95%CI)	1	1		38.57	29.93	47.21	

		Economic and Social Disadvantage	Diabetes mellitus	overall	Incidence rate per 100,000 persons (95%CI)	2	1		-1.75	-9.97	6.47
		Economic and Social Disadvantage	Diabetes mellitus	overall	Incidence rate per 100,000 persons (95%CI)	3	1		-5.00	-14.92	4.92
		Economic and Social Disadvantage	Diabetes mellitus	overall	Incidence rate per 100,000 persons (95%CI)	4	1		50.67	39.87	61.47
[61]	Cuthbertson, 2018	Economic and Social Disadvantage	Other forms of heart disease	overall	Incidence rate differences (95%CI)	1	1	low (reference)	0.00	-	-
						1	2	medium	2.41	1.43	3.40
						1	3	high	4.47	3.29	5.65
		Economic and Social Disadvantage	Other forms of heart disease	overall	Incidence rate differences (95%CI)	2	1	high	0.00	-	-
						2	2	medium	1.91	1.05	2.78
						2	3	low	3.73	2.63	4.82
		Economic and Social Disadvantage	Other forms of heart disease	overall	Incidence rate differences (95%CI)	4	1	low (reference)	0.00	-	-
						4	2	medium	0.89	0.21	1.56
						4	3	high	1.41	0.61	2.22
		Economic and Social Disadvantage	Other forms of heart disease	overall	Incidence rate differences (95%CI)	5	1	high	0.00	-	-
						5	2	medium	0.72	0.13	1.30
						5	3	low	1.72	0.97	2.47
[62]	de Oliveira, 2023	Economic and Social Disadvantage	Diabetes mellitus	overall	OR (95%CI)	1	1	High (reference)	1.00	-	-
						1	2	Middle	2.64	0.79	11.94
						1	3	Low	26.18	9.12	110.80
		Economic and Social Disadvantage	Diabetes mellitus	overall	OR (95%CI)	2	1	High (reference)	1.00	-	-
						2	2	Middle	17.20	6.86	57.79
						2	3	Low	136.10	53.16	463.62
[63]	de Silva, 2022	Economic and Social Disadvantage	Diabetes mellitus	overall	negative binomial regression	1	1	Low NSES (reference)	1.00	-	-
						1	2	Med. NSES	1.10	1.08	1.11
						1	3	High NSES	1.13	1.12	1.15
		Discrimination and Segregation	Diabetes mellitus	overall	negative binomial regression	2	1	Low % of non-hispanic Black (reference)	1.00	-	-
						2	2	Med. % of non-hispanic Black	0.97	0.96	0.98
						2	3	High % of non-hispanic Blac	1.04	1.03	1.06

[64]	Desmond, 2015	Economic and Social Disadvantage	Diabetes mellitus	overall	OR (95%CI)	1	1		1.31	not reported	not reported	0.17
		Economic and Social Disadvantage	Diabetes mellitus	overall	OR (95%CI)	2	1		0.92	not reported	not reported	0.13
[65]	Diez Roux, 2002	Economic and Social Disadvantage	Hypertensive diseases	overall	HR (95%CI)	1	1	Lowest socioeconomic category	1.09	0.96	1.24	
						1	2	Middle	1.07	0.95	1.21	
						1	3	Highest (reference)	1.00	-	-	
		Economic and Social Disadvantage	Hypertensive diseases	overall	HR (95%CI)	2	1	Lowest socioeconomic category	0.89	0.72	1.11	
						2	2	Middle	1.00	0.82	1.23	
						2	3	Highest (reference)	1.00	-	-	
[66]	Diez-Roux, 1997	Economic and Social Disadvantage	Ischaemic heart diseases	female	OR (95%CI)	1	1	(90th vs 10th; more-disadvantaged neighborhoods are the reference category)	1.88	1.00	3.32	
		Economic and Social Disadvantage	Ischaemic heart diseases	female	OR (95%CI)	2	1	(90th vs 10th; more-disadvantaged neighborhoods are the reference category)	1.61	1.11	2.87	
		Economic and Social Disadvantage	Ischaemic heart diseases	female	OR (95%CI)	3	1	(90th vs 10th; more-disadvantaged neighborhoods are the reference category)	2.17	1.20	3.94	
		Economic and Social Disadvantage	Ischaemic heart diseases	female	OR (95%CI)	4	1	(90th vs 10th; more-disadvantaged neighborhoods are the reference category)	2.82	1.29	6.16	
		Economic and Social Disadvantage	Ischaemic heart diseases	male	OR (95%CI)	5	1	(90th vs 10th; more-disadvantaged neighborhoods are the reference category)	0.82	0.55	1.17	
		Economic and Social Disadvantage	Ischaemic heart diseases	male	OR (95%CI)	6	1	(90th vs 10th; more-disadvantaged neighborhoods are the reference category)	1.17	0.88	1.53	
		Economic and Social Disadvantage	Ischaemic heart diseases	male	OR (95%CI)	7	1	(90th vs 10th; more-disadvantaged neighborhoods are the reference category)	1.15	0.88	1.50	
		Economic and Social Disadvantage	Ischaemic heart diseases	male	OR (95%CI)	8	1	(90th vs 10th; more-disadvantaged neighborhoods are the reference category)	1.26	0.92	1.71	
		Economic and Social Disadvantage	Ischaemic heart diseases	female	OR (95%CI)	9	1	(90th vs 10th; more-disadvantaged neighborhoods are the reference category)	1.02	0.48	2.29	
		Economic and Social Disadvantage	Ischaemic heart diseases	female	OR (95%CI)	10	1	(90th vs 10th; more-disadvantaged neighborhoods are the reference category)	0.91	0.43	1.92	
		Economic and Social Disadvantage	Ischaemic heart diseases	female	OR (95%CI)	11	1	(90th vs 10th; more-disadvantaged neighborhoods are the reference category)	1.21	0.55	2.68	
		Economic and Social Disadvantage	Ischaemic heart diseases	female	OR (95%CI)	12	1	(90th vs 10th; more-disadvantaged neighborhoods are the reference category)	1.42	0.36	5.59	
		Economic and Social Disadvantage	Ischaemic heart diseases	male	OR (95%CI)	13	1	(90th vs 10th; more-disadvantaged neighborhoods are the reference category)	0.92	0.45	1.82	

		Economic and Social Disadvantage	Ischaemic heart diseases	male	OR (95%CI)	14	1	(90th vs 10th; more-disadvantaged neighborhoods are the reference category)	0.76	0.41	1.38
		Economic and Social Disadvantage	Ischaemic heart diseases	male	OR (95%CI)	15	1	(90th vs 10th; more-disadvantaged neighborhoods are the reference category)	1.03	0.34	1.54
		Economic and Social Disadvantage	Ischaemic heart diseases	male	OR (95%CI)	16	1	(90th vs 10th; more-disadvantaged neighborhoods are the reference category)	1.08	0.30	2.36
[67]	Diez-Roux, 2000	Economic and Social Disadvantage	Hypertensive diseases	male	OR (95%CI)	1	1		1.22	0.89	1.68
		Economic and Social Disadvantage	Hypertensive diseases	male	OR (95%CI)	2	1		0.78	0.56	1.08
		Economic and Social Disadvantage	Hypertensive diseases	female	OR (95%CI)	3	1		1.61	1.17	2.21
		Economic and Social Disadvantage	Hypertensive diseases	female	OR (95%CI)	4	1		0.79	0.53	1.18
[68]	Djekic, 2018	Economic and Social Disadvantage	Hypertensive diseases	overall	%	1	1	high	0.04	not reported	not reported
						1	2	low	0.12	not reported	not reported
		Economic and Social Disadvantage	Diabetes mellitus	overall	%	2	1	high	0.37	not reported	not reported
						2	2	low	0.48	not reported	not reported
[69]	Dragano, 2007	Economic and Social Disadvantage	Hypertensive diseases	overall	OR (95%CI)	1	1	I (lowest unemployment, reference)	1.00	-	-
						1	2	II	1.02	0.90	1.17
						1	3	III	1.06	0.90	1.25
						1	4	IV	0.89	0.69	1.16
		Economic and Social Disadvantage	Hypertensive diseases	overall	OR (95%CI)	2	1	I (lowest unemployment, reference)	1.00	-	-
						2	2	II	1.06	0.91	1.23
						2	3	III	1.07	0.91	1.25
						2	4	IV	1.14	0.95	1.37
[70]	Dubowitz, 2012	Economic and Social Disadvantage	Hypertensive diseases	female	OR (95%CI)	1	1		0.99	0.99	1.00
[71]	Dwane, 2020	Economic and Social Disadvantage	Hypertensive diseases	overall	OR (95%CI)	1	1	I (least deprived)	0.80	0.30	1.60
						1	2	II	1.10	0.50	2.30
						1	3	III	1.10	0.60	2.00
						1	4	IV	1.20	0.70	2.30

						1	5	V (most deprived, reference)	1.00	-	-
		Economic and Social Disadvantage	Ischaemic heart diseases	overall	OR (95%CI)	2	1	I (least deprived)	1.50	0.50	4.20
						2	2	II	2.10	0.80	5.70
						2	3	III	1.80	0.80	4.20
						2	4	IV	1.50	0.70	3.50
						2	5	V (most deprived, reference)	1.00	-	-
		Economic and Social Disadvantage	Cerebrovascular diseases	overall	OR (95%CI)	3	1	I (least deprived)	1.50	0.60	3.90
						3	2	II	0.80	0.30	2.00
						3	3	III	1.30	0.60	2.70
						3	4	IV	1.30	0.60	2.70
						3	5	V (most deprived, reference)	1.00	-	-
[72]	Dyck, 2021	Discrimination and Segregation	Ischaemic heart diseases	overall	RR (95%CI)	1	1	Increase % of indigenous population (0.1%)	1.05	1.02	1.09
		Economic and Social Disadvantage	Ischaemic heart diseases	overall	RR (95%CI)	2	1	Increase of 1 in the deprivation index	1.07	0.99	1.13
[73]	Ekholuenetale, 2020	Economic and Social Disadvantage	Hypertensive diseases	female	OR (95%CI)	1	1	I (lowest disadvantage, reference)	1.00	-	-
						1	2	II	0.89	0.67	1.20
						1	3	III (highest)	0.70	0.50	0.99
[74]	Eng, 2002	Social Relationships and Norms	Ischaemic heart diseases	male	RR (95%CI)	1	1	IV (high, reference)	1.00	-	-
						1	2	III	1.18	0.96	1.44
						1	3	II	0.86	0.70	1.06
						1	4	I (low social integration)	1.11	0.80	1.53
[75]	Engström, 2001	Economic and Social Disadvantage	Hypertensive diseases	male	correlation	1	1		-0.44	not reported	not reported
		Economic and Social Disadvantage	Diabetes mellitus	male	correlation	2	1		-0.42	not reported	not reported
		Economic and Social Disadvantage	Hypertensive diseases	female	correlation	3	1		-0.51	not reported	not reported
		Economic and Social Disadvantage	Diabetes mellitus	female	correlation	4	1		-0.62	not reported	not reported
[76]	Eschbach, 2004	Discrimination and Segregation	Cerebrovascular diseases	overall	OR (95%CI)	1	1		0.33	0.16	0.71
		Discrimination and Segregation	Ischaemic heart diseases	overall	OR (95%CI)	2	1		0.82	0.38	1.76
		Discrimination and Segregation	Hypertensive diseases	overall	OR (95%CI)	3	1		0.84	0.49	1.44

		Discrimination and Segregation	Diabetes mellitus	overall	OR (95%CI)	4	1		1.24	0.70	2.23
[77]	Essien, 2022	Economic and Social Disadvantage	Other forms of heart disease	overall	OR (95%CI)	1	1	low neighborhood-level poverty (reference)	1.00	-	-
						1	2	intermediate neighborhood-level poverty	1.21	1.00	1.46
						1	3	high neighborhood-level poverty	1.23	0.96	1.57
[78]	Exeter, 2015	Economic and Social Disadvantage	Diseases of the circulatory system	overall	RR (95%CI)	1	1	stayers (reference)	1.00	-	-
						1	2	move upward	1.27	1.22	1.32
						1	3	move within same quintile	1.19	1.14	1.24
						1	4	move downward	1.24	1.19	1.30
[79]	Faka, 2018	Economic and Social Disadvantage	Diabetes mellitus	overall	Spatial quasi-Poisson coefficient (95%CI)	1	1		-0.04	-0.09	0.00
[80]	Feero, 1995	Economic and Social Disadvantage	Other forms of heart disease	overall	Rates (95%CI)	1	1	high-income census tract cardiac arrest rate	0.47	not reported	not reported
						1	2	low-income census tract cardiac arrest rate	0.85	not reported	not reported
[81]	Ferguson, 2020	Economic and Social Disadvantage	Hypertensive diseases	male	OR (95%CI)	1	1	T1 (lower SES, reference)	1.00	-	-
						1	2	T2	0.81	0.58	1.13
						1	3	T3 (upper)	0.67	0.44	1.00
		Economic and Social Disadvantage	Hypertensive diseases	male	OR (95%CI)	2	1	T1 (lower SES, reference)	1.00	-	-
						2	2	T2	1.02	0.70	1.49
						2	3	T3 (upper)	0.85	0.59	1.24
		Economic and Social Disadvantage	Hypertensive diseases	female	OR (95%CI)	3	1	T1 (lower SES, reference)	1.00	-	-
						3	2	T2	0.94	0.65	1.37
						3	3	T3 (upper)	1.08	0.67	1.74
		Economic and Social Disadvantage	Hypertensive diseases	female	OR (95%CI)	4	1	T1 (lower SES, reference)	1.00	-	-
						4	2	T2	1.21	0.79	1.86
						4	3	T3 (upper)	1.10	0.70	1.74
[82]	Fitzpatrick, 2020	Economic and Social Disadvantage	Diabetes mellitus	overall	Beta (95%CI)	1	1	median home value	0.00	0.00	0.00
		Economic and Social Disadvantage	Diabetes mellitus	overall	Beta (95%CI)	2	1	% with no high school education	1.41	0.90	1.91
		Economic and Social Disadvantage	Diabetes mellitus	overall	Beta (95%CI)	3	1	% households receiving SNAP without any children under the age of 18	0.06	0.05	0.07

		Economic and Social Disadvantage	Diabetes mellitus	overall	Beta (95%CI)	4	1	Gini index of inequality	0.09	0.08	0.09	
		Economic and Social Disadvantage	Cerebrovascular diseases	overall	Beta (95%CI)	5	1	median home value	0.00	0.00	0.00	
		Economic and Social Disadvantage	Cerebrovascular diseases	overall	Beta (95%CI)	6	1	% with no high school education	6.66	5.66	7.65	
		Economic and Social Disadvantage	Cerebrovascular diseases	overall	Beta (95%CI)	7	1	% households receiving SNAP without any children under the age of 18	0.09	0.08	0.10	
		Economic and Social Disadvantage	Cerebrovascular diseases	overall	Beta (95%CI)	8	1	Gini index of inequality	-0.04	-0.04	-0.03	
		Economic and Social Disadvantage	Hypertensive diseases	overall	Beta (95%CI)	9	1	median home value	0.00	0.00	0.00	
		Economic and Social Disadvantage	Hypertensive diseases	overall	Beta (95%CI)	10	1	% with no high school education	0.96	0.80	1.13	
		Economic and Social Disadvantage	Hypertensive diseases	overall	Beta (95%CI)	11	1	% households receiving SNAP without any children under the age of 18	0.03	0.03	0.03	
		Economic and Social Disadvantage	Hypertensive diseases	overall	Beta (95%CI)	12	1	Gini index of inequality	0.01	0.01	0.01	
[83]	Fleischer, 2008	Economic and Social Disadvantage	Hypertensive diseases	overall	OR (95%CI)	1	1		0.99	0.85	1.16	
		Economic and Social Disadvantage	Diabetes mellitus	overall	OR (95%CI)	2	1		1.07	0.85	1.35	
[84]	Ford, 2006	Social Relationships and Norms	Hypertensive diseases	male	%	1	1	0-1 (few ties)	33.10	not reported	not reported	1.50
						1	2	2	31.60	not reported	not reported	1.60
						1	3	3	26.90	not reported	not reported	1.30
						1	4	4 (most ties)	27.80	not reported	not reported	2.70
		Social Relationships and Norms	Hypertensive diseases	female	%	2	1	0-1 (few ties)	30.80	not reported	not reported	1.80
						2	2	2	28.80	not reported	not reported	1.00
						2	3	3	25.60	not reported	not reported	1.00
						2	4	4 (most ties)	21.90	not reported	not reported	1.70
		Social Relationships and Norms	Diabetes mellitus	male	%	3	1	0-1 (few ties)	6.60	not reported	not reported	0.70
						3	2	2	4.00	not reported	not reported	0.50

						3	3	3	6.60	not reported	not reported	0.70
						3	4	4 (most ties)	4.70	not reported	not reported	0.70
		Social Relationships and Norms	Diabetes mellitus	female	%	4	1	0-1 (few ties)	7.50	not reported	not reported	0.90
						4	2	2	6.80	not reported	not reported	0.70
						4	3	3	5.20	not reported	not reported	0.60
						4	4	4 (most ties)	3.90	not reported	not reported	0.70
[85]	Forsberg, 2018	Economic and Social Disadvantage	Ischaemic heart diseases	male	HR (95%CI)	1	1		1.07	1.06	1.09	
		Economic and Social Disadvantage	Ischaemic heart diseases	male	HR (95%CI)	2	1		1.05	1.05	1.06	
		Economic and Social Disadvantage	Ischaemic heart diseases	male	HR (95%CI)	3	1		1.04	1.04	1.05	
		Economic and Social Disadvantage	Ischaemic heart diseases	female	HR (95%CI)	4	1		1.11	1.09	1.13	
		Economic and Social Disadvantage	Ischaemic heart diseases	female	HR (95%CI)	5	1		1.10	1.09	1.11	
		Economic and Social Disadvantage	Ischaemic heart diseases	female	HR (95%CI)	6	1		1.08	1.07	1.09	
		Economic and Social Disadvantage	Cerebrovascular diseases	male	HR (95%CI)	7	1		1.04	1.02	1.06	
		Economic and Social Disadvantage	Cerebrovascular diseases	male	HR (95%CI)	8	1		1.03	1.02	1.04	
		Economic and Social Disadvantage	Cerebrovascular diseases	male	HR (95%CI)	9	1		1.02	1.01	1.03	
		Economic and Social Disadvantage	Cerebrovascular diseases	female	HR (95%CI)	10	1		1.07	1.05	1.09	
		Economic and Social Disadvantage	Cerebrovascular diseases	female	HR (95%CI)	11	1		1.04	1.03	1.05	
		Economic and Social Disadvantage	Cerebrovascular diseases	female	HR (95%CI)	12	1		1.04	1.03	1.05	
[86]	Forsberg, 2023	Economic and Social Disadvantage	Ischaemic heart diseases	male	OR (95%CI)	1	1	high (reference)	1.00	-	-	
						1	2	middle	1.06	1.01	1.12	
						1	3	low	1.11	1.05	1.17	

		Economic and Social Disadvantage	Ischaemic heart diseases	male	OR (95%CI)	2	1	high (reference)	1.00	-	-
						2	2	middle	1.07	1.00	1.15
						2	3	low	1.02	0.94	1.12
		Economic and Social Disadvantage	Ischaemic heart diseases	male	OR (95%CI)	3	1	high (reference)	1.00	-	-
						3	2	middle	1.13	1.08	1.18
						3	3	low	1.21	1.13	1.29
		Economic and Social Disadvantage	Ischaemic heart diseases	female	OR (95%CI)	4	1	high (reference)	1.00	-	-
						4	2	middle	1.04	0.96	1.12
						4	3	low	1.13	1.05	1.22
		Economic and Social Disadvantage	Ischaemic heart diseases	female	OR (95%CI)	5	1	high (reference)	1.00	-	-
						5	2	middle	1.03	0.94	1.14
						5	3	low	1.03	0.91	1.16
		Economic and Social Disadvantage	Ischaemic heart diseases	female	OR (95%CI)	6	1	high (reference)	1.00	-	-
						6	2	middle	1.12	1.05	1.20
						6	3	low	1.35	1.23	1.48
[87]	Freedman, 2011	Economic and Social Disadvantage	Other forms of heart disease	male	OR (95%CI)	1	1		0.98	0.79	1.22
		Economic and Social Disadvantage	Other forms of heart disease	male	OR (95%CI)	2	1		0.94	0.80	1.12
		Discrimination and Segregation	Other forms of heart disease	male	OR (95%CI)	3	1		1.00	0.82	1.22
		Crime and Safety	Other forms of heart disease	male	OR (95%CI)	4	1		1.01	0.87	1.17
		Economic and Social Disadvantage	Other forms of heart disease	female	OR (95%CI)	5	1		1.20	1.00	1.43
		Economic and Social Disadvantage	Other forms of heart disease	female	OR (95%CI)	6	1		0.95	0.81	1.11
		Discrimination and Segregation	Other forms of heart disease	female	OR (95%CI)	7	1		1.04	0.87	1.24
		Crime and Safety	Other forms of heart disease	female	OR (95%CI)	8	1		1.06	0.93	1.22
		Economic and Social Disadvantage	Hypertensive diseases	male	OR (95%CI)	9	1		1.06	0.85	1.32
		Economic and Social Disadvantage	Hypertensive diseases	male	OR (95%CI)	10	1		1.11	0.94	1.30

		Discrimination and Segregation	Hypertensive diseases	male	OR (95%CI)	11	1		1.15	0.95	1.39
		Crime and Safety	Hypertensive diseases	male	OR (95%CI)	12	1		1.15	0.99	1.33
		Economic and Social Disadvantage	Hypertensive diseases	female	OR (95%CI)	13	1		1.13	0.95	1.34
		Economic and Social Disadvantage	Hypertensive diseases	female	OR (95%CI)	14	1		0.92	0.81	1.05
		Discrimination and Segregation	Hypertensive diseases	female	OR (95%CI)	15	1		0.94	0.80	1.10
		Crime and Safety	Hypertensive diseases	female	OR (95%CI)	16	1		1.05	0.94	1.18
		Economic and Social Disadvantage	Cerebrovascular diseases	male	OR (95%CI)	17	1		0.81	0.57	1.14
		Economic and Social Disadvantage	Cerebrovascular diseases	male	OR (95%CI)	18	1		0.92	0.70	1.21
		Discrimination and Segregation	Cerebrovascular diseases	male	OR (95%CI)	19	1		1.11	0.83	1.49
		Crime and Safety	Cerebrovascular diseases	male	OR (95%CI)	20	1		1.05	0.84	1.32
		Economic and Social Disadvantage	Cerebrovascular diseases	female	OR (95%CI)	21	1		0.78	0.60	1.02
		Economic and Social Disadvantage	Cerebrovascular diseases	female	OR (95%CI)	22	1		0.85	0.66	1.08
		Discrimination and Segregation	Cerebrovascular diseases	female	OR (95%CI)	23	1		1.08	0.85	1.37
		Crime and Safety	Cerebrovascular diseases	female	OR (95%CI)	24	1		1.16	0.96	1.40
		Economic and Social Disadvantage	Diabetes mellitus	male	OR (95%CI)	25	1		0.97	0.75	1.25
		Economic and Social Disadvantage	Diabetes mellitus	male	OR (95%CI)	26	1		0.88	0.71	1.11
		Discrimination and Segregation	Diabetes mellitus	male	OR (95%CI)	27	1		1.01	0.80	1.28
		Crime and Safety	Diabetes mellitus	male	OR (95%CI)	28	1		0.97	0.81	1.16
		Economic and Social Disadvantage	Diabetes mellitus	female	OR (95%CI)	29	1		0.99	0.80	1.22
		Economic and Social Disadvantage	Diabetes mellitus	female	OR (95%CI)	30	1		0.86	0.69	1.07
		Discrimination and Segregation	Diabetes mellitus	female	OR (95%CI)	31	1		0.82	0.66	1.02
		Crime and Safety	Diabetes mellitus	female	OR (95%CI)	32	1		1.07	0.91	1.26
[88]	Gao, 2022	Discrimination and Segregation	Hypertensive diseases	overall	HR (95%CI)	1	1	segregated neighborhood	1.31	1.08	1.58

						1	2	nonsegregated neighborhoods (reference)	1.00	-	-
		Discrimination and Segregation	Hypertensive diseases	overall	HR (95%CI)	2	1	segregated neighborhood	1.32	1.05	1.66
						2	2	nonsegregated neighborhoods (reference)	1.00	-	-
		Discrimination and Segregation	Hypertensive diseases	overall	HR (95%CI)	3	1	segregated neighborhood	1.12	0.78	1.61
						3	2	nonsegregated neighborhoods (reference)	1.00	-	-
[89]	Garcia, 2015	Economic and Social Disadvantage	Diabetes mellitus	overall	OR (95%CI)	1	1		0.76	0.61	0.93
[90]	Garcia, 2016	Economic and Social Disadvantage	Diabetes mellitus	overall	HR (95%CI)	1	1		1.66	1.14	2.42
		Economic and Social Disadvantage	Diabetes mellitus	overall	HR (95%CI)	2	1		0.83	0.62	1.10
[91]	Gary-Webb, 2023	Crime and Safety	Diabetes mellitus	female	PR (95%CI)	1	1	lower tertile (reference)	1.00	-	-
						1	2	Middle tertile	1.65	0.98	2.78
						1	3	Upper tertile	1.48	0.70	3.14
		Crime and Safety	Hypertensive diseases	female	PR (95%CI)	2	1	lower tertile (reference)	1.00	-	-
						2	2	Middle tertile	1.17	0.78	1.73
						2	3	Upper tertile	0.90	0.59	1.38
		Crime and Safety	Diabetes mellitus	female	PR (95%CI)	3	1	lower tertile (reference)	1.00	-	-
						3	2	Middle tertile	1.34	0.74	2.43
						3	3	Upper tertile	1.31	0.69	2.51
		Crime and Safety	Hypertensive diseases	female	PR (95%CI)	4	1	lower tertile (reference)	1.00	-	-
						4	2	Middle tertile	1.14	0.84	1.56
						4	3	Upper tertile	1.28	0.92	1.78
		Crime and Safety	Diabetes mellitus	male	PR (95%CI)	5	1	lower tertile (reference)	1.00	-	-
						5	2	Middle tertile	1.15	0.49	2.71
						5	3	Upper tertile	0.80	0.31	2.10
		Crime and Safety	Hypertensive diseases	male	PR (95%CI)	6	1	lower tertile (reference)	1.00	-	-
						6	2	Middle tertile	1.51	0.96	2.37
						6	3	Upper tertile	0.80	0.52	1.24
		Crime and Safety	Diabetes mellitus	male	PR (95%CI)	7	1	lower tertile (reference)	1.00	-	-
						7	2	Middle tertile	0.70	0.35	1.42
						7	3	Upper tertile	0.69	0.29	1.67
		Crime and Safety	Hypertensive diseases	male	PR (95%CI)	8	1	lower tertile (reference)	1.00	-	-
						8	2	Middle tertile	0.95	0.66	1.37

						8	3	Upper tertile	0.79	0.49	1.27
[92]	Gaskin, 2014	Economic and Social Disadvantage	Diabetes mellitus	overall	OR (95%CI)	1	1	nonpoor neighborhood (reference)	1.00	-	-
						1	2	poor neighborhood	1.13	0.75	1.72
		Discrimination and Segregation	Diabetes mellitus	overall	OR (95%CI)	2	1	White in predominantly White neighborhood (reference)	1.00	-	-
						2	2	White in predominantly Black neighborhood	1.70	0.24	11.87
						2	3	White in other race neighborhood	1.32	0.34	5.11
						2	4	White in integrated neighborhood	1.32	0.78	2.24
						2	5	Black in predominantly Black neighborhood	1.44	0.92	2.25
						2	6	Black in predominantly White neighborhood	1.78	0.87	3.66
						2	7	Black in other race neighborhood	1.30	0.31	5.55
						2	8	Black in integrated neighborhood	2.13	1.26	3.60
[93]	Gebreab, 2017	Social Cohesion and Social Capital	Diabetes mellitus	overall	PR (95%CI)	1	1		0.93	0.86	0.99
		Crime and Safety	Diabetes mellitus	overall	PR (95%CI)	2	1		1.10	1.02	1.18
		Social Cohesion and Social Capital	Diabetes mellitus	overall	HR (95%CI)	3	1		0.85	0.67	1.09
		Crime and Safety	Diabetes mellitus	overall	HR (95%CI)	4	1		1.13	0.91	1.40
[94]	Gero, 2022	Crime and Safety	Diabetes mellitus	overall	OR (95%CI)	1	1	1-SD increase (in crime rates)	0.96	0.88	1.05
		Crime and Safety	Hypertensive diseases		OR (95%CI)	2	1	1-SD increase (in crime rates)	1.09	1.02	1.17
[95]	Glover, 2019	Social Relationships and Norms	Diabetes mellitus	female	PR (95%CI)	1	1	Low (reference)	1.00	-	-
						1	2	High	1.02	0.88	1.18
		Social Relationships and Norms	Diabetes mellitus	female	PR (95%CI)	2	1		0.97	0.91	1.04
		Social Relationships and Norms	Diabetes mellitus	male	PR (95%CI)	3	1	Low (reference)	1.00	-	-
						3	2	High	0.69	0.56	0.86
		Social Relationships and Norms	Diabetes mellitus	male	PR (95%CI)	4	1		0.83	0.75	0.92
		Social Relationships and Norms	Diabetes mellitus	female	PR (95%CI)	5	1	Low (reference)	1.00	-	-
						5	2	High	0.83	0.72	0.96

		Social Relationships and Norms	Diabetes mellitus	female	PR (95%CI)	6	1		0.94	0.89	0.99
		Social Relationships and Norms	Diabetes mellitus	male	PR (95%CI)	7	1	Low (reference)	1.00	-	-
						7	2	High	0.92	0.70	1.20
		Social Relationships and Norms	Diabetes mellitus	male	PR (95%CI)	8	1		0.87	0.77	0.98
[96]	Grundmann, 2014	Economic and Social Disadvantage	Diabetes mellitus	overall	OR (95%CI)	1	1	Q1 (least deprived, reference)	1.00	-	-
						1	2	Q2	1.19	0.98	1.44
						1	3	Q3	1.22	1.01	1.48
						1	4	Q4	1.18	0.98	1.42
						1	5	Q5 (most deprived)	1.37	1.13	1.65
[97]	Guion, 2024	Economic and Social Disadvantage	Diabetes mellitus	male	Relative Risk (95%credibility interval)	1	1	Q1 (least deprived, reference)	0.99	0.98	0.99
						1	2	Q2	0.99	0.99	0.99
						1	3	Q3	0.99	0.99	0.99
						1	4	Q4	0.91	0.99	0.99
						1	5	Q5	0.99	0.99	1.00
		Economic and Social Disadvantage	Diabetes mellitus	female	Relative Risk (95%credibility interval)	2	1	Q1 (least deprived, reference)	0.98	0.98	0.99
						2	2	Q2	0.98	0.98	0.99
						2	3	Q3	0.98	0.98	0.99
						2	4	Q4	0.98	0.98	0.99
						2	5	Q5	0.99	0.99	0.99
[98]	Gwon, 2020	Economic and Social Disadvantage	Ischaemic heart diseases	overall	HR (95%CI)	1	1	Low	1.16	1.01	1.32
						1	2	Medium	1.03	0.88	1.22
						1	3	High SES (reference)	1.00	-	-
[99]	Halonen, 2015	Economic and Social Disadvantage	Hypertensive diseases	overall	RR (95%CI)	1	1	low childhood adversity-low adult disadvantage (reference)	1.00	-	-
						1	2	low childhood adversity-high adult disadvantage	1.11	1.07	1.15
						1	3	high childhood adversity-low adult disadvantage	1.07	1.03	1.11
						1	4	high childhood adversity-high adult disadvantage	1.15	1.09	1.20
		Economic and Social Disadvantage	Diabetes mellitus	overall	RR (95%CI)	2	1	low childhood adversity-low adult disadvantage (reference)	1.00	-	-
						2	2	low childhood adversity-high adult disadvantage	1.26	1.14	1.39

						2	3	high childhood adversity-low adult disadvantage	1.08	0.96	1.22
						2	4	high childhood adversity-high adult disadvantage	1.53	1.37	1.70
[100]	Hamad, 2020	Economic and Social Disadvantage	Hypertensive diseases	overall	Beta (95%CI)	1	1		0.71	0.30	1.13
		Economic and Social Disadvantage	Diabetes mellitus	overall	Beta (95%CI)	2	1		0.45	0.09	0.81
		Economic and Social Disadvantage	Ischaemic heart diseases	overall	Beta (95%CI)	3	1		0.14	0.03	0.25
		Economic and Social Disadvantage	Cerebrovascular diseases	overall	Beta (95%CI)	4	1		0.01	-0.16	0.17
[101]	Hanefeld, 2018	Economic and Social Disadvantage	Diseases of the circulatory system	overall	IRR (95%CI)	1	1	<5%	0.86	0.81	0.92
						1	2	5 to <6%	0.95	0.89	1.02
						1	3	6 to <7.5%	1.00	0.93	1.06
						1	4	7.5 to <9.5%	1.06	0.97	1.15
						1	5	9.5% or more	1.19	1.11	1.27
		Economic and Social Disadvantage	Diseases of the circulatory system	overall	IRR (95%CI)	2	1	<5%	0.90	0.81	1.00
						2	2	5 to <6%	0.96	0.85	1.07
						2	3	6 to <7.5%	0.96	0.86	1.06
						2	4	7.5 to <9.5%	1.05	0.91	1.20
						2	5	9.5% or more	1.18	1.05	1.31
		Economic and Social Disadvantage	Cerebrovascular diseases	overall	IRR (95%CI)	3	1	<5%	0.89	0.79	0.99
						3	2	5 to <6%	1.00	0.87	1.11
						3	3	6 to <7.5%	1.00	0.89	1.11
						3	4	7.5 to <9.5%	1.06	0.90	1.22
						3	5	9.5% or more	1.12	0.99	1.26
[102]	Hanigan, 2017	Crime and Safety	Diabetes mellitus	overall	Beta (95%CI)	1	1		0.24	0.10	0.39
		Crime and Safety	Diabetes mellitus	overall	Beta (95%CI)	2	1		0.36	0.18	0.53
		Crime and Safety	Diabetes mellitus	overall	Beta (95%CI)	3	1		0.32	0.10	0.54
		Crime and Safety	Diabetes mellitus	overall	Beta (95%CI)	4	1		0.33	0.04	0.62
[103]	Harding, 2022	Social Relationships and Norms	Hypertensive diseases	overall	IRR (Incidence rate ratio)	1	1	low level structural support (reference)	1.00	-	-
					IRR (95%CI)	1	2	high level structural support	1.06	0.90	1.24

		Social Relationships and Norms	Hypertensive diseases	overall	IRR (95%CI)	2	1	per 5 point increase in structural social support	1.03	0.96	1.11
		Social Relationships and Norms	Hypertensive diseases	overall	IRR (95%CI)	3	1	low level functional support (reference)	1.00	-	-
					IRR (95%CI)	3	2	high level functional support	0.65	0.42	0.98
		Social Relationships and Norms	Hypertensive diseases	overall	IRR (95%CI)	4	1	per 3 point increase in functional social support	0.97	0.89	1.05
[104]	Hashemi, 2023	Economic and Social Disadvantage	Diabetes mellitus	overall	OR (95%CI)	1	1	most affluent	1.39	1.21	1.60
						1	2	affluent	1.25	1.08	1.46
						1	3	moderate	0.98	0.84	1.14
						1	4	Deprived	0.93	0.78	1.11
						1	5	most deprived (reference)	1.00	-	-
[105]	Hassen, 2020	Social Relationships and Norms	Diseases of the circulatory system	overall	OR (95%CI)	1	1	No (reference)	1.00	-	-
						1	2	Yes	0.63	0.58	0.69
[106]	Hawkins, 2012	Economic and Social Disadvantage	Other forms of heart disease	overall	IRR (95%CI)	1	1	Q5 (most deprived) vs. Q1 (most affluent)	2.33	1.83	2.96
		Economic and Social Disadvantage	Other forms of heart disease	overall	PRR (95%CI)	2	1	Q5 vs. Q1	1.95	1.82	2.09
[107]	Heeley, 2011	Economic and Social Disadvantage	Cerebrovascular diseases	overall	Relative difference in incidence rates (95%CI)	1	1		70% higher	47.00	95.00
		Economic and Social Disadvantage	Cerebrovascular diseases	male	Relative difference in incidence rates (95%CI)	2	1		0.58	30.00	93.00
		Economic and Social Disadvantage	Cerebrovascular diseases	female	Relative difference in incidence rates (95%CI)	3	1		0.87	51.00	130.00
[108]	Hendryx, 2020	Social Relationships and Norms	Diabetes mellitus	female	HR (95%CI)	1	1	1 (reference)	1.00	-	-
						1	2	2	0.91	0.88	0.95
						1	3	3	0.85	0.82	0.89
						1	4	4 (greater social support)	0.83	0.79	0.86
		Social Relationships and Norms	Diabetes mellitus	female	HR (95%CI)	2	1		0.99	0.99	0.99
		Social Relationships and Norms	Diabetes mellitus	female	HR (95%CI)	3	1	1	1.00	-	-
						3	2	2	1.03	0.98	1.08
						3	3	3	1.08	1.04	1.13
						3	4	4	1.21	1.17	1.26

		Social Relationships and Norms	Diabetes mellitus	female	HR (95%CI)	4	1		1.04	1.03	1.05
		Social Relationships and Norms	Diabetes mellitus	female	HR (95%CI)	5	1	1	1.00	-	-
						5	2	2	0.95	0.91	0.99
						5	3	3	0.97	0.93	1.01
						5	4	4	0.95	0.91	0.99
		Social Relationships and Norms	Diabetes mellitus	female	HR (95%CI)	6	1		0.99	0.98	1.00
[109]	Henriksson, 2010	Economic and Social Disadvantage	Ischaemic heart diseases	overall	RR (95%CI)	1	1		0.75	0.65	0.87
[110]	Heredia, 2022	Crime and Safety	Diabetes mellitus	overall	OR (95%CI)	1	1	Crime	1.02	0.63	1.65
						1	2	No Crime (reference)	1.00	-	-
[111]	Herrera-Añazco, 2019	Economic and Social Disadvantage	Hypertensive diseases	overall	Beta (95%CI)	1	1		-12.30	-23.10	-1.50
		Economic and Social Disadvantage	Hypertensive diseases	overall	Beta (95%CI)	2	1		0.50	-51.00	52.10
		Economic and Social Disadvantage	Hypertensive diseases	overall	Beta (95%CI)	3	1		-1.50	-39.30	36.40
		Economic and Social Disadvantage	Hypertensive diseases	overall	Beta (95%CI)	4	1		-10.30	-65.70	45.10
		Economic and Social Disadvantage	Hypertensive diseases	overall	Beta (95%CI)	5	1		-36.10	-81.80	9.70
		Economic and Social Disadvantage	Hypertensive diseases	overall	Beta (95%CI)	6	1		-362.70	-681.60	-43.80
[112]	Herrick, 2016	Economic and Social Disadvantage	Diabetes mellitus	overall	OR (95%CI)	1	1		1.25	1.13	1.37
[113]	Hilding, 2015	Social Relationships and Norms	Diabetes mellitus	female	OR (95%CI)	1	1	low (reference)	1.00	-	-
						1	2	middle	0.91	0.46	1.80
						1	3	high	0.50	0.22	1.16
		Civic Participation and Engagement	Diabetes mellitus	female	OR (95%CI)	2	1	no (reference)	1.00	-	-
						2	2	yes	0.59	0.31	1.13
		Social Relationships and Norms	Diabetes mellitus	male	OR (95%CI)	3	1	low (reference)	1.00	-	-
						3	2	middle	2.46	1.40	4.32

						3	3	high	1.93	1.03	3.60
		Civic Participation and Engagement	Diabetes mellitus	male	OR (95%CI)	4	1	no (reference)	1.00	-	-
						4	2	yes	1.13	0.66	1.92
[114]	Hill, 2014	Social Relationships and Norms	Hypertensive diseases	overall	OR (95%CI)	1	1		0.99	0.97	1.01
		Social Relationships and Norms	Hypertensive diseases	overall	OR (95%CI)	2	1		0.97	0.89	1.07
		Social Relationships and Norms	Hypertensive diseases	overall	OR (95%CI)	3	1		0.89	0.78	1.02
		Social Relationships and Norms	Hypertensive diseases	overall	OR (95%CI)	4	1		1.24	1.03	1.50
		Social Relationships and Norms	Diseases of the circulatory system	overall	OR (95%CI)	5	1		0.99	0.97	1.00
		Social Relationships and Norms	Diseases of the circulatory system	overall	OR (95%CI)	6	1		0.96	0.87	1.05
		Social Relationships and Norms	Diseases of the circulatory system	overall	OR (95%CI)	7	1		0.93	0.81	1.07
		Social Relationships and Norms	Diseases of the circulatory system	overall	OR (95%CI)	8	1		1.13	0.93	1.37
		Social Relationships and Norms	Cerebrovascular diseases	overall	OR (95%CI)	9	1		0.99	0.96	1.02
		Social Relationships and Norms	Cerebrovascular diseases	overall	OR (95%CI)	10	1		0.97	0.84	1.12
		Social Relationships and Norms	Cerebrovascular diseases	overall	OR (95%CI)	11	1		0.81	0.67	0.99
		Social Relationships and Norms	Cerebrovascular diseases	overall	OR (95%CI)	12	1		1.22	0.93	1.61
		Social Relationships and Norms	Diabetes mellitus	overall	OR (95%CI)	13	1		0.99	0.97	1.01
		Social Relationships and Norms	Diabetes mellitus	overall	OR (95%CI)	14	1		1.02	0.92	1.13
		Social Relationships and Norms	Diabetes mellitus	overall	OR (95%CI)	15	1		0.98	0.84	1.13
		Social Relationships and Norms	Diabetes mellitus	overall	OR (95%CI)	16	1		1.29	1.06	1.58
[115]	Höfelmann, 2012	Economic and Social Disadvantage	Hypertensive diseases	overall	OR (95%CI)	1	1	lower tertile (reference)	1.00	-	-

						1	2	intermediary	0.66	0.64	0.69
						1	3	higher income	0.54	0.52	0.56
[116]	Holstiege, 2019	Economic and Social Disadvantage	Other forms of heart disease	overall	OR (95%CI)	1	1	Lowest deprivation (reference)	1.00	-	-
						1	2	Low deprivation	1.10	0.97	1.24
						1	3	Medium deprivation	1.14	1.01	1.29
						1	4	High deprivation	1.24	1.10	1.40
						1	5	Highest deprivation	1.20	1.06	1.36
[117]	Holtgrave, 2006	Social Cohesion and Social Capital	Diabetes mellitus	overall	correlation	1	1		-0.67	not reported	not reported
		Economic and Social Disadvantage	Diabetes mellitus	overall	correlation	2	1		0.38	not reported	not reported
		Economic and Social Disadvantage	Diabetes mellitus	overall	correlation	3	1		0.24	not reported	not reported
[118]	Honda, 2021	Social Relationships and Norms	Diseases of arteries, arterioles and capillaries	overall	HR (95%CI)	1	1	Q1 high social support (reference)	1.00	-	-
						1	2	Q2	0.86	0.62	1.19
						1	3	Q3	0.98	0.71	1.33
						1	4	Q4 low social support	1.40	1.05	1.87
[119]	Honjo, 2015	Economic and Social Disadvantage	Cerebrovascular diseases	overall	HR (95%CI)	1	1	0 (reference, least deprived)	1.00	-	-
						1	2	1	1.16	1.04	1.29
						1	3	2	1.12	1.00	1.26
						1	4	3	1.18	1.02	1.35
						1	5	4 (most deprived)	1.19	1.01	1.41
[120]	Horsten, 1999	Social Relationships and Norms	Hypertensive diseases	female	%	1	1	% of participants in Quartile 1, with hypertension (%)	19.00	not reported	not reported
						1	2	% of participants in Quartile 1, no hypertension (%)	81.00	not reported	not reported
						1	3	% of participants in Quartile 2 and 3, with hypertension (%)	8.00	not reported	not reported
						1	4	% of participants in Quartile 2 and 3, no hypertension (%)	92.00	not reported	not reported
						1	5	% of participants in Quartile 4, with hypertension (%)	7.00	not reported	not reported
						1	6	% of participants in Quartile 4, no hypertension (%)	93.00	not reported	not reported
[121]	Hosseini, 2020	Social Relationships and Norms	Hypertensive diseases	female	OR (95%CI)	1	1	Q4 (largest, reference)	1.00	-	-
						1	2	Q3	1.01	0.91	1.11
						1	3	Q2	0.97	0.87	1.07

						1	4	Q1, smallest	1.15	1.03	1.28
		Civic Participation and Engagement	Hypertensive diseases	female	OR (95%CI)	2	1	a lot (reference)	1.00	-	-
						2	2	some	1.04	0.96	1.12
						2	3	a few	1.22	1.09	1.36
						2	4	none	1.30	0.94	1.79
		Social Relationships and Norms	Hypertensive diseases	male	OR (95%CI)	3	1	Q4 (largest, reference)	1.00	-	-
						3	2	Q3	0.97	0.88	1.07
						3	3	Q2	0.97	0.88	1.08
						3	4	Q1, smallest	1.04	0.94	1.16
		Civic Participation and Engagement	Hypertensive diseases	male	OR (95%CI)	4	1	a lot (reference)	1.00	-	-
						4	2	some	1.01	0.93	1.10
						4	3	a few	1.09	0.98	1.21
						4	4	none	1.07	0.80	1.42
[122]	Howard, 2016	Economic and Social Disadvantage	Cerebrovascular diseases	overall	HR (95%CI)	1	1	Q4, highest (reference)	1.00	-	-
						1	2	Q3	1.15	0.94	1.40
						1	3	Q2	1.16	0.95	1.44
						1	4	Q1	1.25	0.99	1.56
[123]	Hu, 2020	Economic and Social Disadvantage	Cerebrovascular diseases	overall	Change in the 90th quantile of the distribution	1	1		-0.57	-0.61	-0.51
		Economic and Social Disadvantage	Cerebrovascular diseases	overall	Change in the 90th quantile of the distribution	2	1		-0.62	-0.66	-0.57
		Economic and Social Disadvantage	Cerebrovascular diseases	overall	Change in the 90th quantile of the distribution	3	1		-0.16	-0.17	-0.15
		Economic and Social Disadvantage	Cerebrovascular diseases	overall	Change in the 90th quantile of the distribution	4	1		-0.12	-0.13	-0.11
		Discrimination and Segregation	Cerebrovascular diseases	overall	Change in the 90th quantile of the distribution	5	1		0.33	0.32	0.34
		Discrimination and Segregation	Cerebrovascular diseases	overall	Change in the 90th quantile of the distribution	6	1		0.14	0.13	0.15
[124]	Hu, 2021	Economic and Social Disadvantage	Diabetes mellitus	overall	PR (95%CI)	1	1	Q1 (least deprived, reference)	1.00	-	-
						1	2	Q2	1.37	0.95	1.96
						1	3	Q3	1.57	1.10	1.24
						1	4	Q4, most deprived	1.49	1.03	2.14

		Economic and Social Disadvantage	Diabetes mellitus	overall	PR (95%CI)	2	1	Q1 and Q2, least deprived	1.00	-	-
						2	2	Q3	1.48	1.17	1.37
						2	3	Q4, most deprived	1.46	1.14	1.86
[125]	Huang, 2022	Discrimination and Segregation	Hypertensive diseases	overall	linear regression	1	1	racial segregation (score: 0-1)	2.27	-	-
		Economic and Social Disadvantage	Hypertensive diseases	overall	linear regression	2	1	less than high school (%)	-1.03	-	-
		Discrimination and Segregation	Diabetes mellitus	overall	linear regression	3	1	racial segregation (score: 0-1)	-0.86	-	-
		Economic and Social Disadvantage	Diabetes mellitus	overall	linear regression	4	1	less than high school (%)	1.67	-	-
		Discrimination and Segregation	Ischaemic heart diseases	overall	linear regression	5	1	racial segregation (score: 0-1)	1.38	-	-
		Economic and Social Disadvantage	Ischaemic heart diseases	overall	linear regression	6	1	less than high school (%)	0.19	-	-
		Discrimination and Segregation	Cerebrovascular diseases	overall	linear regression	7	1	racial segregation (score: 0-1)	-0.13	-	-
		Economic and Social Disadvantage	Cerebrovascular diseases	overall	linear regression	8	1	less than high school (%)	0.03	-	-
[126]	Hwang, 2020	Social Cohesion and Social Capital	Diseases of the circulatory system	overall	HR (95%CI)	1	1	1st quintile, lowest social trust (reference)	1.00	-	-
						1	2	2nd	0.93	0.91	0.95
						1	3	3rd	0.96	0.94	0.98
						1	4	4th	0.95	0.93	0.97
						1	5	5th (highest)	0.91	0.89	0.93
		Social Cohesion and Social Capital	Ischaemic heart diseases	overall	HR (95%CI)	2	1	1st quintile, lowest social trust (reference)	1.00	-	-
						2	2	2nd	0.93	0.90	0.96
						2	3	3rd	0.94	0.91	0.97
						2	4	4th	0.95	0.92	0.98
						2	5	5th (highest)	0.92	0.89	0.95
		Social Cohesion and Social Capital	Cerebrovascular diseases	overall	HR (95%CI)	3	1	1st quintile, lowest social trust (reference)	1.00	-	-
						3	2	2nd	0.93	0.90	0.95
						3	3	3rd	0.97	0.95	0.99
						3	4	4th	0.95	0.92	0.97
						3	5	5th (highest)	0.90	0.87	0.93
[127]	Jack, 2019	Economic and Social Disadvantage	Cerebrovascular diseases	overall	RR (95%CI)	1	1		1.06	1.06	1.07

		Economic and Social Disadvantage	Ischaemic heart diseases	overall	RR (95%CI)	2	1		1.07	1.06	1.07
[128]	Jackson, 2008	Economic and Social Disadvantage	Diseases of the circulatory system	overall	OR (95%CI)	1	1		0.99	0.81	1.15
		Economic and Social Disadvantage	Diseases of the circulatory system	overall	OR (95%CI)	2	1		1.02	0.87	1.18
		Discrimination and Segregation	Diseases of the circulatory system	overall	OR (95%CI)	3	1		0.99	0.89	1.09
		Economic and Social Disadvantage	Diseases of the circulatory system	overall	OR (95%CI)	4	1		1.01	0.90	1.12
		Economic and Social Disadvantage	Diseases of the circulatory system	overall	OR (95%CI)	5	1		1.06	0.75	1.46
[129]	Jadow, 2023	Economic and Social Disadvantage	Cerebrovascular diseases	overall	OR (95%CI)	1	1	per 1-unit change	1.00	1.00	1.00
		Economic and Social Disadvantage	Cerebrovascular diseases	overall	OR (95%CI)	2	1	per 1% change in exposure	1.01	1.01	1.01
		Economic and Social Disadvantage	Cerebrovascular diseases	overall	OR (95%CI)	3	1	per 1% change in exposure	1.01	1.01	1.01
		Discrimination and Segregation	Cerebrovascular diseases	overall	OR (95%CI)	4	1	per 1% change in exposure	1.00	1.00	1.00
		Social Cohesion and Social Capital	Cerebrovascular diseases	overall	OR (95%CI)	5	1	per 1% change in exposure	1.00	1.00	1.00
		Economic and Social Disadvantage	Cerebrovascular diseases	overall	OR (95%CI)	6	1	per 1-unit change	1.02	1.02	1.05
[130]	Jain, 2022	Economic and Social Disadvantage	Diseases of the circulatory system	overall	OR (95%CI)	1	1	least vulnerable (reference)	1.00	-	-
						1	2		1.09	1.06	1.12
						1	3	most vulnerable	1.15	1.12	1.19
		Economic and Social Disadvantage	Hypertensive diseases	overall	OR (95%CI)	2	1	least vulnerable (reference)	1.00	-	-
						2	2		1.03	1.01	1.06
						2	3	most vulnerable	1.14	1.11	1.17
		Economic and Social Disadvantage	Diabetes mellitus	overall	OR (95%CI)	3	1	least vulnerable (reference)	1.00	-	-
						3	2		1.06	1.03	1.08
						3	3	most vulnerable	1.12	1.09	1.15
[131]	Jonsson, 2020	Economic and Social Disadvantage	Other forms of heart disease	overall	IRR (95%CI)	1	1	low SES (reference)	1.00	-	-
						1	2	med-low SES	0.97	0.89	1.07

						1	3	medium SES	0.82	0.75	0.91
						1	4	med-high SES	0.78	0.70	0.86
						1	5	high SES	0.63	0.56	0.71
[132]	Jung, 2019	Economic and Social Disadvantage	Diabetes mellitus	overall	OR (95%CI)	1	1	1 (poorest, reference)	0.00	-	-
						1	2	2	1.09	1.04	1.14
						1	3	3	1.26	1.20	1.32
						1	4	4	1.45	1.39	1.52
						1	5	5 (richest)	1.84	1.76	1.92
		Economic and Social Disadvantage	Diabetes mellitus	overall	OR (95%CI)	2	1	1 (poorest, reference)	0.00	-	-
						2	2	2	1.05	1.03	1.07
						2	3	3	1.13	1.11	1.16
						2	4	4	1.27	1.24	1.29
						2	5	5 (richest)	1.50	1.47	1.54
		Economic and Social Disadvantage	Hypertensive diseases	overall	OR (95%CI)	3	1	1 (poorest, reference)	0.00	-	-
						3	2	2	1.05	1.03	1.07
						3	3	3	1.12	1.10	1.14
						3	4	4	1.20	1.17	1.22
						3	5	5 (richest)	1.33	1.30	1.35
		Economic and Social Disadvantage	Hypertensive diseases	overall	OR (95%CI)	4	1	1 (poorest, reference)	0.00	-	-
						4	2	2	0.98	0.97	1.00
						4	3	3	1.03	1.02	1.05
						4	4	4	1.08	1.07	1.10
						4	5	5 (richest)	1.21	1.19	1.22
[133]	Kaiser, 2016	Crime and Safety	Hypertensive diseases	overall	HR (95%CI)	1	1		0.98	0.92	1.04
		Social Cohesion and Social Capital	Hypertensive diseases	overall	HR (95%CI)	2	1		0.99	0.94	1.05
[134]	Kakinami, 2017	Economic and Social Disadvantage	Diseases of the circulatory system	male	HR (95%CI)	1	1		1.68	0.98	2.90
		Economic and Social Disadvantage	Diseases of the circulatory system	female	HR (95%CI)	2	1		1.51	0.94	2.41
[135]	Kauhl, 2018	Economic and Social Disadvantage	Hypertensive diseases	overall	Beta (95%CI)	1	1		0.00	not reported	not reported
[136]	Kawachi, 1996	Social Relationships and Norms	Cerebrovascular diseases	male	RR (95%CI)	1	1	IV (high, reference)	1.00	-	-

						1	2	III	1.50	0.87	2.60
						1	3	II	1.61	0.97	2.67
						1	4	I	1.86	0.85	4.06
		Social Relationships and Norms	Ischaemic heart diseases	male	RR (95%CI)	2	1	IV (high, reference)	1.00	-	-
						2	2	III	1.32	0.98	1.79
						2	3	II	1.04	0.76	1.40
						2	4	I	1.00	0.58	1.71
[137]	Keita, 2014	Economic and Social Disadvantage	Hypertensive diseases	overall	OR (95%CI)	1	1	Q1	1.34	1.07	1.68
						1	2	Q2	1.32	1.05	1.66
						1	3	Q3	1.20	0.95	1.51
						1	4	Q4	1.09	0.86	1.38
						1	5	Q5 (reference, least deprived)	1.00	-	-
		Economic and Social Disadvantage	Hypertensive diseases	overall	OR (95%CI)	2	1	Q1	1.13	0.97	1.31
						2	2	Q2	1.20	1.06	1.36
						2	3	Q3	1.14	1.02	1.28
						2	4	Q4	1.14	1.03	1.26
						2	5	Q5 (least deprived, reference)	1.00	-	-
[138]	Kelli, 2017	Economic and Social Disadvantage	Hypertensive diseases	overall	prevalence (t-test)	1	1	low income area	0.42	not reported	not reported
						1	2	high income area	0.31	not reported	not reported
		Economic and Social Disadvantage	Diabetes mellitus	overall	prevalence (t-test)	2	1	low income area	0.11	not reported	not reported
						2	2	high income area	0.08	not reported	not reported
		Economic and Social Disadvantage	Ischaemic heart diseases	overall	prevalence (t-test)	3	1	low income area	0.06	not reported	not reported
						3	2	high income area	0.03	not reported	not reported
[139]	Kershaw, 2011	Discrimination and Segregation	Hypertensive diseases	overall	OR (95%CI)	1	1	(higher index = more isolation)	0.96	0.86	1.07
		Economic and Social Disadvantage	Hypertensive diseases	overall	OR (95%CI)	2	1		1.07	0.97	1.18
[140]	Kim, 2022	Discrimination and Segregation	Ischaemic heart diseases	overall	Risk difference	1	1	% change per 1 SD change	-0.01	-0.03	0.02
		Discrimination and Segregation	Hypertensive diseases	overall	Risk difference	2	1	% change per 1 SD change	-0.06	-0.16	0.04
		Discrimination and Segregation	Diabetes mellitus	overall	Risk difference	3	1	% change per 1 SD change	0.00	-0.03	0.04

[141]	Kim, 2018	Economic and Social Disadvantage	Ischaemic heart diseases	overall	HR (95%CI)	1	1	Q1 (lowest neighborhood socioeconomic position, reference)	1.00	-	-
						1	2	Q2	1.17	0.89	1.52
						1	3	Q3	0.79	0.57	1.09
						1	4	Q4 (highest)	1.05	0.70	1.57
		Economic and Social Disadvantage	Hypertensive diseases	overall	HR (95%CI)	2	1	Q1 (lowest neighborhood socioeconomic position, reference)	1.00	-	-
						2	2	Q2	1.06	0.98	1.14
						2	3	Q3	1.09	0.99	1.19
						2	4	Q4 (highest)	1.12	0.94	1.32
		Economic and Social Disadvantage	Diabetes mellitus	overall	HR (95%CI)	3	1	Q1 (lowest neighborhood socioeconomic position, reference)	1.00	-	-
						3	2	Q2	1.00	0.88	1.14
						3	3	Q3	0.98	0.85	1.13
						3	4	Q4 (highest)	1.01	0.84	1.21
		Economic and Social Disadvantage	Ischaemic heart diseases	overall	HR (95%CI)	4	1	Q1 (lowest neighborhood socioeconomic position, reference)	1.00	-	-
						4	2	Q2	1.09	0.83	1.43
						4	3	Q3	0.76	0.54	1.07
						4	4	Q4 (highest)	0.99	0.66	1.50
		Economic and Social Disadvantage	Hypertensive diseases	overall	HR (95%CI)	5	1	Q1 (lowest neighborhood socioeconomic position, reference)	1.00	-	-
						5	2	Q2	1.05	0.97	1.13
						5	3	Q3	1.08	0.98	1.19
						5	4	Q4 (highest)	1.11	0.93	1.31
		Economic and Social Disadvantage	Diabetes mellitus	overall	HR (95%CI)	6	1	Q1 (lowest neighborhood socioeconomic position, reference)	1.00	-	-
						6	2	Q2	1.00	0.88	1.14
						6	3	Q3	0.98	0.85	1.13
						6	4	Q4 (highest)	1.01	0.84	1.21
[142]	Kim, 2022	Social Cohesion and Social Capital	Diseases of the circulatory system	overall	HR (95%CI)	1	1	neighborhood social cohesion score	0.94	0.85	1.04
		Disorder and Incivilities	Diseases of the circulatory system	overall	HR (95%CI)	2	1	neighborhood physical disorder score	1.08	0.98	1.19
		Crime and Safety	Diseases of the circulatory system	overall	HR (95%CI)	3	1	annual murder rate	0.95	0.88	1.03
[143]	Kivimäki, 2018	Economic and Social Disadvantage	Hypertensive diseases	overall	OR (95%CI)	1	1	low disadvantage (<= -0.5SD)	1.00	-	-
						1	2	low intermediate (-0.5 to 0 SD)	1.19	0.81	1.74

						1	3	high intermediate (>0 to 0.5 SD)	1.39	0.92	2.09
						1	4	high (>= 0.5SD)	1.83	1.14	2.93
		Economic and Social Disadvantage	Diabetes mellitus	overall	OR (95%CI)	2	1	low disadvantage (<= -0.5SD)	1.00	-	-
						2	2	low intermediate (-0.5 to 0 SD)	1.63	0.83	3.22
						2	3	high intermediate (>0 to 0.5 SD)	1.83	0.90	3.70
						2	4	high (>= 0.5SD)	3.71	1.77	7.75
		Economic and Social Disadvantage	Diabetes mellitus	overall	OR (95%CI)	3	1	stable low	1.00	-	-
						3	2	low to high	1.48	0.78	2.84
						3	3	high to low	1.33	0.73	2.42
						3	4	stable high	2.27	1.30	3.97
[144]	Kivimaki, 2021	Economic and Social Disadvantage	Diabetes mellitus	overall	HR (95%CI)	1	1	advantaged	0.83	0.75	0.92
						1	2	disadvantaged (reference)	1.00	-	-
		Economic and Social Disadvantage	Diseases of the circulatory system	overall	HR (95%CI)	2	1	advantaged	0.96	0.89	1.04
						2	2	disadvantaged (reference)	1.00	-	-
		Economic and Social Disadvantage	Hypertensive diseases	overall	HR (95%CI)	3	1	advantaged	0.92	0.83	1.02
						3	2	disadvantaged (reference)	1.00	-	-
		Economic and Social Disadvantage	Ischaemic heart diseases	overall	HR (95%CI)	4	1	advantaged	1.02	0.88	1.18
						4	2	disadvantaged (reference)	1.00	-	-
		Economic and Social Disadvantage	Ischaemic heart diseases	overall	HR (95%CI)	5	1	advantaged	1.04	0.82	1.30
						5	2	disadvantaged (reference)	1.00	-	-
		Economic and Social Disadvantage	Other forms of heart disease	overall	HR (95%CI)	6	1	advantaged	0.95	0.70	1.31
						6	2	disadvantaged (reference)	1.00	-	-
		Economic and Social Disadvantage	Cerebrovascular diseases	overall	HR (95%CI)	7	1	advantaged	1.12	0.93	1.36
						7	2	disadvantaged (reference)	1.00	-	-
		Economic and Social Disadvantage	Cerebrovascular diseases	overall	HR (95%CI)	8	1	advantaged	1.12	0.91	1.38
						8	2	disadvantaged (reference)	1.00	-	-

Economic and Social Disadvantage	Cerebrovascular diseases	overall	HR (95%CI)	9	1	advantaged	0.83	0.48	1.42
				9	2	disadvantaged (reference)	1.00	-	-
Economic and Social Disadvantage	Diabetes mellitus	overall	HR (95%CI)	10	1	advantaged	0.75	0.67	0.85
				10	2	disadvantaged (reference)	1.00	-	-
Economic and Social Disadvantage	Diseases of the circulatory system	overall	HR (95%CI)	11	1	advantaged	1.09	1.00	1.19
				11	2	disadvantaged (reference)	1.00	-	-
Economic and Social Disadvantage	Hypertensive diseases	overall	HR (95%CI)	12	1	advantaged	0.79	0.70	0.90
				12	2	disadvantaged (reference)	1.00	-	-
Economic and Social Disadvantage	Ischaemic heart diseases	overall	HR (95%CI)	13	1	advantaged	0.99	0.83	1.18
				13	2	disadvantaged (reference)	1.00	-	-
Economic and Social Disadvantage	Ischaemic heart diseases	overall	HR (95%CI)	14	1	advantaged	1.06	0.80	1.42
				14	2	disadvantaged (reference)	1.00	-	-
Economic and Social Disadvantage	Other forms of heart disease	overall	HR (95%CI)	15	1	advantaged	0.84	0.57	1.23
				15	2	disadvantaged (reference)	1.00	-	-
Economic and Social Disadvantage	Cerebrovascular diseases	overall	HR (95%CI)	16	1	advantaged	0.93	0.73	1.17
				16	2	disadvantaged (reference)	1.00	-	-
Economic and Social Disadvantage	Cerebrovascular diseases	overall	HR (95%CI)	17	1	advantaged	0.85	0.65	1.11
				17	2	disadvantaged (reference)	1.00	-	-
Economic and Social Disadvantage	Cerebrovascular diseases	overall	HR (95%CI)	18	1	advantaged	0.85	0.46	1.57
				18	2	disadvantaged (reference)	1.00	-	-
Economic and Social Disadvantage	Diabetes mellitus	overall	HR (95%CI)	19	1	advantaged	0.86	0.79	0.94
				19	2	disadvantaged (reference)	1.00	-	-
Economic and Social Disadvantage	Diseases of the circulatory system	overall	HR (95%CI)	20	1	advantaged	0.97	0.90	1.04
				20	2	disadvantaged (reference)	1.00	-	-
Economic and Social Disadvantage	Hypertensive diseases	overall	HR (95%CI)	21	1	advantaged	0.98	0.89	1.07

						21	2	disadvantaged (reference)	1.00	-	-	
		Economic and Social Disadvantage	Ischaemic heart diseases	overall	HR (95%CI)	22	1	advantaged	0.95	0.84	1.08	
						22	2	disadvantaged (reference)	1.00	-	-	
		Economic and Social Disadvantage	Ischaemic heart diseases	overall	HR (95%CI)	23	1	advantaged	1.02	0.83	1.25	
						23	2	disadvantaged (reference)	1.00	-	-	
		Economic and Social Disadvantage	Other forms of heart disease	overall	HR (95%CI)	24	1	advantaged	0.74	0.55	1.00	
						24	2	disadvantaged (reference)	1.00	-	-	
		Economic and Social Disadvantage	Cerebrovascular diseases	overall	HR (95%CI)	25	1	advantaged	0.86	0.72	1.04	
						25	2	disadvantaged (reference)	1.00	-	-	
		Economic and Social Disadvantage	Cerebrovascular diseases	overall	HR (95%CI)	26	1	advantaged	0.80	0.66	0.98	
						26	2	disadvantaged (reference)	1.00	-	-	
		Economic and Social Disadvantage	Cerebrovascular diseases	overall	HR (95%CI)	27	1	advantaged	0.77	0.48	1.23	
						27	2	disadvantaged (reference)	1.00	-	-	
[145]	Kling, 2007	Economic and Social Disadvantage	Hypertensive diseases	overall	RR (SE)	1	1	Control group (reference)	1.00	-	-	
						1	2	Experimental	0.02	not reported	not reported	0.02
		Economic and Social Disadvantage	Hypertensive diseases	overall	RR (SE)	2	1	Control group (reference)	1.00	-	-	
						2	2	Tradicional voucher	0.02	not reported	not reported	0.02
[146]	Jensen, 2023	Economic and Social Disadvantage	Hypertensive diseases	overall	HR (95%CI)	1	1	per 1 SD change	1.02	0.97	1.07	
		Economic and Social Disadvantage	Hypertensive diseases	overall	HR (95%CI)	2	1	per 1 SD change	1.02	1.00	1.07	
		Economic and Social Disadvantage	Hypertensive diseases	overall	HR (95%CI)	3	1	per 1 SD change	0.99	0.94	1.05	
		Economic and Social Disadvantage	Hypertensive diseases	overall	HR (95%CI)	4	1	per 1 SD change	1.00	0.96	1.04	
		Economic and Social Disadvantage	Diabetes mellitus	overall	HR (95%CI)	5	1	per 1 SD change	1.03	0.96	1.10	
		Economic and Social Disadvantage	Diabetes mellitus	overall	HR (95%CI)	6	1	per 1 SD change	1.01	0.95	1.07	

		Economic and Social Disadvantage	Diabetes mellitus	overall	HR (95%CI)	7	1	per 1 SD change	0.97	0.91	1.05	
		Economic and Social Disadvantage	Diabetes mellitus	overall	HR (95%CI)	8	1	per 1 SD change	1.01	0.95	1.07	
		Economic and Social Disadvantage	Ischaemic heart diseases	overall	HR (95%CI)	9	1	per 1 SD change	0.95	0.83	1.09	
		Economic and Social Disadvantage	Ischaemic heart diseases	overall	HR (95%CI)	10	1	per 1 SD change	1.06	0.94	1.18	
		Economic and Social Disadvantage	Ischaemic heart diseases	overall	HR (95%CI)	11	1	per 1 SD change	0.99	0.86	1.15	
		Economic and Social Disadvantage	Ischaemic heart diseases	overall	HR (95%CI)	12	1	per 1 SD change	1.08	0.97	1.20	
		Economic and Social Disadvantage	Ischaemic heart diseases	overall	HR (95%CI)	9	1	per 1 SD change	1.05	0.97	1.13	
		Economic and Social Disadvantage	Ischaemic heart diseases	overall	HR (95%CI)	10	1	per 1 SD change	1.01	0.94	1.08	
		Economic and Social Disadvantage	Ischaemic heart diseases	overall	HR (95%CI)	11	1	per 1 SD change	0.97	0.89	1.05	
		Economic and Social Disadvantage	Ischaemic heart diseases	overall	HR (95%CI)	12	1	per 1 SD change	1.05	0.99	1.12	
[147]	Kolpak, 2017	Economic and Social Disadvantage	Diabetes mellitus	overall	Beta (95%CI)	1	1		0.08	not reported	not reported	0.02
		Economic and Social Disadvantage	Diabetes mellitus	overall	Beta (95%CI)	2	1		0.34	not reported	not reported	0.03
		Economic and Social Disadvantage	Diabetes mellitus	overall	Beta (95%CI)	3	1		0.15	not reported	not reported	0.09
[148]	Krieger, 1992	Economic and Social Disadvantage	Hypertensive diseases	overall	OR (95%CI)	1	1	(>=66% working class vs. <66%)	1.00	-	-	
						1	2		1.00	0.90	1.20	
		Economic and Social Disadvantage	Hypertensive diseases	overall	OR (95%CI)	2	1	(>= 25% high school degree vs. < 25%)	1.00	-	-	
						2	2		1.20	1.00	1.40	
		Economic and Social Disadvantage	Hypertensive diseases	overall	OR (95%CI)	3	1	(>=66% working class vs. <66%)	1.00	-	-	
						3	2		1.10	0.90	1.30	
		Economic and Social Disadvantage	Hypertensive diseases	overall	OR (95%CI)	4	1	(>= 25% high school degree vs. < 25%)	1.00	-	-	
						4	2		1.20	1.00	1.40	

[149]	Krishnan, 2010	Economic and Social Disadvantage	Diabetes mellitus	male	IRR (95%CI)	1	1	Q1 (lowest neighborhood socioeconomic status)	1.65	1.46	1.85
						1	2	Q2	1.40	1.25	1.58
						1	3	Q3	1.38	1.23	1.55
						1	4	Q4	1.22	1.08	1.36
						1	5	Q5 (highest neighborhood socioeconomic status, reference)	1.00	-	-
[150]	Kwok, 2021	Economic and Social Disadvantage	Diseases of the circulatory system	overall	OR (95%CI)	1	1	Quartile 1 (lowest income, reference)	1.00	-	-
						1	2	Quartile 2	0.96	0.77	1.20
						1	3	Quartile 3	0.81	0.64	1.02
						1	4	Quartile 4 (highest income)	0.89	0.69	1.15
		Economic and Social Disadvantage	Diabetes mellitus	overall	OR (95%CI)	2	1	Quartile 1 (lowest income, reference)	1.00	-	-
						2	2	Quartile 2	0.96	0.78	1.18
						2	3	Quartile 3	0.97	0.79	1.20
						2	4	Quartile 4 (highest income)	0.95	0.75	1.20
		Economic and Social Disadvantage	Diseases of the circulatory system	overall	OR (95%CI)	3	1	Quartile 1 (more equality, reference)	1.00	-	-
						3	2	Quartile 2	0.94	0.72	1.21
						3	3	Quartile 3	0.94	0.73	1.20
						3	4	Quartile 4 (more inequality)	1.24	0.98	1.57
		Economic and Social Disadvantage	Diabetes mellitus	overall	OR (95%CI)	4	1	Quartile 1 (more equality, reference)	1.00	-	-
						4	2	Quartile 2	0.88	0.71	1.11
						4	3	Quartile 3	0.98	0.78	1.23
						4	4	Quartile 4 (more inequality)	0.84	0.68	1.04
[151]	Lachkhem, 2018	Economic and Social Disadvantage	Cerebrovascular diseases	male	p-value	1	1	Persistent High stroke rate (reference)	0.31	not reported	not reported
		Economic and Social Disadvantage	Cerebrovascular diseases	overall	p-value	2	1	Low stroke rate	0.11	not reported	not reported
		Economic and Social Disadvantage	Cerebrovascular diseases	male	p-value	3	1	Into high stroke rate	0.30	not reported	not reported
		Economic and Social Disadvantage	Cerebrovascular diseases	male	p-value	4	1	Out of High stroke rate	0.27	not reported	not reported
		Economic and Social Disadvantage	Cerebrovascular diseases	overall	p-value	5	1	Into low stroke rate	0.14	not reported	not reported

[152]	Lagisetty, 2016	Social Cohesion and Social Capital	Hypertensive diseases	male	OR (95%CI)	1	1	lowest social cohesion (reference)	1.00	-	-
						1	2	medium tertile	0.93	0.66	1.29
						1	3	highest tertile	0.71	0.46	1.10
		Social Cohesion and Social Capital	Diabetes mellitus	male	OR (95%CI)	2	1	lowest social cohesion (reference)	1.00	-	-
						2	2	medium tertile	1.02	0.70	1.49
						2	3	highest tertile	0.67	0.39	1.13
[153]	Larrañaga, 2005	Economic and Social Disadvantage	Diabetes mellitus	male	OR (95%CI)	1	1	I (least deprived)	1.00	-	-
						1	2	II	1.38	1.15	1.66
						1	3	III	1.44	1.20	1.75
						1	4	IV	1.77	1.47	2.14
						1	5	V (most deprived)	1.69	1.41	2.03
		Economic and Social Disadvantage	Diabetes mellitus	female	OR (95%CI)	2	1	I (least deprived)	1.00	-	-
						2	2	II	1.27	1.05	1.54
						2	3	III	1.91	1.60	2.31
						2	4	IV	2.22	1.85	2.68
						2	5	V (most deprived)	2.28	1.91	2.73
[154]	Laursen, 2017	Social Relationships and Norms	Diabetes mellitus	overall	HR (95%CI)	1	1		0.99	0.98	1.00
		Social Relationships and Norms	Diabetes mellitus	overall	HR (95%CI)	2	1	< 3 ties (limited social network)	1.14	0.94	1.39
						2	2	>= 3 ties (reference)	1.00	-	-
[155]	Lawlor, 2005	Economic and Social Disadvantage	Ischaemic heart diseases	female	OR (95%CI)	1	1	Living in affluent ward (below or equal to Britain median for Carstairs score, reference)	1.00	-	-
						1	2	Living in deprived ward (above Britain median for Carstairs score)	1.27	1.02	1.57
[156]	Lee, 2018	Economic and Social Disadvantage	Diabetes mellitus	overall	Relative change in the prevalence	1	1		-0.17	not reported	not reported
		Economic and Social Disadvantage	Diabetes mellitus	male	Relative change in the prevalence	2	1		-0.09	not reported	not reported
		Economic and Social Disadvantage	Diabetes mellitus	male	Relative change in the prevalence	3	1		-0.02	not reported	not reported
		Discrimination and Segregation	Diabetes mellitus	male	Relative change in the prevalence	4	1		0.03	not reported	not reported

		Discrimination and Segregation	Diabetes mellitus	male	Relative change in the prevalence	5	1		-0.02	not reported	not reported
[157]	Lee, 2018	Economic and Social Disadvantage	Cerebrovascular diseases	male	Beta (95%CI)	1	1		33.23	13.81	52.64
		Economic and Social Disadvantage	Cerebrovascular diseases	female	Beta (95%CI)	2	1		-2.35	21.99	17.28
		Economic and Social Disadvantage	Cerebrovascular diseases	overall	Beta (95%CI)	2	1		17.00	-2.36	36.37
[158]	Lemstra, 2006	Economic and Social Disadvantage	Diabetes mellitus	overall	RR (95%CI)	1	1		3.98	2.72	5.82
						1	2		12.86	5.42	30.51
						1	3		1.34	1.07	1.68
						1	4		1.70	1.14	2.53
						1	5		1.33	0.91	1.93
						1	6		1.82	0.89	3.72
[159]	Leyland, 2005	Economic and Social Disadvantage	Diseases of the circulatory system	overall	OR (95%CI)	1	1		1.04	1.02	1.06
[160]	Li, 2017	Discrimination and Segregation	Hypertensive diseases	overall	OR (95%CI)	1	1	percentage latino >=25% (1 = high Latino concentration; 0 = low, reference)	1.34	0.91	1.97
		Discrimination and Segregation	Hypertensive diseases	overall	OR (95%CI)	2	1	immigrant concentration (low, reference)	1.00	-	-
						2	2	immigrant concentration (medium)	0.73	0.49	1.09
						2	3	immigrant concentration (high)	0.63	0.43	0.92
[161]	Linde, 2023	Economic and Social Disadvantage	Diabetes mellitus	overall	elasticities (/ %change)	1	1	% change	-0.64	-0.67	-0.60
		Social Cohesion and Social Capital	Diabetes mellitus	overall	elasticities (/ %change)	2	1	% change	0.21	0.16	0.26
		Civic Participation and Engagement	Diabetes mellitus	overall	elasticities (/ %change)	3	1	% change	0.01	0.00	0.02
		Economic and Social Disadvantage	Hypertensive diseases	overall	elasticities (/ %change)	4	1	% change	-0.31	-0.33	-0.29
		Social Relationships and Norms	Hypertensive diseases	overall	elasticities (/ %change)	5	1	% change	0.16	0.13	0.19
		Civic Participation and Engagement	Hypertensive diseases	overall	elasticities (/ %change)	6	1	% change	0.02	0.01	0.02
[162]	Ling, 2009	Economic and Social Disadvantage	Hypertensive diseases	overall	OR (95%CI)	1	1		0.65	not reported	not reported

		Economic and Social Disadvantage	Hypertensive diseases	overall	OR (95%CI)	2	1		1.00	not reported	not reported
		Economic and Social Disadvantage	Hypertensive diseases	overall	OR (95%CI)	3	1		0.99	not reported	not reported
[163]	Lippert, 2017	Economic and Social Disadvantage	Diabetes mellitus	overall	OR (95%CI)	1	1	Consistently lived in poor neighborhoods vs. Never lived in poor neighborhood	1.28	0.93	1.76
		Economic and Social Disadvantage	Diabetes mellitus	overall	OR (95%CI)	2	1	Entered neighborhood poverty vs. Never lived in poor neighborhood	1.40	1.03	1.90
		Economic and Social Disadvantage	Diabetes mellitus	overall	OR (95%CI)	3	1	Entered neighborhood poverty vs. Consistently lived in poor neighborhoods	1.09	0.76	1.57
		Economic and Social Disadvantage	Diabetes mellitus	overall	OR (95%CI)	4	1	Exited neighborhood poverty vs. Never lived in poor neighborhood	1.12	0.82	1.53
		Economic and Social Disadvantage	Diabetes mellitus	overall	OR (95%CI)	5	1	Exited neighborhood poverty vs. Consistently lived in poor neighborhoods	0.88	0.62	1.25
		Economic and Social Disadvantage	Diabetes mellitus	overall	OR (95%CI)	6	1	Exited neighborhood poverty vs. Entered neighborhood poverty	0.80	0.56	1.16
[164]	Lönn, 2019	Economic and Social Disadvantage	Ischaemic heart diseases	male	OR (95%CI)	1	1	Middle	1.12	0.99	1.26
						1	2	High	1.36	1.17	1.58
						1	3	Low (least deprived, reference)	1.00	-	-
		Economic and Social Disadvantage	Ischaemic heart diseases	male	OR (95%CI)	2	1	Middle	1.18	1.09	1.28
						2	2	High	1.42	1.29	1.57
						2	3	Low (least deprived, reference)	1.00	-	-
		Economic and Social Disadvantage	Ischaemic heart diseases	male	OR (95%CI)	3	1	Middle	1.03	0.97	1.10
						3	2	High	1.24	1.14	1.35
						3	3	Low (least deprived, reference)	1.00	-	-
		Economic and Social Disadvantage	Ischaemic heart diseases	male	OR (95%CI)	4	1	Middle	1.08	1.03	1.13
						4	2	High	1.22	1.14	1.30
						4	3	Low (least deprived, reference)	1.00	-	-
		Economic and Social Disadvantage	Ischaemic heart diseases	male	OR (95%CI)	5	1	Middle	1.07	1.01	1.13
						5	2	High	1.20	1.12	1.29
						5	3	Low (least deprived, reference)	1.00	-	-
		Economic and Social Disadvantage	Ischaemic heart diseases	male	OR (95%CI)	6	1	Middle	1.11	1.05	1.17

				6	2	High	1.24	1.16	1.32
				6	3	Low (least deprived, reference)	1.00	-	-
Economic and Social Disadvantage	Ischaemic heart diseases	male	OR (95%CI)	7	1	Middle	1.11	1.05	1.18
				7	2	High	1.20	1.12	1.29
				7	3	Low (least deprived, reference)	1.00	-	-
Economic and Social Disadvantage	Ischaemic heart diseases	male	OR (95%CI)	8	1	Middle	1.07	1.01	1.13
				8	2	High	1.12	1.05	1.20
				8	3	Low (least deprived, reference)	1.00	-	-
Economic and Social Disadvantage	Ischaemic heart diseases	female	OR (95%CI)	9	1	Middle	1.18	0.99	1.40
				9	2	High	1.56	1.26	1.92
				9	3	Low (least deprived, reference)	1.00	-	-
Economic and Social Disadvantage	Ischaemic heart diseases	female	OR (95%CI)	10	1	Middle	1.14	1.01	1.27
				10	2	High	1.48	1.29	1.71
				10	3	Low (least deprived, reference)	1.00	-	-
Economic and Social Disadvantage	Ischaemic heart diseases	female	OR (95%CI)	11	1	Middle	1.28	1.15	1.42
				11	2	High	1.52	1.34	1.74
				11	3	Low (least deprived, reference)	1.00	-	-
Economic and Social Disadvantage	Ischaemic heart diseases	female	OR (95%CI)	12	1	Middle	1.16	1.08	1.24
				12	2	High	1.42	1.30	1.55
				12	3	Low (least deprived, reference)	1.00	-	-
Economic and Social Disadvantage	Ischaemic heart diseases	female	OR (95%CI)	13	1	Middle	1.22	1.13	1.32
				13	2	High	1.43	1.30	1.57
				13	3	Low (least deprived, reference)	1.00	-	-
Economic and Social Disadvantage	Ischaemic heart diseases	female	OR (95%CI)	14	1	Middle	1.10	1.04	1.17
				14	2	High	1.24	1.15	1.34
				14	3	Low (least deprived, reference)	1.00	-	-
Economic and Social Disadvantage	Ischaemic heart diseases	female	OR (95%CI)	15	1	Middle	1.11	1.05	1.19
				15	2	High	1.22	1.13	1.32
				15	3	Low (least deprived, reference)	1.00	-	-

		Economic and Social Disadvantage	Ischaemic heart diseases	female	OR (95%CI)	16	1	Middle	1.15	1.09	1.21
						16	2	High	1.20	1.12	1.28
						16	3	Low (least deprived, reference)	1.00	-	-
[165]	Loucks, 2006	Social Relationships and Norms	Hypertensive diseases	female	p trend	1	1	1 (most isolated, low index)	45.00	not reported	not reported
						1	2	2	45.00	not reported	not reported
						1	3	3	41.00	not reported	not reported
						1	4	4 (high)	39.00	not reported	not reported
		Social Relationships and Norms	Hypertensive diseases	male	p trend	2	1	1 (most isolated, low index)	52.00	not reported	not reported
						2	2	2	49.00	not reported	not reported
						2	3	3	49.00	not reported	not reported
						2	4	4 (high)	47.00	not reported	not reported
		Social Relationships and Norms	Diseases of the circulatory system	female	p trend	3	1	1 (most isolated, low index)	13.00	not reported	not reported
						3	2	2	10.00	not reported	not reported
						3	3	3	9.00	not reported	not reported
						3	4	4 (high)	6.00	not reported	not reported
		Social Relationships and Norms	Diseases of the circulatory system	male	p trend	4	1	1 (most isolated, low index)	21.00	not reported	not reported
						4	2	2	20.00	not reported	not reported
						4	3	3	17.00	not reported	not reported
						4	4	4 (high)	12.00	not reported	not reported
[166]	Lu, 2019	Social Relationships and Norms	Hypertensive diseases	male	OR (95%CI)	1	1	Socially isolated (reference)	1.00	-	-
						1	2	Moderately isolated	0.22	0.07	0.71
						1	3	Moderately integrated	0.23	0.07	0.76
						1	4	Socially integrated	0.25	0.08	0.82
		Social Relationships and Norms	Hypertensive diseases	female	OR (95%CI)	2	1	Socially isolated (reference)	1.00	-	-
						2	2	Moderately isolated	1.22	0.35	4.29
						2	3	Moderately integrated	0.79	0.24	2.66
						2	4	Socially integrated	1.03	0.31	3.36

		Social Relationships and Norms	Hypertensive diseases	male	OR (95%CI)	3	1	Low	1.00	-	-
						3	2	High	0.50	0.26	0.93
		Social Relationships and Norms	Hypertensive diseases	female	OR (95%CI)	4	1	Low	1.00	-	-
						4	2	High	0.48	0.24	0.94
[167]	Ludwig, 2011	Economic and Social Disadvantage	Diabetes mellitus	female	OR (95%CI)	1	1	Control (reference)	1.00	-	-
						1	2	Low-poverty voucher	-4.31	-7.82	-0.80
						1	3	Tradicional voucher	-0.08	-5.18	5.02
[168]	Lukachko, 2014	Discrimination and Segregation	Ischaemic heart diseases	overall	OR (95%CI)	1	1	high versus low exposure	1.08	0.92	1.27
		Discrimination and Segregation	Ischaemic heart diseases	overall	OR (95%CI)	2	1		0.97	0.81	1.15
		Discrimination and Segregation	Ischaemic heart diseases	overall	OR (95%CI)	3	1		1.35	1.09	1.69
		Discrimination and Segregation	Ischaemic heart diseases	overall	OR (95%CI)	4	1		1.22	1.04	1.44
		Discrimination and Segregation	Ischaemic heart diseases	overall	OR (95%CI)	5	1		1.74	1.48	2.04
		Discrimination and Segregation	Ischaemic heart diseases	overall	OR (95%CI)	6	1		0.76	0.65	0.89
		Discrimination and Segregation	Ischaemic heart diseases	overall	OR (95%CI)	7	1		0.55	0.46	0.65
		Discrimination and Segregation	Ischaemic heart diseases	overall	OR (95%CI)	8	1		1.12	0.91	1.38
		Discrimination and Segregation	Ischaemic heart diseases	overall	OR (95%CI)	9	1		1.01	0.87	1.18
		Discrimination and Segregation	Ischaemic heart diseases	overall	OR (95%CI)	10	1		0.85	0.74	0.98
		Discrimination and Segregation	Ischaemic heart diseases	overall	OR (95%CI)	11	1		0.80	0.70	0.91
		Discrimination and Segregation	Ischaemic heart diseases	overall	OR (95%CI)	12	1		0.94	0.82	1.07
		Discrimination and Segregation	Ischaemic heart diseases	overall	OR (95%CI)	13	1		0.90	0.78	1.04
		Discrimination and Segregation	Ischaemic heart diseases	overall	OR (95%CI)	14	1		1.07	0.94	1.21
		Discrimination and Segregation	Ischaemic heart diseases	overall	OR (95%CI)	15	1		1.03	0.89	1.19
		Discrimination and Segregation	Ischaemic heart diseases	overall	OR (95%CI)	16	1		1.10	0.97	1.24
[169]	Lukaschek, 2017	Social Relationships and Norms	Diabetes mellitus	male	HR (95%CI)	1	1	high (reference)	1.00	-	-
						1	2	moderate	0.99	0.79	1.23
						1	3	low	1.93	1.21	3.08
		Social Relationships and Norms	Diabetes mellitus	female	HR (95%CI)	2	1	high (reference)	1.00	-	-

						2	2	moderate	1.00	0.76	1.31	
						2	3	low	1.49	0.76	2.95	
[170]	Lund, 2012	Social Relationships and Norms	Diseases of the circulatory system	overall	OR (95%CI)	1	1	highest	1.79	1.28	2.51	
						1	2	middle	1.29	0.95	1.74	
						1	3	lowest (reference)	1.00	-	-	
		Social Relationships and Norms	Diseases of the circulatory system	overall	OR (95%CI)	2	1	highest	0.87	0.62	1.22	
						2	2	middle	0.88	0.66	1.17	
						2	3	lowest (reference)	1.00	-	-	
[171]	Lund, 2014	Social Relationships and Norms	Ischaemic heart diseases	overall	HR (95%CI)	1	1	always/often/sometimes	1.43	0.98	2.09	
						1	2	never	0.93	0.71	1.23	
						1	3	seldom (reference)	1.00	-	-	
		Social Relationships and Norms	Ischaemic heart diseases	overall	HR (95%CI)	2	1	always/often/sometimes	1.42	0.86	2.35	
						2	2	never	1.02	0.78	1.33	
						2	3	seldom (reference)	1.00	-	-	
[172]	Ma, 2021	Civic Participation and Engagement	Cerebrovascular diseases	overall	linear regression	1	1		-0.20	-0.38	-0.02	
		Civic Participation and Engagement	Diabetes mellitus	overall	linear regression	2	1		-0.06	-0.20	0.07	
		Civic Participation and Engagement	Hypertensive diseases	overall	linear regression	3	1		-0.01	-0.09	0.07	
		Civic Participation and Engagement	Diseases of the circulatory system	overall	linear regression	4	1		0.00	-0.11	0.10	
[173]	Madela, 2023	Economic and Social Disadvantage	Hypertensive diseases	overall	OR (95%CI)	1	1	Quantile 2: least deprived (reference)	1.00	-	-	
						1	2	Quantile 3	1.86	-	-	0.39
						1	3	Quantile 4: more deprived	2.25	-	-	0.44
[174]	Madela, 2023	Economic and Social Disadvantage	Diabetes mellitus	overall	PR (95%CI)	1	1	Quantile 2 & 3: least deprived (reference)	1.00	-	-	
						1	2	Quantile 4: more deprived	0.84	-	-	0.05
[175]	Maheswaran, 2018	Economic and Social Disadvantage	Ischaemic heart diseases	overall	prevalence OR (95%CI)	1	1		0.72	0.40	1.27	
		Economic and Social Disadvantage	Cerebrovascular diseases	overall	prevalence OR (95%CI)	2	1		1.25	0.76	2.06	

		Economic and Social Disadvantage	Diabetes mellitus	overall	prevalence OR (95%CI)	3	1		0.88	0.49	1.57
		Economic and Social Disadvantage	Ischaemic heart diseases	overall	prevalence OR (95%CI)	4	1		1.56	0.84	2.88
		Economic and Social Disadvantage	Cerebrovascular diseases	overall	prevalence OR (95%CI)	5	1		1.88	0.98	3.61
		Economic and Social Disadvantage	Diabetes mellitus	overall	prevalence OR (95%CI)	6	1		3.03	1.70	5.41
[176]	Maier, 2014	Economic and Social Disadvantage	Diabetes mellitus	male	OR (95%CI)	1	1	Q1, least deprived (reference)	1.00	-	-
						1	2	Q2	0.93	0.77	1.14
						1	3	Q3	1.13	0.93	1.38
						1	4	Q4	1.10	0.90	1.36
						1	5	Q5, most deprived	1.12	0.92	1.36
		Economic and Social Disadvantage	Diabetes mellitus	female	OR (95%CI)	2	1	Q1, least deprived (reference)	1.00	-	-
						2	2	Q2	1.09	0.89	1.33
						2	3	Q3	1.10	0.90	1.35
						2	4	Q4	1.17	0.96	1.43
						2	5	Q5, most deprived	1.39	1.14	1.69
[177]	Malino, 2014	Social Cohesion and Social Capital	Hypertensive diseases	female	OR (95%CI)	1	1		0.59	0.46	0.77
		Social Cohesion and Social Capital	Hypertensive diseases	female	OR (95%CI)	2	1		0.75	0.59	0.95
		Social Cohesion and Social Capital	Hypertensive diseases	female	OR (95%CI)	3	1		0.78	0.65	0.92
		Social Cohesion and Social Capital	Hypertensive diseases	female	OR (95%CI)	4	1		0.38	0.15	0.97
		Social Cohesion and Social Capital	Hypertensive diseases	female	OR (95%CI)	5	1		0.41	0.19	0.88
[178]	Marley, 2015	Social Cohesion and Social Capital	Diabetes mellitus	overall	OR (95%CI)	1	1		1.02	not reported	not reported
		Economic and Social Disadvantage	Diabetes mellitus	overall	OR (95%CI)	2	1		1.04	not reported	not reported
[179]	Massa, 2016	Economic and Social Disadvantage	Diseases of the circulatory system	overall	OR (95%CI)	1	1	Q1 (reference highest income inequality)	1.00	-	-
						1	2	Q2	1.14	0.97	1.33
						1	3	Q3	0.76	0.63	0.92

						1	4	Q4	0.49	0.39	0.63	
		Economic and Social Disadvantage	Diseases of the circulatory system	overall	OR (95%CI)	2	1	Q1 (reference)	1.00	-	-	
						2	2	Q2	1.35	1.15	1.59	
						2	3	Q3	2.71	2.18	3.36	
						2	4	Q4	1.43	1.14	1.79	
[180]	Matheson, 2010	Economic and Social Disadvantage	Hypertensive diseases	male	OR (95%CI)	1	1		0.99	0.96	1.02	
[181]	Matthew, 2018	Economic and Social Disadvantage	Diabetes mellitus	overall	Probit regression coefficient (SE)	1	1		0.01	not reported	not reported	0.01
		Economic and Social Disadvantage	Other forms of heart disease	overall	Probit regression coefficient (SE)	2	1		-0.01	not reported	not reported	0.00
		Economic and Social Disadvantage	Ischaemic heart diseases	overall	Probit regression coefficient (SE)	3	1		-0.01	not reported	not reported	0.01
		Economic and Social Disadvantage	Cerebrovascular diseases	overall	Probit regression coefficient (SE)	4	1		0.00	not reported	not reported	0.00
[182]	Mayne, 2020	Discrimination and Segregation	Diabetes mellitus	overall	HR (95%CI)	1	1	high segregation (reference) (e.g. higher positive values = higher than expected percentage of black residents)	1.00	-	-	
						1	2	medium	0.79	0.61	1.02	
						1	3	low	0.98	0.77	1.24	
[183]	McDoom, 2018	Economic and Social Disadvantage	Hypertensive diseases	overall	HR (95%CI)	1	1	highest tertile	0.79	0.70	0.90	
						1	2	middle	na	not reported	not reported	
						1	3	lowest neighborhood socioeconomic status tertile (reference)	1.00	-	-	
[184]	Menec, 2010	Economic and Social Disadvantage	Diabetes mellitus	overall	OR (95%CI)	1	1	Q1 (poorest)	1.47	not reported	not reported	
						1	2	Q2	1.23	not reported	not reported	
						1	3	Q3	1.18	not reported	not reported	
						1	4	Q4	1.08	not reported	not reported	
						1	5	Q5 (richest, reference)	1.00	-	-	
		Economic and Social Disadvantage	Hypertensive diseases	overall	OR (95%CI)	2	1	Q1 (poorest)	1.19	not reported	not reported	
						2	2	Q2	1.16	not reported	not reported	
						2	3	Q3	1.10	not reported	not reported	

						2	4	Q4	1.07	not reported	not reported
						2	5	Q5 (richest, reference)	1.00	-	-
		Economic and Social Disadvantage	Other forms of heart disease	male	OR (95%CI)	3	1	Q1 (poorest)	1.53	not reported	not reported
						3	2	Q2	1.31	not reported	not reported
						3	3	Q3	1.10	not reported	not reported
						3	4	Q4	1.09	not reported	not reported
						3	5	Q5 (richest, reference)	1.00	-	-
		Economic and Social Disadvantage	Ischaemic heart diseases	overall	OR (95%CI)	4	1	Q1 (poorest)	1.37	not reported	not reported
						4	2	Q2	1.20	not reported	not reported
						4	3	Q3	1.15	not reported	not reported
						4	4	Q4	1.09	not reported	not reported
						4	5	Q5 (richest, reference)	1.00	-	-
		Economic and Social Disadvantage	Ischaemic heart diseases	overall	OR (95%CI)	5	1	Q1 (poorest)	1.33	not reported	not reported
						5	2	Q2	0.98	not reported	not reported
						5	3	Q3	1.06	not reported	not reported
						5	4	Q4	0.95	not reported	not reported
						5	5	Q5 (richest, reference)	1.00	-	-
		Economic and Social Disadvantage	Cerebrovascular diseases	overall	OR (95%CI)	6	1	Q1 (poorest)	1.27	not reported	not reported
						6	2	Q2	1.15	not reported	not reported
						6	3	Q3	1.17	not reported	not reported
						6	4	Q4	1.05	not reported	not reported
						6	5	Q5 (richest, reference)	1.00	-	-
[185]	Mentias, 2023	Economic and Social Disadvantage	Other forms of heart disease	overall	RR (95%CI)	1	1	Quartile 1 to 3 (lowest proportion of redlines area, reference)	1.00	-	-
						1	2	Quartile 4 (highest proportion redlined area)	1.04	1.00	1.08
		Economic and Social Disadvantage	Other forms of heart disease	overall	RR (95%CI)	2	1	Quartile 1 to 3 (lowest proportion of redlines area, reference)	1.00	-	-
						2	2	Quartile 4 (highest proportion redlined area)	0.90	0.85	0.95

Economic and Social Disadvantage	Diabetes mellitus	overall	disease prevalence (%)	3	1	% of disease Q1-Q3 (lowest proportion redlined area)	8.80	7.20	10.60
				3	2	% of disease Q4 (highest proportion redlined area)	9.10	8.00	10.50
Economic and Social Disadvantage	Diabetes mellitus	overall	disease prevalence (%)	4	1	% of disease Q1-Q3 (lowest proportion redlined area)	5.30	3.90	6.80
				4	2	% of disease Q4 (highest proportion redlined area)	6.40	4.40	8.70
Economic and Social Disadvantage	Hypertensive diseases	overall	disease prevalence (%)	5	1	% of disease Q1-Q3 (lowest proportion redlined area)	18.80	16.30	22.00
				5	2	% of disease Q4 (highest proportion redlined area)	19.30	17.20	21.90
Economic and Social Disadvantage	Hypertensive diseases	overall	disease prevalence (%)	6	1	% of disease Q1-Q3 (lowest proportion redlined area)	15.60	13.60	17.90
				6	2	% of disease Q4 (highest proportion redlined area)	16.2	13.40	20.0
Economic and Social Disadvantage	Ischaemic heart diseases	overall	disease prevalence (%)	7	1	% of disease Q1-Q3 (lowest proportion redlined area)	4.00	3.00	5.20
				7	2	% of disease Q4 (highest proportion redlined area)	4.70	3.10	5.00
Economic and Social Disadvantage	Ischaemic heart diseases	overall	disease prevalence (%)	8	1	% of disease Q1-Q3 (lowest proportion redlined area)	4.70	3.80	5.70
				8	2	% of disease Q4 (highest proportion redlined area)	4.50	3.60	6.10
Economic and Social Disadvantage	Cerebrovascular diseases	overall	disease prevalence (%)	9	1	% of disease Q1-Q3 (lowest proportion redlined area)	1.60	1.00	2.20
				9	2	% of disease Q4 (highest proportion redlined area)	1.50	1.00	2.00
Economic and Social Disadvantage	Cerebrovascular diseases	overall	disease prevalence (%)	10	1	% of disease Q1-Q3 (lowest proportion redlined area)	1.40	1.00	1.70
				10	2	% of disease Q4 (highest proportion redlined area)	1.30	0.90	2.00
Economic and Social Disadvantage	Diseases of arteries, arterioles and capillaries	overall	disease prevalence (%)	11	1	% of disease Q1-Q3 (lowest proportion redlined area)	2.00	1.40	2.70
				11	2	% of disease Q4 (highest proportion redlined area)	2.20	1.70	2.70
Economic and Social Disadvantage	Diseases of arteries, arterioles and capillaries	overall	disease prevalence (%)	12	1	% of disease Q1-Q3 (lowest proportion redlined area)	1.90	1.40	2.40
				12	2	% of disease Q4 (highest proportion redlined area)	2.00	1.40	2.70
Economic and Social Disadvantage	Diseases of arteries, arterioles and capillaries	overall	disease prevalence (%)	13	1	% of disease Q1-Q3 (lowest proportion redlined area)	0.80	0.30	1.30
				13	2	% of disease Q4 (highest proportion redlined area)	0.80	0.60	1.30

		Economic and Social Disadvantage	Diseases of arteries, arterioles and capillaries	overall	disease prevalence (%)	14	1	% of disease Q1-Q3 (lowest proportion redlined area)	1.50	1.20	2.00
						14	2	% of disease Q4 (highest proportion redlined area)	1.40	0.90	1.70
[186]	Metcalf, 2008	Economic and Social Disadvantage	Hypertensive diseases	overall	OR (95%CI)	1	1	1&2 (least deprived, reference)	1.00	-	-
						1	2	3&4	1.39	1.03	1.87
						1	3	5&6	1.69	1.25	2.30
						1	4	7&8	1.37	1.02	1.85
						1	5	9&10	1.43	1.04	1.96
		Economic and Social Disadvantage	Diabetes mellitus	overall	OR (95%CI)	2	1	1&2 (least deprived, reference)	1.00	-	-
						2	2	3&4	1.24	0.78	1.98
						2	3	5&6	1.14	0.71	1.85
						2	4	7&8	1.64	1.04	2.59
						2	5	9&10	1.67	1.05	2.68
[187]	Mezuk, 2014	Discrimination and Segregation	Diabetes mellitus	overall	OR (95%CI)	1	1	Iraqi not in an enclave (reference)	1.00	-	-
						1	2	Iraqi living in an enclave	0.98	0.79	1.23
		Discrimination and Segregation	Diabetes mellitus	overall	OR (95%CI)	2	1	Other immigrants not in an enclave (reference)	1.00	-	-
						2	2	Other immigrant living in an Iraqi enclave	1.07	0.97	1.19
		Discrimination and Segregation	Diabetes mellitus	overall	OR (95%CI)	3	1	Swedish not in an enclave (reference)	1.00	-	-
						3	2	Swedish living in an Iraqi enclave	1.23	1.11	1.36
[188]	Mohottige, 2023	Discrimination and Segregation	Diabetes mellitus	overall	PR (95%CI)	1	1	1 SD decrease	1.43	1.37	1.52
		Discrimination and Segregation	Diabetes mellitus	overall	PR (95%CI)	2	1	1 SD decrease	1.35	1.28	1.43
		Economic and Social Disadvantage	Diabetes mellitus	overall	PR (95%CI)	3	1	1 unit increase in score	1.35	1.30	1.43
		Discrimination and Segregation	Hypertensive diseases	overall	PR (95%CI)	4	1	1 SD decrease	1.19	1.14	1.25
		Discrimination and Segregation	Hypertensive diseases	overall	PR (95%CI)	5	1	1 SD decrease	1.14	1.09	1.19
		Economic and Social Disadvantage	Hypertensive diseases	overall	PR (95%CI)	6	1	1 unit increase in score	1.15	1.10	1.19
[189]	Moore, 2014	Civic Participation and Engagement	Hypertensive diseases	overall	Probit regression coefficient (95%CI)	1	1		0.00	-0.02	0.03
		Social Cohesion and Social Capital	Hypertensive diseases	overall	Probit regression coefficient (95%CI)	2	1	higher numbers = greater cohesion	-0.01	-0.04	0.01

[190]	Morenoff, 2007	Economic and Social Disadvantage	Hypertensive diseases	overall	OR (95%CI)	1	1		1.00	0.90	1.20
		Economic and Social Disadvantage	Hypertensive diseases	overall	OR (95%CI)	2	1		0.70	0.60	0.90
		Discrimination and Segregation	Hypertensive diseases	male	OR (95%CI)	3	1	higher values indicate more Hispanic and foreign born and fewer non-Hispanic blacks	0.80	0.70	1.00
[191]	Morris, 2008	Economic and Social Disadvantage	Ischaemic heart diseases	male	IRR (95%CI)	1	1	most deprived vs. least deprived (reference)	1.22	0.93	1.59
[192]	Mujahid, 2008	Crime and Safety	Hypertensive diseases	overall	RP (95%CI)	1	1	90th (greater saffety) vs. 10th (reference)	0.93	0.83	1.04
		Social Cohesion and Social Capital	Hypertensive diseases	overall	RP (95%CI)	2	1	90st (greater cohesion) vs. 10th (reference)	1.02	0.90	1.15
[193]	Müller, 2013	Economic and Social Disadvantage	Diabetes mellitus	female	OR (95%CI)	1	1	low (reference)	1.00	-	-
						1	2	medium	1.36	0.96	1.93
						1	3	high	1.13	0.79	1.62
		Economic and Social Disadvantage	Diabetes mellitus	male	OR (95%CI)	2	1	low (reference)	1.00	-	-
						2	2	medium	1.08	0.83	1.40
						2	3	high	1.52	1.18	1.96
[194]	Müller, 2013	Economic and Social Disadvantage	Diabetes mellitus	overall	OR (95%CI)	1	1	Q1 (low, reference)	1.00	-	-
						1	2	Q2	1.31	1.00	1.72
						1	3	Q3	1.39	1.07	1.82
						1	4	Q4	1.62	1.25	2.09
						1	5	Q5	1.44	1.10	1.87
		Discrimination and Segregation	Diabetes mellitus	overall	OR (95%CI)	2	1	Q1 (reference)	1.00	-	-
						2	2	Q2	1.20	0.93	1.56
						2	3	Q3	1.07	0.83	1.38
						2	4	Q4	1.11	0.86	1.43
						2	5	Q5	1.16	0.89	1.51
[195]	Murray, 2010	Economic and Social Disadvantage	Hypertensive diseases	female	RP (95%CI)	1	1	90st (greater poverty) vs. 10th (reference)	1.10	0.80	1.40
		Economic and Social Disadvantage	Hypertensive diseases	female	RP (95%CI)	2	1	90st (greater poverty) vs. 10th (reference)	1.00	0.70	1.40
		Economic and Social Disadvantage	Diabetes mellitus	female	RP (95%CI)	3	1	90st (greater poverty) vs. 10th (reference)	1.30	0.90	1.80

		Economic and Social Disadvantage	Diabetes mellitus	female	RP (95%CI)	4	1	90st (greater poverty) vs. 10th (reference)	1.90	1.40	2.30
[196]	Nakagomi, 2019	Social Cohesion and Social Capital	Hypertensive diseases	male	PR (95%CI)	1	1		0.98	0.96	1.01
		Social Cohesion and Social Capital	Hypertensive diseases	male	PR (95%CI)	2	1		0.99	0.97	1.01
		Social Cohesion and Social Capital	Hypertensive diseases	male	PR (95%CI)	3	1		1.01	0.99	1.03
		Social Cohesion and Social Capital	Hypertensive diseases	female	PR (95%CI)	4	1		0.97	0.95	0.99
		Social Cohesion and Social Capital	Hypertensive diseases	female	PR (95%CI)	5	1		1.00	0.98	1.02
		Social Cohesion and Social Capital	Hypertensive diseases	female	PR (95%CI)	6	1		1.00	0.98	1.03
[197]	Nazmi, 2010	Economic and Social Disadvantage	Diabetes mellitus	overall	%	1	1	I- Least deprived	8.70	not reported	not reported
						1	2	II	10.40	not reported	not reported
						1	3	III	12.70	not reported	not reported
						1	4	IV- Most deprived	15.70	not reported	not reported
[198]	Neufcourt, 2019	Economic and Social Disadvantage	Hypertensive diseases	male	OR (95%CI)	1	1		1.03	1.01	1.05
		Economic and Social Disadvantage	Hypertensive diseases	male	OR (95%CI)	2	1	Q3 vs. Q1	1.06	1.01	1.11
		Economic and Social Disadvantage	Hypertensive diseases	female	OR (95%CI)	3	1		1.03	1.01	1.06
		Economic and Social Disadvantage	Hypertensive diseases	female	OR (95%CI)	4	1	Q3 vs. Q1	1.06	1.01	1.11
[199]	Nikulina, 2014	Economic and Social Disadvantage	Hypertensive diseases	overall	Beta (95%CI)	1	1	higher = more poverty	-0.14	-0.46	0.18
[200]	Odoi, 2020	Economic and Social Disadvantage	Ischaemic heart diseases	overall	Likelihood ratio test	1	1		1.03	not reported	not reported
		Discrimination and Segregation	Ischaemic heart diseases	overall	RR (95%CI)	2	1		1.00	not reported	not reported
[201]	Ogungbe, 2021	Social Relationships and Norms	Hypertensive diseases	overall	OR (95%CI)	1	1	low social support (reference)	1.00	-	-
						1	2	high social support	1.13	0.70	1.81

		Social Relationships and Norms	Diabetes mellitus	overall	OR (95%CI)	2	1	low social support (reference)	1.00	-	-	
						2	2	high social support	1.13	0.61	2.08	
[202]	Ohanyan, 2022	Economic and Social Disadvantage	Diabetes mellitus	overall	log(OR)	1	1	Increase in average value of homes (x1000 euros)	-0.19	-	-	0.05
		Economic and Social Disadvantage	Diabetes mellitus	overall	log(OR)	2	1	Increase in % of inhabitants with income above 20th percentile	-0.17	-	-	0.05
		Discrimination and Segregation	Diabetes mellitus	overall	log(OR)	3	1	Increase in % of inhabitants with non-western origins	0.13	-	-	0.04
[203]	Oktamianti, 2022	Economic and Social Disadvantage	Hypertensive diseases	overall	OLS (linear regression)	1	1	Q1 (poor, reference)	1.00	-	-	
						1	2	Q2	1.93	-	-	
						1	3	Q3	2.74	-	-	
						1	4	Q4	1.86	-	-	
						1	5	Q5 (rich)	1.07	-	-	
		Economic and Social Disadvantage	Hypertensive diseases	overall	OLS (linear regression)	2	1	Q1 (least educated, reference)	1.00	-	-	
						2	2	Q2	-0.35	-	-	
						2	3	Q3	0.32	-	-	
						2	4	Q4	-0.31	-	-	
						2	5	Q5 (most educated)	0.52	-	-	
		Economic and Social Disadvantage	Hypertensive diseases	female	OLS (linear regression)	3	1	Q1 (poor, reference)	1.00	-	-	
						3	2	Q2	1.35	-	-	
						3	3	Q3	2.23	-	-	
						3	4	Q4	1.69	-	-	
						3	5	Q5 (rich)	2.18	-	-	
		Economic and Social Disadvantage	Hypertensive diseases	female	OLS (linear regression)	4	1	Q1 (poor, reference)	1.00	-	-	
						4	2	Q2	-0.74	-	-	
						4	3	Q3	0.63	-	-	
						4	4	Q4	-0.03	-	-	
						4	5	Q5 (rich)	1.20	-	-	
		Economic and Social Disadvantage	Hypertensive diseases	male	OLS (linear regression)	5	1	Q1 (poor, reference)	1.00	-	-	
						5	2	Q2	2.48	-	-	
						5	3	Q3	3.17	-	-	

						5	4	Q4	2.00	-	-
						5	5	Q5 (rich)	-0.40	-	-
		Economic and Social Disadvantage	Hypertensive diseases	male	OLS (linear regression)	6	1	Q1 (poor, reference)	1.00	-	-
						6	2	Q2	0.19	-	-
						6	3	Q3	0.14	-	-
						6	4	Q4	-0.58	-	-
						6	5	Q5 (rich)	-0.21	-	-
[204]	Oladele, 2020	Social Relationships and Norms	Hypertensive diseases	overall	OR (95%CI)	1	1		0.98	0.93	1.05
		Social Relationships and Norms	Hypertensive diseases	overall	OR (95%CI)	2	1		1.00	1.00	1.00
[205]	Omariba, 2014	Discrimination and Segregation	Diseases of the circulatory system	female	OR (95%CI)	1	1	lowest concentration of immigrants (reference)	1.00	-	-
						1	2	medium	0.92	0.87	0.98
						1	3	highest	0.86	0.79	0.93
		Discrimination and Segregation	Diseases of the circulatory system	male	OR (95%CI)	2	1	lowest (reference)	1.00	-	-
						2	2	medium	0.96	0.91	1.01
						2	3	highest	0.94	0.88	1.01
[206]	Osborn, 2023	Social Cohesion and Social Capital	Hypertensive diseases	overall	OR (95%CI)	1	1	Low cohesion (reference)	1.00	-	-
						1	2	High cohesion	0.84	0.74	0.96
[207]	Pantell, 2019	Economic and Social Disadvantage	Hypertensive diseases	male	HR (95%CI)	1	1	No living in a area with concentrated neighborhood poverty	1.00	-	-
						1	2	living in a area concentrated neighborhood poverty	1.26	1.00	1.59
		Economic and Social Disadvantage	Diabetes mellitus	male	HR (95%CI)	2	1	No living in a area with concentrated neighborhood poverty	1.00	-	-
						2	2	living in a area concentrated neighborhood poverty	1.31	1.07	1.60
[208]	Penninx, 1999	Social Relationships and Norms	Diseases of the circulatory system	overall	Beta	1	1		0.00	not reported	not reported
		Social Relationships and Norms	Diseases of arteries, arterioles and capillaries	overall	Beta	2	1		0.00	not reported	not reported
		Social Relationships and Norms	Cerebrovascular diseases	overall	Beta	3	1		-0.04	not reported	not reported
		Social Relationships and Norms	Diabetes mellitus	male	Beta	4	1		0.01	not reported	not reported

		Social Relationships and Norms	Diseases of the circulatory system	overall	Beta	5	1		0.02	not reported	not reported
		Social Relationships and Norms	Diseases of arteries, arterioles and capillaries	male	Beta	6	1		0.01	not reported	not reported
		Social Relationships and Norms	Cerebrovascular diseases	overall	Beta	7	1		0.03	not reported	not reported
		Social Relationships and Norms	Diabetes mellitus	overall	Beta	8	1		0.03	not reported	not reported
		Social Relationships and Norms	Diseases of the circulatory system	overall	Beta	9	1		0.01	not reported	not reported
		Social Relationships and Norms	Diseases of arteries, arterioles and capillaries	overall	Beta	10	1		0.00	not reported	not reported
		Social Relationships and Norms	Cerebrovascular diseases	male	Beta	11	1		0.02	not reported	not reported
		Social Relationships and Norms	Diabetes mellitus	overall	Beta	12	1		-0.03	not reported	not reported
[209]	Pichora, 2018	Economic and Social Disadvantage	Diabetes mellitus	male	RR (95%CI)	1	1	lowest income vs. highest (reference)	1.52	1.29	1.80
		Economic and Social Disadvantage	Diabetes mellitus	overall	Rate difference (95%CI)	2	1	lowest income vs. highest (reference)	3.00	1.80	4.20
[210]	Pinheiro, 2020	Economic and Social Disadvantage	Other forms of heart disease	overall	HR (95%CI)	1	1		1.21	1.05	1.40
		Social Relationships and Norms	Other forms of heart disease	overall	HR (95%CI)	2	1		1.07	0.82	1.41
		Social Relationships and Norms	Other forms of heart disease	overall	HR (95%CI)	3	1		1.05	0.88	1.26
[211]	Piwońska, 2023	Social Relationships and Norms	Hypertensive diseases	male	p trend	1	1	low social network index (% of individuals with disease outcome)	55.60	-	-
						1	2	moderate social network index (% of individuals with disease outcome)	49.40	-	-
						1	3	high social network index (% of individuals with disease outcome)	34.70	-	-
		Social Relationships and Norms	Diabetes mellitus	male	p trend	2	1	low social network index (% of individuals with disease outcome)	15.20	-	-
						2	2	moderate social network index (% of individuals with disease outcome)	9.40	-	-

						2	3	high social network index (% of individuals with disease outcome)	6.20	-	-
		Social Relationships and Norms	Hypertensive diseases	female	p trend	3	1	low social network index (% of individuals with disease outcome)	50.40	-	-
						3	2	moderate social network index (% of individuals with disease outcome)	34.60	-	-
						3	3	high social network index (% of individuals with disease outcome)	23.30	-	-
		Social Relationships and Norms	Diabetes mellitus	female	p trend	4	1	low social network index (% of individuals with disease outcome)	12.30	-	-
						4	2	moderate social network index (% of individuals with disease outcome)	6.50	-	-
						4	3	high social network index (% of individuals with disease outcome)	3.10	-	-
		Social Relationships and Norms	Diseases of the circulatory system	male	OR (95%CI)	5	1	low social network index	0.85	0.58	1.24
						5	2	moderate social network index	0.97	0.66	1.42
						5	3	high social network index (reference)	1.00	-	-
		Social Relationships and Norms	Ischaemic heart diseases	male	OR (95%CI)	6	1	low social network index	0.79	0.49	1.27
						6	2	moderate social network index	1.02	0.63	1.66
						6	3	high social network index (reference)	1.00	-	-
		Social Relationships and Norms	Diseases of the circulatory system	female	OR (95%CI)	7	1	low social network index	0.76	0.54	1.07
						7	2	moderate social network index	0.93	0.66	1.31
						7	3	high social network index (reference)	1.00	-	-
		Social Relationships and Norms	Ischaemic heart diseases	female	OR (95%CI)	7	1	low social network index	0.61	0.35	1.05
						7	2	moderate social network index	0.70	0.40	1.24
						7	3	high social network index (reference)	1.00	-	-
[212]	Ptushkina, 2021	Economic and Social Disadvantage	Diseases of the circulatory system	overall	OR (95%CI)	1	1	high deprivation (reference)	1.00	-	-
						1	2	low	0.95	0.67	1.34
		Economic and Social Disadvantage	Diseases of the circulatory system	male	OR (95%CI)	2	1	high deprivation (reference)	1.00	-	-
						2	2	low	0.79	0.52	1.20

		Economic and Social Disadvantage	Diseases of the circulatory system	female	OR (95%CI)	3	1	high deprivation (reference)	1.00	-	-
						3	2	low	1.43	0.76	2.70
		Economic and Social Disadvantage	Diabetes mellitus	overall	p-value	1	1	low/middle deprivation (% of individuals with disease outcome)	5.50	-	-
						1	2	high deprivation (% of individuals with disease outcome)	11.30	-	-
[213]	Quashie, 2023	Economic and Social Disadvantage	combined outcome	overall	OR (95%CI)	1	1	Q1 (high neighborhood SEP, reference)	1.00	-	-
						1	2	Q2	1.97	1.10	3.50
						1	3	Q3	1.29	0.70	2.36
						1	4	Q4	1.30	0.72	2.35
						1	5	Q5	1.46	0.82	2.60
[214]	Quiñones, 2021	Discrimination and Segregation	Diabetes mellitus	overall	OLS (linear regression)	1	1	(% population below poverty level)	0.18	-	-
		Discrimination and Segregation	Diabetes mellitus	overall	OLS (linear regression)	2	1	(% population with no higher education)	0.65	-	-
[215]	Rachele, 2016	Economic and Social Disadvantage	Ischaemic heart diseases	male	OR (95%CI)	1	1	Q1 (least disadvantaged, reference)	1.00	-	-
						1	2	Q2	1.00	0.73	1.39
						1	3	Q3	0.96	0.69	1.35
						1	4	Q4	0.97	0.71	1.34
						1	5	Q5 (most disadvantaged)	1.26	0.90	1.78
		Economic and Social Disadvantage	Diabetes mellitus	overall	OR (95%CI)	2	1	Q1 (least disadvantaged, reference)	1.00	-	-
						2	2	Q2	0.94	0.60	1.48
						2	3	Q3	1.35	0.87	2.08
						2	4	Q4	1.93	1.30	2.92
						2	5	Q5 (most disadvantaged)	1.81	1.15	2.83
[216]	Redondo-Sendino, 2005	Social Relationships and Norms	Hypertensive diseases	male	OR (95%CI)	1	1		0.94	0.68	1.31
		Social Relationships and Norms	Hypertensive diseases	female	OR (95%CI)	2	1		1.08	0.83	1.39
[217]	Rethy, 2021	Economic and Social Disadvantage	Other forms of heart disease	overall	OR (95%CI)	1	1	Low poverty (reference)	1.00	-	-
						1	2	Intermediate	1.29	0.99	1.68
						1	3	High	1.68	1.26	2.26

[218]	Riddell, 2004	Economic and Social Disadvantage	Other forms of heart disease	overall	RR (95%CI)	1	1		1.11	1.06	1.14
[219]	Rod, 2011	Social Relationships and Norms	Other forms of heart disease	female	HR (95%CI)	1	1	regular contact with friends: yes (reference)	1.00	-	-
						1	2	regular contact with friends: no	1.19	0.95	1.48
		Social Relationships and Norms	Other forms of heart disease	female	HR (95%CI)	2	1	Very (reference)	1.00	-	-
						2	2	somewhat/not at all	0.93	0.73	1.18
		Social Relationships and Norms	Other forms of heart disease	male	HR (95%CI)	3	1	regular contact with friends: yes (reference)	1.00	-	-
						3	2	regular contact with friends: no	0.94	0.75	1.19
		Social Relationships and Norms	Other forms of heart disease	male	HR (95%CI)	4	1	Very (reference)	1.00	-	-
						4	2	somewhat/not at all	0.95	0.74	1.21
[220]	Rose, 2009	Economic and Social Disadvantage	Ischaemic heart diseases	female	IRR (95%CI)	1	1	low neighborhood median household income	2.05	1.69	2.42
						1	2	medium neighborhood median household income	1.40	1.01	1.79
						1	3	high neighborhood median household income (reference)	1.00	-	-
		Economic and Social Disadvantage	Ischaemic heart diseases	male	IRR (95%CI)	2	1	low neighborhood median household income	1.41	1.05	1.76
						2	2	medium neighborhood median household income	1.43	1.04	1.82
						2	3	high neighborhood median household income (reference)	1.00	-	-
		Economic and Social Disadvantage	Ischaemic heart diseases	female	IRR (95%CI)	3	1	low neighborhood median household income	1.74	1.57	1.91
						3	2	medium neighborhood median household income	1.23	1.07	1.39
						3	3	high neighborhood median household income (reference)	1.00	-	-
		Economic and Social Disadvantage	Ischaemic heart diseases	male	IRR (95%CI)	4	1	low neighborhood median household income	1.22	1.09	1.35
						4	2	medium neighborhood median household income	1.11	0.98	1.24
						4	3	high neighborhood median household income (reference)	1.00	-	-
[221]	Safford, 2021	Economic and Social Disadvantage	Ischaemic heart diseases	overall	HR (95%CI)	1	1		1.00	0.84	1.18
[222]	Salinas, 2017	Social Relationships and Norms	Cerebrovascular diseases	overall	HR (95%CI)	1	1		1.15	0.42	3.19

		Social Relationships and Norms	Cerebrovascular diseases	overall	HR (95%CI)	2	1		0.55	0.35	0.87
		Social Relationships and Norms	Cerebrovascular diseases	overall	HR (95%CI)	3	1		0.80	0.52	1.24
		Social Relationships and Norms	Cerebrovascular diseases	overall	HR (95%CI)	4	1		0.73	0.43	1.23
		Social Relationships and Norms	Cerebrovascular diseases	overall	HR (95%CI)	5	1		0.79	0.48	1.29
[223]	Samuel, 2015	Social Cohesion and Social Capital	Hypertensive diseases	overall	PR (95%CI)	1	1		1.03	0.90	1.18
		Social Cohesion and Social Capital	Hypertensive diseases	overall	PR (95%CI)	2	1		1.04	0.93	1.16
[224]	Savin, 2022	Economic and Social Disadvantage	Hypertensive diseases	overall	OR (95%CI)	1	1	Less deprived (reference)	1.00	-	-
						1	2	More deprived	1.47	1.06	2.04
		Disorder and Incivilities	Hypertensive diseases	overall		2	1	Lesser social disorder	1.00	-	-
						2	2	Greater social disorder	1.25	1.02	1.54
[225]	Schieb, 2013	Economic and Social Disadvantage	Cerebrovascular diseases	overall	values for persistently high-rate cluster	1	1		32475.00	not reported	not reported
		Economic and Social Disadvantage	Cerebrovascular diseases	overall	values for persistently low-rate cluster (and p-value for bivariate comparison with persistently high-rate cluster)	2	1		40698.00	not reported	not reported
		Economic and Social Disadvantage	Cerebrovascular diseases	overall	values for transitioned into high-rate cluster (and p-value for bivariate comparison with persistently high-rate cluster)	3	1		36057.00	not reported	not reported
		Economic and Social Disadvantage	Cerebrovascular diseases	overall	values for transitioned out of high-rate cluster (and p-value for bivariate comparison with persistently high-rate cluster)	4	1		35019.00	not reported	not reported
		Economic and Social Disadvantage	Cerebrovascular diseases	overall	values for persistently high-rate cluster	5	1		30.50	not reported	not reported

		Economic and Social Disadvantage	Cerebrovascular diseases	overall	values for persistently low-rate cluster (and p-value for bivariate comparison with persistently high-rate cluster)	6	1	17.20	not reported	not reported
		Economic and Social Disadvantage	Cerebrovascular diseases	overall	values for transitioned into high-rate cluster (and p-value for bivariate comparison with persistently high-rate cluster)	7	1	28.90	not reported	not reported
		Economic and Social Disadvantage	Cerebrovascular diseases	overall	values for transitioned out of high-rate cluster (and p-value for bivariate comparison with persistently high-rate cluster)	8	1	28.10	not reported	not reported
		Economic and Social Disadvantage	Cerebrovascular diseases	overall	values for persistently high-rate cluster	9	1	35.20	not reported	not reported
		Economic and Social Disadvantage	Cerebrovascular diseases	overall	values for persistently low-rate cluster (and p-value for bivariate comparison with persistently high-rate cluster)	10	1	4.70	not reported	not reported
		Economic and Social Disadvantage	Cerebrovascular diseases	overall	values for transitioned into high-rate cluster (and p-value for bivariate comparison with persistently high-rate cluster)	11	1	27.00	not reported	not reported
		Economic and Social Disadvantage	Cerebrovascular diseases	overall	values for transitioned out of high-rate cluster (and p-value for bivariate comparison with persistently high-rate cluster)	12	1	25.10	not reported	not reported
[226]	Schootman, 2007	Crime and Safety	Diabetes mellitus	overall	OR (95%CI)	1	1	0.61	0.35	1.06
		Social Relationships and Norms	Diabetes mellitus	overall	OR (95%CI)	2	1	1.01	0.53	1.92

[227]	Schwartz, 2022	Economic and Social Disadvantage	Diabetes mellitus	overall	OR (95%CI)	1	1	Quartile 1 (least advantage, reference)	1.00	-	-	
						1	2	Quartile 2	0.94	0.86	1.01	
						1	3	Quartile 3	0.90	0.84	0.97	
						1	4	Quartile 4 (most advantage)	0.79	0.74	0.85	
[228]	Schwartz, 2021	Economic and Social Disadvantage	Diabetes mellitus	overall	OR (95%CI)	1	1	Q1	0.82	0.76	0.88	
						1	2	Q2	0.87	0.81	0.93	
						1	3	Q3	0.89	0.83	0.96	
						1	4	Q4 (worst deprivation, reference)	1.00	-	-	
[229]	Sharma, 2023	Economic and Social Disadvantage	Hypertensive diseases	overall	OR (95%CI)	1	1	highly deprived (reference)	1.00	-	-	
						1	2	moderate deprived	1.41	1.26	1.85	
						1	3	less deprived	1.52	1.26	1.85	
						1	4	least deprived	2.25	1.85	2.73	
[230]	Sharp, 2023	Civic Participation and Engagement	Hypertensive diseases	overall	Average marginal effects of OR	1	1		-0.03	-0.05	0.00	
		Social Cohesion and Social Capital	Hypertensive diseases	overall	Average marginal effects of OR	2	1		0.00	-0.03	0.03	
		Economic and Social Disadvantage	Hypertensive diseases	overall	Average marginal effects of OR	3	1		0.05	0.01	0.09	
		Discrimination and Segregation	Hypertensive diseases	overall	Average marginal effects of OR	4	1		-0.01	-0.02	0.00	
[231]	Sheets, 2017	Economic and Social Disadvantage	Diabetes mellitus	overall	OR (95%CI)	1	1	Q1 (least disadvantaged, reference)	1.00	-	-	
						1	2	Q2	1.32	not reported	not reported	
						1	3	Q3	1.35	not reported	not reported	
						1	4	Q4	1.29	not reported	not reported	
						1	5	Q5	1.39	not reported	not reported	
[232]	Shibayama, 2018	Civic Participation and Engagement	Diabetes mellitus	overall	HR (95%CI)	1	1	no (reference)	0.89	0.87	0.92	
		Social Relationships and Norms	Diabetes mellitus	overall	HR (95%CI)	2	1	no (reference)	0.97	0.95	1.00	
[233]	Siegel, 2015	Economic and Social Disadvantage	Hypertensive diseases	male	OR (95%CI)	1	1		-0.03	not reported	not reported	0.01

		Economic and Social Disadvantage	Hypertensive diseases	female	OR (95%CI)	2	1		-0.04	not reported	not reported	0.01
[234]	Singh, 2016	Economic and Social Disadvantage	Diabetes mellitus	overall	OR (95%CI)	1	1		0.30	not reported	not reported	
[235]	Smith, 1998	Economic and Social Disadvantage	Ischaemic heart diseases	male	%	1	1	1	17.60	not reported	not reported	
						1	2	2	12.40	not reported	not reported	
						1	3	3	15.90	not reported	not reported	
						1	4	4	17.30	not reported	not reported	
						1	5	5	18.10	not reported	not reported	
						1	6	6	18.10	not reported	not reported	
						1	7	7	27.80	not reported	not reported	
		Economic and Social Disadvantage	Ischaemic heart diseases	male	%	2	1	1	10.50	not reported	not reported	
						2	2	2	6.90	not reported	not reported	
						2	3	3	11.20	not reported	not reported	
						2	4	4	10.80	not reported	not reported	
						2	5	5	11.30	not reported	not reported	
						2	6	6	9.20	not reported	not reported	
						2	7	7	11.30	not reported	not reported	
		Economic and Social Disadvantage	Ischaemic heart diseases	female	%	3	1	1	16.90	not reported	not reported	
						3	2	2	11.40	not reported	not reported	
						3	3	3	15.60	not reported	not reported	
						3	4	4	14.90	not reported	not reported	
						3	5	5	18.20	not reported	not reported	
						3	6	6	18.90	not reported	not reported	
						3	7	7	19.70	not reported	not reported	
		Economic and Social Disadvantage	Ischaemic heart diseases	female	%	4	1	1	10.00	not reported	not reported	
						4	2	2	8.50	not reported	not reported	

						4	3	3	9.60	not reported	not reported	
						4	4	4	10.10	not reported	not reported	
						4	5	5	9.80	not reported	not reported	
						4	6	6	10.00	not reported	not reported	
						4	7	7	13.10	not reported	not reported	
[236]	Smurthwaite, 2017	Economic and Social Disadvantage	Diseases of the circulatory system	overall	correlation	1	1		-0.13	not reported	not reported	
		Economic and Social Disadvantage	Diabetes mellitus	overall	correlation	2	1		-0.19	not reported	not reported	
[237]	Spicer, 1993	Social Relationships and Norms	Ischaemic heart diseases	male	Beta	1	1		< -0.01	not reported	not reported	
		Social Relationships and Norms	Ischaemic heart diseases	female	Beta	2	1		−0.049	not reported	not reported	
[238]	Splan, 2021	Discrimination and Segregation	Other forms of heart disease	overall	Prevalence rates (betas)	1	1		6.73	2.75	10.72	
		Discrimination and Segregation	Other forms of heart disease	overall	Prevalence rates (betas)	2	1		0.12	0.09	0.15	
		Discrimination and Segregation	Cerebrovascular diseases	overall	Prevalence rates (betas)	3	1		1.66	0.20	3.12	
		Discrimination and Segregation	Cerebrovascular diseases	overall	Prevalence rates (betas)	4	1		0.08	0.07	0.09	
		Discrimination and Segregation	Diabetes mellitus	overall	Prevalence rates (betas)	5	1		-2.24	-6.97	2.49	
		Discrimination and Segregation	Diabetes mellitus	overall	Prevalence rates (betas)	6	1		0.04	0.01	0.08	
[239]	Steckel, 2013	Economic and Social Disadvantage	Diabetes mellitus	overall	Beta (SE)	1	1		0.33	not reported	not reported	0.55
		Discrimination and Segregation	Diabetes mellitus	overall	Beta (SE)	2	1		0.54	not reported	not reported	0.02
[240]	Suchy-Dicey, 2022	Social Relationships and Norms	Diseases of the circulatory system	overall	HR (95%CI)	1	1	lower social support	1.00	-	-	
						1	2	higher social support	0.79	0.70	0.89	
[241]	Sun, 2020	Discrimination and Segregation	Diseases of the circulatory system	overall	OR (95%CI)	1	1		1.00	1.00	1.00	
		Discrimination and Segregation	Diseases of the circulatory system	overall	OR (95%CI)	2	1		1.00	0.99	1.00	
		Discrimination and Segregation	Diseases of the circulatory system	overall	OR (95%CI)	3	1		1.00	1.00	1.00	
		Economic and Social Disadvantage	Diseases of the circulatory system	overall	OR (95%CI)	4	1		1.00	1.00	1.01	
		Economic and Social Disadvantage	Diseases of the circulatory system	overall	OR (95%CI)	5	1		1.01	1.00	1.02	

		Economic and Social Disadvantage	Diseases of the circulatory system	overall	OR (95%CI)	6	1		0.99	0.98	0.99
		Discrimination and Segregation	Ischaemic heart diseases	overall	OR (95%CI)	7	1		1.00	1.00	1.00
		Discrimination and Segregation	Ischaemic heart diseases	overall	OR (95%CI)	8	1		0.99	0.99	1.00
		Discrimination and Segregation	Ischaemic heart diseases	overall	OR (95%CI)	9	1		1.00	1.00	1.00
		Economic and Social Disadvantage	Ischaemic heart diseases	overall	OR (95%CI)	10	1		1.00	1.00	1.01
		Economic and Social Disadvantage	Ischaemic heart diseases	overall	OR (95%CI)	11	1		1.01	1.00	1.02
		Economic and Social Disadvantage	Ischaemic heart diseases	overall	OR (95%CI)	12	1		0.99	0.98	0.99
		Discrimination and Segregation	Ischaemic heart diseases	overall	OR (95%CI)	13	1		1.00	1.00	1.00
		Discrimination and Segregation	Ischaemic heart diseases	overall	OR (95%CI)	14	1		0.99	0.99	1.00
		Discrimination and Segregation	Ischaemic heart diseases	overall	OR (95%CI)	15	1		1.00	1.00	1.00
		Economic and Social Disadvantage	Ischaemic heart diseases	overall	OR (95%CI)	16	1		1.00	1.00	1.01
		Economic and Social Disadvantage	Ischaemic heart diseases	overall	OR (95%CI)	17	1		1.01	1.00	1.03
		Economic and Social Disadvantage	Ischaemic heart diseases	overall	OR (95%CI)	18	1		0.98	0.99	0.99
		Discrimination and Segregation	Cerebrovascular diseases	overall	OR (95%CI)	19	1		1.00	1.00	1.01
		Discrimination and Segregation	Cerebrovascular diseases	overall	OR (95%CI)	20	1		1.01	1.00	1.01
		Discrimination and Segregation	Cerebrovascular diseases	overall	OR (95%CI)	21	1		1.00	1.00	1.00
		Economic and Social Disadvantage	Cerebrovascular diseases	overall	OR (95%CI)	22	1		1.00	1.00	1.00
		Economic and Social Disadvantage	Cerebrovascular diseases	overall	OR (95%CI)	23	1		1.01	1.00	1.02
		Economic and Social Disadvantage	Cerebrovascular diseases	overall	OR (95%CI)	24	1		0.99	0.99	1.00
[242]	Sundquist, 2004	Economic and Social Disadvantage	Ischaemic heart diseases	female	OR (95%CI)	1	1	1 decile (most affluent, reference)	1.00	-	-
						1	2	2	1.07	0.98	1.17
						1	3	3	1.13	1.04	1.24
						1	4	4	1.28	1.17	1.39

						1	5	5	1.23	1.13	1.35	
						1	6	6	1.33	1.21	1.45	
						1	7	7	1.33	1.22	1.45	
						1	8	8	1.39	1.27	1.52	
						1	9	9	1.52	1.40	1.66	
						1	10	10 (most deprived)	1.87	1.72	2.03	
		Economic and Social Disadvantage	Ischaemic heart diseases	male	OR (95%CI)	2	1	1 decile (most affluent, reference)	1.00	-	-	
						2	2	2	1.01	0.96	1.07	
						2	3	3	1.02	0.97	1.08	
						2	4	4	1.10	1.04	1.16	
						2	5	5	1.10	1.04	1.16	
						2	6	6	1.15	1.09	1.21	
						2	7	7	1.16	1.10	1.23	
						2	8	8	1.19	1.13	1.25	
						2	9	9	1.26	1.20	1.33	
						2	10	10 (most deprived)	1.42	1.35	1.49	
[243]	Swain, 2019	Economic and Social Disadvantage	Hypertensive diseases	female	Beta (SE)	1	1		-0.42	not reported	not reported	0.08
		Economic and Social Disadvantage	Hypertensive diseases	female	Beta (SE)	2	1		0.44	not reported	not reported	0.10
		Economic and Social Disadvantage	Hypertensive diseases	female	Beta (SE)	3	1		-0.09	not reported	not reported	0.03
		Economic and Social Disadvantage	Hypertensive diseases	male	Beta (SE)	4	1		-0.22	not reported	not reported	0.14
		Economic and Social Disadvantage	Hypertensive diseases	male	Beta (SE)	5	1		0.32	not reported	not reported	0.02
[244]	Tang, 2015	Economic and Social Disadvantage	Ischaemic heart diseases	overall	RR (95%CI)	1	1	Low (reference)	1.00	-	-	
						1	2	medium-income	1.36	0.91	2.03	
						1	3	high-income	1.66	1.13	2.44	
		Economic and Social Disadvantage	Cerebrovascular diseases	overall	RR (95%CI)	2	1	Low (reference)	1.00	-	-	
						2	2	medium-income	1.21	0.87	1.68	
						2	3	high-income	1.55	1.13	2.13	
[245]	Tapager, 2023	Economic and Social Disadvantage	Diabetes mellitus	overall	linear regression slope coefficient	1	1	least disadvantaged municipality	1.00	-	-	

						1	2	most disadvantaged municipality	1.23	0.97	1.49
[246]	Terashima, 2014	Economic and Social Disadvantage	Ischaemic heart diseases	overall	OR (95%CI)	1	1	Q1 (reference)	1.00	-	-
						1	2	Q5 (lowest quintile)	0.74	0.48	1.16
		Economic and Social Disadvantage	Diabetes mellitus	overall	OR (95%CI)	2	1	Q1 (reference)	1.00	-	-
						2	2	Q5 (lowest quintile)	1.62	1.10	2.38
		Economic and Social Disadvantage	Cerebrovascular diseases	overall	OR (95%CI)	3	1	Q1 (reference)	1.00	-	-
						3	2	Q5 (lowest quintile)	0.69	0.24	1.98
[247]	Thrift, 2006	Economic and Social Disadvantage	Cerebrovascular diseases	overall	Incidence per 100,000/year	1	1	Least disadvantaged	146.00	122.00	169.00
						1	2	Less disadvantaged	181.00	155.00	207.00
						1	3	Disadvantaged	223.00	194.00	252.00
						1	4	Most disadvantaged	280.00	247.00	313.00
[248]	Tompkins, 2010	Discrimination and Segregation	Diabetes mellitus	overall	correlation	1	1		−0.05	not reported	not reported
		Discrimination and Segregation	Diabetes mellitus	overall	correlation	2	1		0.03	not reported	not reported
		Economic and Social Disadvantage	Diabetes mellitus	overall	correlation	3	1		0.33	not reported	not reported
		Economic and Social Disadvantage	Diabetes mellitus	overall	correlation	4	1		0.19	not reported	not reported
		Economic and Social Disadvantage	Diabetes mellitus	overall	correlation	5	1		0.81	not reported	not reported
		Economic and Social Disadvantage	Diabetes mellitus	overall	correlation	6	1		-0.86	not reported	not reported
		Economic and Social Disadvantage	Diabetes mellitus	overall	correlation	7	1		0.42	not reported	not reported
		Economic and Social Disadvantage	Diabetes mellitus	overall	correlation	8	1		0.21	not reported	not reported
		Economic and Social Disadvantage	Diabetes mellitus	overall	correlation	9	1		-0.54	not reported	not reported
		Economic and Social Disadvantage	Diabetes mellitus	overall	correlation	10	1		-0.67	not reported	not reported
[249]	Trifan, 2023	Social Relationships and Norms	Cerebrovascular diseases	overall	OR (95%CI)	1	1	upper tertiles of social support (reference)	1.00	-	-
						1	2	lower tertile of social support	1.30	1.10	1.60

[250]	Tung, 2018	Crime and Safety	Hypertensive diseases	male	Beta (95%CI)	1	1	Low (reference)	1.00	-	-
						1	2	Medium	1.18	0.95	1.46
						1	3	High	1.28	1.01	1.62
						1	4	Very high	1.23	0.95	1.59
		Crime and Safety	Hypertensive diseases	female	Beta (95%CI)	2	1	Low (reference)	1.00	-	-
						2	2	Medium	1.07	0.79	1.44
						2	3	High	1.13	0.81	1.58
						2	4	Very high	1.32	0.91	1.90
[251]	Tung, 2019	Crime and Safety	Hypertensive diseases	overall	OR (95%CI)	1	1		1.03	1.01	1.06
		Crime and Safety	Hypertensive diseases	overall	OR (95%CI)	2	1		1.01	0.97	1.05
		Crime and Safety	Diseases of the circulatory system	overall	OR (95%CI)	3	1		1.06	1.01	1.12
		Crime and Safety	Diseases of the circulatory system	overall	OR (95%CI)	4	1		1.09	0.94	1.27
[252]	Uddin, 2022	Economic and Social Disadvantage	Diabetes mellitus	overall	PR (95%CI)	1	1	Quartile 1 (most advantage, reference)	1.00	-	-
						1	2	Quartile 2	1.06	0.90	1.25
						1	3	Quartile 3	1.10	0.93	1.30
						1	4	Quartile 4 (most disadvantage)	1.16	0.97	1.38
		Economic and Social Disadvantage	Diabetes mellitus	overall	PR (95%CI)	2	1	Quartile 1 (most advantage, reference)	1.00	-	-
						2	2	Quartile 2	1.22	1.04	1.42
						2	3	Quartile 3	1.46	1.26	1.70
						2	4	Quartile 4 (most disadvantage)	1.50	1.29	1.75
		Economic and Social Disadvantage	Diabetes mellitus	overall	PR (95%CI)	3	1	Quartile 1 (most advantage, reference)	1.00	-	-
						3	2	Quartile 2	1.16	0.91	1.47
						3	3	Quartile 3	1.31	1.05	1.64
						3	4	Quartile 4 (most disadvantage)	1.54	1.24	1.92
		Economic and Social Disadvantage	Diabetes mellitus	overall	PR (95%CI)	4	1	Quartile 1 (most advantage, reference)	1.00	-	-
						4	2	Quartile 2	1.06	0.85	1.32
						4	3	Quartile 3	1.27	1.03	1.56
						4	4	Quartile 4 (most disadvantage)	1.10	0.99	1.48
[253]	Uddin, 2023	Economic and Social Disadvantage	Diabetes mellitus	male	OR (95%CI)	1	1	Q1 (better neighborhood socioeconomic environment, reference)	1.00	-	-
						1	2	Q2	1.23	0.92	1.65

				1	3	Q3	0.97	0.73	1.30
				1	4	Q4 (worse neighborhood socioeconomic environment)	0.69	0.46	1.05
Economic and Social Disadvantage	Diabetes mellitus	male	RR (95%CI)	2	1	Q1 (better neighborhood socioeconomic environment, reference)	1.00	-	-
				2	2	Q2	1.44	0.75	2.77
				2	3	Q3	1.59	0.82	3.08
				2	4	Q4 (worse neighborhood socioeconomic environment)	1.44	0.71	2.91
Economic and Social Disadvantage	Diabetes mellitus	male	RR (95%CI)	3	1	Q1 (better neighborhood socioeconomic environment, reference)	1.00	-	-
				3	2	Q2	1.06	1.02	1.11
				3	3	Q3	1.08	1.03	1.14
				3	4	Q4 (worse neighborhood socioeconomic environment)	1.07	1.00	1.15
Economic and Social Disadvantage	Diabetes mellitus	male	OR (95%CI)	4	1	Q1 (better neighborhood socioeconomic environment, reference)	1.00	-	-
				4	2	Q2	1.40	1.08	1.80
				4	3	Q3	1.55	1.19	2.02
				4	4	Q4 (worse neighborhood socioeconomic environment)	1.49	1.14	1.94
Economic and Social Disadvantage	Diabetes mellitus	male	RR (95%CI)	5	1	Q1 (better neighborhood socioeconomic environment, reference)	1.00	-	-
				5	2	Q2	0.96	0.66	1.37
				5	3	Q3	1.13	0.79	1.62
				5	4	Q4 (worse neighborhood socioeconomic environment)	1.04	0.71	1.54
Economic and Social Disadvantage	Diabetes mellitus	male	RR (95%CI)	6	1	Q1 (better neighborhood socioeconomic environment, reference)	1.00	-	-
				6	2	Q2	1.05	1.03	1.08
				6	3	Q3	1.08	1.04	1.12
				6	4	Q4 (worse neighborhood socioeconomic environment)	1.05	1.01	1.09
Economic and Social Disadvantage	Diabetes mellitus	male	OR (95%CI)	7	1	Q1 (better neighborhood socioeconomic environment, reference)	1.00	-	-
				7	2	Q2	0.96	0.82	1.14
				7	3	Q3	1.08	0.92	1.27

				7	4	Q4 (worse neighborhood socioeconomic environment)	0.96	0.80	1.15
Economic and Social Disadvantage	Diabetes mellitus	male	RR (95%CI)	8	1	Q1 (better neighborhood socioeconomic environment, reference)	1.00	-	-
				8	2	Q2	0.78	0.46	1.32
				8	3	Q3	1.12	0.73	1.72
				8	4	Q4 (worse neighborhood socioeconomic environment)	1.14	0.72	1.79
Economic and Social Disadvantage	Diabetes mellitus	male	RR (95%CI)	9	1	Q1 (better neighborhood socioeconomic environment, reference)	1.00	-	-
				9	2	Q2	1.04	1.02	1.07
				9	3	Q3	1.07	1.04	1.11
				9	4	Q4 (worse neighborhood socioeconomic environment)	1.09	1.05	1.13
Economic and Social Disadvantage	Diabetes mellitus	male	OR (95%CI)	10	1	Q1 (better neighborhood socioeconomic environment, reference)	1.00	-	-
				10	2	Q2	1.03	0.91	1.18
				10	3	Q3	1.11	1.01	1.22
				10	4	Q4 (worse neighborhood socioeconomic environment)	1.13	1.02	1.26
Economic and Social Disadvantage	Diabetes mellitus	male	RR (95%CI)	11	1	Q1 (better neighborhood socioeconomic environment, reference)	1.00	-	-
				11	2	Q2	1.97	1.19	3.28
				11	3	Q3	1.60	0.94	2.73
				11	4	Q4 (worse neighborhood socioeconomic environment)	1.23	0.67	2.26
Economic and Social Disadvantage	Diabetes mellitus	male	RR (95%CI)	12	1	Q1 (better neighborhood socioeconomic environment, reference)	1.00	-	-
				12	2	Q2	1.03	1.01	1.05
				12	3	Q3	1.06	1.03	1.08
				12	4	Q4 (worse neighborhood socioeconomic environment)	1.06	1.03	1.09
Economic and Social Disadvantage	Diabetes mellitus	female	OR (95%CI)	13	1	Q1 (better neighborhood socioeconomic environment, reference)	1.00	-	-
				13	2	Q2	1.06	0.83	1.37
				13	3	Q3	1.14	0.87	1.50

				13	4	Q4 (worse neighborhood socioeconomic environment)	0.89	0.66	1.20
Economic and Social Disadvantage	Diabetes mellitus	female	RR (95%CI)	14	1	Q1 (better neighborhood socioeconomic environment, reference)	1.00	-	-
				14	2	Q2	1.56	0.90	2.70
				14	3	Q3	1.84	1.05	3.23
				14	4	Q4 (worse neighborhood socioeconomic environment)	1.45	0.80	2.62
Economic and Social Disadvantage	Diabetes mellitus	female	RR (95%CI)	15	1	Q1 (better neighborhood socioeconomic environment, reference)	1.00	-	-
				15	2	Q2	1.03	0.90	1.17
				15	3	Q3	1.13	0.99	1.28
				15	4	Q4 (worse neighborhood socioeconomic environment)	1.11	0.97	1.27
Economic and Social Disadvantage	Diabetes mellitus	female	OR (95%CI)	16	1	Q1 (better neighborhood socioeconomic environment, reference)	1.00	-	-
				16	2	Q2	1.72	1.15	2.57
				16	3	Q3	1.83	1.23	2.72
				16	4	Q4 (worse neighborhood socioeconomic environment)	1.88	1.25	2.83
Economic and Social Disadvantage	Diabetes mellitus	female	RR (95%CI)	17	1	Q1 (better neighborhood socioeconomic environment, reference)	1.00	-	-
				17	2	Q2	1.44	0.89	2.31
				17	3	Q3	2.09	1.33	3.27
				17	4	Q4 (worse neighborhood socioeconomic environment)	2.20	1.37	3.53
Economic and Social Disadvantage	Diabetes mellitus	female	RR (95%CI)	18	1	Q1 (better neighborhood socioeconomic environment, reference)	1.00	-	-
				18	2	Q2	1.12	1.04	1.20
				18	3	Q3	1.21	1.13	1.31
				18	4	Q4 (worse neighborhood socioeconomic environment)	1.25	1.15	1.36
Economic and Social Disadvantage	Diabetes mellitus	female	OR (95%CI)	19	1	Q1 (better neighborhood socioeconomic environment, reference)	1.00	-	-
				19	2	Q2	1.09	0.94	1.27
				19	3	Q3	1.20	1.03	1.40

						19	4	Q4 (worse neighborhood socioeconomic environment)	1.34	1.12	1.60
		Economic and Social Disadvantage	Diabetes mellitus	female	RR (95%CI)	20	1	Q1 (better neighborhood socioeconomic environment, reference)	1.00	-	-
						20	2	Q2	1.34	0.69	2.59
						20	3	Q3	1.34	0.71	2.51
						20	4	Q4 (worse neighborhood socioeconomic environment)	0.90	1.01	3.54
		Economic and Social Disadvantage	Diabetes mellitus	female	RR (95%CI)	21	1	Q1 (better neighborhood socioeconomic environment, reference)	1.00	-	-
						21	2	Q2	1.18	1.07	1.29
						21	3	Q3	1.24	1.13	1.37
						21	4	Q4 (worse neighborhood socioeconomic environment)	1.31	1.19	1.45
		Economic and Social Disadvantage	Diabetes mellitus	female	OR (95%CI)	22	1	Q1 (better neighborhood socioeconomic environment, reference)	1.00	-	-
						22	2	Q2	1.00	0.89	1.13
						22	3	Q3	1.15	1.03	1.28
						22	4	Q4 (worse neighborhood socioeconomic environment)	1.12	0.99	1.27
		Economic and Social Disadvantage	Diabetes mellitus	female	RR (95%CI)	23	1	Q1 (better neighborhood socioeconomic environment, reference)	1.00	-	-
						23	2	Q2	1.27	0.73	2.23
						23	3	Q3	1.27	0.73	2.22
						23	4	Q4 (worse neighborhood socioeconomic environment)	1.35	0.75	2.45
		Economic and Social Disadvantage	Diabetes mellitus	female	RR (95%CI)	24	1	Q1 (better neighborhood socioeconomic environment, reference)	1.00	-	-
						24	2	Q2	1.09	0.99	1.19
						24	3	Q3	1.15	1.05	1.23
						24	4	Q4 (worse neighborhood socioeconomic environment)	1.28	1.17	1.40
[254]	Usher, 2018	Economic and Social Disadvantage	Hypertensive diseases	overall	OR (95%CI)	1	1	(poor = reference)	1.21	0.87	1.68
		Discrimination and Segregation	Hypertensive diseases	overall	OR (95%CI)	2	1	Predominantly White neighborhood (reference)	1.00	-	-
						2	2	Predominantly Black neighborhood	0.73	0.43	1.23

						2	3	Predominantly Other race neighborhood	1.02	0.48	2.17
						2	4	Integrated neighborhood	0.83	0.59	1.15
[255]	Vart, 2017	Economic and Social Disadvantage	Diseases of arteries, arterioles and capillaries	overall	HR (95%CI)	1	1	Q1 (least deprived, reference)	1.00	-	-
						1	2	Q2	1.27	0.91	1.78
						1	3	Q3	1.03	0.72	1.47
						1	4	Q4	1.27	0.85	1.91
						1	5	Q5 (most deprived)	1.33	0.81	2.17
[256]	Villani, 2018	Discrimination and Segregation	Diabetes mellitus	overall	IR (95%CI)	1	1	1 (low proportion overseas-born residents, reference)	1.00	-	-
						1	2	2	1.29	0.97	1.72
						1	3	3	1.60	1.18	2.18
						1	4	4	1.86	1.27	2.65
						1	5	5 (high proportion overseas-born residents)	2.02	1.37	2.91
		Economic and Social Disadvantage	Diabetes mellitus	overall	IR (95%CI)	2	1	1 (most disadvantaged, reference)	1.00	-	-
						2	2	2	0.89	0.68	1.16
						2	3	3	0.91	0.68	1.22
						2	4	4	0.89	0.63	1.24
						2	5	5 (least disadvantaged)	0.70	0.51	0.96
[257]	Vintimilla, 2023	Economic and Social Disadvantage	Hypertensive diseases	overall	OR (95%CI)	1	1	Quartile 1 (least deprived, reference)	1.00	-	-
						1	2	Quartile 4 (most deprived)	1.18	0.71	1.96
		Economic and Social Disadvantage	Hypertensive diseases	overall	OR (95%CI)	2	1	Quartile 1 (least deprived, reference)	1.00	-	-
						2	2	Quartile 4 (most deprived)	2.14	1.31	3.48
		Economic and Social Disadvantage	Diabetes mellitus	overall	OR (95%CI)	3	1	Quartile 1 (least deprived, reference)	1.00	-	-
						3	2	Quartile 4 (most deprived)	1.66	0.98	2.81
		Economic and Social Disadvantage	Diabetes mellitus	overall	OR (95%CI)	4	1	Quartile 1 (least deprived, reference)	1.00	-	-
						4	2	Quartile 4 (most deprived)	3.42	1.67	7.01
[258]	Vogt, 1992	Social Relationships and Norms	Ischaemic heart diseases	overall	RH (95%CI)	1	1	T1 (lowest) vs. T3 (highest, reference)	1.50	1.00	2.30
		Social Relationships and Norms	Ischaemic heart diseases	overall	RH (95%CI)	2	1	T1 (lowest) vs. T3 (highest, reference)	1.10	0.80	1.50

		Social Relationships and Norms	Ischaemic heart diseases	overall	RH (95%CI)	3	1	T1 (lowest) vs. T3 (highest, reference)	1.20	0.90	1.60
		Social Relationships and Norms	Hypertensive diseases	overall	RH (95%CI)	4	1	T1 (lowest) vs. T3 (highest, reference)	0.90	0.70	1.20
		Social Relationships and Norms	Hypertensive diseases	overall	RH (95%CI)	5	1	T1 (lowest) vs. T3 (highest, reference)	0.90	0.80	1.20
		Social Relationships and Norms	Hypertensive diseases	overall	RH (95%CI)	6	1	T1 (lowest) vs. T3 (highest, reference)	1.00	0.80	1.20
		Social Relationships and Norms	Cerebrovascular diseases	overall	RH (95%CI)	7	1	T1 (lowest) vs. T3 (highest, reference)	1.00	0.60	1.70
		Social Relationships and Norms	Cerebrovascular diseases	overall	RH (95%CI)	8	1	T1 (lowest) vs. T3 (highest, reference)	0.90	0.60	1.30
		Social Relationships and Norms	Cerebrovascular diseases	overall	RH (95%CI)	9	1	T1 (lowest) vs. T3 (highest, reference)	0.90	0.60	1.30
[259]	Wagner, 2016	Economic and Social Disadvantage	Hypertensive diseases	overall	OR (95%CI)	1	1	>= 12 years (reference)	1.00	-	-
						1	2	9-11 years	1.20	0.85	1.68
						1	3	<= 8 years	1.42	0.95	2.12
[260]	Walter, 2019	Social Relationships and Norms	Hypertensive diseases	female	OR (95%CI)	1	1		0.82	0.66	1.01
[261]	Wang, 2021	Economic and Social Disadvantage	Hypertensive diseases	overall	Spatial Durbin error model	1	1	% change in disease prevalence per unit increase in exposure	-2.95	-5.46	-0.45
		Economic and Social Disadvantage	Hypertensive diseases	overall	Spatial Durbin error model	2	1	% change in disease prevalence per unit increase in exposure	-0.47	-5.70	4.75
[262]	Wang, 2019	Economic and Social Disadvantage	Diseases of the circulatory system	overall	Standard coefficient from spatial regression	1	1		0.31	not reported	not reported
		Economic and Social Disadvantage	Hypertensive diseases	overall	Standard coefficient from spatial regression	2	1		0.05	not reported	not reported
[263]	Welin, 1996	Civic Participation and Engagement	Ischaemic heart diseases	male	OR (95%CI)	1	1		1.06	0.71	1.59
		Social Relationships and Norms	Ischaemic heart diseases	male	OR (95%CI)	2	1		1.12	0.72	1.72
		Civic Participation and Engagement	Ischaemic heart diseases	male	OR (95%CI)	3	1		1.09	0.70	1.67
		Social Relationships and Norms	Ischaemic heart diseases	male	OR (95%CI)	4	1		0.84	0.52	1.36

		Civic Participation and Engagement	Ischaemic heart diseases	male	OR (95%CI)	5	1		1.20	0.78	1.84
		Social Relationships and Norms	Ischaemic heart diseases	male	OR (95%CI)	6	1		1.55	1.03	2.35
		Civic Participation and Engagement	Ischaemic heart diseases	male	OR (95%CI)	7	1		0.91	0.57	1.44
		Social Relationships and Norms	Ischaemic heart diseases	male	OR (95%CI)	8	1		2.10	1.32	3.34
		Civic Participation and Engagement	Ischaemic heart diseases	female	OR (95%CI)	9	1		0.98	0.41	2.35
		Social Relationships and Norms	Ischaemic heart diseases	female	OR (95%CI)	10	1		0.95	0.38	2.34
		Civic Participation and Engagement	Ischaemic heart diseases	female	OR (95%CI)	11	1		1.07	0.44	2.63
		Social Relationships and Norms	Ischaemic heart diseases	female	OR (95%CI)	12	1		0.65	0.24	1.75
		Civic Participation and Engagement	Ischaemic heart diseases	female	OR (95%CI)	13	1		1.96	0.39	2.40
		Social Relationships and Norms	Ischaemic heart diseases	female	OR (95%CI)	14	1		1.28	0.56	2.94
		Civic Participation and Engagement	Ischaemic heart diseases	female	OR (95%CI)	15	1		1.04	0.41	2.64
		Social Relationships and Norms	Ischaemic heart diseases	female	OR (95%CI)	16	1		2.72	1.21	6.12
[264]	White, 2011	Discrimination and Segregation	Hypertensive diseases	overall	PR (95%CI)	1	1	high segregation	1.05	0.95	1.17
						1	2	low segregation (reference)	1.00	-	-
		Discrimination and Segregation	Hypertensive diseases	overall	PR (95%CI)	2	1	high segregation	0.90	0.74	1.09
						2	2	low segregation (reference)	1.00	-	-
[265]	White, 2016	Economic and Social Disadvantage	Diabetes mellitus	overall	OR (95%CI)	1	1	Low deprivation (reference)	1.00	-	-
						1	2	Moderate	1.15	1.01	1.31
						1	3	High	1.22	1.07	1.38
[266]	Wight, 2008	Economic and Social Disadvantage	Diseases of the circulatory system	overall	OR (95%CI)	1	1		1.04	0.95	1.14
[267]	Williams, 2023	Social Cohesion and Social Capital	Diabetes mellitus	overall	PR (95%CI)	1	1	High social cohesion (reference)	1.00	-	-
						1	2	Medium social cohesion	1.03	0.99	1.08

						1	3	Low social cohesion	1.22	1.16	1.27
		Social Cohesion and Social Capital	Diabetes mellitus	female	PR (95%CI)	2	1	High social cohesion (reference)	1.00	-	-
						2	2	Medium social cohesion	1.02	0.96	1.08
						2	3	Low social cohesion	1.24	1.17	1.32
		Social Cohesion and Social Capital	Diabetes mellitus	male	PR (95%CI)	3	1	High social cohesion (reference)	1.00	-	-
						3	2	Medium social cohesion	1.04	0.98	1.10
						3	3	Low social cohesion	1.19	1.11	1.27
[268]	Xiao, 2022	Economic and Social Disadvantage	Other forms of heart disease	overall	HR (95%CI)	1	1	consistently high quartile nSES (reference)	1.00	-	-
						1	2	from low to high quartile nSES	1.16	0.99	1.37
						1	3	from high to low quartile nSES	1.19	0.99	1.41
						1	4	consistently low quartile nSES	1.23	1.09	1.40
		Economic and Social Disadvantage	Other forms of heart disease	overall	HR (95%CI)	2	1	consistently high quartile nSES (reference)	1.00	-	-
						2	2	from low to high quartile nSES	1.12	0.93	1.34
						2	3	from high to low quartile nSES	1.16	0.97	1.38
						2	4	consistently low quartile nSES	1.24	1.08	1.42
		Economic and Social Disadvantage	Other forms of heart disease	overall	HR (95%CI)	3	1	consistently high quartile nSES (reference)	1.00	-	-
						3	2	from low to high quartile nSES	1.05	0.86	1.28
						3	3	from high to low quartile nSES	1.14	0.92	1.43
						3	4	consistently low quartile nSES	1.16	0.99	1.37
		Economic and Social Disadvantage	Cerebrovascular diseases	overall	HR (95%CI)	4	1	consistently high quartile nSES (reference)	1.00	-	-
						4	2	from low to high quartile nSES	0.81	0.63	1.05
						4	3	from high to low quartile nSES	0.91	0.69	1.19
						4	4	consistently low quartile nSES	0.94	0.78	1.14
		Economic and Social Disadvantage	Cerebrovascular diseases	overall	HR (95%CI)	5	1	consistently high quartile nSES (reference)	1.00	-	-
						5	2	from low to high quartile nSES	0.98	0.76	1.25
						5	3	from high to low quartile nSES	0.81	0.60	1.08
						5	4	consistently low quartile nSES	0.89	0.71	1.11
		Economic and Social Disadvantage	Cerebrovascular diseases	overall	HR (95%CI)	6	1	consistently high quartile nSES (reference)	1.00	-	-
						6	2	from low to high quartile nSES	0.79	0.59	1.05

						6	3	from high to low quartile nSES	0.96	0.76	1.22	
						6	4	consistently low quartile nSES	0.91	0.70	1.18	
[269]	Xie, 2021	Economic and Social Disadvantage	Hypertensive diseases	overall	linear regression	1	1	beta	1.03	-	-	0.40
[270]	Xu, 2022	Economic and Social Disadvantage	Diseases of the circulatory system	overall	linear regression	1	1	beta	0.07	-	-	0.02
[271]	Xu, 2023	Economic and Social Disadvantage	Hypertensive diseases	female	PR (95%CI)	1	1	Lowest deprivation (<= 10%, reference)	1.00	-	-	
						1	2	11-20%	1.16	1.11	1.21	
						1	3	21-35%	1.19	1.14	1.25	
						1	4	36-55%	1.25	1.20	1.31	
						1	5	Highest deprivation (>55%)	1.26	1.21	1.32	
[272]	Yadav, 2022	Economic and Social Disadvantage	Cerebrovascular diseases	overall	OR (95%CI)	1	1		1.13	0.94	1.36	
		Economic and Social Disadvantage	Cerebrovascular diseases	overall	OR (95%CI)	2	1		1.04	0.94	1.16	
[273]	Yadav, 2021	Social Relationships and Norms	Hypertensive diseases	overall	OR (95%CI)	1	1	good family and social support	1.00	-	-	
						1	2	compromised family and social support	3.00	1.14	6.35	
[274]	Yan, 2022	Social Cohesion and Social Capital	Hypertensive diseases	overall	PR (95%CI)	1	1	neighborhood cohesion tertile 1 (low, reference)	1.00	-	-	
						1	2	neighborhood cohesion tertile 2	1.01	0.90	1.13	
						1	3	neighborhood cohesion tertile 3 (high)	1.00	0.88	1.13	
		Social Relationships and Norms	Hypertensive diseases	overall	PR (95%CI)	2	1	neighborhood violence tertile 1 (high, reference)	1.00	-	-	
						2	2	neighborhood violence tertile 2	0.93	0.83	1.04	
						2	3	neighborhood violence tertile 3 (low)	0.99	0.87	1.13	
[275]	Yan, 2013	Economic and Social Disadvantage	Cerebrovascular diseases	overall	HR (95%CI)	1	1	Q1 (highest neighborhood socioeconomic status, reference)	1.00	-	-	
						1	2	Q2	1.22	0.96	1.54	
						1	3	Q3	1.24	0.96	1.61	
						1	4	Q4 (lowest neighborhood socioeconomic status)	1.29	0.99	1.68	
		Economic and Social Disadvantage	Cerebrovascular diseases	overall	HR (95%CI)	2	1	Q1 (highest neighborhood socioeconomic status, reference)	1.00	-	-	
						2	2	Q2	0.65	0.38	1.11	
						2	3	Q3	0.67	0.40	1.10	

						2	4	Q4 (lowest neighborhood socioeconomic status)	0.57	0.31	1.04
[276]	Yang, 2013	Social Relationships and Norms	Diabetes mellitus	overall	%	1	1	low social integration	0.47	not reported	not reported
						1	2	high social integration	0.43	not reported	not reported
		Social Relationships and Norms	Hypertensive diseases	overall	%	2	1	low social integration	0.14	not reported	not reported
						2	2	high social integration	0.13	not reported	not reported
[277]	Yang, 2015	Social Relationships and Norms	Hypertensive diseases	overall	OR (95%CI)	1	1	Low	1.75	1.04	2.95
						1	2	Moderate	1.23	0.79	1.91
						1	3	Most integrated (reference)	1.00	-	-
		Social Relationships and Norms	Hypertensive diseases	overall	OR (95%CI)	2	1	Low	1.46	0.79	2.68
						2	2	Moderate	1.34	0.95	1.87
						2	3	Most integrated (reference)	1.00	-	-
[278]	Yang, 2016	Social Relationships and Norms	Hypertensive diseases	overall	OR (95%CI)	1	1		1.16	0.83	1.63
		Social Relationships and Norms	Hypertensive diseases	overall	OR (95%CI)	2	1		1.69	0.73	3.93
		Social Relationships and Norms	Hypertensive diseases	overall	OR (95%CI)	3	1		0.89	0.80	0.99
		Social Relationships and Norms	Hypertensive diseases	overall	OR (95%CI)	4	1		0.41	0.22	0.76
[279]	Yazawa, 2016	Civic Participation and Engagement	Hypertensive diseases	overall	Poisson regression coefficient (95%CI)	1	1		0.98	0.92	1.04
		Civic Participation and Engagement	Hypertensive diseases	overall	Poisson regression coefficient (95%CI)	2	1		0.95	0.90	1.00
[280]	Young, 2018	Economic and Social Disadvantage	Hypertensive diseases	overall	PR (95%CI)	1	1	Highest neighborhood education level	45.50	45.20	45.70
						1	2	High	46.80	46.60	47.10
						1	3	Low	46.90	46.60	47.10
						1	4	Lowest education level	45.00	44.70	45.20
		Economic and Social Disadvantage	Hypertensive diseases	overall	PR (95%CI)	2	1	Highest neighborhood education level	36.60	35.40	37.50
						2	2	High	37.60	36.60	38.70
						2	3	Low	38.60	37.40	39.60

						2	4	Lowest education level	36.30	35.10	37.40	
		Economic and Social Disadvantage	Hypertensive diseases	overall	PR (95%CI)	3	1	Highest neighborhood education level	38.60	38.50	38.80	
						3	2	High	41.50	41.30	41.80	
						3	3	Low	42.00	41.80	42.20	
						3	4	Lowest education level	39.60	39.30	39.80	
		Economic and Social Disadvantage	Hypertensive diseases	overall	PR (95%CI)	4	1	Highest neighborhood education level	34.60	34.30	34.90	
						4	2	High	35.80	35.50	36.00	
						4	3	Low	35.40	35.20	35.60	
						4	4	Lowest education level	33.10	32.00	33.20	
		Economic and Social Disadvantage	Hypertensive diseases	overall	PR (95%CI)	5	1	Highest neighborhood education level	44.60	43.60	45.40	
						5	2	High	46.10	45.20	46.80	
						5	3	Low	45.60	45.00	46.20	
						5	4	Lowest education level	42.90	42.20	43.50	
		Economic and Social Disadvantage	Hypertensive diseases	overall	PR (95%CI)	6	1	Highest neighborhood education level	33.70	33.60	33.90	
						6	2	High	35.50	35.40	35.60	
						6	3	Low	36.10	35.90	36.20	
						6	4	Lowest education level	34.70	34.60	34.90	
[281]	Yu, 2024	Disorder and Incivilities	Diabetes mellitus	overall	OR (95%CI)	1	1	neighborhood disorder score	1.13	-	-	0.03

Reference list

1. Abba, M.S., et al., *Influence of contextual socioeconomic position on hypertension risk in low- and middle-income countries: disentangling context from composition.* BMC Public Health, 2021. **21**(1): p. 2218.
2. Adams, R.J., et al., *Effects of area deprivation on health risks and outcomes: a multilevel, cross-sectional, Australian population study.* Int J Public Health, 2009. **54**(3): p. 183-92.
3. Agabiti, N., et al., *Income level and chronic ambulatory care sensitive conditions in adults: a multicity population-based study in Italy.* BMC Public Health, 2009. **9**: p. 457.
4. Agyemang, C., et al., *Ethnic differences in the effect of environmental stressors on blood pressure and hypertension in the Netherlands.* BMC Public Health, 2007. **7**: p. 118.
5. Ahern, M.M. and M.S. Hendryx, *Social capital and risk for chronic illnesses.* Chronic Illn, 2005. **1**(3): p. 183-90.
6. Akwo, E.A., et al., *Neighborhood Deprivation Predicts Heart Failure Risk in a Low-Income Population of Blacks and Whites in the Southeastern United States.* Circ Cardiovasc Qual Outcomes, 2018. **11**(1): p. e004052.
7. Alemi, F., et al., *Social and Medical Determinants of Diabetes: A Time-Constrained Multiple Mediator Analysis.* Cureus, 2023. **15**(9): p. e46227.
8. Allan, K.S., et al., *High risk neighbourhoods: The effect of neighbourhood level factors on cardiac arrest incidence.* Resuscitation, 2020. **149**: p. 100-108.
9. Altevers, J., et al., *Poor structural social support is associated with an increased risk of Type 2 diabetes mellitus: findings from the MONICA/KORA Augsburg cohort study.* Diabet Med, 2016. **33**(1): p. 47-54.
10. Andersen, A.F., et al., *Life-course socio-economic position, area deprivation and Type 2 diabetes: findings from the British Women's Heart and Health Study.* Diabet Med, 2008. **25**(12): p. 1462-8.
11. Anderson, K.F., E. Bjorklund, and S. Rambotti, *Income Inequality and Chronic Health Conditions: A Multilevel Analysis of the U.S. States.* Sociol. Focus, 2019. **52**(1): p. 65-85.
12. Atasoy, S., et al., *The Association of Social Connectivity and Body Weight With the Onset of Type 2 Diabetes: Findings From the Population-Based Prospective MONICA/KORA Cohort.* Psychosom Med, 2022. **84**(9): p. 1050-1055.
13. Augustin, T., T.A. Glass, B.D. James, and B.S. Schwartz, *Neighborhood psychosocial hazards and cardiovascular disease: the Baltimore Memory Study.* Am J Public Health, 2008. **98**(9): p. 1664-70.
14. Avogo, W.A., *Community characteristics and the risk of non-communicable diseases in Ghana.* PLOS Glob Public Health, 2023. **3**(1): p. e0000692.
15. Baek, J., N.W. Hur, H.C. Kim, and Y. Youm, *Sex-specific effects of social networks on the prevalence, awareness, and control of hypertension among older Korean adults.* J Geriatr Cardiol, 2016. **13**(7): p. 580-6.
16. Banchani, E., E.Y. Tenkorang, and W. Midodzi, *Examining the effects of individual and neighbourhood socioeconomic status/wealth on hypertension among women in the Greater Accra Region of Ghana.* Health Soc Care Community, 2020.
17. Bancks, M.P., et al., *Association of Modifiable Risk Factors in Young Adulthood With Racial Disparity in Incident Type 2 Diabetes During Middle Adulthood.* Jama, 2017. **318**(24): p. 2457-2465.
18. Barber, S., et al., *Neighborhood Disadvantage, Poor Social Conditions, and Cardiovascular Disease Incidence Among African American Adults in the Jackson Heart Study.* Am J Public Health, 2016. **106**(12): p. 2219-2226.
19. Barber, S., et al., *At the intersection of place, race, and health in Brazil: Residential segregation and cardio-metabolic risk factors in the Brazilian Longitudinal Study of Adult Health (ELSA-Brasil).* Soc Sci Med, 2018. **199**: p. 67-76.

20. Barefoot, J.C., et al., *Social network diversity and risks of ischemic heart disease and total mortality: findings from the Copenhagen City Heart Study.* Am J Epidemiol, 2005. **161**(10): p. 960-7.
21. Basile Ibrahim, B., et al., *The Association Between Neighborhood Social Vulnerability and Cardiovascular Health Risk Among Black/African American Women in the InterGEN Study.* Nurs Res, 2021. **70**(5): p. S3-s12.
22. Bevan, G., et al., *Neighborhood-level Social Vulnerability and Prevalence of Cardiovascular Risk Factors and Coronary Heart Disease.* Curr Probl Cardiol, 2023. **48**(8): p. 101182.
23. Bhavsar, N.A., et al., *Association between Gentrification and Health and Healthcare Utilization.* J Urban Health, 2022. **99**(6): p. 984-997.
24. Bhopal, R., et al., *Ethnic and socio-economic inequalities in coronary heart disease, diabetes and risk factors in Europeans and South Asians.* J Public Health Med, 2002. **24**(2): p. 95-105.
25. Bilal, U., et al., *Association of neighbourhood socioeconomic status and diabetes burden using electronic health records in Madrid (Spain): the HeartHealthyHoods study.* BMJ Open, 2018. **8**(9): p. e021143.
26. Bocour, A. and M. Tria, *Preventable Hospitalization Rates and Neighborhood Poverty among New York City Residents, 2008-2013.* J Urban Health, 2016. **93**(6): p. 974-983.
27. Borges, C.M., et al., *Social capital or vulnerability: Which has the stronger connection with selected U.S. health outcomes?* SSM Popul Health, 2021. **15**: p. 100812.
28. Boruzs, K., et al., *High Inequalities Associated With Socioeconomic Deprivation in Cardiovascular Disease Burden and Antihypertensive Medication in Hungary.* Front Pharmacol, 2018. **9**: p. 839.
29. Bravo, M.A., R. Anthopolos, R.T. Kimbro, and M.L. Miranda, *Residential Racial Isolation and Spatial Patterning of Type 2 Diabetes Mellitus in Durham, North Carolina.* Am J Epidemiol, 2018. **187**(7): p. 1467-1476.
30. Bravo, M.A., B.C. Batch, and M.L. Miranda, *Residential Racial Isolation and Spatial Patterning of Hypertension in Durham, North Carolina.* Prev Chronic Dis, 2019. **16**: p. E36.
31. Bray, B.D., et al., *Socioeconomic disparities in first stroke incidence, quality of care, and survival: a nationwide registry-based cohort study of 44 million adults in England.* Lancet Public Health, 2018. **3**(4): p. e185-e193.
32. Brinkhues, S., et al., *Socially isolated individuals are more prone to have newly diagnosed and prevalent type 2 diabetes mellitus - the Maastricht study.* BMC Public Health, 2017. **17**(1): p. 955.
33. Bush, K.J., et al., *Influence of neighborhood-level socioeconomic deprivation and individual socioeconomic position on risk of developing type 2 diabetes in older men: a longitudinal analysis in the British Regional Heart Study cohort.* BMJ Open Diabetes Res Care, 2023. **11**(5).
34. Butler, D.C., S. Petterson, A. Bazemore, and K.A. Douglas, *Use of measures of socioeconomic deprivation in planning primary health care workforce and defining health care need in Australia.* Aust J Rural Health, 2010. **18**(5): p. 199-204.
35. Buys, D.R., et al., *Association between neighborhood disadvantage and hypertension prevalence, awareness, treatment, and control in older adults: results from the University of Alabama at Birmingham Study of Aging.* Am J Public Health, 2015. **105**(6): p. 1181-8.
36. Carlsson, A.C., et al., *Neighbourhood socioeconomic status and coronary heart disease in individuals between 40 and 50 years.* Heart, 2016. **102**(10): p. 775-82.
37. Carlsson, A.C., et al., *Neighborhood socioeconomic status at the age of 40 years and ischemic stroke before the age of 50 years: A nationwide cohort study from Sweden.* Int J Stroke, 2017. **12**(8): p. 815-826.
38. Cebrecos, A., et al., *Geographic and statistic stability of deprivation aggregated measures at different spatial units in health research.* Appl. Geogr., 2018. **95**: p. 9-18.
39. Cené, C.W., et al., *Social Isolation and Incident Heart Failure Hospitalization in Older Women: Women's Health Initiative Study Findings.* J Am Heart Assoc, 2022. **11**(5): p. e022907.
40. Chaix, B., et al., *Neighborhood effects on health: correcting bias from neighborhood effects on participation.* Epidemiology, 2011. **22**(1): p. 18-26.

41. Chamberlain, A.M., et al., *Associations of Neighborhood Socioeconomic Disadvantage With Chronic Conditions by Age, Sex, Race, and Ethnicity in a Population-Based Cohort.* Mayo Clin Proc, 2022. **97**(1): p. 57-67.
42. Chan, J.J.L., et al., *Inequalities in the prevalence of cardiovascular disease risk factors in Brazilian slum populations: A cross-sectional study.* PLOS Glob Public Health, 2022. **2**(9): p. e0000990.
43. Chang, S.C., et al., *Social Integration and Reduced Risk of Coronary Heart Disease in Women: The Role of Lifestyle Behaviors.* Circ Res, 2017. **120**(12): p. 1927-1937.
44. Chatzi, G., et al., *Sociodemographic disparities in non-diabetic hyperglycaemia and the transition to type 2 diabetes: evidence from the English Longitudinal Study of Ageing.* Diabet Med, 2020. **37**(9): p. 1536-1544.
45. Cheruvalath, H., et al., *Associations Between Residential Greenspace, Socioeconomic Status, and Stroke: A Matched Case-Control Study.* J Patient Cent Res Rev, 2022. **9**(2): p. 89-97.
46. Child, S.T., E.H. Ruppel, M.A. Albert, and L. Lawton, *Network Support and Negative Life Events Associated With Chronic Cardiometabolic Disease Outcomes.* Am J Prev Med, 2022. **62**(1): p. e21-e28.
47. Christine, P.J., et al., *Longitudinal Associations Between Neighborhood Physical and Social Environments and Incident Type 2 Diabetes Mellitus: The Multi-Ethnic Study of Atherosclerosis (MESA).* JAMA Intern Med, 2015. **175**(8): p. 1311-20.
48. Chum, A. and P. O'Campo, *Cross-sectional associations between residential environmental exposures and cardiovascular diseases.* BMC Public Health, 2015. **15**: p. 438.
49. Clark, C.J., et al., *Neighborhood cohesion is associated with reduced risk of stroke mortality.* Stroke, 2011. **42**(5): p. 1212-7.
50. Claudel, S.E., et al., *Association between neighborhood-level socioeconomic deprivation and incident hypertension: A longitudinal analysis of data from the Dallas heart study.* Am Heart J, 2018. **204**: p. 109-118.
51. Coelho, D.M., et al., *Gender differences in the association of individual and contextual socioeconomic status with hypertension in 230 Latin American cities from the SALURBAL study: a multilevel analysis.* BMC Public Health, 2023. **23**(1): p. 1532.
52. Cofie, L.E., J.M. Hirth, and J.G.L. Lee, *Social Support Networks and Foreign-Birth Status Associated With Obesity, Hypertension and Diabetes Prevalence Among 21-30 and 50-70 Year Old Adults Living in the San Francisco Bay Area.* Am J Health Promot, 2021. **35**(8): p. 1105-1113.
53. Connolly, V., et al., *Diabetes prevalence and socioeconomic status: a population based study showing increased prevalence of type 2 diabetes mellitus in deprived areas.* Journal of Epidemiology and Community Health, 2000. **54**(3): p. 173-177.
54. Consolazio, D., et al., *Neighbourhood property value and type 2 diabetes mellitus in the Maastricht study: A multilevel study.* PLoS One, 2020. **15**(6): p. e0234324.
55. Cookson, R., M. Laudicella, and P.L. Donni, *Measuring change in health care equity using small-area administrative data - evidence from the English NHS 2001-2008.* Soc Sci Med, 2012. **75**(8): p. 1514-22.
56. Cox, M., et al., *Locality deprivation and Type 2 diabetes incidence: a local test of relative inequalities.* Soc Sci Med, 2007. **65**(9): p. 1953-64.
57. Cozier, Y.C., et al., *Relation between neighborhood median housing value and hypertension risk among black women in the United States.* Am J Public Health, 2007. **97**(4): p. 718-24.
58. Cromer, S.J., et al., *Association and Interaction of Genetics and Area-Level Socioeconomic Factors on the Prevalence of Type 2 Diabetes and Obesity.* Diabetes Care, 2023. **46**(5): p. 944-952.
59. Cubbin, C., et al., *Neighborhood deprivation and cardiovascular disease risk factors: protective and harmful effects.* Scand J Public Health, 2006. **34**(3): p. 228-37.
60. Cunningham, S.A., et al., *County-level contextual factors associated with diabetes incidence in the United States.* Ann Epidemiol, 2018. **28**(1): p. 20-25.e2.
61. Cuthbertson, C.C., et al., *Socioeconomic status and access to care and the incidence of a heart failure diagnosis in the inpatient and outpatient settings.* Ann Epidemiol, 2018. **28**(6): p. 350-355.
62. de Oliveira, F.L.P., et al., *Spatial clusters of diabetes: individual and neighborhood characteristics in the ELSA-Brasil cohort study.* CADERNOS DE SAUDE PUBLICA, 2023. **39**(5).

63. De Silva, S.S.A., et al., *Comparing Global and Spatial Composite Measures of Neighborhood Socioeconomic Status Across US Counties.* J Urban Health, 2022. **99**(3): p. 457-468.
64. Desmond, M. and W.H. An, *Neighborhood and Network Disadvantage among Urban Renters.* Sociol. Sci., 2015. **2**: p. 329-349.
65. Diez Roux, A.V., et al., *Socioeconomic disadvantage and change in blood pressure associated with aging.* Circulation, 2002. **106**(6): p. 703-10.
66. Diez-Roux, A.V., et al., *Neighborhood environments and coronary heart disease: a multilevel analysis.* Am J Epidemiol, 1997. **146**(1): p. 48-63.
67. Diez-Roux, A.V., B.G. Link, and M.E. Northridge, *A multilevel analysis of income inequality and cardiovascular disease risk factors.* Soc. Sci. Med., 2000. **50**(5): p. 673-687.
68. Djekic, D., et al., *Impact of socioeconomic status on coronary artery calcification.* Eur J Prev Cardiol, 2018. **25**(16): p. 1756-1764.
69. Dragano, N., et al., *Neighbourhood socioeconomic status and cardiovascular risk factors: a multilevel analysis of nine cities in the Czech Republic and Germany.* BMC Public Health, 2007. **7**: p. 255.
70. Dubowitz, T., et al., *The Women's Health Initiative: The food environment, neighborhood socioeconomic status, BMI, and blood pressure.* Obesity (Silver Spring), 2012. **20**(4): p. 862-71.
71. Dwane, N., N. Wabiri, and S. Manda, *Small-area variation of cardiovascular diseases and select risk factors and their association to household and area poverty in South Africa: Capturing emerging trends in South Africa to better target local level interventions.* PLoS One, 2020. **15**(4): p. e0230564.
72. Dyck, J., R. Tate, J. Uhanova, and M. Torabi, *Social determinants and spatio-temporal variation of Ischemic Heart Disease in Manitoba.* BMC Public Health, 2021. **21**(1): p. 2325.
73. Ekholuenetale, M. and A. Barrow, *Prevalence and determinants of self-reported high blood pressure among women of reproductive age in Benin: a population-based study.* Clin Hypertens, 2020. **26**: p. 12.
74. Eng, P.M., E.B. Rimm, G. Fitzmaurice, and I. Kawachi, *Social ties and change in social ties in relation to subsequent total and cause-specific mortality and coronary heart disease incidence in men.* Am J Epidemiol, 2002. **155**(8): p. 700-9.
75. Engström, G., et al., *Geographic distribution of stroke incidence within an urban population: Relations to socioeconomic circumstances and prevalence of cardiovascular risk factors.* Stroke, 2001. **32**(5): p. 1098-1103.
76. Eschbach, K., et al., *Neighborhood context and mortality among older Mexican Americans: is there a barrio advantage?* Am J Public Health, 2004. **94**(10): p. 1807-12.
77. Essien, U.R., et al., *Association Between Neighborhood-Level Poverty and Incident Atrial Fibrillation: a Retrospective Cohort Study.* J Gen Intern Med, 2022. **37**(6): p. 1436-1443.
78. Exeter, D.J., et al., *Movers and stayers: The geography of residential mobility and CVD hospitalisations in Auckland, New Zealand.* Soc Sci Med, 2015. **133**: p. 331-9.
79. Faka, A., et al., *Association of Socio-Environmental Determinants with Diabetes Prevalence in the Athens Metropolitan Area, Greece: A Spatial Analysis.* Rev Diabet Stud, 2018. **14**(4): p. 381-389.
80. Feero, S., J.R. Hedges, and P. Stevens, *Demographics of cardiac arrest: association with residence in a low-income area.* Acad Emerg Med, 1995. **2**(1): p. 11-6.
81. Ferguson, T.S., et al., *Neighbourhood socioeconomic characteristics and blood pressure among Jamaican youth: a pooled analysis of data from observational studies.* PeerJ, 2020. **8**: p. e10058.
82. Fitzpatrick, K.M. and D. Willis, *Chronic Disease, the Built Environment, and Unequal Health Risks in the 500 Largest U.S. Cities.* Int J Environ Res Public Health, 2020. **17**(8).
83. Fleischer, N.L., A.V. Diez Roux, M. Alazraqui, and H. Spinelli, *Social patterning of chronic disease risk factors in a Latin American city.* J Urban Health, 2008. **85**(6): p. 923-37.
84. Ford, E.S., E.B. Loucks, and L.F. Berkman, *Social integration and concentrations of C-reactive protein among US adults.* Ann Epidemiol, 2006. **16**(2): p. 78-84.
85. Forsberg, P.O., H. Ohlsson, and K. Sundquist, *Causal nature of neighborhood deprivation on individual risk of coronary heart disease or ischemic stroke: A prospective national Swedish co-relative control study in men and women.* Health Place, 2018. **50**: p. 1-5.

86. Forsberg, P.O., H. Ohlsson, and K. Sundquist, *Workplace socioeconomic characteristics and coronary heart disease: a nationwide follow-up study.* BMJ Open, 2023. **13**(7): p. e065285.
87. Freedman, V.A., I.B. Grafova, and J. Rogowski, *Neighborhoods and chronic disease onset in later life.* Am J Public Health, 2011. **101**(1): p. 79-86.
88. Gao, X., et al., *Associations Between Residential Segregation and Incident Hypertension: The Multi-Ethnic Study of Atherosclerosis.* J Am Heart Assoc, 2022. **11**(3): p. e023084.
89. Garcia, L., et al., *The Impact of Neighborhood Socioeconomic Position on Prevalence of Diabetes and Prediabetes in Older Latinos: The Sacramento Area Latino Study on Aging.* Hisp Health Care Int, 2015. **13**(2): p. 77-85.
90. Garcia, L., et al., *Influence of neighbourhood socioeconomic position on the transition to type II diabetes in older Mexican Americans: the Sacramento Area Longitudinal Study on Aging.* BMJ Open, 2016. **6**(8): p. e010905.
91. Gary-Webb, T.L., et al., *Community stressors (violence, victimization, and neighborhood disorder) with cardiometabolic outcomes in urban Jamaica.* Front Public Health, 2023. **11**: p. 1130830.
92. Gaskin, D.J., et al., *Disparities in diabetes: the nexus of race, poverty, and place.* Am J Public Health, 2014. **104**(11): p. 2147-55.
93. Gebreab, S.Y., et al., *Neighborhood social and physical environments and type 2 diabetes mellitus in African Americans: The Jackson Heart Study.* Health Place, 2017. **43**: p. 128-137.
94. Gero, K., et al., *Associations of state-level and county-level hate crimes with individual-level cardiovascular risk factors in a prospective cohort study of middle-aged Americans: the National Longitudinal Survey of Youths 1979.* BMJ Open, 2022. **12**(1): p. e054360.
95. Glover, L.M., et al., *Sex differences in the association of psychosocial resources with prevalent type 2 diabetes among African Americans: The Jackson Heart Study.* J Diabetes Complications, 2019. **33**(2): p. 113-117.
96. Grundmann, N., A. Mielck, M. Siegel, and W. Maier, *Area deprivation and the prevalence of type 2 diabetes and obesity: analysis at the municipality level in Germany.* BMC Public Health, 2014. **14**: p. 1264.
97. Guion, M., et al., *Eleven-year trends in socioeconomic inequalities in the prevalence and incidence of pharmacologically treated type 2 diabetes in France, 2010-2020.* Diabetes Metab, 2024. **50**(2): p. 101509.
98. Gwon, J.G., J. Choi, and Y.J. Han, *Community-level socioeconomic inequality in the incidence of ischemic heart disease: a nationwide cohort study.* BMC Cardiovasc Disord, 2020. **20**(1): p. 87.
99. Halonen, J.I., et al., *Childhood Psychosocial Adversity and Adult Neighborhood Disadvantage as Predictors of Cardiovascular Disease: A Cohort Study.* Circulation, 2015. **132**(5): p. 371-9.
100. Hamad, R., et al., *Association of Neighborhood Disadvantage With Cardiovascular Risk Factors and Events Among Refugees in Denmark.* JAMA Netw Open, 2020. **3**(8): p. e2014196.
101. Hanefeld, C., et al., *Social Gradients in Myocardial Infarction and Stroke Diagnoses in Emergency Medicine.* Dtsch Arztebl Int, 2018. **115**(4): p. 41-48.
102. Hanigan, I.C., T. Cochrane, and R. Davey, *Impact of scale of aggregation on associations of cardiovascular hospitalization and socio-economic disadvantage.* PLoS One, 2017. **12**(11): p. e0188161.
103. Harding, B.N., et al., *Relationship between social support and incident hypertension in the Jackson Heart Study: a cohort study.* BMJ Open, 2022. **12**(3): p. e054812.
104. Hashemi, S.J., et al., *Social determinants of health and diabetes: Results from a cohort study in Iran.* ASIAN JOURNAL OF SOCIAL HEALTH AND BEHAVIOR, 2023. **6**(2): p. 86-91.
105. Hassen, H.Y., H. Bastiaens, K. Van Royen, and S. Abrams, *Socioeconomic and behavioral determinants of cardiovascular diseases among older adults in Belgium and France: A longitudinal analysis from the SHARE study.* PLoS One, 2020. **15**(12): p. e0243422.
106. Hawkins, N.M., et al., *Community care in England: reducing socioeconomic inequalities in heart failure.* Circulation, 2012. **126**(9): p. 1050-7.

107. Heeley, E.L., et al., *Socioeconomic disparities in stroke rates and outcome: pooled analysis of stroke incidence studies in Australia and New Zealand.* Med J Aust, 2011. **195**(1): p. 10-4.
108. Hendryx, M., et al., *Social Relationships and Risk of Type 2 Diabetes Among Postmenopausal Women.* J Gerontol B Psychol Sci Soc Sci, 2020. **75**(7): p. 1597-1608.
109. Henriksson, G., G.R. Weitoft, and P. Allebeck, *Associations between income inequality at municipality level and health depend on context - a multilevel analysis on myocardial infarction in Sweden.* Soc Sci Med, 2010. **71**(6): p. 1141-9.
110. Heredia, N.I., et al., *The Neighborhood Environment and Hispanic/Latino Health.* Am J Health Promot, 2022. **36**(1): p. 38-45.
111. Herrera-Añazco, P., et al., *Association between social determinants of health and trends in prevalence of hypertension in patients of the Peruvian Ministry of Health.* Trop Med Int Health, 2019. **24**(12): p. 1434-1441.
112. Herrick, C.J., B.W. Yount, and A.A. Eyler, *Implications of supermarket access, neighbourhood walkability and poverty rates for diabetes risk in an employee population.* Public Health Nutr, 2016. **19**(11): p. 2040-8.
113. Hilding, A., C. Shen, and C.G. Östenson, *Social network and development of prediabetes and type 2 diabetes in middle-aged Swedish women and men.* Diabetes Res Clin Pract, 2015. **107**(1): p. 166-77.
114. Hill, P.L., S.J. Weston, and J.J. Jackson, *Connecting social environment variables to the onset of major specific health outcomes.* Psychol Health, 2014. **29**(7): p. 753-67.
115. Höfelmann, D.A., J.L. Antunes, D.A. Santos Silva, and M.A. Peres, *Is income area level associated with blood pressure in adults regardless of individual-level characteristics? A multilevel approach.* Health Place, 2012. **18**(5): p. 971-7.
116. Holstiege, J., et al., *Higher prevalence of heart failure in rural regions: a population-based study covering 87% of German inhabitants.* Clin Res Cardiol, 2019. **108**(10): p. 1102-1106.
117. Holtgrave, D.R. and R. Crosby, *Is social capital a protective factor against obesity and diabetes? Findings from an exploratory study.* Ann Epidemiol, 2006. **16**(5): p. 406-8.
118. Honda, Y., et al., *Psychosocial factors and subsequent risk of hospitalizations with peripheral artery disease: The Atherosclerosis Risk in Communities (ARIC) Study.* Atherosclerosis, 2021. **329**: p. 36-43.
119. Honjo, K., et al., *Impact of neighborhood socioeconomic conditions on the risk of stroke in Japan.* J Epidemiol, 2015. **25**(3): p. 254-60.
120. Horsten, M., et al., *Social relations and the metabolic syndrome in middle-aged Swedish women.* J Cardiovasc Risk, 1999. **6**(6): p. 391-7.
121. Hosseini, Z., G. Veenstra, N.A. Khan, and A.I. Conklin, *Social connections and hypertension in women and men: a population-based cross-sectional study of the Canadian Longitudinal Study on Aging.* J Hypertens, 2020.
122. Howard, V.J., et al., *Neighborhood socioeconomic index and stroke incidence in a national cohort of blacks and whites.* Neurology, 2016. **87**(22): p. 2340-2347.
123. Hu, L., et al., *Quantile Regression Forests to Identify Determinants of Neighborhood Stroke Prevalence in 500 Cities in the USA: Implications for Neighborhoods with High Prevalence.* J Urban Health, 2020.
124. Hu, M.D., et al., *Neighborhood Deprivation, Obesity, and Diabetes in Residents of the US Gulf Coast.* Am J Epidemiol, 2021. **190**(2): p. 295-304.
125. Huang, H., *Moderating Effects of Racial Segregation on the Associations of Cardiovascular Outcomes with Walkability in Chicago Metropolitan Area.* Int J Environ Res Public Health, 2022. **19**(21).
126. Hwang, S.E., et al., *Association between social trust and the risk of cardiovascular disease in older adults in Korea: a nationwide retrospective cohort study.* BMC Public Health, 2020. **20**(1): p. 1844.
127. Jack, E., D. Lee, and N. Dean, *Estimating the changing nature of Scotland's health inequalities by using a multivariate spatiotemporal model.* J R Stat Soc Ser A Stat Soc, 2019. **182**(3): p. 1061-1080.
128. Jackson, C.H., S. Richardson, and N.G. Best, *Studying place effects on health by synthesising individual and area-level outcomes.* Soc Sci Med, 2008. **67**(12): p. 1995-2006.

129. Jadow, B.M., et al., *Historical Redlining, Social Determinants of Health, and Stroke Prevalence in Communities in New York City.* JAMA Netw Open, 2023. **6**(4): p. e235875.
130. Jain, V., et al., *Association Between Social Vulnerability Index and Cardiovascular Disease: A Behavioral Risk Factor Surveillance System Study.* J Am Heart Assoc, 2022. **11**(15): p. e024414.
131. Jonsson, M., et al., *Relationship between socioeconomic status and incidence of out-of-hospital cardiac arrest is dependent on age.* J Epidemiol Community Health, 2020. **74**(9): p. 726-731.
132. Jung, L., et al., *Nationally representative household survey data for studying the interaction between district-level development and individual-level socioeconomic gradients of cardiovascular disease risk factors in India.* Data Brief, 2019. **27**: p. 104486.
133. Kaiser, P., et al., *Neighborhood Environments and Incident Hypertension in the Multi-Ethnic Study of Atherosclerosis.* Am J Epidemiol, 2016. **183**(11): p. 988-97.
134. Kakinami, L., et al., *Neighbourhood disadvantage and behavioural problems during childhood and the risk of cardiovascular disease risk factors and events from a prospective cohort.* Prev Med Rep, 2017. **8**: p. 294-300.
135. Kauhl, B., et al., *Exploring the small-scale spatial distribution of hypertension and its association to area deprivation based on health insurance claims in Northeastern Germany.* BMC Public Health, 2018. **18**(1).
136. Kawachi, I., et al., *A prospective study of social networks in relation to total mortality and cardiovascular disease in men in the USA.* J Epidemiol Community Health, 1996. **50**(3): p. 245-51.
137. Keita, A.D., et al., *Associations of neighborhood area level deprivation with the metabolic syndrome and inflammation among middle- and older- age adults.* BMC Public Health, 2014. **14**: p. 1319.
138. Kelli, H.M., et al., *Association Between Living in Food Deserts and Cardiovascular Risk.* Circ Cardiovasc Qual Outcomes, 2017. **10**(9).
139. Kershaw, K.N., et al., *Metropolitan-level racial residential segregation and black-white disparities in hypertension.* Am J Epidemiol, 2011. **174**(5): p. 537-45.
140. Kim, M.H., et al., *School racial segregation and long-term cardiovascular health among Black adults in the US: A quasi-experimental study.* PLoS Med, 2022. **19**(6): p. e1004031.
141. Kim, D., et al., *Neighbourhood socioeconomic position and risks of major chronic diseases and all-cause mortality: a quasi-experimental study.* BMJ Open, 2018. **8**(5): p. e018793.
142. Kim, Y., A. Lee, and C. Cubbin, *Effect of Social Environments on Cardiovascular Disease in the United States.* J Am Heart Assoc, 2022. **11**(20): p. e025923.
143. Kivimäki, M., et al., *Neighbourhood socioeconomic disadvantage, risk factors, and diabetes from childhood to middle age in the Young Finns Study: a cohort study.* Lancet Public Health, 2018. **3**(8): p. e365-e373.
144. Kivimäki, M., et al., *Modifications to residential neighbourhood characteristics and risk of 79 common health conditions: a prospective cohort study.* Lancet Public Health, 2021. **6**(6): p. e396-e407.
145. Kling, J.R., J.B. Liebman, and L.F. Katz, *Experimental analysis of neighborhood effects.* Econometrica, 2007. **75**(1): p. 83-119.
146. Jensen, N.K., et al., *The association of neighborhood socioeconomic characteristics with cardiovascular health: A quasi-experimental study of refugees to Denmark.* Health Place, 2023. **84**: p. 103128.
147. Kolpak, P. and L. Wang, *Exploring the social and neighbourhood predictors of diabetes: a comparison between Toronto and Chicago.* Prim Health Care Res Dev, 2017. **18**(3): p. 291-299.
148. Krieger, N., *Overcoming the absence of socioeconomic data in medical records: validation and application of a census-based methodology.* Am J Public Health, 1992. **82**(5): p. 703-10.
149. Krishnan, S., Y.C. Cozier, L. Rosenberg, and J.R. Palmer, *Socioeconomic status and incidence of type 2 diabetes: results from the Black Women's Health Study.* Am J Epidemiol, 2010. **171**(5): p. 564-70.

150. Kwok, M.K., et al., *Relative Deprivation, Income Inequality, and Cardiovascular Health: Observational and Mendelian Randomization Studies in Hong Kong Chinese.* Front Public Health, 2021. **9**: p. 726617.
151. Lachkhem, Y., É. Minvielle, and S. Rican, *Geographic Variations of Stroke Hospitalization across France: A Diachronic Cluster Analysis.* Stroke Res Treat, 2018. **2018**: p. 1897569.
152. Lagisetty, P.A., et al., *Neighborhood Social Cohesion and Prevalence of Hypertension and Diabetes in a South Asian Population.* J Immigr Minor Health, 2016. **18**(6): p. 1309-1316.
153. Larrañaga, I., et al., *Socio-economic inequalities in the prevalence of Type 2 diabetes, cardiovascular risk factors and chronic diabetic complications in the Basque Country, Spain.* Diabet Med, 2005. **22**(8): p. 1047-53.
154. Laursen, K.R., A. Hulman, D.R. Witte, and H. Terkildsen Maindal, *Social relations, depressive symptoms, and incident type 2 diabetes mellitus: The English Longitudinal Study of Ageing.* Diabetes Res Clin Pract, 2017. **126**: p. 86-94.
155. Lawlor, D.A., G. Davey Smith, R. Patel, and S. Ebrahim, *Life-course socioeconomic position, area deprivation, and coronary heart disease: findings from the British Women's Heart and Health Study.* Am J Public Health, 2005. **95**(1): p. 91-7.
156. Lee, J., et al., *Geographic Variation in Morbidity and Mortality of Cerebrovascular Diseases in Korea during 2011-2015.* J Stroke Cerebrovasc Dis, 2018. **27**(3): p. 747-757.
157. Lee, D.C., et al., *Identifying Geographic Disparities in Diabetes Prevalence Among Adults and Children Using Emergency Claims Data.* J Endocr Soc, 2018. **2**(5): p. 460-470.
158. Lemstra, M., C. Neudorf, and J. Opondo, *Health disparity by neighbourhood income.* Can J Public Health, 2006. **97**(6): p. 435-9.
159. Leyland, A.H., *Socioeconomic gradients in the prevalence of cardiovascular disease in Scotland: the roles of composition and context.* J Epidemiol Community Health, 2005. **59**(9): p. 799-803.
160. Li, K., M. Wen, and K.A. Henry, *Ethnic density, immigrant enclaves, and Latino health risks: A propensity score matching approach.* Soc Sci Med, 2017. **189**: p. 44-52.
161. Linde, S. and L.E. Egede, *Community Social Capital and Population Health Outcomes.* JAMA Netw Open, 2023. **6**(8): p. e2331087.
162. Ling, D.C., *Do the Chinese "Keep up with the Jones"?: Implications of peer effects, growing economic disparities and relative deprivation on health outcomes among older adults in China.* China Econ. Rev., 2009. **20**(1): p. 65-81.
163. Lippert, A.M., C.R. Evans, F. Razak, and S.V. Subramanian, *Associations of Continuity and Change in Early Neighborhood Poverty With Adult Cardiometabolic Biomarkers in the United States: Results From the National Longitudinal Study of Adolescent to Adult Health, 1995-2008.* Am J Epidemiol, 2017. **185**(9): p. 765-776.
164. Lönn, S.L., O. Melander, C. Crump, and K. Sundquist, *Accumulated neighbourhood deprivation and coronary heart disease: a nationwide cohort study from Sweden.* BMJ Open, 2019. **9**(9): p. e029248.
165. Loucks, E.B., et al., *Social networks and inflammatory markers in the Framingham Heart Study.* J Biosoc Sci, 2006. **38**(6): p. 835-42.
166. Lu, X., et al., *The Association Between Perceived Stress and Hypertension Among Asian Americans: Does Social Support and Social Network Make a Difference?* J Community Health, 2019. **44**(3): p. 451-462.
167. Ludwig, J., et al., *Neighborhoods, obesity, and diabetes--a randomized social experiment.* N Engl J Med, 2011. **365**(16): p. 1509-19.
168. Lukachko, A., M.L. Hatzenbuehler, and K.M. Keyes, *Structural racism and myocardial infarction in the United States.* Soc Sci Med, 2014. **103**: p. 42-50.
169. Lukaschek, K., et al., *Sex differences in the association of social network satisfaction and the risk for type 2 diabetes.* BMC Public Health, 2017. **17**(1): p. 379.
170. Lund, R., N.H. Rod, and U. Christensen, *Are negative aspects of social relations predictive of angina pectoris? A 6-year follow-up study of middle-aged Danish women and men.* J Epidemiol Community Health, 2012. **66**(4): p. 359-65.
171. Lund, R., et al., *Negative aspects of close social relations and 10-year incident ischaemic heart disease hospitalization among middle-aged Danes.* Eur J Prev Cardiol, 2014. **21**(10): p. 1249-56.

172. Ma, R., et al., *Physical Multimorbidity and Social Participation in Adult Aged 65 Years and Older From Six Low- and Middle-Income Countries.* J Gerontol B Psychol Sci Soc Sci, 2021. **76**(7): p. 1452-1462.
173. Madela, S., et al., *Individual and area-level socioeconomic correlates of hypertension prevalence, awareness, treatment, and control in uMgungundlovu, KwaZulu-Natal, South Africa.* BMC Public Health, 2023. **23**(1): p. 417.
174. Madela, S.L.M., et al., *Area-level deprivation and individual-level socioeconomic correlates of the diabetes care cascade among black south africans in uMgungundlovu, KwaZulu-Natal, South Africa.* PLoS One, 2023. **18**(12): p. e0293250.
175. Maheswaran, R., et al., *Socio-economic deprivation and excess winter mortality and emergency hospital admissions in the South Yorkshire Coalfields Health Action Zone, UK.* Public Health, 2004. **118**(3): p. 167-76.
176. Maier, W., et al., *Area Level Deprivation Is an Independent Determinant of Prevalent Type 2 Diabetes and Obesity at the National Level in Germany. Results from the National Telephone Health Interview Surveys 'German Health Update' GEDA 2009 and 2010.* Plos One, 2014. **9**(2).
177. Malino, C., et al., *Social capital and hypertension in rural Haitian women.* Matern Child Health J, 2014. **18**(10): p. 2253-60.
178. Marley, T.L. and M.W. Metzger, *A longitudinal study of structural risk factors for obesity and diabetes among American Indian young adults, 1994-2008.* Prev Chronic Dis, 2015. **12**: p. E69.
179. Massa, K.H., R. Pabayo, M.L. Lebrão, and A.D. Chiavegatto Filho, *Environmental factors and cardiovascular diseases: the association of income inequality and green spaces in elderly residents of São Paulo, Brazil.* BMJ Open, 2016. **6**(9): p. e011850.
180. Matheson, F.I., et al., *Neighbourhood chronic stress and gender inequalities in hypertension among Canadian adults: a multilevel analysis.* J Epidemiol Community Health, 2010. **64**(8): p. 705-13.
181. Matthew, P. and D.M. Brodersen, *Income inequality and health outcomes in the United States: An empirical analysis.* Soc. Sci. J., 2018. **55**(4): p. 432-442.
182. Mayne, S.L., et al., *Racial residential segregation, racial discrimination, and diabetes: The Coronary Artery Risk Development in Young Adults study.* Health Place, 2020. **62**: p. 102286.
183. McDoom, M.M., et al., *Late life socioeconomic status and hypertension in an aging cohort: the Atherosclerosis Risk in Communities Study.* J Hypertens, 2018. **36**(6): p. 1382-1390.
184. Menec, V.H., S. Shooshtari, S. Nowicki, and S. Fournier, *Does the relationship between neighborhood socioeconomic status and health outcomes persist into very old age? A population-based study.* J Aging Health, 2010. **22**(1): p. 27-47.
185. Mentias, A., et al., *Historical Redlining, Socioeconomic Distress, and Risk of Heart Failure Among Medicare Beneficiaries.* Circulation, 2023. **148**(3): p. 210-219.
186. Metcalf, P.A., et al., *Comparison of different markers of socioeconomic status with cardiovascular disease and diabetes risk factors in the Diabetes, Heart and Health Survey.* N Z Med J, 2008. **121**(1269): p. 45-56.
187. Mezuk, B., et al., *Immigrant enclaves and risk of diabetes: a prospective study.* BMC Public Health, 2014. **14**: p. 1093.
188. Mohottige, D., et al., *Residential Structural Racism and Prevalence of Chronic Health Conditions.* JAMA Netw Open, 2023. **6**(12): p. e2348914.
189. Moore, S., S. Stewart, and A. Teixeira, *Decomposing social capital inequalities in health.* J Epidemiol Community Health, 2014. **68**(3): p. 233-8.
190. Morenoff, J.D., et al., *Understanding social disparities in hypertension prevalence, awareness, treatment, and control: the role of neighborhood context.* Soc Sci Med, 2007. **65**(9): p. 1853-66.
191. Morris, R.W., et al., *Do socioeconomic characteristics of neighbourhood of residence independently influence incidence of coronary heart disease and all-cause mortality in older British men?* Eur J Cardiovasc Prev Rehabil, 2008. **15**(1): p. 19-25.
192. Mujahid, M.S., et al., *Neighborhood characteristics and hypertension.* Epidemiology, 2008. **19**(4): p. 590-8.
193. Müller, G., et al., *Gender differences in the association of individual social class and neighbourhood unemployment rate with prevalent type 2 diabetes mellitus: a cross-sectional study from the DIAB-CORE consortium.* BMJ Open, 2013. **3**(6).

194. Müller, G., et al., *Regional and neighborhood disparities in the odds of type 2 diabetes: results from 5 population-based studies in Germany (DIAB-CORE consortium).* Am J Epidemiol, 2013. **178**(2): p. 221-30.
195. Murray, E.T., et al., *Trajectories of neighborhood poverty and associations with subclinical atherosclerosis and associated risk factors: the multi-ethnic study of atherosclerosis.* Am J Epidemiol, 2010. **171**(10): p. 1099-108.
196. Nakagomi, A., et al., *Association Between Community-Level Social Participation and Self-reported Hypertension in Older Japanese: A JAGES Multilevel Cross-sectional Study.* Am J Hypertens, 2019. **32**(5): p. 503-514.
197. Nazmi, A., et al., *Cross-sectional and longitudinal associations of neighborhood characteristics with inflammatory markers: findings from the multi-ethnic study of atherosclerosis.* Health Place, 2010. **16**(6): p. 1104-12.
198. Neufcourt, L., et al., *Geographical variations in the prevalence of hypertension in France: Cross-sectional analysis of the CONSTANCES cohort.* Eur J Prev Cardiol, 2019. **26**(12): p. 1242-1251.
199. Nikulina, V. and C.S. Widom, *Do race, neglect, and childhood poverty predict physical health in adulthood? A multilevel prospective analysis.* Child Abuse Negl, 2014. **38**(3): p. 414-24.
200. Odoi, E.W., et al., *Sociodemographic Determinants of Acute Myocardial Infarction Hospitalization Risks in Florida.* J Am Heart Assoc, 2020. **9**(11): p. e012712.
201. Ogungbe, O., et al., *Social determinants of hypertension and diabetes among African immigrants: the African immigrants health study.* Ethn Health, 2021: p. 1-13.
202. Ohanyan, H., et al., *Associations between the urban exposome and type 2 diabetes: Results from penalised regression by least absolute shrinkage and selection operator and random forest models.* Environ Int, 2022. **170**: p. 107592.
203. Oktamianti, P., et al., *District-Level Inequalities in Hypertension among Adults in Indonesia: A Cross-Sectional Analysis by Sex and Age Group.* Int J Environ Res Public Health, 2022. **19**(20).
204. Oladele, C.R., et al., *Egocentric Health Networks and Cardiovascular Risk Factors in the ECHORN Cohort Study.* J Gen Intern Med, 2020. **35**(3): p. 784-791.
205. Omariba, D.W., N.A. Ross, C. Sanmartin, and J.V. Tu, *Neighbourhood immigrant concentration and hospitalization: a multilevel analysis of cardiovascular-related admissions in Ontario using linked data.* Can J Public Health, 2014. **105**(6): p. e404-11.
206. Osborn, B., B.N. Morey, J. Billimek, and A. Ro, *Food Insecurity and Type 2 Diabetes Among Latinos: Examining Neighborhood Cohesion as a Protective Factor.* J Racial Ethn Health Disparities, 2023. **10**(4): p. 2061-2070.
207. Pantell, M.S., et al., *Association of Social and Behavioral Risk Factors With Earlier Onset of Adult Hypertension and Diabetes.* JAMA Netw Open, 2019. **2**(5): p. e193933.
208. Penninx, B.W., et al., *Social network, social support, and loneliness in older persons with different chronic diseases.* J Aging Health, 1999. **11**(2): p. 151-68.
209. Pichora, E., et al., *Comparing individual and area-based income measures: impact on analysis of inequality in smoking, obesity, and diabetes rates in Canadians 2003-2013.* Can. J. Public Health-Rev. Can. Sante Publ., 2018. **109**(3): p. 410-418.
210. Pinheiro, L.C., et al., *Multiple Vulnerabilities to Health Disparities and Incident Heart Failure Hospitalization in the REGARDS Study.* Circ Cardiovasc Qual Outcomes, 2020. **13**(8): p. e006438.
211. Piwońska, A.M., et al., *Identifying associations between the social network index, its components, and the prevalence of cardiovascular diseases in Polish adults. Results of the cross-sectional WOBASZ II study.* Kardiol Pol, 2023. **81**(12): p. 1237-1246.
212. Ptushkina, V., et al., *Educational Level, but Not Income or Area Deprivation, is Related to Macrovascular Disease: Results From Two Population-Based Cohorts in Germany.* Int J Public Health, 2021. **66**: p. 633909.
213. Quashie, N.T., et al., *Neighborhood socioeconomic position, living arrangements, and cardiometabolic disease among older Puerto Ricans: An examination using PREHCO 2002-2007.* PLoS One, 2023. **18**(8): p. e0289170.
214. Quiñones, S., A. Goyal, and Z.U. Ahmed, *Geographically weighted machine learning model for untangling spatial heterogeneity of type 2 diabetes mellitus (T2D) prevalence in the USA.* Sci Rep, 2021. **11**(1): p. 6955.

215. Rachele, J.N., B. Giles-Corti, and G. Turrell, *Neighbourhood disadvantage and self-reported type 2 diabetes, heart disease and comorbidity: a cross-sectional multilevel study.* Ann Epidemiol, 2016. **26**(2): p. 146-150.
216. Redondo-Sendino, A., P. Guallar-Castillón, J.R. Banegas, and F. Rodríguez-Artalejo, *[Relationship between social network and hypertension in older people in Spain].* Rev Esp Cardiol, 2005. **58**(11): p. 1294-301.
217. Rethy, L.B., et al., *Neighborhood Poverty and Incident Heart Failure: an Analysis of Electronic Health Records from 2005 to 2018.* J Gen Intern Med, 2021. **36**(12): p. 3719-3727.
218. Riddell, T., *Heart failure hospitalisations and deaths in New Zealand: patterns by deprivation and ethnicity.* N Z Med J, 2004. **118**(1208): p. U1254.
219. Rod, N.H., I. Andersen, and E. Prescott, *Psychosocial risk factors and heart failure hospitalization: a prospective cohort study.* Am J Epidemiol, 2011. **174**(6): p. 672-80.
220. Rose, K.M., et al., *Neighborhood disparities in incident hospitalized myocardial infarction in four U.S. communities: the ARIC surveillance study.* Ann Epidemiol, 2009. **19**(12): p. 867-74.
221. Safford, M.M., et al., *Number of Social Determinants of Health and Fatal and Nonfatal Incident Coronary Heart Disease in the REGARDS Study.* Circulation, 2021. **143**(3): p. 244-253.
222. Salinas, J., et al., *Associations between social relationship measures, serum brain-derived neurotrophic factor, and risk of stroke and dementia.* Alzheimers Dement (N Y), 2017. **3**(2): p. 229-237.
223. Samuel, L.J., R.J. Thorpe, Jr., K.M. Bower, and T.A. LaVeist, *Community Characteristics are Associated with Blood Pressure Levels in a Racially Integrated Community.* J Urban Health, 2015. **92**(3): p. 403-14.
224. Savin, K.L., et al., *Social and built neighborhood environments and blood pressure 6 years later: Results from the Hispanic Community Health Study/Study of Latinos and the SOL CASAS ancillary study.* Soc Sci Med, 2022. **292**: p. 114496.
225. Schieb, L.J., L.R. Mobley, M. George, and M. Casper, *Tracking stroke hospitalization clusters over time and associations with county-level socioeconomic and healthcare characteristics.* Stroke, 2013. **44**(1): p. 146-52.
226. Schootman, M., et al., *The effect of adverse housing and neighborhood conditions on the development of diabetes mellitus among middle-aged African Americans.* American Journal of Epidemiology, 2007. **166**(4): p. 379-387.
227. Schwartz, B.S., et al., *Associations of four indexes of social determinants of health and two community typologies with new onset type 2 diabetes across a diverse geography in Pennsylvania.* PLoS One, 2022. **17**(9): p. e0274758.
228. Schwartz, B.S., et al., *Association of community types and features in a case-control analysis of new onset type 2 diabetes across a diverse geography in Pennsylvania.* BMJ Open, 2021. **11**(1): p. e043528.
229. Sharma, I., et al., *Does the place of residence influence your risk of being hypertensive? A study-based on Nepal Demographic and Health Survey.* Hypertens Res, 2023. **46**(6): p. 1363-1374.
230. Sharp, G. and R.M. Carpiano, *Neighborhood social organization exposures and racial/ethnic disparities in hypertension risk in Los Angeles.* PLoS One, 2023. **18**(3): p. e0282648.
231. Sheets, L., et al., *The Effect of Neighborhood Disadvantage on Diabetes Prevalence.* AMIA Annu Symp Proc, 2017. **2017**: p. 1547-1553.
232. Shibayama, T., H. Noguchi, H. Takahashi, and N. Tamiya, *Relationship between social engagement and diabetes incidence in a middle-aged population: Results from a longitudinal nationwide survey in Japan.* J Diabetes Investig, 2018. **9**(5): p. 1060-1066.
233. Siegel, M., A. Mielck, and W. Maier, *Individual Income, Area Deprivation, and Health: Do Income-Related Health Inequalities Vary by Small Area Deprivation?* Health Econ, 2015. **24**(11): p. 1523-30.
234. Singh, S., R. Zhou, X. Li, and L.P. Tong, *The complex relationship with health: Rural and urban "poor' women.* Int. Soc. Work, 2016. **59**(1): p. 32-46.

235. Smith, G.D., et al., *Individual social class, area-based deprivation, cardiovascular disease risk factors, and mortality: the Renfrew and Paisley Study.* J Epidemiol Community Health, 1998. **52**(6): p. 399-405.
236. Smurthwaite, K. and N. Bagheri, *Using Geographical Convergence of Obesity, Cardiovascular Disease, and Type 2 Diabetes at the Neighborhood Level to Inform Policy and Practice.* Prev Chronic Dis, 2017. **14**: p. E91.
237. Spicer, J., R. Jackson, and R. Scragg, *The Effects of Anger Management and Social Contact on Risk of Myocardial-Infarction in Type-as and Type-Bs.* Psychol. Health, 1993. **8**(4): p. 243-255.
238. Splan, E.D., A.B. Magerman, and C.E. Forbes, *Associations of regional racial attitudes with chronic illness in the United States.* Soc Sci Med, 2021. **281**: p. 114077.
239. Steckel, R.H., *The hidden cost of moving up: type 2 diabetes and the escape from persistent poverty in the American South.* Am J Hum Biol, 2013. **25**(4): p. 508-15.
240. Suchy-Dicey, A., et al., *Psychological and social support associations with mortality and cardiovascular disease in middle-aged American Indians: the Strong Heart Study.* Soc Psychiatry Psychiatr Epidemiol, 2022. **57**(7): p. 1421-1433.
241. Sun, W.J., F. Gong, and J. Xu, *Individual and contextual correlates of cardiovascular diseases among adults in the United States: a geospatial and multilevel analysis.* GeoJournal, 2020. **85**(6): p. 1685-1700.
242. Sundquist, K., M. Malmström, and S.E. Johansson, *Neighbourhood deprivation and incidence of coronary heart disease: a multilevel study of 2.6 million women and men in Sweden.* J Epidemiol Community Health, 2004. **58**(1): p. 71-7.
243. Swain, P.K., B. Behera, and D. Das, *Association between Area-Level Socio-Economic Status and Hypertension in Eag States of India : An Insight from Nfhs-Iv 2015-16.* Int. J. Agric. Stat. Sci., 2019. **15**(1): p. 39-52.
244. Tang, X., et al., *Neighborhood socioeconomic status and the prevalence of stroke and coronary heart disease in rural China: a population-based study.* Int J Stroke, 2015. **10**(3): p. 388-95.
245. Tapager, I., A.M. Bender, and I. Andersen, *A decade of socioeconomic inequality in type 2 diabetes area-level prevalence: an unshakeable status quo?* Scand J Public Health, 2023. **51**(2): p. 268-274.
246. Terashima, M., D.G. Rainham, and A.R. Levy, *A small-area analysis of inequalities in chronic disease prevalence across urban and non-urban communities in the Province of Nova Scotia, Canada, 2007-2011.* BMJ Open, 2014. **4**(5): p. e004459.
247. Thrift, A.G., et al., *Greater incidence of both fatal and nonfatal strokes in disadvantaged areas: the Northeast Melbourne Stroke Incidence Study.* Stroke, 2006. **37**(3): p. 877-82.
248. Tompkins, J.W., I.N. Luginaah, G.L. Booth, and S.B. Harris, *The geography of diabetes in London, Canada: the need for local level policy for prevention and management.* Int J Environ Res Public Health, 2010. **7**(5): p. 2407-22.
249. Trifan, G., et al., *Association of Unfavorable Social Determinants of Health With Stroke/Transient Ischemic Attack and Vascular Risk Factors in Hispanic/Latino Adults: Results From Hispanic Community Health Study/Study of Latinos.* J Stroke, 2023. **25**(3): p. 361-370.
250. Tung, E.L., et al., *Police-Recorded Crime and Disparities in Obesity and Blood Pressure Status in Chicago.* J Am Heart Assoc, 2018. **7**(7).
251. Tung, E.L., et al., *Association of Rising Violent Crime With Blood Pressure and Cardiovascular Risk: Longitudinal Evidence From Chicago, 2014-2016.* Am J Hypertens, 2019. **32**(12): p. 1192-1198.
252. Uddin, J., et al., *The association between neighborhood social and economic environment and prevalent diabetes in urban and rural communities: The Reasons for Geographic and Racial Differences in Stroke (REGARDS) study.* SSM Popul Health, 2022. **17**: p. 101050.
253. Uddin, J., et al., *Age and sex differences in the association between neighborhood socioeconomic environment and incident diabetes: Results from the diabetes location, environmental attributes and disparities (LEAD) network.* SSM Popul Health, 2023. **24**: p. 101541.
254. Usher, T., et al., *Residential Segregation and Hypertension Prevalence in Black and White Older Adults.* J Appl Gerontol, 2018. **37**(2): p. 177-202.
255. Vart, P., et al., *Socioeconomic Status and Incidence of Hospitalization With Lower-Extremity Peripheral Artery Disease: Atherosclerosis Risk in Communities Study.* J Am Heart Assoc, 2017. **6**(8).

256. Villani, M., et al., *Geographical variation of diabetic emergencies attended by prehospital Emergency Medical Services is associated with measures of ethnicity and socioeconomic status.* Sci Rep, 2018. **8**(1): p. 5122.
257. Vintimilla, R., et al., *Association of Area Deprivation Index and hypertension, diabetes, dyslipidemia, and Obesity: A Cross-Sectional Study of the HABS-HD Cohort.* Gerontol Geriatr Med, 2023. **9**: p. 23337214231182240.
258. Vogt, T.M., et al., *Social networks as predictors of ischemic heart disease, cancer, stroke and hypertension: incidence, survival and mortality.* J Clin Epidemiol, 1992. **45**(6): p. 659-66.
259. Wagner, K.J., et al., *Effects of neighborhood socioeconomic status on blood pressure in older adults.* Rev Saude Publica, 2016. **50**: p. 78.
260. Walter, N., C. Robbins, S.T. Murphy, and S.J. Ball-Rokeach, *The weight of networks: the role of social ties and ethnic media in mitigating obesity and hypertension among Latinas.* Ethn Health, 2019. **24**(7): p. 790-803.
261. Wang, W., et al., *Hypertension Prevalence, Awareness, Treatment, and Control and Their Associated Socioeconomic Factors in China: A Spatial Analysis of A National Representative Survey.* Biomed Environ Sci, 2021. **34**(12): p. 937-951.
262. Wang, Q. and Z.L. Lan, *Park green green spaces, public health and social inequalities: Understanding the interrelationships for policy implications.* Land Use Pol., 2019. **83**: p. 66-74.
263. Welin, C.L., A. Rosengren, and L.W. Wilhelmsen, *Social relationships and myocardial infarction: a case-control study.* J Cardiovasc Risk, 1996. **3**(2): p. 183-90.
264. White, K., et al., *Racial/ethnic residential segregation and self-reported hypertension among US- and foreign-born blacks in New York City.* Am J Hypertens, 2011. **24**(8): p. 904-10.
265. White, J.S., et al., *Long-term effects of neighbourhood deprivation on diabetes risk: quasi-experimental evidence from a refugee dispersal policy in Sweden.* Lancet Diabetes Endocrinol, 2016. **4**(6): p. 517-24.
266. Wight, R.G., et al., *A multilevel analysis of urban neighborhood socioeconomic disadvantage and health in late life.* Soc Sci Med, 2008. **66**(4): p. 862-72.
267. Williams, P.C., et al., *Perceived neighborhood social cohesion and type 2 diabetes mellitus by age, sex/gender, and race/ethnicity in the United States.* Prev Med, 2023. **170**: p. 107477.
268. Xiao, Q., et al., *Life-Course Neighborhood Socioeconomic Status and Cardiovascular Events in Black and White Adults in the Atherosclerosis Risk in Communities Study.* Am J Epidemiol, 2022. **191**(8): p. 1470-1484.
269. Xie, H.J., et al., *Built Environment Factors Influencing Prevalence of Hypertension at Community Level in China: The Case of Wuhan.* SUSTAINABILITY, 2021. **13**(10).
270. Xu, J., et al., *Association between neighbourhood deprivation and hypertension in a US-wide Cohort.* J Epidemiol Community Health, 2022. **76**(3): p. 268-273.
271. Xu, J., et al., *Spatial scale analysis for the relationships between the built environment and cardiovascular disease based on multi-source data.* Health Place, 2023. **83**: p. 103048.
272. Yadav, R.S., et al., *Social Determinants of Stroke Hospitalization and Mortality in United States' Counties.* J Clin Med, 2022. **11**(14).
273. Yadav, S., S. Garg, and A.V. Raut, *Evaluation of association of psychosocial stress and hypertension in adults >30 years of age: A community-based case-control study from Rural Central India.* INTERNATIONAL JOURNAL OF NONCOMMUNICABLE DISEASES, 2021. **6**(3): p. 142-148.
274. Yan, L.D., et al., *Neighborhood cohesion and violence in Port-au-Prince, Haiti, and their relationship to stress, depression, and hypertension: Findings from the Haiti cardiovascular disease cohort study.* PLOS Glob Public Health, 2022. **2**(7).
275. Yan, T., et al., *Exploring psychosocial pathways between neighbourhood characteristics and stroke in older adults: the cardiovascular health study.* Age Ageing, 2013. **42**(3): p. 391-7.
276. Yang, Y.C., T. Li, and Y. Ji, *Impact of social integration on metabolic functions: evidence from a nationally representative longitudinal study of US older adults.* BMC Public Health, 2013. **13**: p. 1210.
277. Yang, Y.C., C. Boen, and K. Mullan Harris, *Social relationships and hypertension in late life: evidence from a nationally representative longitudinal study of older adults.* J Aging Health, 2015. **27**(3): p. 403-31.

278. Yang, Y.C., et al., *Social relationships and physiological determinants of longevity across the human life span.* Proc Natl Acad Sci U S A, 2016. **113**(3): p. 578-83.
279. Yazawa, A., et al., *Association between social participation and hypertension among older people in Japan: the JAGES Study.* Hypertens. Res., 2016. **39**(11): p. 818-824.
280. Young, D.R., et al., *Associations of overweight/obesity and socioeconomic status with hypertension prevalence across racial and ethnic groups.* J Clin Hypertens (Greenwich), 2018. **20**(3): p. 532-540.
281. Yu, M.Y., A.J. Velasquez, B. Campos, and J.W. Robinette, *Perceived neighborhood disorder and type 2 diabetes disparities in Hispanic, Black, and White Americans.* Front Public Health, 2024. **12**: p. 1258348.
