# Supplementary material for: Associations between dimensions of the social environment and cardiometabolic health outcomes: a systematic review and meta-analysis
Source: BMJ Open. 2024 Aug 28;14(8):e079987. doi: 10.1136/bmjopen-2023-079987 (PMC11367359; doi:10.1136/bmjopen-2023-079987)
Supplement: online supplemental file 8 [file bmjopen-14-8-s008.pdf]

Supplementary Table 3a. Quality assessment of cross-sectional studies, based on NOS

Reference	First author, year	Total score (max. 10)	%	Selection: Representativeness of the sample (max. 1 point)	Sample size (max. 1 point)	Non-respondents (max. 1 point)	Ascertainment of the exposure (max. 2 points)	Comparability: The subjects in different outcome groups are comparable, based on the study design or analysis. Confounding factors are controlled. (max. 2 point)	Outcome: Assessment of the outcome (max. 2 points)	Statistical test (max. 1 point)
[1]	Abba, 2021	6	60%	1	0	0	1	2	1	1
[2]	Adams, 2009	7	70%	1	0	0	2	1	2	1
[3]	Agabiti, 2009	6	60%	1	0	1	1	1	2	0
[4]	Agyemang, 2007	7	70%	1	0	0	2	1	2	1
[5]	Ahern, 2005	2	20%	0	0	0	0	1	0	1
[6]	Allan, 2020	7	70%	1	0	1	1	2	2	0
[7]	Andersen, 2008	5	50%	1	0	0	2	1	1	0
[8]	Augustin, 2008	6	60%	1	0	0	2	1	1	1
[9]	Avogo, 2023	8	80%	1	0	1	1	2	2	1
[10]	Baek, 2016	5	50%	1	0	0	2	1	1	0
[11]	Banchani, 2020	7	70%	1	0	0	2	1	2	1
[12]	Barber, 2016	3	30%	0	0	0	0	1	2	0
[13]	Barber, 2018	5	50%	0	0	1	2	1	1	0
[14]	Basile Ibrahim, 2021	4	40%	0	0	0	1	1	2	0
[15]	Bevan, 2023	4	40%	1	0	0	1	0	1	1
[16]	Bhopal, 2002	3	30%	0	0	0	0	1	2	0
[17]	Borges, 2021	2	20%	1	0	0	1	0	0	0
[18]	Boruzs, 2018	5	50%	1	0	1	0	1	2	0
[19]	Bravo, 2018	7	70%	1	0	1	1	1	2	1
[20]	Bravo, 2019	7	70%	1	0	1	1	1	2	1
[21]	Brinkhues, 2017	6	60%	0	0	0	2	1	2	1
[22]	Butler, 2010	4	40%	1	0	1	0	0	2	0
[23]	Buys, 2015	8	80%	1	0	0	2	2	2	1
[24]	Cebrecos, 2018	2	20%	1	0	0	0	1	0	0
[25]	Chaix, 2011	6	60%	0	0	0	2	1	2	1
[26]	Chamberlain, 2022	8	80%	1	0	1	1	2	2	1

[27]	Chan, 2022	7	70%	1	0	1	1	2	1	1
[28]	Chum, 2015	6	60%	1	0	0	2	1	1	1
[29]	Coelho, 2023	6	60%	1	0	0	1	2	1	1
[30]	Cofie, 2021	5	50%	1	0	0	1	2	1	0
[31]	Connolly, 2000	6	60%	1	0	1	1	1	2	0
[32]	Consolazio, 2020	6	60%	0	0	0	2	1	2	1
[33]	Cookson, 2012	6	60%	1	0	1	1	1	2	0
[34]	Cox, 2007	6	60%	1	0	1	2	1	1	0
[35]	Cromer, 2023	3	30%	0	0	0	1	1	1	0
[36]	Cubbin, 2006	5	50%	1	0	0	2	1	1	0
[37]	Cunningham, 2018	5	50%	1	0	1	0	1	1	1
[38]	de Oliveira, 2023	4	40%	0	0	0	1	2	1	0
[39]	de Silva, 2022	3	30%	1	0	0	1	0	1	0
[40]	Desmond, 2015	5	50%	0	0	0	2	1	1	1
[41]	Diez-Roux, 1997	6	60%	1	0	0	2	1	1	1
[42]	Diez-Roux, 2000	6	60%	1	0	0	2	1	1	1
[43]	Djekic, 2018	3	30%	1	0	0	0	0	2	0
[44]	Dragano, 2007	7	70%	1	0	0	2	1	2	1
[45]	Dubowitz, 2012	6	60%	0	0	0	2	1	2	1
[46]	Dwane, 2020	4	40%	0	0	0	2	1	1	0
[47]	Dyck, 2021	7	70%	1	0	1	1	1	2	1
[48]	Ekholuenetale, 2020	6	60%	1	0	0	2	1	1	1
[49]	Engström, 2001	5	50%	0	0	0	1	1	1	1
[50]	Eschbach, 2004	5	50%	1	0	0	1	1	1	1
[51]	Faka, 2018	2	20%	1	0	0	0	0	0	1
[52]	Feero, 1995	6	60%	1	0	1	1	1	2	0
[53]	Ferguson, 2020	7	70%	1	1	1	2	0	2	1
[54]	Fitzpatrick, 2020	4	40%	1	0	0	0	1	1	1
[55]	Fleischer, 2008	6	60%	1	0	0	2	1	1	1
[56]	Ford, 2006	6	60%	1	0	0	1	2	1	1
[57]	Gary-webb, 2023	7	70%	1	0	1	1	2	1	1
[58]	Gaskin, 2014	4	40%	0	0	0	2	1	1	0
[59]	Glover, 2019	5	50%	0	0	0	2	1	1	1
[60]	Grundmann, 2014	6	60%	1	0	0	2	1	1	1
[61]	Hanigan, 2017	4	40%	1	0	1	0	1	0	1

[62]	Hashemi, 2023	4	40%	0	0	1	1	1	1	0
[63]	Hawkins, 2012	5	50%	1	0	1	1	1	1	0
[64]	Heredia, 2022	5	50%	0	0	0	1	2	1	1
[65]	Herrick, 2016	4	40%	0	0	0	1	1	2	0
[66]	Höfelmann, 2012	6	60%	1	1	1	2	1	1	1
[67]	Holstiege, 2019	6	60%	1	0	0	1	1	2	1
[68]	Holtgrave, 2006	3	30%	1	0	0	0	1	1	0
[69]	Horsten, 1999	4	40%	1	0	0	0	1	1	1
[70]	Hosseini, 2020	6	60%	1	0	0	2	1	1	1
[71]	Hu, 2020	3	30%	1	0	1	0	0	1	0
[72]	Hu, 2021	4	40%	0	0	0	2	1	1	0
[73]	Huang, 2022	4	40%	1	0	1	1	0	1	0
[74]	Jackson, 2008	4	40%	0	0	0	2	0	1	1
[75]	Jadow, 2023	1	10%	0	0	0	1	0	0	0
[76]	Jain, 2022	6	60%	1	0	1	1	2	1	0
[77]	Jonsson, 2020	5	50%	1	0	1	0	1	2	0
[78]	Jung, 2019	6	60%	1	0	1	0	1	2	1
[79]	Kauhl, 2018	4	40%	1	0	1	1	1	1	0
[80]	Keita, 2014	4	40%	0	0	0	2	1	1	0
[81]	Kelli, 2017	3	30%	0	0	0	0	1	2	0
[82]	Kershaw, 2011	6	60%	1	0	0	2	1	1	1
[83]	Kolpak, 2017	3	30%	1	0	1	0	0	1	0
[84]	Krieger, 1992	4	40%	0	0	0	1	1	2	0
[85]	Kwok, 2021	5	50%	1	0	0	1	1	1	1
[86]	Lagisetty, 2016	6	60%	0	0	0	2	2	2	0
[87]	Larrañaga, 2005	6	60%	1	0	1	1	1	2	0
[88]	Lawlor, 2005	6	60%	1	0	0	2	1	1	1
[89]	Lee, 2018	5	50%	0	0	0	2	1	2	0
[90]	Lee, 2018	4	40%	1	0	1	1	0	1	0
[91]	Lemstra, 2006	6	60%	1	0	1	0	1	2	1
[92]	Leyland, 2005	6	60%	1	0	0	2	1	1	1
[93]	Li, 2017	5	50%	0	0	0	2	1	1	1
[94]	Linde, 2023	4	40%	1	0	0	1	2	0	0
[95]	Loucks, 2006	3	30%	0	0	0	1	1	0	1
[96]	Lu, 2019	6	60%	0	0	0	2	1	2	1
[97]	Lukachko, 2014	5	50%	1	0	0	2	1	1	0

[98]	Ma, 2021	5	50%	1	0	0	1	2	1	0
[99]	Madela, 2023	5	50%	0	0	0	1	2	1	1
[100]	Madela, 2023	5	50%	0	0	0	1	2	1	1
[101]	Maheswaran, 2018	4	40%	1	0	0	1	1	1	0
[102]	Maier, 2014	6	60%	1	0	0	2	1	1	1
[103]	Malino, 2014	6	60%	0	1	1	1	1	2	0
[104]	Massa, 2016	5	50%	1	0	0	2	0	1	1
[105]	Matheson, 2010	6	60%	1	0	0	2	1	1	1
[106]	Menec, 2010	9	90%	1	0	1	2	2	2	1
[107]	Metcalf, 2008	2	20%	1	0	0	1	0	0	0
[108]	Mezuk, 2014	8	80%	1	0	1	2	1	2	1
[109]	Mohottige, 2023	6	60%	1	0	1	1	1	2	0
[110]	Moore, 2014	5	50%	1	0	0	2	1	1	0
[111]	Morenoff, 2007	6	60%	1	0	0	2	1	1	1
[112]	Mujahid, 2008	5	50%	1	0	0	2	1	1	0
[113]	Müller, 2013	6	60%	1	0	0	2	1	1	1
[114]	Müller, 2013	6	60%	1	0	0	2	1	1	1
[115]	Nakagomi, 2019	7	70%	1	0	0	2	2	1	1
[116]	Nazmi, 2010	4	40%	1	0	0	0	2	1	0
[117]	Neufcourt, 2019	7	70%	1	0	0	2	1	2	1
[118]	Ogungbe, 2021	5	50%	0	0	0	1	2	1	1
[119]	Ohanyan, 2022	6	60%	1	0	1	1	2	1	0
[120]	Oktamianti, 2022	4	40%	1	0	0	1	0	2	0
[121]	Oladele, 2020	6	60%	1	0	0	2	1	1	1
[122]	Osborn, 2023	4	40%	0	0	0	1	2	1	0
[123]	Penninx, 1999	7	70%	1	0	1	2	1	1	1
[124]	Pichora, 2018	4	40%	1	0	1	0	1	1	0
[125]	Piwońska, 2023	5	50%	1	0	0	1	2	1	0
[126]	Ptushkina, 2021	5	50%	1	0	0	1	2	1	0
[127]	Quinones, 2021	4	40%	1	0	1	1	0	1	0
[128]	Rachele, 2016	6	60%	1	0	0	2	1	1	1
[129]	Redondo-Sendino, 2005	8	80%	1	0	1	2	1	2	1
[130]	Riddell, 2004	3	30%	1	0	0	0	0	2	0
[131]	Samuel, 2015	5	50%	0	0	0	2	1	1	1
[132]	Sharma, 2023	7	70%	1	0	0	2	2	1	1

[133]	Sheets, 2017	3	30%	0	0	0	1	1	1	0
[134]	Siegel, 2015	4	40%	1	0	0	1	1	1	0
[135]	Singh, 2016	4	40%	1	0	1	0	1	1	0
[136]	Smith, 1998	6	60%	1	0	0	2	1	2	0
[137]	Smurthwaite, 2017	3	30%	0	0	1	0	0	2	0
[138]	Splan, 2021	5	50%	0	0	0	1	2	1	1
[139]	Sun, 2020	6	60%	1	0	0	2	1	1	1
[140]	Swain, 2019	2	20%	1	0	1	0	0	0	0
[141]	Tang, 2015	5	50%	0	0	0	2	1	1	1
[142]	Tapager, 2023	6	67%	1	0	1	1	1	2	0
[143]	Terashima, 2014	5	50%	1	0	0	1	1	1	1
[144]	Tompkins, 2010	4	40%	1	0	1	0	0	2	0
[145]	Trifan, 2023	3	30%	0	0	0	1	1	1	0
[146]	Tung, 2018	7	70%	0	0	1	2	1	2	1
[147]	Uddin, 2022	3	30%	0	0	0	1	1	1	0
[148]	Usher, 2018	2	20%	0	0	0	0	1	1	0
[149]	Vintimilla, 2023	4	40%	0	0	0	1	2	1	0
[150]	Wagner, 2016	8	80%	1	1	1	2	1	1	1
[151]	Walter, 2019	6	60%	0	0	0	2	1	2	1
[152]	Wang, 2019	6	60%	1	0	1	0	1	2	1
[153]	Wang, 2021	6	60%	1	0	1	1	2	1	0
[154]	White, 2011	5	50%	0	0	0	2	1	1	1
[155]	Wight, 2008	6	60%	1	0	0	2	1	1	1
[156]	Williams, 2023	5	50%	1	0	0	2	2	0	0
[157]	Xie, 2021	2	20%	1	0	0	1	0	0	0
[158]	Xu, 2023	3	30%	1	0	0	1	0	1	0
[159]	Xu, 2022	5	50%	1	0	0	1	2	1	0
[160]	Yadav, 2022	2	20%	0	0	0	1	0	1	0
[161]	Yan, 2022	6	60%	1	0	0	2	2	1	0
[162]	Yang, 2013	3	30%	1	0	1	0	1	0	0
[163]	Yazawa, 2016	6	60%	1	0	0	2	1	1	1
[164]	Young, 2018	4	40%	0	0	1	0	1	2	0
[165]	Yu, 2024	4	40%	1	0	0	1	2	0	0

Supplementary Table 3b. Quality assessment of longitudinal / cohort studies

				Selection				Comparability	Outcome		
**Reference**	**First author, year**	**Total score (max. 9)**	**%**	Representativeness of the exposed cohort (max. 1 point)	Selection of the non-exposed cohort (max. 1 point)	Ascertainment of exposure (max. 1 point)	Demonstration that outcome of interest was not present at start of study (max. 1 point)	Comparability of cohorts on the basis of the design or analysis (max. 2 points)	Assessment of outcome (max. 1 point)	Was follow-up long enough for outcomes to occur (max. 1 point)	Adequacy of follow up of cohorts (max. 1 point)
[166]	Akwo, 2018	7	78%	0	1	1	2	1	1	1	0
[167]	Alemi, 2023	4	44%	1	1	0	0	0	0	1	1
[168]	Altevers, 2016	8	89%	1	1	1	2	1	1	1	0
[169]	Anderson, 2019	5	56%	1	1	0	2	0	0	1	0
[170]	Atasoy, 2022	7	78%	0	1	1	1	2	1	1	0
[171]	Bancks, 2017	9	100%	1	1	1	2	1	1	1	1
[172]	Barefoot, 2005	6	67%	1	1	1	2	0	1	0	0
[173]	Bhavsar, 2022	6	67%	0	1	1	0	1	1	1	1
[174]	Bilal, 2018	7	78%	1	1	1	1	1	1	0	1
[175]	Bocour, 2016	6	67%	1	1	0	0	1	1	1	1
[176]	Bray, 2018	8	89%	1	1	1	1	1	1	1	1
[177]	Bush, 2023	8	89%	1	1	1	1	2	0	1	1
[178]	Carlsson, 2016	8	89%	1	1	0	2	1	1	1	1
[179]	Carlsson, 2017	8	89%	1	1	0	2	1	1	1	1
[180]	Cené, 2022	6	67%	0	1	1	1	2	0	1	0
[181]	Chang, 2017	6	67%	0	1	1	2	0	1	1	0
[182]	Chatzi, 2020	7	78%	1	1	1	2	0	1	1	0
[183]	Child , 2022	2	22%	0	1	1	0	0	0	0	0
[184]	Christine, 2015	8	89%	1	1	1	2	1	1	1	0
[185]	Clark, 2011	7	78%	0	1	1	2	1	1	1	0
[186]	Claudel, 2018	7	78%	0	1	1	2	1	0	1	1
[187]	Cozier, 2007	6	67%	0	1	1	2	1	0	0	1
[188]	Cuthbertson, 2018	6	67%	0	1	1	1	1	1	0	1
[189]	Diez Roux, 2002	8	89%	0	1	1	2	1	1	1	1
[190]	Eng, 2002	8	89%	0	1	1	2	1	1	1	1
[191]	Essien, 2022	8	89%	1	1	1	1	1	1	1	1

[192]	Exeter, 2015	8	89%	1	1	1	2	1	1	0	1
[193]	Forsberg, 2018	9	100%	1	1	1	2	1	1	1	1
[194]	Forsberg, 2023	9	100%	1	1	1	1	2	1	1	1
[195]	Freedman, 2011	7	78%	1	1	1	2	1	0	0	1
[196]	Gao, 2022	6	67%	0	1	1	1	2	0	1	0
[197]	Garcia, 2015	6	67%	0	1	1	2	1	0	1	0
[198]	Garcia, 2016	5	56%	0	1	1	1	1	0	1	0
[199]	Gebreab, 2017	6	67%	0	1	1	2	1	0	1	0
[200]	Gero, 2022	7	78%	1	1	1	1	2	0	1	0
[201]	Guion, 2024	8	89%	1	1	1	1	1	1	1	1
[202]	Gwon, 2020	8	89%	1	1	1	2	1	1	1	0
[203]	Halonen, 2015	6	67%	0	1	1	1	1	1	0	1
[204]	Hamad, 2020	7	78%	0	1	1	1	1	1	1	1
[205]	Hanefeld, 2018	9	100%	1	1	1	1	1	1	1	1
[206]	Harding, 2022	6	67%	0	1	1	1	2	0	1	0
[207]	Hassen, 2020	6	67%	1	1	0	2	1	0	1	0
[208]	Heeley, 2011	3	33%	0	1	0	1	1	0	0	0
[209]	Hendryx, 2020	8	89%	1	1	1	2	1	0	1	1
[210]	Henriksson, 2010	9	100%	1	1	1	2	1	1	1	1
[211]	Herrera-Añazco, 2019	8	89%	0	1	1	2	1	1	1	1
[212]	Hilding, 2015	8	89%	0	1	1	2	1	1	1	1
[213]	Hill, 2014	6	67%	1	1	1	2	1	0	0	0
[214]	Honda, 2021	7	78%	0	1	1	1	2	1	1	0
[215]	Honjo, 2015	8	89%	1	1	1	2	1	1	1	0
[216]	Howard, 2016	8	89%	0	1	1	2	1	1	1	1
[217]	Hwang, 2020	9	100%	1	1	1	2	1	1	1	1
[218]	Jack, 2019	8	89%	1	1	1	1	1	1	1	1
[219]	Kaiser, 2016	6	67%	1	1	1	2	1	0	1	0
[220]	Kakinami, 2017	7	78%	1	1	1	1	1	1	0	1
[221]	Kawachi, 1996	5	56%	0	1	1	1	1	0	0	1
[222]	Kim, 2022	7	78%	1	1	1	1	2	0	1	0
[223]	Kivimäki, 2018	8	89%	1	1	1	2	1	1	1	0
[224]	Kivimaki, 2021	7	78%	0	1	1	0	2	1	1	1
[225]	Krishnan, 2010	7	78%	0	1	1	2	1	0	1	1
[226]	Lachkhem, 2018	8	89%	1	1	1	1	1	1	1	1

[227]	Laursen, 2017	7	78%	1	1	1	2	1	0	1	0
[228]	Ling, 2009	6	67%	1	1	0	1	1	1	1	0
[229]	Lippert, 2017	6	67%	1	1	0	2	1	0	1	0
[230]	Lönn, 2019	9	100%	1	1	1	2	1	1	1	1
[231]	Lukaschek, 2017	6	67%	1	1	1	1	0	0	1	1
[232]	Lund, 2012	5	56%	1	1	1	2	1	0	0	0
[233]	Lund, 2014	8	89%	1	1	1	2	1	1	0	1
[234]	Marley, 2015	6	67%	1	1	0	2	1	0	0	1
[235]	Matthew, 2018	7	78%	1	1	0	2	1	0	1	1
[236]	Mayne, 2020	8	89%	0	1	1	2	1	1	1	1
[237]	McDoom, 2018	7	78%	1	1	1	2	1	0	1	0
[238]	Mentias, 2023	8	89%	1	1	1	1	2	1	1	0
[239]	Morris, 2008	8	89%	1	1	1	1	1	1	1	1
[240]	Murray, 2010	6	67%	1	1	0	2	1	0	1	0
[241]	Nikulina, 2014	7	78%	0	1	0	2	1	1	1	1
[242]	Odoi, 2020	8	89%	1	1	0	2	1	1	1	1
[243]	Omariba, 2014	8	89%	1	1	0	2	1	1	1	1
[244]	Pantell, 2019	6	67%	0	1	1	1	1	1	0	1
[245]	Pinheiro, 2020	6	67%	0	1	1	1	1	1	1	0
[246]	Quashie, 2023	6	67%	1	1	1	0	2	0	0	1
[247]	Rethy, 2021	6	67%	0	1	1	1	1	1	1	0
[248]	Rod, 2011	9	100%	1	1	1	2	1	1	1	1
[249]	Rose, 2009	8	89%	1	1	1	1	1	1	1	1
[250]	Safford, 2021	7	78%	0	1	1	1	1	1	1	1
[251]	Salinas, 2017	8	89%	1	1	1	2	1	0	1	1
[252]	Savin, 2022	5	56%	0	1	1	1	0	1	1	0
[253]	Schieb, 2013	4	44%	0	1	0	0	0	1	1	1
[254]	Schootman, 2007	4	44%	0	1	1	0	1	0	0	1
[255]	Schwartz, 2021	9	100%	1	1	1	2	1	1	1	1
[256]	Sharp, 2023	6	67%	0	1	1	0	2	0	1	1
[257]	Shibayama, 2018	6	67%	1	1	1	1	1	0	1	0
[258]	Steckel, 2013	7	78%	1	1	1	0	1	1	1	1
[259]	Suchy-Dicey, 2022	7	78%	0	1	1	1	2	0	1	1
[260]	Sundquist, 2004	9	100%	1	1	1	2	1	1	1	1
[261]	Thrift, 2006	7	78%	1	1	1	0	1	1	1	1
[262]	Tung, 2019	7	78%	0	1	1	2	1	1	0	1

[263]	Uddin, 2023	7	78%	0	1	1	1	2	1	1	0
[264]	Vart, 2017	8	89%	1	1	1	1	1	1	1	1
[265]	Villani, 2018	7	78%	1	1	0	1	1	1	1	1
[266]	Vogt, 1992	7	78%	0	1	1	2	1	1	1	0
[267]	Xiao, 2022	8	89%	1	1	1	1	2	1	1	0
[268]	Yan, 2013	5	56%	1	1	0	2	0	0	1	0
[269]	Yang, 2015	7	78%	1	1	0	2	1	1	1	0
[270]	Yang, 2016	8	89%	1	1	1	2	1	1	1	0

Supplementary Table 3c. Quality assessment of case-control studies

				**Selection**				**Comparability**	**Exposure**		
**Reference**	**First author, year**	**Total score (max. 9)**	**%**	Is the case definition adequate ? (max. 1 point)	Representativeness of the cases (max. 1 point)	Selection of controls (max. 1 point)	Definition of controls (max. 1 point)	Comparability of cases and controls on the basis of the design or analysis (max. 1 point)	Ascertainment of exposure (max. 2 point)	Same method of ascertainment for cases and controls (max. 1 point)	Non-Response rate (max. 1 point)
[271]	Spicer, 1993	4	44%	1	0	1	0	2	0	0	0
[272]	Welin, 1996	5	56%	1	1	1	1	0	0	0	1
[273]	Cheruvalath, 2022	7	78%	1	1	0	1	1	1	1	1
[274]	Schwartz, 2022	7	78%	1	1	0	1	1	1	1	1
[275]	Yadav, 2021	6	67%	1	1	1	1	1	0	1	0

Supplementary Table 3d. Quality assessment of randomized studies, based on Rob 2

**Reference**	**First author, year**	**Risk of bias arising from randomisation process**	**Risk of bias due to deviation from intended intervention**	**Risk of bias due to missing outcome data**	**Risk of bias in measurement of outcome**	**Risk of bias in selection of the reported result**	**Overall Risk of bias**
[276]	Ludwig, 2011	low risk	low risk	high risk	low risk	some concerns	High risk of bias
[277]	Kling, 2018	low risk	low risk	high risk	high risk	some concerns	High risk of bias

Supplementary Table 3e. Quality assessment of non-randomized studies of interventions, based on ROBINS-I

Reference	First author, year	Bias due to confounding	Bias in selection of participants into the study	Bias in classification of interventions	Bias due to deviations from intended interventions	Bias due to missing data	Bias in measurement of outcomes	Bias in selection of the reported result	Overall Risk of bias
[278]	White, 2016	moderate risk	low risk	low risk	moderate risk	low risk	low risk	moderate risk	Moderate risk of bias
[279]	Kim, 2018	serious risk	low risk	low risk	low risk	no information	moderate risk	moderate risk	Serious risk of bias
[280]	Jensen, 2023	moderate risk	low risk	low risk	No information	moderate risk	low risk	moderate risk	Moderate risk of bias
[281]	Kim, 2022	moderate risk	low risk	low risk	low risk	serious risk	serious risk	low risk	Serious risk of bias

1. Abba, M.S., et al., *Influence of contextual socioeconomic position on hypertension risk in low- and middle-income countries: disentangling context from composition.* BMC Public Health, 2021. **21**(1): p. 2218.
2. Adams, R.J., et al., *Effects of area deprivation on health risks and outcomes: a multilevel, cross-sectional, Australian population study.* Int J Public Health, 2009. **54**(3): p. 183-92.
3. Agabiti, N., et al., *Income level and chronic ambulatory care sensitive conditions in adults: a multicity population-based study in Italy.* BMC Public Health, 2009. **9**: p. 457.
4. Agyemang, C., et al., *Ethnic differences in the effect of environmental stressors on blood pressure and hypertension in the Netherlands.* BMC Public Health, 2007. **7**: p. 118.
5. Ahern, M.M. and M.S. Hendryx, *Social capital and risk for chronic illnesses.* Chronic Illn, 2005. **1**(3): p. 183-90.
6. Allan, K.S., et al., *High risk neighbourhoods: The effect of neighbourhood level factors on cardiac arrest incidence.* Resuscitation, 2020. **149**: p. 100-108.
7. Andersen, A.F., et al., *Life-course socio-economic position, area deprivation and Type 2 diabetes: findings from the British Women's Heart and Health Study.* Diabet Med, 2008. **25**(12): p. 1462-8.
8. Augustin, T., T.A. Glass, B.D. James, and B.S. Schwartz, *Neighborhood psychosocial hazards and cardiovascular disease: the Baltimore Memory Study.* Am J Public Health, 2008. **98**(9): p. 1664-70.
9. Avogo, W.A., *Community characteristics and the risk of non-communicable diseases in Ghana.* PLOS Glob Public Health, 2023. **3**(1): p. e0000692.
10. Baek, J., N.W. Hur, H.C. Kim, and Y. Youm, *Sex-specific effects of social networks on the prevalence, awareness, and control of hypertension among older Korean adults.* J Geriatr Cardiol, 2016. **13**(7): p. 580-6.
11. Banchani, E., E.Y. Tenkorang, and W. Midodzi, *Examining the effects of individual and neighbourhood socioeconomic status/wealth on hypertension among women in the Greater Accra Region of Ghana.* Health Soc Care Community, 2020.
12. Barber, S., et al., *Neighborhood Disadvantage, Poor Social Conditions, and Cardiovascular Disease Incidence Among African American Adults in the Jackson Heart Study.* Am J Public Health, 2016. **106**(12): p. 2219-2226.
13. Barber, S., et al., *At the intersection of place, race, and health in Brazil: Residential segregation and cardio-metabolic risk factors in the Brazilian Longitudinal Study of Adult Health (ELSA-Brasil).* Soc Sci Med, 2018. **199**: p. 67-76.

14. Basile Ibrahim, B., et al., *The Association Between Neighborhood Social Vulnerability and Cardiovascular Health Risk Among Black/African American Women in the InterGEN Study.* Nurs Res, 2021. **70**(5): p. S3-s12.
15. Bevan, G., et al., *Neighborhood-level Social Vulnerability and Prevalence of Cardiovascular Risk Factors and Coronary Heart Disease.* Curr Probl Cardiol, 2023. **48**(8): p. 101182.
16. Bhopal, R., et al., *Ethnic and socio-economic inequalities in coronary heart disease, diabetes and risk factors in Europeans and South Asians.* J Public Health Med, 2002. **24**(2): p. 95-105.
17. Borges, C.M., et al., *Social capital or vulnerability: Which has the stronger connection with selected U.S. health outcomes?* SSM Popul Health, 2021. **15**: p. 100812.
18. Boruzs, K., et al., *High Inequalities Associated With Socioeconomic Deprivation in Cardiovascular Disease Burden and Antihypertensive Medication in Hungary.* Front Pharmacol, 2018. **9**: p. 839.
19. Bravo, M.A., R. Anthopolos, R.T. Kimbro, and M.L. Miranda, *Residential Racial Isolation and Spatial Patterning of Type 2 Diabetes Mellitus in Durham, North Carolina.* Am J Epidemiol, 2018. **187**(7): p. 1467-1476.
20. Bravo, M.A., B.C. Batch, and M.L. Miranda, *Residential Racial Isolation and Spatial Patterning of Hypertension in Durham, North Carolina.* Prev Chronic Dis, 2019. **16**: p. E36.
21. Brinkhues, S., et al., *Socially isolated individuals are more prone to have newly diagnosed and prevalent type 2 diabetes mellitus - the Maastricht study.* BMC Public Health, 2017. **17**(1): p. 955.
22. Butler, D.C., S. Petterson, A. Bazemore, and K.A. Douglas, *Use of measures of socioeconomic deprivation in planning primary health care workforce and defining health care need in Australia.* Aust J Rural Health, 2010. **18**(5): p. 199-204.
23. Buys, D.R., et al., *Association between neighborhood disadvantage and hypertension prevalence, awareness, treatment, and control in older adults: results from the University of Alabama at Birmingham Study of Aging.* Am J Public Health, 2015. **105**(6): p. 1181-8.
24. Cebrecos, A., et al., *Geographic and statistic stability of deprivation aggregated measures at different spatial units in health research.* Appl. Geogr., 2018. **95**: p. 9-18.
25. Chaix, B., et al., *Neighborhood effects on health: correcting bias from neighborhood effects on participation.* Epidemiology, 2011. **22**(1): p. 18-26.
26. Chamberlain, A.M., et al., *Associations of Neighborhood Socioeconomic Disadvantage With Chronic Conditions by Age, Sex, Race, and Ethnicity in a Population-Based Cohort.* Mayo Clin Proc, 2022. **97**(1): p. 57-67.
27. Chan, J.J.L., et al., *Inequalities in the prevalence of cardiovascular disease risk factors in Brazilian slum populations: A cross-sectional study.* PLOS Glob Public Health, 2022. **2**(9): p. e0000990.
28. Chum, A. and P. O'Campo, *Cross-sectional associations between residential environmental exposures and cardiovascular diseases.* BMC Public Health, 2015. **15**: p. 438.
29. Coelho, D.M., et al., *Gender differences in the association of individual and contextual socioeconomic status with hypertension in 230 Latin American cities from the SALURBAL study: a multilevel analysis.* BMC Public Health, 2023. **23**(1): p. 1532.
30. Cofie, L.E., J.M. Hirth, and J.G.L. Lee, *Social Support Networks and Foreign-Birth Status Associated With Obesity, Hypertension and Diabetes Prevalence Among 21-30 and 50-70 Year Old Adults Living in the San Francisco Bay Area.* Am J Health Promot, 2021. **35**(8): p. 1105-1113.
31. Connolly, V., et al., *Diabetes prevalence and socioeconomic status: a population based study showing increased prevalence of type 2 diabetes mellitus in deprived areas.* Journal of Epidemiology and Community Health, 2000. **54**(3): p. 173-177.
32. Consolazio, D., et al., *Neighbourhood property value and type 2 diabetes mellitus in the Maastricht study: A multilevel study.* PLoS One, 2020. **15**(6): p. e0234324.
33. Cookson, R., M. Laudicella, and P.L. Donni, *Measuring change in health care equity using small-area administrative data - evidence from the English NHS 2001-2008.* Soc Sci Med, 2012. **75**(8): p. 1514-22.
34. Cox, M., et al., *Locality deprivation and Type 2 diabetes incidence: a local test of relative inequalities.* Soc Sci Med, 2007. **65**(9): p. 1953-64.

35. Cromer, S.J., et al., *Association and Interaction of Genetics and Area-Level Socioeconomic Factors on the Prevalence of Type 2 Diabetes and Obesity.* Diabetes Care, 2023. **46**(5): p. 944-952.
36. Cubbin, C., et al., *Neighborhood deprivation and cardiovascular disease risk factors: protective and harmful effects.* Scand J Public Health, 2006. **34**(3): p. 228-37.
37. Cunningham, S.A., et al., *County-level contextual factors associated with diabetes incidence in the United States.* Ann Epidemiol, 2018. **28**(1): p. 20-25.e2.
38. de Oliveira, F.L.P., et al., *Spatial clusters of diabetes: individual and neighborhood characteristics in the ELSA-Brasil cohort study.* CADERNOS DE SAUDE PUBLICA, 2023. **39**(5).
39. De Silva, S.S.A., et al., *Comparing Global and Spatial Composite Measures of Neighborhood Socioeconomic Status Across US Counties.* J Urban Health, 2022. **99**(3): p. 457-468.
40. Desmond, M. and W.H. An, *Neighborhood and Network Disadvantage among Urban Renters.* Sociol. Sci., 2015. **2**: p. 329-349.
41. Diez-Roux, A.V., et al., *Neighborhood environments and coronary heart disease: a multilevel analysis.* Am J Epidemiol, 1997. **146**(1): p. 48-63.
42. Diez-Roux, A.V., B.G. Link, and M.E. Northridge, *A multilevel analysis of income inequality and cardiovascular disease risk factors.* Soc. Sci. Med., 2000. **50**(5): p. 673-687.
43. Djekic, D., et al., *Impact of socioeconomic status on coronary artery calcification.* Eur J Prev Cardiol, 2018. **25**(16): p. 1756-1764.
44. Dragano, N., et al., *Neighbourhood socioeconomic status and cardiovascular risk factors: a multilevel analysis of nine cities in the Czech Republic and Germany.* BMC Public Health, 2007. **7**: p. 255.
45. Dubowitz, T., et al., *The Women's Health Initiative: The food environment, neighborhood socioeconomic status, BMI, and blood pressure.* Obesity (Silver Spring), 2012. **20**(4): p. 862-71.
46. Dwane, N., N. Wabiri, and S. Manda, *Small-area variation of cardiovascular diseases and select risk factors and their association to household and area poverty in South Africa: Capturing emerging trends in South Africa to better target local level interventions.* PLoS One, 2020. **15**(4): p. e0230564.
47. Dyck, J., R. Tate, J. Uhanova, and M. Torabi, *Social determinants and spatio-temporal variation of Ischemic Heart Disease in Manitoba.* BMC Public Health, 2021. **21**(1): p. 2325.
48. Ekholuenetale, M. and A. Barrow, *Prevalence and determinants of self-reported high blood pressure among women of reproductive age in Benin: a population-based study.* Clin Hypertens, 2020. **26**: p. 12.
49. Engström, G., et al., *Geographic distribution of stroke incidence within an urban population: Relations to socioeconomic circumstances and prevalence of cardiovascular risk factors.* Stroke, 2001. **32**(5): p. 1098-1103.
50. Eschbach, K., et al., *Neighborhood context and mortality among older Mexican Americans: is there a barrio advantage?* Am J Public Health, 2004. **94**(10): p. 1807-12.
51. Faka, A., et al., *Association of Socio-Environmental Determinants with Diabetes Prevalence in the Athens Metropolitan Area, Greece: A Spatial Analysis.* Rev Diabet Stud, 2018. **14**(4): p. 381-389.
52. Feero, S., J.R. Hedges, and P. Stevens, *Demographics of cardiac arrest: association with residence in a low-income area.* Acad Emerg Med, 1995. **2**(1): p. 11-6.
53. Ferguson, T.S., et al., *Neighbourhood socioeconomic characteristics and blood pressure among Jamaican youth: a pooled analysis of data from observational studies.* PeerJ, 2020. **8**: p. e10058.
54. Fitzpatrick, K.M. and D. Willis, *Chronic Disease, the Built Environment, and Unequal Health Risks in the 500 Largest U.S. Cities.* Int J Environ Res Public Health, 2020. **17**(8).
55. Fleischer, N.L., A.V. Diez Roux, M. Alazraqui, and H. Spinelli, *Social patterning of chronic disease risk factors in a Latin American city.* J Urban Health, 2008. **85**(6): p. 923-37.
56. Ford, E.S., E.B. Loucks, and L.F. Berkman, *Social integration and concentrations of C-reactive protein among US adults.* Ann Epidemiol, 2006. **16**(2): p. 78-84.
57. Gary-Webb, T.L., et al., *Community stressors (violence, victimization, and neighborhood disorder) with cardiometabolic outcomes in urban Jamaica.* Front Public Health, 2023. **11**: p. 1130830.

58. Gaskin, D.J., et al., *Disparities in diabetes: the nexus of race, poverty, and place.* Am J Public Health, 2014. **104**(11): p. 2147-55.
59. Glover, L.M., et al., *Sex differences in the association of psychosocial resources with prevalent type 2 diabetes among African Americans: The Jackson Heart Study.* J Diabetes Complications, 2019. **33**(2): p. 113-117.
60. Grundmann, N., A. Mielck, M. Siegel, and W. Maier, *Area deprivation and the prevalence of type 2 diabetes and obesity: analysis at the municipality level in Germany.* BMC Public Health, 2014. **14**: p. 1264.
61. Hanigan, I.C., T. Cochrane, and R. Davey, *Impact of scale of aggregation on associations of cardiovascular hospitalization and socio-economic disadvantage.* PLoS One, 2017. **12**(11): p. e0188161.
62. Hashemi, S.J., et al., *Social determinants of health and diabetes: Results from a cohort study in Iran.* ASIAN JOURNAL OF SOCIAL HEALTH AND BEHAVIOR, 2023. **6**(2): p. 86-91.
63. Hawkins, N.M., et al., *Community care in England: reducing socioeconomic inequalities in heart failure.* Circulation, 2012. **126**(9): p. 1050-7.
64. Heredia, N.I., et al., *The Neighborhood Environment and Hispanic/Latino Health.* Am J Health Promot, 2022. **36**(1): p. 38-45.
65. Herrick, C.J., B.W. Yount, and A.A. Eyler, *Implications of supermarket access, neighbourhood walkability and poverty rates for diabetes risk in an employee population.* Public Health Nutr, 2016. **19**(11): p. 2040-8.
66. Höfelmann, D.A., J.L. Antunes, D.A. Santos Silva, and M.A. Peres, *Is income area level associated with blood pressure in adults regardless of individual-level characteristics? A multilevel approach.* Health Place, 2012. **18**(5): p. 971-7.
67. Holstiege, J., et al., *Higher prevalence of heart failure in rural regions: a population-based study covering 87% of German inhabitants.* Clin Res Cardiol, 2019. **108**(10): p. 1102-1106.
68. Holtgrave, D.R. and R. Crosby, *Is social capital a protective factor against obesity and diabetes? Findings from an exploratory study.* Ann Epidemiol, 2006. **16**(5): p. 406-8.
69. Horsten, M., et al., *Social relations and the metabolic syndrome in middle-aged Swedish women.* J Cardiovasc Risk, 1999. **6**(6): p. 391-7.
70. Hosseini, Z., G. Veenstra, N.A. Khan, and A.I. Conklin, *Social connections and hypertension in women and men: a population-based cross-sectional study of the Canadian Longitudinal Study on Aging.* J Hypertens, 2020.
71. Hu, L., et al., *Quantile Regression Forests to Identify Determinants of Neighborhood Stroke Prevalence in 500 Cities in the USA: Implications for Neighborhoods with High Prevalence.* J Urban Health, 2020.
72. Hu, M.D., et al., *Neighborhood Deprivation, Obesity, and Diabetes in Residents of the US Gulf Coast.* Am J Epidemiol, 2021. **190**(2): p. 295-304.
73. Huang, H., *Moderating Effects of Racial Segregation on the Associations of Cardiovascular Outcomes with Walkability in Chicago Metropolitan Area.* Int J Environ Res Public Health, 2022. **19**(21).
74. Jackson, C.H., S. Richardson, and N.G. Best, *Studying place effects on health by synthesising individual and area-level outcomes.* Soc Sci Med, 2008. **67**(12): p. 1995-2006.
75. Jadow, B.M., et al., *Historical Redlining, Social Determinants of Health, and Stroke Prevalence in Communities in New York City.* JAMA Netw Open, 2023. **6**(4): p. e235875.
76. Jain, V., et al., *Association Between Social Vulnerability Index and Cardiovascular Disease: A Behavioral Risk Factor Surveillance System Study.* J Am Heart Assoc, 2022. **11**(15): p. e024414.
77. Jonsson, M., et al., *Relationship between socioeconomic status and incidence of out-of-hospital cardiac arrest is dependent on age.* J Epidemiol Community Health, 2020. **74**(9): p. 726-731.
78. Jung, L., et al., *Nationally representative household survey data for studying the interaction between district-level development and individual-level socioeconomic gradients of cardiovascular disease risk factors in India.* Data Brief, 2019. **27**: p. 104486.

79. Kauhl, B., et al., *Exploring the small-scale spatial distribution of hypertension and its association to area deprivation based on health insurance claims in Northeastern Germany.* BMC Public Health, 2018. **18**(1).
80. Keita, A.D., et al., *Associations of neighborhood area level deprivation with the metabolic syndrome and inflammation among middle- and older- age adults.* BMC Public Health, 2014. **14**: p. 1319.
81. Kelli, H.M., et al., *Association Between Living in Food Deserts and Cardiovascular Risk.* Circ Cardiovasc Qual Outcomes, 2017. **10**(9).
82. Kershaw, K.N., et al., *Metropolitan-level racial residential segregation and black-white disparities in hypertension.* Am J Epidemiol, 2011. **174**(5): p. 537-45.
83. Kolpak, P. and L. Wang, *Exploring the social and neighbourhood predictors of diabetes: a comparison between Toronto and Chicago.* Prim Health Care Res Dev, 2017. **18**(3): p. 291-299.
84. Krieger, N., *Overcoming the absence of socioeconomic data in medical records: validation and application of a census-based methodology.* Am J Public Health, 1992. **82**(5): p. 703-10.
85. Kwok, M.K., et al., *Relative Deprivation, Income Inequality, and Cardiovascular Health: Observational and Mendelian Randomization Studies in Hong Kong Chinese.* Front Public Health, 2021. **9**: p. 726617.
86. Lagisetty, P.A., et al., *Neighborhood Social Cohesion and Prevalence of Hypertension and Diabetes in a South Asian Population.* J Immigr Minor Health, 2016. **18**(6): p. 1309-1316.
87. Larrañaga, I., et al., *Socio-economic inequalities in the prevalence of Type 2 diabetes, cardiovascular risk factors and chronic diabetic complications in the Basque Country, Spain.* Diabet Med, 2005. **22**(8): p. 1047-53.
88. Lawlor, D.A., G. Davey Smith, R. Patel, and S. Ebrahim, *Life-course socioeconomic position, area deprivation, and coronary heart disease: findings from the British Women's Heart and Health Study.* Am J Public Health, 2005. **95**(1): p. 91-7.
89. Lee, D.C., et al., *Identifying Geographic Disparities in Diabetes Prevalence Among Adults and Children Using Emergency Claims Data.* J Endocr Soc, 2018. **2**(5): p. 460-470.
90. Lee, J., et al., *Geographic Variation in Morbidity and Mortality of Cerebrovascular Diseases in Korea during 2011-2015.* J Stroke Cerebrovasc Dis, 2018. **27**(3): p. 747-757.
91. Lemstra, M., C. Neudorf, and J. Opondo, *Health disparity by neighbourhood income.* Can J Public Health, 2006. **97**(6): p. 435-9.
92. Leyland, A.H., *Socioeconomic gradients in the prevalence of cardiovascular disease in Scotland: the roles of composition and context.* J Epidemiol Community Health, 2005. **59**(9): p. 799-803.
93. Li, K., M. Wen, and K.A. Henry, *Ethnic density, immigrant enclaves, and Latino health risks: A propensity score matching approach.* Soc Sci Med, 2017. **189**: p. 44-52.
94. Linde, S. and L.E. Egede, *Community Social Capital and Population Health Outcomes.* JAMA Netw Open, 2023. **6**(8): p. e2331087.
95. Loucks, E.B., et al., *Social networks and inflammatory markers in the Framingham Heart Study.* J Biosoc Sci, 2006. **38**(6): p. 835-42.
96. Lu, X., et al., *The Association Between Perceived Stress and Hypertension Among Asian Americans: Does Social Support and Social Network Make a Difference?* J Community Health, 2019. **44**(3): p. 451-462.
97. Lukachko, A., M.L. Hatzenbuehler, and K.M. Keyes, *Structural racism and myocardial infarction in the United States.* Soc Sci Med, 2014. **103**: p. 42-50.
98. Ma, R., et al., *Physical Multimorbidity and Social Participation in Adult Aged 65 Years and Older From Six Low- and Middle-Income Countries.* J Gerontol B Psychol Sci Soc Sci, 2021. **76**(7): p. 1452-1462.
99. Madela, S., et al., *Individual and area-level socioeconomic correlates of hypertension prevalence, awareness, treatment, and control in uMgungundlovu, KwaZulu-Natal, South Africa.* BMC Public Health, 2023. **23**(1): p. 417.
100. Madela, S.L.M., et al., *Area-level deprivation and individual-level socioeconomic correlates of the diabetes care cascade among black south africans in uMgungundlovu, KwaZulu-Natal, South Africa.* PLoS One, 2023. **18**(12): p. e0293250.

101. Maheswaran, R., et al., *Socio-economic deprivation and excess winter mortality and emergency hospital admissions in the South Yorkshire Coalfields Health Action Zone, UK.* Public Health, 2004. **118**(3): p. 167-76.
102. Maier, W., et al., *Area Level Deprivation Is an Independent Determinant of Prevalent Type 2 Diabetes and Obesity at the National Level in Germany. Results from the National Telephone Health Interview Surveys 'German Health Update' GEDA 2009 and 2010.* Plos One, 2014. **9**(2).
103. Malino, C., et al., *Social capital and hypertension in rural Haitian women.* Matern Child Health J, 2014. **18**(10): p. 2253-60.
104. Massa, K.H., R. Pabayo, M.L. Lebrão, and A.D. Chiavegatto Filho, *Environmental factors and cardiovascular diseases: the association of income inequality and green spaces in elderly residents of São Paulo, Brazil.* BMJ Open, 2016. **6**(9): p. e011850.
105. Matheson, F.I., et al., *Neighbourhood chronic stress and gender inequalities in hypertension among Canadian adults: a multilevel analysis.* J Epidemiol Community Health, 2010. **64**(8): p. 705-13.
106. Menec, V.H., S. Shooshtari, S. Nowicki, and S. Fournier, *Does the relationship between neighborhood socioeconomic status and health outcomes persist into very old age? A population-based study.* J Aging Health, 2010. **22**(1): p. 27-47.
107. Metcalf, P.A., et al., *Comparison of different markers of socioeconomic status with cardiovascular disease and diabetes risk factors in the Diabetes, Heart and Health Survey.* N Z Med J, 2008. **121**(1269): p. 45-56.
108. Mezuk, B., et al., *Immigrant enclaves and risk of diabetes: a prospective study.* BMC Public Health, 2014. **14**: p. 1093.
109. Mohottige, D., et al., *Residential Structural Racism and Prevalence of Chronic Health Conditions.* JAMA Netw Open, 2023. **6**(12): p. e2348914.
110. Moore, S., S. Stewart, and A. Teixeira, *Decomposing social capital inequalities in health.* J Epidemiol Community Health, 2014. **68**(3): p. 233-8.
111. Morenoff, J.D., et al., *Understanding social disparities in hypertension prevalence, awareness, treatment, and control: the role of neighborhood context.* Soc Sci Med, 2007. **65**(9): p. 1853-66.
112. Mujahid, M.S., et al., *Neighborhood characteristics and hypertension.* Epidemiology, 2008. **19**(4): p. 590-8.
113. Müller, G., et al., *Gender differences in the association of individual social class and neighbourhood unemployment rate with prevalent type 2 diabetes mellitus: a cross-sectional study from the DIAB-CORE consortium.* BMJ Open, 2013. **3**(6).
114. Müller, G., et al., *Regional and neighborhood disparities in the odds of type 2 diabetes: results from 5 population-based studies in Germany (DIAB-CORE consortium).* Am J Epidemiol, 2013. **178**(2): p. 221-30.
115. Nakagomi, A., et al., *Association Between Community-Level Social Participation and Self-reported Hypertension in Older Japanese: A JAGES Multilevel Cross-sectional Study.* Am J Hypertens, 2019. **32**(5): p. 503-514.
116. Nazmi, A., et al., *Cross-sectional and longitudinal associations of neighborhood characteristics with inflammatory markers: findings from the multi-ethnic study of atherosclerosis.* Health Place, 2010. **16**(6): p. 1104-12.
117. Neufcourt, L., et al., *Geographical variations in the prevalence of hypertension in France: Cross-sectional analysis of the CONSTANCES cohort.* Eur J Prev Cardiol, 2019. **26**(12): p. 1242-1251.
118. Ogungbe, O., et al., *Social determinants of hypertension and diabetes among African immigrants: the African immigrants health study.* Ethn Health, 2021: p. 1-13.
119. Ohanyan, H., et al., *Associations between the urban exposome and type 2 diabetes: Results from penalised regression by least absolute shrinkage and selection operator and random forest models.* Environ Int, 2022. **170**: p. 107592.
120. Oktamianti, P., et al., *District-Level Inequalities in Hypertension among Adults in Indonesia: A Cross-Sectional Analysis by Sex and Age Group.* Int J Environ Res Public Health, 2022. **19**(20).
121. Oladele, C.R., et al., *Egocentric Health Networks and Cardiovascular Risk Factors in the ECHORN Cohort Study.* J Gen Intern Med, 2020. **35**(3): p. 784-791.
122. Osborn, B., B.N. Morey, J. Billimek, and A. Ro, *Food Insecurity and Type 2 Diabetes Among Latinos: Examining Neighborhood Cohesion as a Protective Factor.* J Racial Ethn Health Disparities, 2023. **10**(4): p. 2061-2070.
123. Penninx, B.W., et al., *Social network, social support, and loneliness in older persons with different chronic diseases.* J Aging Health, 1999. **11**(2): p. 151-68.

124. Pichora, E., et al., *Comparing individual and area-based income measures: impact on analysis of inequality in smoking, obesity, and diabetes rates in Canadians 2003-2013.* Can. J. Public Health-Rev. Can. Sante Publ., 2018. **109**(3): p. 410-418.
125. Piwońska, A.M., et al., *Identifying associations between the social network index, its components, and the prevalence of cardiovascular diseases in Polish adults. Results of the cross-sectional WOBASZ II study.* Kardiol Pol, 2023. **81**(12): p. 1237-1246.
126. Ptushkina, V., et al., *Educational Level, but Not Income or Area Deprivation, is Related to Macrovascular Disease: Results From Two Population-Based Cohorts in Germany.* Int J Public Health, 2021. **66**: p. 633909.
127. Quiñones, S., A. Goyal, and Z.U. Ahmed, *Geographically weighted machine learning model for untangling spatial heterogeneity of type 2 diabetes mellitus (T2D) prevalence in the USA.* Sci Rep, 2021. **11**(1): p. 6955.
128. Rachele, J.N., B. Giles-Corti, and G. Turrell, *Neighbourhood disadvantage and self-reported type 2 diabetes, heart disease and comorbidity: a cross-sectional multilevel study.* Ann Epidemiol, 2016. **26**(2): p. 146-150.
129. Redondo-Sendino, A., P. Guallar-Castillón, J.R. Banegas, and F. Rodríguez-Artalejo, *[Relationship between social network and hypertension in older people in Spain].* Rev Esp Cardiol, 2005. **58**(11): p. 1294-301.
130. Riddell, T., *Heart failure hospitalisations and deaths in New Zealand: patterns by deprivation and ethnicity.* N Z Med J, 2004. **118**(1208): p. U1254.
131. Samuel, L.J., R.J. Thorpe, Jr., K.M. Bower, and T.A. LaVeist, *Community Characteristics are Associated with Blood Pressure Levels in a Racially Integrated Community.* J Urban Health, 2015. **92**(3): p. 403-14.
132. Sharma, I., et al., *Does the place of residence influence your risk of being hypertensive? A study-based on Nepal Demographic and Health Survey.* Hypertens Res, 2023. **46**(6): p. 1363-1374.
133. Sheets, L., et al., *The Effect of Neighborhood Disadvantage on Diabetes Prevalence.* AMIA Annu Symp Proc, 2017. **2017**: p. 1547-1553.
134. Siegel, M., A. Mielck, and W. Maier, *Individual Income, Area Deprivation, and Health: Do Income-Related Health Inequalities Vary by Small Area Deprivation?* Health Econ, 2015. **24**(11): p. 1523-30.
135. Singh, S., R. Zhou, X. Li, and L.P. Tong, *The complex relationship with health: Rural and urban "poor' women.* Int. Soc. Work, 2016. **59**(1): p. 32-46.
136. Smith, G.D., et al., *Individual social class, area-based deprivation, cardiovascular disease risk factors, and mortality: the Renfrew and Paisley Study.* J Epidemiol Community Health, 1998. **52**(6): p. 399-405.
137. Smurthwaite, K. and N. Bagheri, *Using Geographical Convergence of Obesity, Cardiovascular Disease, and Type 2 Diabetes at the Neighborhood Level to Inform Policy and Practice.* Prev Chronic Dis, 2017. **14**: p. E91.
138. Splan, E.D., A.B. Magerman, and C.E. Forbes, *Associations of regional racial attitudes with chronic illness in the United States.* Soc Sci Med, 2021. **281**: p. 114077.
139. Sun, W.J., F. Gong, and J. Xu, *Individual and contextual correlates of cardiovascular diseases among adults in the United States: a geospatial and multilevel analysis.* GeoJournal, 2020. **85**(6): p. 1685-1700.
140. Swain, P.K., B. Behera, and D. Das, *Association between Area-Level Socio-Economic Status and Hypertension in Eag States of India : An Insight from Nfhs-Iv 2015-16.* Int. J. Agric. Stat. Sci., 2019. **15**(1): p. 39-52.
141. Tang, X., et al., *Neighborhood socioeconomic status and the prevalence of stroke and coronary heart disease in rural China: a population-based study.* Int J Stroke, 2015. **10**(3): p. 388-95.
142. Tapager, I., A.M. Bender, and I. Andersen, *A decade of socioeconomic inequality in type 2 diabetes area-level prevalence: an unshakeable status quo?* Scand J Public Health, 2023. **51**(2): p. 268-274.
143. Terashima, M., D.G. Rainham, and A.R. Levy, *A small-area analysis of inequalities in chronic disease prevalence across urban and non-urban communities in the Province of Nova Scotia, Canada, 2007-2011.* BMJ Open, 2014. **4**(5): p. e004459.
144. Tompkins, J.W., I.N. Luginaah, G.L. Booth, and S.B. Harris, *The geography of diabetes in London, Canada: the need for local level policy for prevention and management.* Int J Environ Res Public Health, 2010. **7**(5): p. 2407-22.

145. Trifan, G., et al., *Association of Unfavorable Social Determinants of Health With Stroke/Transient Ischemic Attack and Vascular Risk Factors in Hispanic/Latino Adults: Results From Hispanic Community Health Study/Study of Latinos.* J Stroke, 2023. **25**(3): p. 361-370.
146. Tung, E.L., et al., *Police-Recorded Crime and Disparities in Obesity and Blood Pressure Status in Chicago.* J Am Heart Assoc, 2018. **7**(7).
147. Uddin, J., et al., *The association between neighborhood social and economic environment and prevalent diabetes in urban and rural communities: The Reasons for Geographic and Racial Differences in Stroke (REGARDS) study.* SSM Popul Health, 2022. **17**: p. 101050.
148. Usher, T., et al., *Residential Segregation and Hypertension Prevalence in Black and White Older Adults.* J Appl Gerontol, 2018. **37**(2): p. 177-202.
149. Vintimilla, R., et al., *Association of Area Deprivation Index and hypertension, diabetes, dyslipidemia, and Obesity: A Cross-Sectional Study of the HABS-HD Cohort.* Gerontol Geriatr Med, 2023. **9**: p. 23337214231182240.
150. Wagner, K.J., et al., *Effects of neighborhood socioeconomic status on blood pressure in older adults.* Rev Saude Publica, 2016. **50**: p. 78.
151. Walter, N., C. Robbins, S.T. Murphy, and S.J. Ball-Rokeach, *The weight of networks: the role of social ties and ethnic media in mitigating obesity and hypertension among Latinas.* Ethn Health, 2019. **24**(7): p. 790-803.
152. Wang, Q. and Z.L. Lan, *Park green green spaces, public health and social inequalities: Understanding the interrelationships for policy implications.* Land Use Pol., 2019. **83**: p. 66-74.
153. Wang, W., et al., *Hypertension Prevalence, Awareness, Treatment, and Control and Their Associated Socioeconomic Factors in China: A Spatial Analysis of A National Representative Survey.* Biomed Environ Sci, 2021. **34**(12): p. 937-951.
154. White, K., et al., *Racial/ethnic residential segregation and self-reported hypertension among US- and foreign-born blacks in New York City.* Am J Hypertens, 2011. **24**(8): p. 904-10.
155. Wight, R.G., et al., *A multilevel analysis of urban neighborhood socioeconomic disadvantage and health in late life.* Soc Sci Med, 2008. **66**(4): p. 862-72.
156. Williams, P.C., et al., *Perceived neighborhood social cohesion and type 2 diabetes mellitus by age, sex/gender, and race/ethnicity in the United States.* Prev Med, 2023. **170**: p. 107477.
157. Xie, H.J., et al., *Built Environment Factors Influencing Prevalence of Hypertension at Community Level in China: The Case of Wuhan.* SUSTAINABILITY, 2021. **13**(10).
158. Xu, J., et al., *Association between neighbourhood deprivation and hypertension in a US-wide Cohort.* J Epidemiol Community Health, 2022. **76**(3): p. 268-273.
159. Xu, J., et al., *Spatial scale analysis for the relationships between the built environment and cardiovascular disease based on multi-source data.* Health Place, 2023. **83**: p. 103048.
160. Yadav, R.S., et al., *Social Determinants of Stroke Hospitalization and Mortality in United States' Counties.* J Clin Med, 2022. **11**(14).
161. Yan, L.D., et al., *Neighborhood cohesion and violence in Port-au-Prince, Haiti, and their relationship to stress, depression, and hypertension: Findings from the Haiti cardiovascular disease cohort study.* PLOS Glob Public Health, 2022. **2**(7).
162. Yang, Y.C., T. Li, and Y. Ji, *Impact of social integration on metabolic functions: evidence from a nationally representative longitudinal study of US older adults.* BMC Public Health, 2013. **13**: p. 1210.
163. Yazawa, A., et al., *Association between social participation and hypertension among older people in Japan: the JAGES Study.* Hypertens. Res., 2016. **39**(11): p. 818-824.
164. Young, D.R., et al., *Associations of overweight/obesity and socioeconomic status with hypertension prevalence across racial and ethnic groups.* J Clin Hypertens (Greenwich), 2018. **20**(3): p. 532-540.
165. Yu, M.Y., A.J. Velasquez, B. Campos, and J.W. Robinette, *Perceived neighborhood disorder and type 2 diabetes disparities in Hispanic, Black, and White Americans.* Front Public Health, 2024. **12**: p. 1258348.
166. Akwo, E.A., et al., *Neighborhood Deprivation Predicts Heart Failure Risk in a Low-Income Population of Blacks and Whites in the Southeastern United States.* Circ Cardiovasc Qual Outcomes, 2018. **11**(1): p. e004052.
167. Alemi, F., et al., *Social and Medical Determinants of Diabetes: A Time-Constrained Multiple Mediator Analysis.* Cureus, 2023. **15**(9): p. e46227.

168. Altevers, J., et al., *Poor structural social support is associated with an increased risk of Type 2 diabetes mellitus: findings from the MONICA/KORA Augsburg cohort study.* Diabet Med, 2016. **33**(1): p. 47-54.
169. Anderson, K.F., E. Bjorklund, and S. Rambotti, *Income Inequality and Chronic Health Conditions: A Multilevel Analysis of the U.S. States.* Sociol. Focus, 2019. **52**(1): p. 65-85.
170. Atasoy, S., et al., *The Association of Social Connectivity and Body Weight With the Onset of Type 2 Diabetes: Findings From the Population-Based Prospective MONICA/KORA Cohort.* Psychosom Med, 2022. **84**(9): p. 1050-1055.
171. Bancks, M.P., et al., *Association of Modifiable Risk Factors in Young Adulthood With Racial Disparity in Incident Type 2 Diabetes During Middle Adulthood.* Jama, 2017. **318**(24): p. 2457-2465.
172. Barefoot, J.C., et al., *Social network diversity and risks of ischemic heart disease and total mortality: findings from the Copenhagen City Heart Study.* Am J Epidemiol, 2005. **161**(10): p. 960-7.
173. Bhavsar, N.A., et al., *Association between Gentrification and Health and Healthcare Utilization.* J Urban Health, 2022. **99**(6): p. 984-997.
174. Bilal, U., et al., *Association of neighbourhood socioeconomic status and diabetes burden using electronic health records in Madrid (Spain): the HeartHealthyHoods study.* BMJ Open, 2018. **8**(9): p. e021143.
175. Bocour, A. and M. Tria, *Preventable Hospitalization Rates and Neighborhood Poverty among New York City Residents, 2008-2013.* J Urban Health, 2016. **93**(6): p. 974-983.
176. Bray, B.D., et al., *Socioeconomic disparities in first stroke incidence, quality of care, and survival: a nationwide registry-based cohort study of 44 million adults in England.* Lancet Public Health, 2018. **3**(4): p. e185-e193.
177. Bush, K.J., et al., *Influence of neighborhood-level socioeconomic deprivation and individual socioeconomic position on risk of developing type 2 diabetes in older men: a longitudinal analysis in the British Regional Heart Study cohort.* BMJ Open Diabetes Res Care, 2023. **11**(5).
178. Carlsson, A.C., et al., *Neighbourhood socioeconomic status and coronary heart disease in individuals between 40 and 50 years.* Heart, 2016. **102**(10): p. 775-82.
179. Carlsson, A.C., et al., *Neighborhood socioeconomic status at the age of 40 years and ischemic stroke before the age of 50 years: A nationwide cohort study from Sweden.* Int J Stroke, 2017. **12**(8): p. 815-826.
180. Cené, C.W., et al., *Social Isolation and Incident Heart Failure Hospitalization in Older Women: Women's Health Initiative Study Findings.* J Am Heart Assoc, 2022. **11**(5): p. e022907.
181. Chang, S.C., et al., *Social Integration and Reduced Risk of Coronary Heart Disease in Women: The Role of Lifestyle Behaviors.* Circ Res, 2017. **120**(12): p. 1927-1937.
182. Chatzi, G., et al., *Sociodemographic disparities in non-diabetic hyperglycaemia and the transition to type 2 diabetes: evidence from the English Longitudinal Study of Ageing.* Diabet Med, 2020. **37**(9): p. 1536-1544.
183. Child, S.T., E.H. Ruppel, M.A. Albert, and L. Lawton, *Network Support and Negative Life Events Associated With Chronic Cardiometabolic Disease Outcomes.* Am J Prev Med, 2022. **62**(1): p. e21-e28.
184. Christine, P.J., et al., *Longitudinal Associations Between Neighborhood Physical and Social Environments and Incident Type 2 Diabetes Mellitus: The Multi-Ethnic Study of Atherosclerosis (MESA).* JAMA Intern Med, 2015. **175**(8): p. 1311-20.
185. Clark, C.J., et al., *Neighborhood cohesion is associated with reduced risk of stroke mortality.* Stroke, 2011. **42**(5): p. 1212-7.
186. Claudel, S.E., et al., *Association between neighborhood-level socioeconomic deprivation and incident hypertension: A longitudinal analysis of data from the Dallas heart study.* Am Heart J, 2018. **204**: p. 109-118.
187. Cozier, Y.C., et al., *Relation between neighborhood median housing value and hypertension risk among black women in the United States.* Am J Public Health, 2007. **97**(4): p. 718-24.
188. Cuthbertson, C.C., et al., *Socioeconomic status and access to care and the incidence of a heart failure diagnosis in the inpatient and outpatient settings.* Ann Epidemiol, 2018. **28**(6): p. 350-355.

189. Diez Roux, A.V., et al., *Socioeconomic disadvantage and change in blood pressure associated with aging.* Circulation, 2002. **106**(6): p. 703-10.
190. Eng, P.M., E.B. Rimm, G. Fitzmaurice, and I. Kawachi, *Social ties and change in social ties in relation to subsequent total and cause-specific mortality and coronary heart disease incidence in men.* Am J Epidemiol, 2002. **155**(8): p. 700-9.
191. Essien, U.R., et al., *Association Between Neighborhood-Level Poverty and Incident Atrial Fibrillation: a Retrospective Cohort Study.* J Gen Intern Med, 2022. **37**(6): p. 1436-1443.
192. Exeter, D.J., et al., *Movers and stayers: The geography of residential mobility and CVD hospitalisations in Auckland, New Zealand.* Soc Sci Med, 2015. **133**: p. 331-9.
193. Forsberg, P.O., H. Ohlsson, and K. Sundquist, *Workplace socioeconomic characteristics and coronary heart disease: a nationwide follow-up study.* BMJ Open, 2023. **13**(7): p. e065285.
194. Forsberg, P.O., H. Ohlsson, and K. Sundquist, *Causal nature of neighborhood deprivation on individual risk of coronary heart disease or ischemic stroke: A prospective national Swedish co-relative control study in men and women.* Health Place, 2018. **50**: p. 1-5.
195. Freedman, V.A., I.B. Grafova, and J. Rogowski, *Neighborhoods and chronic disease onset in later life.* Am J Public Health, 2011. **101**(1): p. 79-86.
196. Gao, X., et al., *Associations Between Residential Segregation and Incident Hypertension: The Multi-Ethnic Study of Atherosclerosis.* J Am Heart Assoc, 2022. **11**(3): p. e023084.
197. Garcia, L., et al., *The Impact of Neighborhood Socioeconomic Position on Prevalence of Diabetes and Prediabetes in Older Latinos: The Sacramento Area Latino Study on Aging.* Hisp Health Care Int, 2015. **13**(2): p. 77-85.
198. Garcia, L., et al., *Influence of neighbourhood socioeconomic position on the transition to type II diabetes in older Mexican Americans: the Sacramento Area Longitudinal Study on Aging.* BMJ Open, 2016. **6**(8): p. e010905.
199. Gebreab, S.Y., et al., *Neighborhood social and physical environments and type 2 diabetes mellitus in African Americans: The Jackson Heart Study.* Health Place, 2017. **43**: p. 128-137.
200. Gero, K., et al., *Associations of state-level and county-level hate crimes with individual-level cardiovascular risk factors in a prospective cohort study of middle-aged Americans: the National Longitudinal Survey of Youths 1979.* BMJ Open, 2022. **12**(1): p. e054360.
201. Guion, M., et al., *Eleven-year trends in socioeconomic inequalities in the prevalence and incidence of pharmacologically treated type 2 diabetes in France, 2010-2020.* Diabetes Metab, 2024. **50**(2): p. 101509.
202. Gwon, J.G., J. Choi, and Y.J. Han, *Community-level socioeconomic inequality in the incidence of ischemic heart disease: a nationwide cohort study.* BMC Cardiovasc Disord, 2020. **20**(1): p. 87.
203. Halonen, J.I., et al., *Childhood Psychosocial Adversity and Adult Neighborhood Disadvantage as Predictors of Cardiovascular Disease: A Cohort Study.* Circulation, 2015. **132**(5): p. 371-9.
204. Hamad, R., et al., *Association of Neighborhood Disadvantage With Cardiovascular Risk Factors and Events Among Refugees in Denmark.* JAMA Netw Open, 2020. **3**(8): p. e2014196.
205. Hanefeld, C., et al., *Social Gradients in Myocardial Infarction and Stroke Diagnoses in Emergency Medicine.* Dtsch Arztebl Int, 2018. **115**(4): p. 41-48.
206. Harding, B.N., et al., *Relationship between social support and incident hypertension in the Jackson Heart Study: a cohort study.* BMJ Open, 2022. **12**(3): p. e054812.
207. Hassen, H.Y., H. Bastiaens, K. Van Royen, and S. Abrams, *Socioeconomic and behavioral determinants of cardiovascular diseases among older adults in Belgium and France: A longitudinal analysis from the SHARE study.* PLoS One, 2020. **15**(12): p. e0243422.
208. Heeley, E.L., et al., *Socioeconomic disparities in stroke rates and outcome: pooled analysis of stroke incidence studies in Australia and New Zealand.* Med J Aust, 2011. **195**(1): p. 10-4.
209. Hendryx, M., et al., *Social Relationships and Risk of Type 2 Diabetes Among Postmenopausal Women.* J Gerontol B Psychol Sci Soc Sci, 2020. **75**(7): p. 1597-1608.
210. Henriksson, G., G.R. Weitoft, and P. Allebeck, *Associations between income inequality at municipality level and health depend on context - a multilevel analysis on myocardial infarction in Sweden.* Soc Sci Med, 2010. **71**(6): p. 1141-9.

211. Herrera-Añazco, P., et al., *Association between social determinants of health and trends in prevalence of hypertension in patients of the Peruvian Ministry of Health.* Trop Med Int Health, 2019. **24**(12): p. 1434-1441.
212. Hilding, A., C. Shen, and C.G. Östenson, *Social network and development of prediabetes and type 2 diabetes in middle-aged Swedish women and men.* Diabetes Res Clin Pract, 2015. **107**(1): p. 166-77.
213. Hill, P.L., S.J. Weston, and J.J. Jackson, *Connecting social environment variables to the onset of major specific health outcomes.* Psychol Health, 2014. **29**(7): p. 753-67.
214. Honda, Y., et al., *Psychosocial factors and subsequent risk of hospitalizations with peripheral artery disease: The Atherosclerosis Risk in Communities (ARIC) Study.* Atherosclerosis, 2021. **329**: p. 36-43.
215. Honjo, K., et al., *Impact of neighborhood socioeconomic conditions on the risk of stroke in Japan.* J Epidemiol, 2015. **25**(3): p. 254-60.
216. Howard, V.J., et al., *Neighborhood socioeconomic index and stroke incidence in a national cohort of blacks and whites.* Neurology, 2016. **87**(22): p. 2340-2347.
217. Hwang, S.E., et al., *Association between social trust and the risk of cardiovascular disease in older adults in Korea: a nationwide retrospective cohort study.* BMC Public Health, 2020. **20**(1): p. 1844.
218. Jack, E., D. Lee, and N. Dean, *Estimating the changing nature of Scotland's health inequalities by using a multivariate spatiotemporal model.* J R Stat Soc Ser A Stat Soc, 2019. **182**(3): p. 1061-1080.
219. Kaiser, P., et al., *Neighborhood Environments and Incident Hypertension in the Multi-Ethnic Study of Atherosclerosis.* Am J Epidemiol, 2016. **183**(11): p. 988-97.
220. Kakinami, L., et al., *Neighbourhood disadvantage and behavioural problems during childhood and the risk of cardiovascular disease risk factors and events from a prospective cohort.* Prev Med Rep, 2017. **8**: p. 294-300.
221. Kawachi, I., et al., *A prospective study of social networks in relation to total mortality and cardiovascular disease in men in the USA.* J Epidemiol Community Health, 1996. **50**(3): p. 245-51.
222. Kim, Y., A. Lee, and C. Cubbin, *Effect of Social Environments on Cardiovascular Disease in the United States.* J Am Heart Assoc, 2022. **11**(20): p. e025923.
223. Kivimäki, M., et al., *Neighbourhood socioeconomic disadvantage, risk factors, and diabetes from childhood to middle age in the Young Finns Study: a cohort study.* Lancet Public Health, 2018. **3**(8): p. e365-e373.
224. Kivimäki, M., et al., *Modifications to residential neighbourhood characteristics and risk of 79 common health conditions: a prospective cohort study.* Lancet Public Health, 2021. **6**(6): p. e396-e407.
225. Krishnan, S., Y.C. Cozier, L. Rosenberg, and J.R. Palmer, *Socioeconomic status and incidence of type 2 diabetes: results from the Black Women's Health Study.* Am J Epidemiol, 2010. **171**(5): p. 564-70.
226. Lachkhem, Y., É. Minvielle, and S. Rican, *Geographic Variations of Stroke Hospitalization across France: A Diachronic Cluster Analysis.* Stroke Res Treat, 2018. **2018**: p. 1897569.
227. Laursen, K.R., A. Hulman, D.R. Witte, and H. Terkildsen Maindal, *Social relations, depressive symptoms, and incident type 2 diabetes mellitus: The English Longitudinal Study of Ageing.* Diabetes Res Clin Pract, 2017. **126**: p. 86-94.
228. Ling, D.C., *Do the Chinese "Keep up with the Jones"?: Implications of peer effects, growing economic disparities and relative deprivation on health outcomes among older adults in China.* China Econ. Rev., 2009. **20**(1): p. 65-81.
229. Lippert, A.M., C.R. Evans, F. Razak, and S.V. Subramanian, *Associations of Continuity and Change in Early Neighborhood Poverty With Adult Cardiometabolic Biomarkers in the United States: Results From the National Longitudinal Study of Adolescent to Adult Health, 1995-2008.* Am J Epidemiol, 2017. **185**(9): p. 765-776.
230. Lönn, S.L., O. Melander, C. Crump, and K. Sundquist, *Accumulated neighbourhood deprivation and coronary heart disease: a nationwide cohort study from Sweden.* BMJ Open, 2019. **9**(9): p. e029248.
231. Lukaschek, K., et al., *Sex differences in the association of social network satisfaction and the risk for type 2 diabetes.* BMC Public Health, 2017. **17**(1): p. 379.

232. Lund, R., N.H. Rod, and U. Christensen, *Are negative aspects of social relations predictive of angina pectoris? A 6-year follow-up study of middle-aged Danish women and men.* J Epidemiol Community Health, 2012. **66**(4): p. 359-65.
233. Lund, R., et al., *Negative aspects of close social relations and 10-year incident ischaemic heart disease hospitalization among middle-aged Danes.* Eur J Prev Cardiol, 2014. **21**(10): p. 1249-56.
234. Marley, T.L. and M.W. Metzger, *A longitudinal study of structural risk factors for obesity and diabetes among American Indian young adults, 1994-2008.* Prev Chronic Dis, 2015. **12**: p. E69.
235. Matthew, P. and D.M. Brodersen, *Income inequality and health outcomes in the United States: An empirical analysis.* Soc. Sci. J., 2018. **55**(4): p. 432-442.
236. Mayne, S.L., et al., *Racial residential segregation, racial discrimination, and diabetes: The Coronary Artery Risk Development in Young Adults study.* Health Place, 2020. **62**: p. 102286.
237. McDoom, M.M., et al., *Late life socioeconomic status and hypertension in an aging cohort: the Atherosclerosis Risk in Communities Study.* J Hypertens, 2018. **36**(6): p. 1382-1390.
238. Mentias, A., et al., *Historical Redlining, Socioeconomic Distress, and Risk of Heart Failure Among Medicare Beneficiaries.* Circulation, 2023. **148**(3): p. 210-219.
239. Morris, R.W., et al., *Do socioeconomic characteristics of neighbourhood of residence independently influence incidence of coronary heart disease and all-cause mortality in older British men?* Eur J Cardiovasc Prev Rehabil, 2008. **15**(1): p. 19-25.
240. Murray, E.T., et al., *Trajectories of neighborhood poverty and associations with subclinical atherosclerosis and associated risk factors: the multi-ethnic study of atherosclerosis.* Am J Epidemiol, 2010. **171**(10): p. 1099-108.
241. Nikulina, V. and C.S. Widom, *Do race, neglect, and childhood poverty predict physical health in adulthood? A multilevel prospective analysis.* Child Abuse Negl, 2014. **38**(3): p. 414-24.
242. Odoi, E.W., et al., *Sociodemographic Determinants of Acute Myocardial Infarction Hospitalization Risks in Florida.* J Am Heart Assoc, 2020. **9**(11): p. e012712.
243. Omariba, D.W., N.A. Ross, C. Sanmartin, and J.V. Tu, *Neighbourhood immigrant concentration and hospitalization: a multilevel analysis of cardiovascular-related admissions in Ontario using linked data.* Can J Public Health, 2014. **105**(6): p. e404-11.
244. Pantell, M.S., et al., *Association of Social and Behavioral Risk Factors With Earlier Onset of Adult Hypertension and Diabetes.* JAMA Netw Open, 2019. **2**(5): p. e193933.
245. Pinheiro, L.C., et al., *Multiple Vulnerabilities to Health Disparities and Incident Heart Failure Hospitalization in the REGARDS Study.* Circ Cardiovasc Qual Outcomes, 2020. **13**(8): p. e006438.
246. Quashie, N.T., et al., *Neighborhood socioeconomic position, living arrangements, and cardiometabolic disease among older Puerto Ricans: An examination using PREHCO 2002-2007.* PLoS One, 2023. **18**(8): p. e0289170.
247. Rethy, L.B., et al., *Neighborhood Poverty and Incident Heart Failure: an Analysis of Electronic Health Records from 2005 to 2018.* J Gen Intern Med, 2021. **36**(12): p. 3719-3727.
248. Rod, N.H., I. Andersen, and E. Prescott, *Psychosocial risk factors and heart failure hospitalization: a prospective cohort study.* Am J Epidemiol, 2011. **174**(6): p. 672-80.
249. Rose, K.M., et al., *Neighborhood disparities in incident hospitalized myocardial infarction in four U.S. communities: the ARIC surveillance study.* Ann Epidemiol, 2009. **19**(12): p. 867-74.
250. Safford, M.M., et al., *Number of Social Determinants of Health and Fatal and Nonfatal Incident Coronary Heart Disease in the REGARDS Study.* Circulation, 2021. **143**(3): p. 244-253.
251. Salinas, J., et al., *Associations between social relationship measures, serum brain-derived neurotrophic factor, and risk of stroke and dementia.* Alzheimers Dement (N Y), 2017. **3**(2): p. 229-237.

252. Savin, K.L., et al., *Social and built neighborhood environments and blood pressure 6 years later: Results from the Hispanic Community Health Study/Study of Latinos and the SOL CASAS ancillary study.* Soc Sci Med, 2022. **292**: p. 114496.
253. Schieb, L.J., L.R. Mobley, M. George, and M. Casper, *Tracking stroke hospitalization clusters over time and associations with county-level socioeconomic and healthcare characteristics.* Stroke, 2013. **44**(1): p. 146-52.
254. Schootman, M., et al., *The effect of adverse housing and neighborhood conditions on the development of diabetes mellitus among middle-aged African Americans.* American Journal of Epidemiology, 2007. **166**(4): p. 379-387.
255. Schwartz, B.S., et al., *Association of community types and features in a case-control analysis of new onset type 2 diabetes across a diverse geography in Pennsylvania.* BMJ Open, 2021. **11**(1): p. e043528.
256. Sharp, G. and R.M. Carpiano, *Neighborhood social organization exposures and racial/ethnic disparities in hypertension risk in Los Angeles.* PLoS One, 2023. **18**(3): p. e0282648.
257. Shibayama, T., H. Noguchi, H. Takahashi, and N. Tamiya, *Relationship between social engagement and diabetes incidence in a middle-aged population: Results from a longitudinal nationwide survey in Japan.* J Diabetes Investig, 2018. **9**(5): p. 1060-1066.
258. Steckel, R.H., *The hidden cost of moving up: type 2 diabetes and the escape from persistent poverty in the American South.* Am J Hum Biol, 2013. **25**(4): p. 508-15.
259. Suchy-Dicey, A., et al., *Psychological and social support associations with mortality and cardiovascular disease in middle-aged American Indians: the Strong Heart Study.* Soc Psychiatry Psychiatr Epidemiol, 2022. **57**(7): p. 1421-1433.
260. Sundquist, K., M. Malmström, and S.E. Johansson, *Neighbourhood deprivation and incidence of coronary heart disease: a multilevel study of 2.6 million women and men in Sweden.* J Epidemiol Community Health, 2004. **58**(1): p. 71-7.
261. Thrift, A.G., et al., *Greater incidence of both fatal and nonfatal strokes in disadvantaged areas: the Northeast Melbourne Stroke Incidence Study.* Stroke, 2006. **37**(3): p. 877-82.
262. Tung, E.L., et al., *Association of Rising Violent Crime With Blood Pressure and Cardiovascular Risk: Longitudinal Evidence From Chicago, 2014-2016.* Am J Hypertens, 2019. **32**(12): p. 1192-1198.
263. Uddin, J., et al., *Age and sex differences in the association between neighborhood socioeconomic environment and incident diabetes: Results from the diabetes location, environmental attributes and disparities (LEAD) network.* SSM Popul Health, 2023. **24**: p. 101541.
264. Vart, P., et al., *Socioeconomic Status and Incidence of Hospitalization With Lower-Extremity Peripheral Artery Disease: Atherosclerosis Risk in Communities Study.* J Am Heart Assoc, 2017. **6**(8).
265. Villani, M., et al., *Geographical variation of diabetic emergencies attended by prehospital Emergency Medical Services is associated with measures of ethnicity and socioeconomic status.* Sci Rep, 2018. **8**(1): p. 5122.
266. Vogt, T.M., et al., *Social networks as predictors of ischemic heart disease, cancer, stroke and hypertension: incidence, survival and mortality.* J Clin Epidemiol, 1992. **45**(6): p. 659-66.
267. Xiao, Q., et al., *Life-Course Neighborhood Socioeconomic Status and Cardiovascular Events in Black and White Adults in the Atherosclerosis Risk in Communities Study.* Am J Epidemiol, 2022. **191**(8): p. 1470-1484.
268. Yan, T., et al., *Exploring psychosocial pathways between neighbourhood characteristics and stroke in older adults: the cardiovascular health study.* Age Ageing, 2013. **42**(3): p. 391-7.
269. Yang, Y.C., C. Boen, and K. Mullan Harris, *Social relationships and hypertension in late life: evidence from a nationally representative longitudinal study of older adults.* J Aging Health, 2015. **27**(3): p. 403-31.
270. Yang, Y.C., et al., *Social relationships and physiological determinants of longevity across the human life span.* Proc Natl Acad Sci U S A, 2016. **113**(3): p. 578-83.
271. Spicer, J., R. Jackson, and R. Scragg, *The Effects of Anger Management and Social Contact on Risk of Myocardial-Infarction in Type-as and Type-Bs.* Psychol. Health, 1993. **8**(4): p. 243-255.

272. Welin, C.L., A. Rosengren, and L.W. Wilhelmsen, *Social relationships and myocardial infarction: a case-control study.* J Cardiovasc Risk, 1996. **3**(2): p. 183-90.
273. Cheruvalath, H., et al., *Associations Between Residential Greenspace, Socioeconomic Status, and Stroke: A Matched Case-Control Study.* J Patient Cent Res Rev, 2022. **9**(2): p. 89-97.
274. Schwartz, B.S., et al., *Associations of four indexes of social determinants of health and two community typologies with new onset type 2 diabetes across a diverse geography in Pennsylvania.* PLoS One, 2022. **17**(9): p. e0274758.
275. Yadav, S., S. Garg, and A.V. Raut, *Evaluation of association of psychosocial stress and hypertension in adults >30 years of age: A community-based case-control study from Rural Central India.* INTERNATIONAL JOURNAL OF NONCOMMUNICABLE DISEASES, 2021. **6**(3): p. 142-148.
276. Ludwig, J., et al., *Neighborhoods, obesity, and diabetes--a randomized social experiment.* N Engl J Med, 2011. **365**(16): p. 1509-19.
277. Kling, J.R., J.B. Liebman, and L.F. Katz, *Experimental analysis of neighborhood effects.* Econometrica, 2007. **75**(1): p. 83-119.
278. White, J.S., et al., *Long-term effects of neighbourhood deprivation on diabetes risk: quasi-experimental evidence from a refugee dispersal policy in Sweden.* Lancet Diabetes Endocrinol, 2016. **4**(6): p. 517-24.
279. Kim, D., et al., *Neighbourhood socioeconomic position and risks of major chronic diseases and all-cause mortality: a quasi-experimental study.* BMJ Open, 2018. **8**(5): p. e018793.
280. Jensen, N.K., et al., *The association of neighborhood socioeconomic characteristics with cardiovascular health: A quasi-experimental study of refugees to Denmark.* Health Place, 2023. **84**: p. 103128.
281. Kim, M.H., et al., *School racial segregation and long-term cardiovascular health among Black adults in the US: A quasi-experimental study.* PLoS Med, 2022. **19**(6): p. e1004031.
