# Supplementary material for: Associations between dimensions of the social environment and cardiometabolic health outcomes: a systematic review and meta-analysis
Source: BMJ Open. 2024 Aug 28;14(8):e079987. doi: 10.1136/bmjopen-2023-079987 (PMC11367359; doi:10.1136/bmjopen-2023-079987)
Supplement: online supplemental file 9 [file bmjopen-14-8-s009.pdf]

Supplementary Table 4. Summary pooled effects and between-study variance estimates and 95% confidence intervals from meta-analysis models (middle-income countries only)

Exposure	Outcome	n	Odds ratio	95% CI		Total I^2
Economic and Social Disadvantage	*Hypertensive diseases*	7	1.11	0.63 - 1.96		100%
Economic and Social Disadvantage	*Diabetes mellitus*	5	0.77	0.53 - 1.13		96%
Economic and Social Disadvantage	*Unspecified cardiovascular diseases*	4	1.46	0.74 - 2.88		88%

'**' = p <= 0.01 ; '*' = p <= 0.05

Supplementary Table 5. Summary pooled effects and between-study variance estimates and 95% confidence intervals from meta-analysis models (longitudinal studies only)

Exposure	Outcome	n	Odds ratio	95% CI		Total I^2
Economic and Social Disadvantage	*Hypertensive diseases*	11	**1.12**	**1.03 - 1.23**		64%
Economic and Social Disadvantage	*Diabetes mellitus*	10	**1.16**	**1.04 - 1.30**		69%
Economic and Social Disadvantage	*Unspecified cardiovascular diseases*	4	1.01	0.91 - 1.12		46%
Economic and Social Disadvantage	*Ischaemic heart diseases*	8	1.04	0.94 - 1.14		16%
Economic and Social Disadvantage	*Heart failure*	11	1.11	0.96 - 1.28		84%
Economic and Social Disadvantage	*Stroke*	18	1.14	0.92 - 1.42		80%
Social Relationships and Norms	*Hypertensive diseases*	11	1.15	0.90 - 1.48		87%
Social Relationships and Norms	*Diabetes mellitus*	3	1.07	0.85 - 1.34		30%
Social Relationships and Norms	*Unspecified cardiovascular diseases*	4	1.30	0.82 - 2.05		94%
Social Relationships and Norms	*Ischaemic heart diseases*	12	**1.30**	**1.05 - 1.62**	*	86%
Social Relationships and Norms	*Stroke*	8	1.17	0.67 - 2.04		73%

'**' = p <= 0.01 ; '*' = p <= 0.05

Supplementary Table 6. Summary pooled effects and between-study variance estimates and 95% confidence intervals from meta-analysis models (odds ratio only)

Exposure	Outcome	n	Odds ratio	95% CI	Total I^2
Economic and Social Disadvantage	*Hypertensive diseases*	16	1.26	0.98 - 1.63	100%
Economic and Social Disadvantage	*Diabetes mellitus*	19	1.17	0.97 - 1.42	97%
Economic and Social Disadvantage	*Unspecified cardiovascular diseases*	11	1.36	0.98 - 1.87	96%
Economic and Social Disadvantage	*Ischaemic heart diseases*	6	1.44	0.72 - 2.88	79%
Economic and Social Disadvantage	*Heart failure*	5	1.15	0.69 - 1.92	85%
Social Relationships and Norms	*Hypertensive diseases*	7	1.26	0.79 - 1.99	81%
Social Relationships and Norms	*Unspecified cardiovascular diseases*	3	1.32	0.51 - 3.45	86%
Civic Participation and Engagement	*Hypertensive diseases*	3	1.41	0.90 - 2.21	0%
Crime and Safety	*Hypertensive diseases*	3	1.17	0.56 - 2.44	0%

'**' = p <= 0.01 ; '*' = p <= 0.05
