# Supplementary material for: Associations between dimensions of the social environment and cardiometabolic health outcomes: a systematic review and meta-analysis
Source: BMJ Open. 2024 Aug 28;14(8):e079987. doi: 10.1136/bmjopen-2023-079987 (PMC11367359; doi:10.1136/bmjopen-2023-079987)
Supplement: Supplementary file 10 [file bmjopen-2023-079987.reviewer_comments.pdf]

PEER REVIEW HISTORY

BMJ Open publishes all reviews undertaken for accepted manuscripts. Reviewers are asked to complete a checklist review form and are provided with free text boxes to elaborate on their assessment. These free text comments are reproduced below.

ARTICLE DETAILS

**TITLE (PROVISIONAL)**	Associations between dimensions of the social environment and cardiometabolic health outcomes: systematic review and meta-analysis
**AUTHORS**	Abreu, Taymara; Beulens, Joline; Heuvelman, Fleur; Schoonmade, Linda; Mackenbach, Joreintje

VERSION 1 - REVIEW

**REVIEWER NAME**	Braune, Tanya
**REVIEWER AFFILIATION**	University of Cambridge, MRC Epidemiology Unit
**REVIEWER CONFLICT OF INTEREST**	There are no known competing interests
**DATE REVIEW RETURNED**	09-Feb-2024

**GENERAL COMMENTS**	This review is very comprehensive and well written. Please see some comments below on some suggestions to improve clarity on the methods for reproducibility and additional points for the discussion:<br>1. You might want to consider updating the search as it is 3 years old, particularly that you have mentioned that the majority of studies included were published in the last 10 years. This may provide more insights into the less studied dimensions of the social environment.<br>2. Introduction - you could introduce the conceptual framework you used in the introduction and why this is a useful framework but you should discuss your choice to divide the social environment into the 'dimensions' in the methods. It may be useful to define each dimension with an example attached as well, you did this for two of the dimensions in the introduction but it would be good to see it for the others as well.<br>3. In the methods, it would be useful to describe how you went about categorising your exposures into the eight dimensions and your rationale for this.<br>4. Line 22 on page 7 – XXXX et al. – I think this reference may be missing.<br>5. Could provide more detail on agreement across reviewers for the study selection.<br>6. Qualitative studies are only mentioned once in the results - clarify whether these were included but there were none, or whether they were excluded from the start. This could be added to the methods inclusion/exclusion criteria.<br>7. Sex-specific effects – could you include more detail on the reason for looking at these – why is it useful? Currently, you are mentioning in the methods that you did it when data was available but it is not clear why you did this.<br>8. In the discussion you mention the commonly observed outcomes – it would be helpful to see what are the % and n of studies that looked at these most common outcomes.

	9. Throughout, you talk about ‘worse’ social environments – it would be good to see this defined somewhere, what you consider to be a worse social environment and include some examples.<br>10. Discussion: You have mixed the strengths and limitations of your included studies with the strengths and limitations of your methodology. For example, in the paragraph about your study’s strengths and limitations, you mention that the majority of studies were conducted in high income countries – was this a limitation of your review or of the evidence? Was this a limitation due to your imposed exclusion criteria or the available evidence? It may be useful to clearly distinguish these in different paragraphs in your discussion.<br>11. It would be helpful to add some more detail in the discussion about the implications of your research in addition to your suggestions for future research.

**REVIEWER NAME**	Idema, Demy
**REVIEWER AFFILIATION**	University Medical Centre Utrecht, Julius Center for Health Sciences and Primary Care
**REVIEWER CONFLICT OF INTEREST**	None
**DATE REVIEW RETURNED**	16-Feb-2024

**GENERAL COMMENTS**	First, we would like to congratulate the authors on the extensive work they have done on this interesting subject. We have some comments that we have divided into the sections of the manuscript that they apply to.<br><br>Second, I would like to mention that I have performed this peer-review together with my colleague Linde Huis in ‘t Veld.<br><br>General remarks<br>1) The search was performed in February 2021, and as the authors stated themselves, research on some of the social environment dimensions is only recently emerging, so it might be worthwhile to update the search before publication.<br><br>2) We think it would be good to add an appendix with the list of studies that were excluded during full text screening with reasons for exclusion.<br><br>3) There is no mention of assessment of publication bias, or why assessment would not be possible.<br><br>4) Have you considered doing a certainty assessment for the investigated associations, for example with GRADE?<br><br>Abstract<br>5) Page 3, line 18: Starting the methods section with “we performed a systematic review and meta-analysis…” seems to be double considering the design mentioned above.<br><br>6) The order in which the methods are presented in the abstract are not in line with the methods section. It would make more sense to mention quality assessment after data extraction, and there is no mention of full text screening in the abstract.<br><br>7) Page 3, line 43: In the sentence “Meta-analyzed sex-specific

associations consistently showed results in the same direction.", it is unclear to us whether you mean the same direction as each other (i.e. the same direction for both sexes) or the same direction as the main effect (or both).

Strengths and limitations of this study
8) Page 4, line 12: Would it be possible to elaborate on this sentence to make it clearer: "The broad scope of the search strategy helps to mitigate the impact of heterogeneity on social environmental factors on the retrieval and synthesizes of findings;" Wouldn't a narrower search where you identify factors that are more similar yield an analysis with less heterogeneity?

Introduction
9) Page 5, line 16: You mention that there is a reduction in the burden of CMD in high-income countries and a disproportionate burden in low- and middle- income countries, could you elaborate on why you decided to not specifically focus on low- and middle-income countries and rather included all settings?

Methods
10) Page 7, line 20: You mention that you divided the results into references with 'hard outcomes' and 'risk factors' as outcomes, but from this part of the text it is not yet clear what exactly you mean with risk factors. The term in this context is a bit confusing with the risk factors mentioned in the introduction at the environmental level (i.e. the social environment dimensions).

11) Page 7, line 48: You mention that you use the mobile app Covidence to 'expedite the screening process', could you explain what you mean by this? Did you only use Covidence for systematic review management or also used any machine learning tools in the screening process?

12) Page 8, line 14: You have explained that you do not include CMD with little or no influence of lifestyle behaviors, but we miss an explanation for why you have not included non-alcoholic fatty liver disease, which seems to us as a type of CMD that does have lifestyle influence. Could you elaborate?

13) Page 8, line 41 and Page 9, line 5: Data extraction and quality assessment was performed by one author, with a second author checking a random 10% sample, but what was the agreement (e.g. kappa score) between the two reviewers?

14) Page 9, line 45: You state that low quality studies (that got <50% of available points) were excluded from the vote count and meta-analyses, but why did you choose this value in particular? And have you considered including all studies but doing a sensitivity analysis without all the low-quality (or high risk of bias) studies excluded?

15) Are the included effect measures from the primary studies adjusted for any variables? Or all they all from univariate analyses?

Results
16) Page 12, line 43 and Page 13, line 10: You don't mention that vote counting was not possible for associations with the social environment dimension 'Discrimination and segregation'. You explain why in the discussion, but without any disclaimer in the results, the statement that "all social environment dimensions were

	found […] except for Sense of Place/Belonging" is not true.<br><br>17) Page 13, line 10: Same as comment 16 above.<br><br>18) Page 13, line 20: "We observed in all 14 meta-analyses that worse social environment was associated with increased risk of CMD outcomes". We think you could be more careful in making this statement when there is no statistical significance for 10 of these results.<br><br>19) We also wonder whether you have considered if the statistically significant results are clinically significant? Have you tried identifying a minimally clinically important difference for example?<br><br>20) Page 13, line 39: (Almost) all meta-analyses have very high heterogeneity, have you considered to not pool the data and present it in another way? Or maybe explore the heterogeneity a bit more with subgroup analyses or meta-regression?<br><br>Discussion<br>21) Page 15, line 28: In the third paragraph you go back to the main finding of the study, which may fit better at the start of the discussion.<br><br>22) Page 15, line 43: "One could argue that living in a deprived neighborhood or a lack of social relationships leads to stress, rising blood pressure and hypertensive diseases "directly" – resulting in larger effect sizes - whereas the level of civic participation may lead to stress via reduced social cohesion only – resulting in smaller effect sizes. However, we found no indication for this explanation." We were curious whether you have found this effect to hold true in other literature?<br><br>23) Page 17, line 9: In the discussion you mention that it may be better to use longitudinal studies to study the effect of cumulative exposure; have you considered performing a sensitivity analysis for only the longitudinal studies to see if this affects your outcome?<br><br>Table 1<br>24) In the characteristics of studies table there is a lot of 'not applicable' filled in for average age and % women for example, but it seems to us that you mean 'not reported'?

VERSION 1 – AUTHOR RESPONSE

Reviewer: 1

Dr. Tanya Braune, University of Cambridge

Comments to the Author:

This review is very comprehensive and well written. Please see some comments below on some suggestions to improve clarity on the methods for reproducibility and additional points for the discussion:

We thank the reviewer for taking the time to evaluate our systematic review and meta-analysis. We appreciate the constructive feedback provided to improve the manuscript. We have carefully considered and responded to the reviewer’s comments in detail below (written in blue).

As you and the other reviewer rightly indicated, this is a quickly developing field and literature reviews are especially time-sensitive in nature. We have therefore conducted an extensive update of the literature review, yielding 73 additional studies to be included. We hope that the revisions we made to the manuscript are satisfactorily and allow for a swift publication of the manuscript.

1. You might want to consider updating the search as it is 3 years old, particularly that you have mentioned that the majority of studies included were published in the last 10 years. This may provide more insights into the less studied dimensions of the social environment.

We acknowledge the importance of staying up-to-date with the latest research in the field and agree with the reviewer’s comments. Therefore, we have updated the study and performed a new search in February 28th 2024.

Changes related to the update search were made throughout the manuscript and the update review process is visually depicted in the PRISMA flowchart.

We highlight that while 73 new articles were added to the review, in general, we did not find different trends in terms of associations explored, direction of the associations, methodological quality and geographical distribution of studies. Thus, the update of the review resulting in the addition of new studies did not impact our overall conclusions.

2. Introduction - you could introduce the conceptual framework you used in the introduction and why this is a useful framework but you should discuss your choice to divide the social environment into the ‘dimensions’ in the methods. It may be useful to define each dimension with an example attached as well, you did this for two of the dimensions in the introduction but it would be good to see it for the others as well.

We thank the reviewer for the suggestion. We present the categorization of the social environment into the ‘dimensions’ in the methods section instead of the introduction section. Also, besides some examples already provided, we now provide an example for each social environmental dimension. See the text added below:

“We categorized social environment factors into one of the eight dimensions depicted in Figure 1, i.e., Economic and Social Disadvantage includes for instance area-level poverty and deprivation, Social Relationships and Norms includes support from the social network, Crime and Safety includes crimes per capita, Social Cohesion and Social Capital includes neighborhood collective efficacy and social trust, Disorders and Incivilities includes neighborhood disorder, Civic Participation and Engagement includes participation in social activities, and Discrimination and Segregation includes percentage of migrants in an area.”

3. In the methods, it would be useful to describe how you went about categorising your exposures into the eight dimensions and your rationale for this.

Thank you or the opportunity to clarify that. We categorized the exposures of the included studies into one of the eight different social environmental dimensions (see conceptual framework) based on the terminology adopted by the authors of the included studies. In very few occasions the classification procedure based on the terminology was not straightforward. We then based our decision on additional information provided by the authors (e.g., assessment method) and add the exposure variable to dimensions of similar characteristics. In order to clarify that in the manuscript, we added the text below:

“Exposures were classified into one of the eight dimensions following the terminology employed by the included studies. When a exposure variable could not be clearly assigned to a category, we classified it based on the variable measurement and operationalization.”

4. Line 22 on page 7 – XXXX et al. – I think this reference may be missing.

Thank you for noting that. We have provided the appropriate reference. This citation refers to the twin review also written by our team and was anonymized for the purpose of submission.

5. Could provide more detail on agreement across reviewers for the study selection.

We appreciate your suggestion on providing information on agreement across reviewers for the study selection and have added information on Cohen’s Kappa to methods and results section.

In the methods section: “Cohen’s kappa coefficients were computed for titles and abstracts and full-text screening to indicate the agreement between pairs of reviewers.”

In the results section: “The Cohen's Kappa value for title and abstract screening ranged between 0.52 and 0.58 (moderate agreement; depending on the combination of authors involved) and for full text screening was 0.66 (substantial agreement)”.

6. Qualitative studies are only mentioned once in the results - clarify whether these were included but there were none, or whether they were excluded from the start. This could be added to the methods inclusion/exclusion criteria.

According to our eligibility criteria, “qualitative study” was not in itself a criteria for exclusion. However, the qualitative studies found by our search strategy were excluded due to other exclusion criteria (e.g., wrong exposure).

In order to clarify that in the text, we added the following to our methods section: “We included studies if they: […] (iv) were (quantitative or qualitative) observational or intervention studies”.

We also mentioned in our results section: “No qualitative studies were included.”.

7. Sex-specific effects – could you include more detail on the reason for looking at these – why is it useful? Currently, you are mentioning in the methods that you did it when data was available but it is not clear why you did this.

Thank you for noting that. There is clear evidence for gender differences in cardiometabolic disease risk (see: Gerdts & Regitz-Zagrosek, 2019 DOI: 10.1038/s41591-019-0643-8), as well as gender differences in social relations (see: Ridgeway, 2009, https://www.jstor.org/stable/20676769).

Thus, we expect that social environment factors would impact cardiometabolic disease outcomes differently for men and women. We have added an explanation on why we considered sex-specific effect metrics to the methods section. See text below:

"There is clear evidence for sex differences in cardiometabolic disease risk (Gerdts & Regitz-Zagrosek, 2019), as well as in social relations (Ridgeway, 2009). We therefore extracted data on sex-specific effect metrics, when available."

8. In the discussion you mention the commonly observed outcomes – it would be helpful to see what are the % and n of studies that looked at these most common outcomes.

Thank you for noting that. We have adjusted the text in the discussion section and included quantitative information on the associations per outcome. The text is now: "Among the CMD outcomes considered, type 2 diabetes mellitus and hypertensive diseases were the only outcomes investigated in association with all dimensions of the social environment (34% and 25% of all associations included, respectively). Other commonly observed outcomes were and ischemic heart disease (17%) and stroke (11%), which were both investigated in association with all dimensions except Disorder and Incivilities."

9. Throughout, you talk about 'worse' social environments – it would be good to see this defined somewhere, what you consider to be a worse social environment and include some examples.

We thank the reviewer for this suggestion. We have added a clarification to the Data synthesis section and in the Meta-analysis section, where we explained how we defined the "best-off" reference categories, we reduced the text to avoid repetition.

See the changes below:

Data synthesis: "We classified exposure categories as indicating "better" and "worse" social environment. For instance, "worse" social environment for the dimension Economic and Social Disadvantage was the group experiencing the most deprivation and "better" social environment for the dimension Social Relationships and Norms was the category with the most social support."

Meta-analysis: "The exposures reference categories were harmonized and defined as the "best-off" category. For instance, in the dimension Economic and Social Disadvantage, the reference category for a social environmental factor like "deprivation" was "the least deprived group", and in the dimension Social Relationships and Norms, the reference category for a factor like "social support" was "the group with the most social support"."

10. Discussion: You have mixed the strengths and limitations of your included studies with the strengths and limitations of your methodology. For example, in the paragraph about your study's strengths and limitations, you mention that the majority of studies were conducted in high income countries – was this a limitation of your review or of the evidence? Was this a limitation due to your imposed exclusion criteria or the available evidence? It may be useful to clearly distinguish these in different paragraphs in your discussion.

We thank the reviewer for noting that. The limitation mentioned (i.e., the majority of studies included were conducted in high income countries) could be a limitation of our review and/or of the evidence, i.e., it could have originated from a gap in the literature or resulted from our exclusion criteria, which restricted the review to papers written in English. Nonetheless, we have further reflected on this limitation and determined that, considering that we excluded only 11 records based on the language criteria (of the studies assessed for eligibility), the lack of studies conducted in LMIC in our review is mainly attributed to an actual gap in the literature and rather due to our language criteria. Therefore, we no longer mention it as a limitation within strengths and limitations of your methodology.

11. It would be helpful to add some more detail in the discussion about the implications of your research in addition to your suggestions for future research.

Thank you for the suggestion. Indeed, there are potential implications for public policy, which we now mention in the Discussion section.

“Translating our findings to policy implications is notoriously challenging, especially given the largely observational nature of the evidence. Nevertheless, we demonstrated consistent evidence on the association between economic disadvantage and increased CMD risk, which reinforces the clear health potential of interventions and policies aimed at reducing poverty and deprivation. For instance, the Moving To Opportunity study successfully demonstrated that moving from high-poverty to low-poverty neighborhoods reduced the risk of type 2 diabetes after 10-15 years (47). In addition, public policies and interventions should target social and norms, for instance through early-life or marital social relationship interventions(89, 90) and through the screening of social circumstances (e.g., “polysocial risk scores” (91) or psychosocial screening protocols (92)) within the healthcare context. Yet, it may be difficult to put social relationships high on the list of public health priorities(93).”

Reviewer: 2

Dr. Demy Idema, University Medical Centre Utrecht

Comments to the Author:

First, we would like to congratulate the authors on the extensive work they have done on this interesting subject. We have some comments that we have divided into the sections of the manuscript that they apply to.

Second, I would like to mention that I have performed this peer-review together with my colleague Linde Huis in ‘t Veld.

We thank the reviewers for taking the time to evaluate our systematic review and meta-analysis. We appreciate the constructive feedback provided to improve the manuscript. We have carefully considered and responded to the reviewer’s comments in detail below (written in blue).

As you and the other reviewer rightly indicated, this is a quickly developing field and literature reviews are especially time-sensitive in nature. We have therefore conducted an extensive update of the literature review, yielding 73 additionally included papers. We hope that the revisions we made to the manuscript are satisfactorily and allow for a swift publication of the manuscript.

General remarks

1) The search was performed in February 2021, and as the authors stated themselves, research on some of the social environment dimensions is only recently emerging, so it might be worthwhile to update the search before publication.

We acknowledge the importance of staying up-to-date with the latest research in the field and agree with the reviewer's comments. Therefore, we have updated the study and performed a new search in February 28th 2024.

Changes related to the update search were made throughout the manuscript and the update review process is visually depicted in the PRISMA flowchart.

We highlight that while 73 new articles were added to the review, in general, we did not find different trends in terms of associations explored, direction of the associations, methodological quality and geographical distribution of studies. Thus, the update of the review resulting in the addition of new studies did not impact our overall conclusions.

2) We think it would be good to add an appendix with the list of studies that were excluded during full text screening with reasons for exclusion.

We have created the Supplementary File 2, which contains a list of papers assessed for eligibility (full-text screening), with the reason for exclusion.

3) There is no mention of assessment of publication bias, or why assessment would not be possible.

We appreciate the comment and the agree with the reviewers on the importance of assessing publication bias. Therefore, we have generated funnel plots for the overall meta-analysis. We would like to mention that performing quantitative tests for publication bias (e.g., Begg test, and Egger's test) is not yet currently supported by the metafor package in R for models performed with the function rma.mv (used for multi-level meta-analysis). Therefore, we have assessed publication bias through funnel plots only.

We mentioned the funnel plots throughout the text:

In the methods: "Funnel plots were generated to qualitatively assess publication bias. We examined funnel plots when at least five exposure-outcome combinations were available and followed the same criteria applied to inclusion of associations in the meta-analysis."

In the results: "We found substantial asymmetry in the funnel plots (n = 9), particularly for some exposure-outcome combinations, for instance Economic and Social Disadvantage and hypertensive diseases (Supplementary Figure 2A-2I). This may suggest publication bias but may also reflect study heterogeneity (Page, 2019).

4) Have you considered doing a certainty assessment for the investigated associations, for example with GRADE?

Indeed we have considered employing GRADE but after careful consideration decided against it given i) the specific expertise needed to conduct the GRADE process, ii) the large volume of evidence to be evaluated, iii) the low number of intervention studies included in our review, and iv) the tool may penalize excessively observational studies. Our research group has not adopted the GRADE certainty assessment and therefore we lack the expertise to conduct a reliable and stable certainty assessment. In addition, given the 281 included studies, with a lot of heterogeneity in study design and focus, we opted for a more straightforward quality assessment tool. Finally, GRADE is especially helpful when recommendations are to be made in favor or against an intervention. However, our systematic review mostly included observational studies and is not evaluating effectiveness of an intervention.

Abstract

5) Page 3, line 18: Starting the methods section with “we performed a systematic review and meta-analysis…” seems to be double considering the design mentioned above.

We appreciate the opportunity to clarify the text. We have adjusted the text in the abstract to avoid the repetition and to meet the journal’s guideline.

6) The order in which the methods are presented in the abstract are not in line with the methods section. It would make more sense to mention quality assessment after data extraction, and there is no mention of full text screening in the abstract.

The abstract was adjusted to align with the methods section and to meet the journal’s guideline (i.e., adding specific sections).

7) Page 3, line 43: In the sentence “Meta-analyzed sex-specific associations consistently showed results in the same direction.”, it is unclear to us whether you mean the same direction as each other (i.e. the same direction for both sexes) or the same direction as the main effect (or both).

Thank you for noticing this. Although the comparison between men and women is also relevant, due to limit word count, in the abstract we meant that sex-specific associations were consistent with the main effect. We have re-written the sentence to clarify: “…meta-analyzed sex-specific associations consistently showed results in the same direction as the overall meta-analysis. ”

Strengths and limitations of this study

8) Page 4, line 12: Would it be possible to elaborate on this sentence to make it clearer: “The broad scope of the search strategy helps to mitigate the impact of heterogeneity on social environmental factors on the retrieval and synthesizes of findings;” Wouldn’t a narrower search where you identify factors that are more similar yield an analysis with less heterogeneity?

We thank the reviewers for the opportunity to clarify the sentence. We agree that this phrasing is confusing and have rephrased as follows:

“The broad scope of the search strategy acknowledges the different dimensions of the social environment that cannot be (re)viewed in isolation.”

Introduction

9) Page 5, line 16: You mention that there is a reduction in the burden of CMD in high-income countries and a disproportionate burden in low- and middle- income countries, could you elaborate on why you decided to not specifically focus on low- and middle-income countries and rather included all settings?

Thank you for this question. We chose not to focus specifically on low- and middle-income countries because a systematic overview of the associations between social environment and CMD risk was also not available for high-income countries. Moreover, not restricting our review to countries based on their income allows us to make comparisons across countries, and with the updated search, we were now able to further stratify our meta-analyses by country-level income.

Methods

10) Page 7, line 20: You mention that you divided the results into references with ‘hard outcomes’ and ‘risk factors’ as outcomes, but from this part of the text it is not yet clear what exactly you mean with risk factors. The term in this context is a bit confusing with the risk factors mentioned in the introduction at the environmental level (i.e. the social environment dimensions).

Thank you for pointing that out. We clarified what we mean by ‘risk factors’ with examples. See text: “(…) “risk factor” for cardiometabolic disease (e.g., glycated hemoglobin, blood lipids, and metabolic syndrome)”

11) Page 7, line 48: You mention that you use the mobile app Covidence to ‘expedite the screening process’, could you explain what you mean by this? Did you only use Covidence for systematic review management or also used any machine learning tools in the screening process?

We would like to clarify that we used the mobile apps Covidence and Rayyan (the latter only used for the search update) for management and organization of the screening process only. No machine learning tools were employed. We clarified that in the text: “We used the mobile apps Covidence and Rayyan to manage and organize the screening process”.

12) Page 8, line 14: You have explained that you do not include CMD with little or no influence of lifestyle behaviors, but we miss an explanation for why you have not included non-alcoholic fatty liver disease, which seems to us as a type of CMD that does have lifestyle influence. Could you elaborate?

Thank you for the opportunity to clarify. While non-alcoholic fatty liver disease is indeed linked to lifestyle behaviours and has receiving increasing attention, we chose to focus on more well-established and widely acknowledged cardiometabolic diseases, for which we expected to find a more robust body of evidence.

13) Page 8, line 41 and Page 9, line 5: Data extraction and quality assessment was performed by one author, with a second author checking a random 10% sample, but what was the agreement (e.g. kappa score) between the two reviewers?

Regarding the agreement for data extraction and quality assessment, because the second author only checked a random 10% sample, but did not independently conduct the data extraction and quality assessment procedure in duplicate, it was not possible to quantify agreement. We have clarified that in the Methods section. See below:

"A second author (JM) qualitatively appraised the data extraction of a random subsample of 10% of the studies included; any inconsistencies were discussed and agreement reached."

The agreement between pairs of reviewers for title and abstract and full-text screening was added to the Results section (Cohen's Kappa coefficients indicated moderate to substantial agreement).

And about Cohen's Kappa coefficients:

In the methods section: "Cohen's kappa coefficients were computed for titles and abstracts and full-text screening to indicate the agreement between pairs of reviewers."

In the results section: "The Cohen's Kappa value for title and abstract screening ranged between 0.52 and 0.58 (moderate agreement; depending on the combination of authors involved) and for full text screening was 0.66 (substantial agreement)".

14) Page 9, line 45: You state that low quality studies (that got <50% of available points) were excluded from the vote count and meta-analyses, but why did you choose this value in particular? And have you considered including all studies but doing a sensitivity analysis without all the low-quality (or high risk of bias) studies excluded?

We would like to clarify that since no threshold for overall study quality is established for the New Castle-Ottawa Scale (NOS) and there is no widely accepted value, we decided on threshold of 50% of available points.

Regarding the comment about sensitivity analysis, we agree and thank the reviewers for the suggestion. In order to explore the impact of not excluding low quality studies from the analysis, we performed sensitivity analysis for the two most commonly examined combinations of exposure-outcome (i.e., Economic and Social Disadvantage and hypertensive diseases and Economic and Social Disadvantage and type 2 diabetes mellitus).

Sensitivity analysis showed no major differences compared to the main meta-analysis, despite the increase in the number of associations included (i.e., Economic and Social Disadvantage and type 2 diabetes mellitus: OR 1.19, 95%CI 1.05-1.36; n=40; I2=95% and Economic and Social Disadvantage and hypertensive diseases: OR 1.18, 95%CI 1.03-1.34; n=35; I2=99%). This suggests that our conclusions are robust to the inclusion of low quality studies.

We mention the sensitivity analysis in the methods and results section.

15) Are the included effect measures from the primary studies adjusted for any variables? Or all they all from univariate analyses?

Thank you for this question. Adjustment of effect measures from the primary studies for covariates varied per study. We extracted data from the model in which effect measures were adjusted to the highest degree (i.e., fully adjusted models), except when adjustment was made for covariates considered potential mediators (e.g., lifestyle behaviors). If a study did not provide sufficient adjustment (i.e., for the variables age, sex and any socioeconomic indicator), it was penalized by the quality assessment tool. We have also described this in the Methods section as follows:

“We extracted data from the model in which effect measures were adjusted to the highest degree (i.e., fully adjusted models), except in cases where adjustments were made for potential confounders (e.g., lifestyle behaviors).”

Results

16) Page 12, line 43 and Page 13, line 10: You don’t mention that vote counting was not possible for associations with the social environment dimension ‘Discrimination and segregation’. You explain why in the discussion, but without any disclaimer in the results, the statement that “all social environment dimensions were found […] except for Sense of Place/Belonging” is not true.

We have adjusted the text to clarify the exclusion of the social environment dimension ‘Discrimination and segregation’ from the vote counting and meta-analysis.

Besides the text in the Methods section:

“In both methods, we did not include associations from low quality studies and associations from the dimension Discrimination and Segregation (e.g., proportion of ethnic minority in a neighborhood). The latter was excluded because its exposure measures were highly heterogeneous in operationalization and poorly defined, and thus the associations were not comparable for vote counting and meta-analysis purposes.”,

see text added regarding vote counting in the Results section:

“All social environment dimensions were found in association with at least one outcome, except for (…) the dimension Discrimination and Segregation, which was not included in the vote counting.”

17) Page 13, line 10: Same as comment 16 above.

We have adjusted the text to clarify the exclusion of the social environment dimension ‘Discrimination and segregation’ from the vote counting and meta-analysis.

Besides the text in the Methods section:

“In both methods, we did not include associations from low quality studies and associations from the dimension Discrimination and Segregation (e.g., proportion of ethnic minority in a neighborhood). The latter was excluded because its exposure measures were highly heterogeneous in operationalization and poorly defined, and thus the associations were not comparable for vote counting and meta-analysis purposes.”,

see text added regarding meta-analysis in the Results section:

“All social environment dimensions were meta-analyzed in relation to at least one outcome, except for (…) the dimension Discrimination and Segregation, which was a priori excluded for the meta-analysis.”

18) Page 13, line 20: “We observed in all 14 meta-analyses that worse social environment was associated with increased risk of CMD outcomes”. We think you could be more careful in making this statement when there is no statistical significance for 10 of these results.

Thank you for this comment. We have adjusted and nuanced the text to reflect the lack of statistical significance.

“We observed an overall trend suggesting that worse social environment was associated with increased risk of CMD outcomes in all but one meta-analysis (i.e., Social Cohesion and Social Capital and hypertensive diseases). Despite this tendency, only four out of 14 meta-analyses were statistically significant.”

19) We also wonder whether you have considered if the statistically significant results are clinically significant? Have you tried identifying a minimally clinically important difference for example?

We agree with the reviewers on the importance of interpreting findings in light of their clinical relevance. However, unfortunately this was not possible due to the heterogeneity observed in the exposure classifications, and the lack of common measurement units makes it challenging to determine their clinical significance. Furthermore, the exposures we identified do not conform in a typically linear or ordinal scale. Thus, we put emphasis on examining the direction of effects and the strength of the associations identified.

20) Page 13, line 39: (Almost) all meta-analyses have very high heterogeneity, have you considered to not pool the data and present it in another way? Or maybe explore the heterogeneity a bit more with subgroup analyses or meta-regression?

We did consider another way to present the data. Because of the broad and complex research question, and the resulting heterogeneous data, we have employed the vote counting method (harvest plots) to synthesize and present the data in an alternative way. In addition, we examined the data in sub-groups, for instance by sex, country income, effect metric and quality. We performed sub-group/sensitivity analysis as much as possible while maintaining the manuscript’s readability.

Discussion

21) Page 15, line 28: In the third paragraph you go back to the main finding of the study, which may fit better at the start of the discussion.

We thank the reviewers for the suggestion. We have adjusted the order of the paragraphs in the discussion accordingly.

22) Page 15, line 43: “One could argue that living in a deprived neighborhood or a lack of social relationships leads to stress, rising blood pressure and hypertensive diseases “directly” – resulting in larger effect sizes - whereas the level of civic participation may lead to stress via reduced social cohesion only – resulting in smaller effect sizes. However, we found no indication for this explanation.” We were curious whether you have found this effect to hold true in other literature?

This is an interesting topic for discussion. While we argue it is expected that some social environmental factors can influence health outcomes more directly than others, there is little research comparing the magnitude of effects of different social environmental factors on a given health outcome, while accounting for direct/indirect pathways and potential mediators. For instance, one may hypothesize that while social support may exert a strong direct effect on mental health outcomes (e.g., well-being), other social environmental factors may influence mental health outcomes predominantly indirectly (e.g., living in a racially segregated neighborhood may reduce social cohesion, which can increase psychological distress, and therefore affect well-being indirectly).

23) Page 17, line 9: In the discussion you mention that it may be better to use longitudinal studies to study the effect of cumulative exposure; have you considered performing a sensitivity analysis for only the longitudinal studies to see if this affects your outcome?

We agree and thank the reviewers for the suggestion. Therefore, we performed sensitivity analysis including only longitudinal studies. The findings suggest that our conclusions are robust to different study designs. We mentioned the sensitivity analysis in the Methods, Results and Discussion sections.

Methods section:

“We performed sensitivity analysis […] ii) including only longitudinal studies […].”

Results section:

“For sensitivity analysis of longitudinal studies (n=11 meta-analyses; Supplementary Table 4), no major differences in pooled effects in comparison with the meta-analysis including both longitudinal and observational studies were observed. Three out of eleven studies differed more than 10% from the respective overall effect, showing attenuated effects. Three out of four pooled effects that were statistically significant in the main meta-analysis remained statistically significant.”

Discussion section:

“Indeed, included longitudinal studies were generally of better quality than the cross-sectional studies; however, for the exposure-outcome combinations where sensitivity analysis could be conducted exclusively using longitudinal studies, we did not observed marked differences.”

Table 1

24) In the characteristics of studies table there is a lot of ‘not applicable’ filled in for average age and % women for example, but it seems to us that you mean ‘not reported’?

We thank the reviewers for noticing this. We have adjusted the table accordingly.

VERSION 2 - REVIEW

**REVIEWER NAME**	Braune, Tanya
**REVIEWER AFFILIATION**	University of Cambridge, MRC Epidemiology Unit
**REVIEWER CONFLICT OF INTEREST**	No competing interests
**DATE REVIEW RETURNED**	25-Jun-2024

**GENERAL COMMENTS**	Thank you for sharing the updated manuscript. The authors have conducted a considerable amount of work to address the issues raised by the reviewers and editor and I feel that it will be a useful contribution to the knowledge base in this field.

**REVIEWER NAME**	Idema, Demy
**REVIEWER AFFILIATION**	University Medical Centre Utrecht, Julius Center for Health Sciences and Primary Care
**REVIEWER CONFLICT OF INTEREST**	None
**DATE REVIEW RETURNED**	24-Jun-2024

**GENERAL COMMENTS**	We would like to thank the authors for the extensive and clear responses to our comments and the extra work they performed to update the search. We only have several small comments on this new version. I again like to acknowledge that I performed this review with my colleague Linde Huis in 't Veld.<br><br>1. In the methods section we noticed that you chose to mention both the original search and the updated search, but since all results are combined and not available separately (for example in a previous publication), we wonder whether it would not be better to just mention that you searched from inception until 29 February 2024 (the same holds true for the flow diagram).<br><br>2. A very short note accompanying this, is that the supplementary file with the full search strategy still has the original search date mentioned in it, not the new search date.<br><br>3. In the data extraction and quality assessment sections of the methods, it is now mentioned that a second author ‘qualitatively’ appraised the data extraction. As you have specifically added

	‘qualitatively’ to this statement, can you explain what you mean by this.<br><br>4. In the data extraction section of the methods you explain that you categorise social environment into eight factors, and proceed by giving an example of each. But you seem to be missing one of them (‘Sense of place/belonging’) here, was this a purposeful choice, or missed by accident?

VERSION 2 – AUTHOR RESPONSE

Reviewer: 2

Dr. Demy Idema, University Medical Centre Utrecht

Comments to the Author:

We would like to thank the authors for the extensive and clear responses to our comments and the extra work they performed to update the search. We only have several small comments on this new version. I again like to acknowledge that I performed this review with my colleague Linde Huis in 't Veld.

We thank the reviewers for taking the time to evaluate our systematic review and meta-analysis. We appreciate the additional constructive feedback provided to improve the manuscript. We have carefully considered and responded to the reviewer’s comments in detail below (written in blue).

1. In the methods section we noticed that you chose to mention both the original search and the updated search, but since all results are combined and not available separately (for example in a previous publication), we wonder whether it would not be better to just mention that you searched from inception until 29 February 2024 (the same holds true for the flow diagram).

We agree with the suggestion given by the reviewers and we now mention only the latest searched (29 February 2024). We have updated the flow diagram (see Figure 2) and the manuscript accordingly.

2. A very short note accompanying this, is that the supplementary file with the full search strategy still has the original search date mentioned in it, not the new search date.

Thank you for pointing this out. We have fixed the date of search in the Supplementary File 1.

3. In the data extraction and quality assessment sections of the methods, it is now mentioned that a second author ‘qualitatively’ appraised the data extraction. As you have specifically added ‘qualitatively’ to this statement, can you explain what you mean by this.

By "qualitatively" we mean that the second reviewer checked the data extraction done by the first reviewer to ensure accuracy and adherence to the review protocol and the pre-defined data extraction form. This appraisal did not generate quantitative metrics, for instance measures of inter-reviewer agreement (e.g., Cohen’s Kappa) and therefore we have mentioned it was "qualitative”. More

specifically, the second reviewer cross-checked and compared the extracted data with the original papers to ensure for instance that the appropriate models and relevant information were correctly identified, and that information was extracted without typos or misinterpretations.

4. In the data extraction section of the methods you explain that you categorise social environment into eight factors, and proceed by giving an example of each. But you seem to be missing one of them ('Sense of place/belonging') here, was this a purposeful choice, or missed by accident?

Thank you for noting that. We have included details for 'Sense of place/belonging' as well. Se text added to the Methods section: "[…] and Sense of Place/Belonging includes place attachment and sense of community."

Reviewer: 1

Dr. Tanya Braune, University of Cambridge

Comments to the Author:

Thank you for sharing the updated manuscript. The authors have conducted a considerable amount of work to address the issues raised by the reviewers and editor and I feel that it will be a useful contribution to the knowledge base in this field.

We thank the reviewer again for taking the time to evaluate our systematic review and meta-analysis. We appreciate the constructive feedback provided to improve the manuscript.

VERSION 3 - REVIEW

**REVIEWER NAME**	Idema, Demy
**REVIEWER AFFILIATION**	University Medical Centre Utrecht, Julius Center for Health Sciences and Primary Care
**REVIEWER CONFLICT OF INTEREST**	None
**DATE REVIEW RETURNED**	01-Jul-2024

**GENERAL COMMENTS**	No remaining comments
